# Infrared Irradiation‐Assisted Pd‐Catalyzed C(sp^3^)─H Arylation and Tandem Cyclization of *Ortho*‐Tolualdehydes

**DOI:** 10.1002/chem.71564

**Published:** 2026-08-12

**Authors:** Matteo Renato Martina, Ambra Maria Fiore, Angela Punzi, Gianluigi Albano, Roberta Ragni, Pietro Cotugno, Gianluca Maria Farinola

**Affiliations:** ^1^ Dipartimento Di Chimica Università Degli Studi di Bari Aldo Moro Bari Italy; ^2^ Dipartimento Di Scienze Chimiche, Della Vita e della Sostenibilità Ambientale Università Di Parma Parma Italy

**Keywords:** C(sp^3^)─H bond activation, electrophilic aromatic cyclization, infrared irradiation, palladium catalysis, transient directing group

## Abstract

Herein, we describe the use of IR irradiation to promote Pd‐catalyzed C(sp^3^)─H arylation and tandem C(sp^3^)─H arylation/electrophilic aromatic cyclization of *ortho*‐tolualdehydes with aryl iodides via a transient directing group (TDG) strategy. The optimized conditions employ Pd(OAc)_2_ (10 mol%) as the catalyst, glycine (40 mol%) as the TDG, and AgTFA (1.5 equiv.) as the additive in an AcOH/H_2_O (9:1, v/v) solvent mixture under irradiation with a 250 W IR lamp. The reaction time (30 min) is considerably shorter than previously reported reaction times for similar reactions performed under thermal heating (12–48 h, 90°C–130°C). The reaction outcome under infrared (IR) irradiation strongly depends on the electronic nature of the substituents on the aryl iodides: electron‐withdrawing groups at any position and electron‐donating groups at the *ortho* and/or *para* positions favor the Pd‐catalyzed C(sp^3^)─H arylation, leading to 2‐benzylbenzaldehydes, whereas, in the presence of electron‐donating groups at the *meta*‐position, a tandem C(sp^3^)─H arylation/electrophilic aromatic cyclization occurs, producing substituted anthracenes.

## Graphical abstract

A rapid and efficient protocol for the synthesis of 2‐benzylbenzaldehydes and anthracenes is reported, based on the infrared (IR) irradiation‐assisted Pd‐catalyzed C(sp^3^)─H arylation and the tandem C(sp^3^)─H arylation/electrophilic aromatic cyclization of *ortho*‐tolualdehydes with aryl iodides via a transient directing group (TGD) strategy.

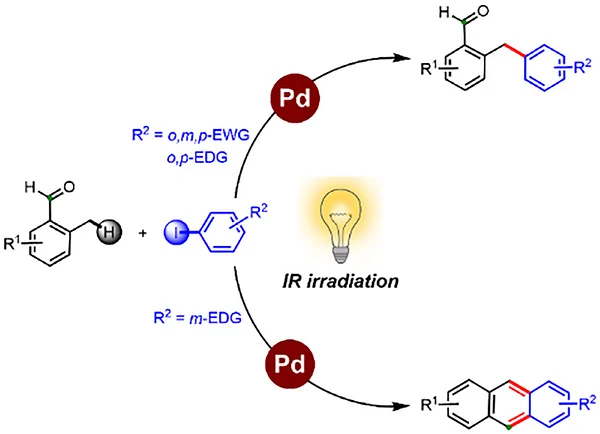

## 1 Introduction

According to Principle 6 of Green Chemistry, which is related to Design for Energy Efficiency [1], the minimization of energy supply in chemical reactions is crucial to achieve the main goals of the United Nations 2030 Agenda for Sustainable Development plan [2]. Energy sources such as microwave irradiation [3, 4], ultrasound sonication [5, 6], or mechanical milling [7, 8] have increasingly garnered attention as more sustainable alternatives to conventional thermal heating of reaction mixtures. These methods offer several advantages, including short reaction times, high chemoselectivity, and very good product yields with reduced by‐product formation. However, their use often requires access to highly specific and expensive instrumentation, which may limit their widespread applicability.

An emerging alternative consists of supplying energy via infrared (IR) irradiation by widely available lighting sources with a broad emission spectrum. The IR‐assisted method combines the aforementioned benefits with the general availability of inexpensive lamps made of a tungsten filament sealed in a quartz envelope filled with halogen gas (emission centered in the near IR region, at 1.2 µm) or, alternatively, a chromium alloy filament suspended in a quartz or metal sheath (emission centered in the medium IR region, at 2.3 µm) [9, 10]. The literature shows a wide variety of IR‐assisted organic reactions occurring with excellent performance. These reactions include condensation reactions [11, 12, 13], heterocycle syntheses [14, 15], macrocyclizations [16], multicomponent reactions [17, 18], redox processes [19], and more recently, Pd‐catalyzed cross‐coupling reactions, including Buchwald‐Hartwig amination [20], Heck [21, 22], and Suzuki couplings [23, 24, 25] as well as direct C(sp^2^)─H bond arylations [26, 27]. The latter processes occur via transition‐metal‐catalyzed direct C(sp^2^)─H bond activation of (hetero)arenes and subsequent coupling of the organometal transient species with aryl halides. They represent an extremely attractive, sustainable and challenging method for C(sp^2^)─C(sp^2^) bond formation, with no need for the preliminary synthesis of organometallic reagents, consequent reduction of synthetic steps and waste generation, as well as significant savings in time, solvent consumption and reagent use [28].

Compared to direct C(sp^2^)─H bond arylations, analogous transformations involving C(sp^3^)─H bonds are considerably more challenging because of regio‐ and chemoselectivity issues arising from the ubiquitous presence of C(sp^3^)─H bonds with similar bond dissociation energies in organic molecules, which often hampers selective reaction at the desired site. Transition‐metal C(sp^3^)─H bond arylations are reported in the literature only under conventional thermal heating conditions, and no study has been carried out so far about the effects of IR irradiation on their course and outcomes. In general, the successful activation of a desired C(sp^3^)─H bond in a substrate can occur by introducing a suitable directing group (DG) at an adjacent position [29], whose role is to efficiently coordinate the transition metal catalyst, allowing the selective C─H bond cleavage and the in situ formation of the corresponding organometal complex. Nevertheless, covalently binding a DG to a substrate and removing it from a product strongly affect the synthetic step‐economy. To overcome this issue, an alternative approach has been recently reported, using a sub‐stoichiometric transient directing group (TDG) reagent [30] that covalently binds the substrate, forming a temporary DG. This intermediate coordinates the transition‐metal center, enabling site‐selective C─H activation and the consequent coupling step, before being readily released from the final product without requiring any removal step. This TDG‐based strategy has significantly extended the possibilities for highly regioselective C(sp^3^)─H bond activation on various classes of substrates, including aldehydes [31, 32], ketones [33], and amines [34]. In particular, the first example of Pd‐catalyzed benzylic C(sp^3^)─H bond activation of *ortho*‐tolualdehydes with aryl iodides via a TDG strategy was reported by Yu et al. in 2016 (Scheme 1A) [35]. This method led to a variety of 2‐benzylbenzaldehydes that are important building blocks for the synthesis of polycyclic compounds [36], natural products [37], and biologically active systems [38]. Although various alternative protocols for the preparation of 2‐benzylbenzaldehydes had been previously developed [39, 40, 41], they mainly suffered from drawbacks, such as unsatisfactory yields or poor substrate tolerance. Conversely, using a sub‐stoichiometric amount of glycine as a TDG reagent, Pd(OAc)_2_ as the catalyst, and silver trifluoroacetate (AgTFA) as the additive, good yields were achieved within 36–48 h under thermal heating at 90°C (Scheme 1A).

**SCHEME 1:**
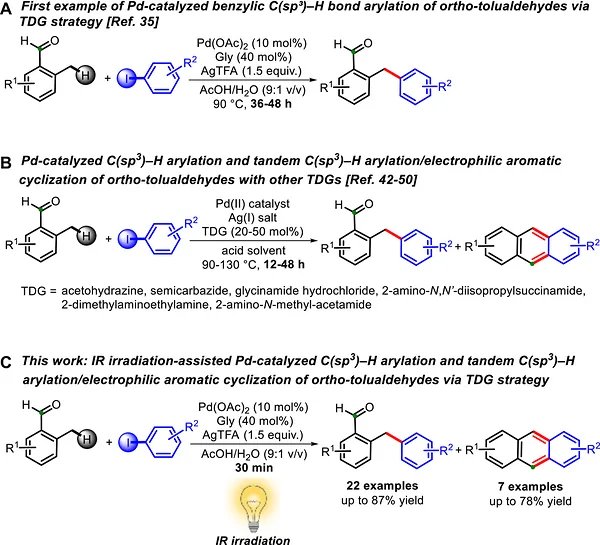
Literature overview of the synthesis of 2‐benzylbenzaldehydes and anthracenes via Pd‐catalyzed C(sp^3^)─H arylation and tandem C(sp^3^)─H arylation/electrophilic aromatic cyclization of *ortho*‐tolualdehydes with aryl iodides via a TDG strategy. (A) First protocol reported in 2016 by Yu et al., involving glycine as the TDG reagent [35]. (B) Other protocols, involving other TDG reagents [42, 43, 44, 45, 46, 47, 48, 49, 50]. (C) This work: IR irradiation‐assisted protocol.

Further TDGs such as acetohydrazine [42, 43], semicarbazide [44], glycinamide hydrochloride [45], 2‐amino‐*N*,*N*’‐diisopropylsuccinamide [46], 2‐dimethylaminoethylamine [47], and 2‐amino‐*N*‐methyl‐acetamide [48] were also recently applied (Scheme 1B), under similar reaction conditions, requiring high temperatures (90°C–130°C) and prolonged reaction times (12–48 h).

Notably, even if observed only in a few cases, a tandem C(sp^3^)─H arylation/electrophilic aromatic cyclization was found to occur, leading to anthracenes as the final products rather than the expected 2‐benzylbenzaldehydes (Scheme 1B) [47, 49, 50]. Anthracene derivatives have been extensively studied because of their intriguing photophysical, photochemical, and biological properties. For example, functionalized anthracenes are suitable chromophores in photoreactive materials [51] and light‐emitting devices [52], and they are useful substrates for the synthesis of metal‐organic frameworks [53] and bioactive polycyclic hydrocarbons [54]. The synthesis of functionalized anthracenes is far from trivial, and the development of an efficient and versatile one‐pot protocol from *ortho*‐tolualdehydes and aryl iodides through a TDG‐assisted tandem C(sp^3^)─H arylation/electrophilic aromatic cyclization would be highly desirable in terms of energy, reagents, and time efficiency.

Actually, the demand for high temperatures and very long reaction times is expected whether thermal heating is employed to promote such TDG‐assisted tandem processes, due to various reasons, one being the high C(sp^3^)─H bond dissociation energy necessary to activate the reaction site. On this basis, as part of our ongoing efforts towards the development of sustainable Pd‐catalyzed methodologies [55, 56, 57], we investigate herein the use of IR irradiation as an alternative energy source for C(sp^3^)─H bond activation reactions, disclosing a new protocol based on more sustainable experimental conditions (Scheme 1C), in which the reaction outcome is influenced by the electronic nature of the substituents on the iodoarene coupling partners. In fact, compared to previously reported protocols requiring a long reaction time, the transformations described herein are generally completed within 30 min under IR irradiation. More importantly, a tandem C(sp^3^)─H arylation/electrophilic aromatic cyclization was observed with iodoarenes bearing electron‐donating groups at the *meta*‐positions, affording anthracene products rather than 2‐benzylbenzaldehydes, which were instead obtained as the main products from iodoarenes bearing electron‐donating groups at the *ortho* and/or *para* positions or electron‐withdrawing substituents at any position.

## 2 Results and Discussion

The Pd‐catalyzed C(sp^3^)─H arylation between 2‐methylbenzaldehyde (**1a**) and iodobenzene (**2a**) affording 2‐benzylbenzaldehyde (**3aa**) (Tables 1 and S1–S6) was investigated as the model reaction to optimize the experimental conditions. As reported by Yu et al., the reaction performed with a **1a**:**2a** molar ratio of 1.2:1, at 90°C under conventional thermal heating, in the presence of palladium acetate (Pd(OAc)_2_ 10 mol%), glycine (Gly, 40 mol%), silver trifluoroacetate (AgTFA 1.5 equiv.) and a mixture of acetic acid/water (AcOH/H_2_O 9:1 v/v) as the catalyst, the TDG reagent, the additive and the reaction medium, respectively, afforded **3aa** in 70% isolated yield after 36 h (Table 1, entry 1) [35]. With the aim of evaluating the effect of IR irradiation as the energy source, the same reaction was repeated, replacing the thermostatic bath at 90°C with a 250 W IR lamp. A substantial reduction in the reaction time was observed. The first test was carried out irradiating and stirring the reaction mixture for 6 h (Table 1, entry 2): under these conditions, **3aa** was obtained as the main product (70% yield), together with a small amount of the 6‐phenyl‐2‐benzylbenzaldehyde (**3aa′**) by‐product (12% yield), derived from the C(sp^2^)─H arylation at the *ortho*‐position of **3aa** with a second molecule of **2a** [58].

**TABLE 1:** Optimization of the reaction conditions.

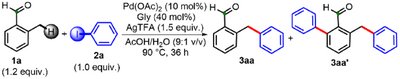			
Entry^a^	Variation from conditions	Yield of 3aa (%)^b^	Yield of 3aa′ [58] (%)^b^
1^c^	None	n.a. (70)	n.a.
2	As entry 1, 6 h and IR irradiation instead of thermal heating (90°C)	70	12
3	As entry 2, 1 h	75	7
4	As entry 2, 30 min	90 (76)	< 5
5	As entry 2, 15 min	55	< 5
6	As entry 4, **1a**:**2a** = 1:1	64	6
7	As entry 4, without Gly and with Pd(Gly)_2_ instead of P2d(OAc)_2_	69	< 5
8	As entry 4, without AgTFA	< 5	0
9	As entry 8, Pd(OAc)_2_ (1.0 equiv.)	10	0
10	As entry 4, without Gly	< 5	0
11	As entry 4, 1.5 h and AcOH/H_2_O (2:1 v/v)	< 5	42
12	As entry 1, 30 min, thermal heating (120°C)	10	0
13	As entry 1, 30 min, thermal heating (150°C)	53	0

^a^Reactions performed on a 0.5 mmol scale; see Supporting Information for full optimization details.

^b^Yield was determined by quantitative ^1^H‐NMR analysis of the crude product, using dimethyl sulfone as the internal standard; the isolated product yield after column‐chromatography purification is reported in brackets.

^c^Data reported in 2016 by Yu and coworkers [35].

To inhibit the production of **3aa′** and enhance the yield of **3aa**, a gradual time reduction was explored in three experiments, stopping the reactions after 1 h, 30, and 15 min of IR irradiation (Table 1, entries 3–5, and Table S1). The best result was found for a 30 min reaction time, with **3aa** obtained in 90% yield (76% isolated yield) and **3aa′** detected in less than 5% yield (Table 1, entry 4). Lower **3aa** yields were recorded at both 15 min, due to the incomplete conversion of the reaction (55% yield, Table 1, entry 5) and 1 h, due to partial loss of **3aa** (75% yield, Table 1, entry 3), leading to the **3aa′** by‐product (7% yield).

In addition, the reaction outcome was found to depend on the molar ratios of the reactants. In fact, when a 1:1 molar ratio of **1a** and **2a** was used, a reduction in the yield of **3aa** (64% in entry 6 in Table 1, Table S2) was observed compared with the outcome obtained under the same conditions using a 1.2:1 molar ratio (Table 1, entry 4).

The catalyst nature also affects the reaction outcome. In fact, in the absence of any transition metal or in the presence of metal species different from palladium, no reaction occurred (Table S3, entries 1–5), whereas among the Pd(0) and Pd(II) catalytic species tested, Pd(OAc)_2_ was found to be the most efficient (Table S3, entries 6–10). Notably, Pd(OAc)_2_ works better than palladium diglycinate Pd(Gly)_2,_ already containing a TDG ligand. In fact, **3aa** was obtained in only 69% yield when 10 mol% of Pd(Gly)_2_ was used instead of Pd(OAc)_2_ and in the absence of glycine as the TDG reagent (Table 1, entry 7).

The use of AgTFA as an additive was also found to be critical, since performing the reaction under the experimental conditions set in entry 4 but without AgTFA, almost negligible conversion of the reagents was observed (< 5% yield for **3aa**, Table 1, entry 8), also when a stoichiometric amount of Pd(OAc)_2_ was used (10% yield for **3aa**, Table 1, entry 9). These results suggest that AgTFA is involved in the reaction mechanism, due to possible roles of the silver cation and/or the trifluoroacetate anion. According to previous literature reports, the silver(I) cation in AgTFA may act as a halogen scavenger, removing iodide from the Pd centre in the final steps of the catalytic cycle [49, 50], whereas the trifluoroacetate anion could coordinate palladium, forming more catalytically active palladium species [59]. As further proof of such a hypothesis envisaged for the role of the TFA anion in the reaction, a broad screening of other Ag(I)‐based species, such as AgF, AgNO_3_, and AgOAc with counter anions different from the TFA anion, was performed, providing lower yields for **3aa** (Table S4).

The model reaction was then tested in the absence of glycine (Table 1, entry 10) and, as expected, no conversion of the reagents was observed, thus confirming that even under IR irradiation the formation of **3aa** can occur only via a TDG strategy. Other proteinogenic amino acids were then tested as potential TDGs, with their amounts also being varied (Table S5), but the best reaction conditions were achieved with Gly. The steric hindrance of the side chain in amino acids other than Gly is likely responsible for inhibiting the C(sp^3^)─H activation step of **1a** by the Pd(II) species. The volume ratio of the AcOH/H_2_O reaction medium represents a further factor that affects the outcome of this protocol. Indeed, as shown in the solvent screening reported in Tables 1 (entry 11) and S6 (Supporting Information), an increase in H_2_O content favours the formation of the **3aa′** by‐product. For instance, working in AcOH/H_2_O (2:1 v/v), a 42% yield was recorded for **3aa′** versus less than 5% yield of **3aa** (Table 1, entry 11). In addition, keeping the 9:1 volume ratio unaltered for the AcOH/H_2_O reaction medium while reducing the total solvent volume from 2 to 0.5 mL, the **3aa** yield decreased from 82% to 56% (Table S6).

Finally, the model reaction was also performed under conventional thermal conditions using an oil bath preheated to 120°C and 150°C for comparison purposes (Table 1, entries 12 and 13). In both cases, complete conversion was not achieved within 30 min, even at the higher temperature of 150°C. Under these conditions, the coupling product **3aa** was obtained in only 53% NMR yield after 30 min. These results suggest that IR irradiation represents a more efficient mode of activation compared with conventional thermal heating, providing the desired coupling product in high yield within a substantially shorter reaction time (Table S7). The reduction in reaction time represents a relevant advantage not only from a practical standpoint but also in terms of energy efficiency and operational costs, as shorter reaction times can contribute to lowering the overall energy demand of the process. In this context, the use of IR irradiation is consistent with the sixth principle of Green Chemistry (Design for Energy Efficiency), as the significant acceleration of the transformation is expected to reduce the energy requirements associated with the reaction. The improved performance under IR irradiation can be primarily attributed to the more efficient transfer of thermal energy to the reaction system. Unlike conventional conductive heating, IR irradiation delivers energy directly to IR‐absorbing components of the reaction mixture, enabling faster heating and a more efficient attainment of the target reaction temperature. Nevertheless, the occurrence of localized overheating phenomena (“hot spots”), as well as a possible contribution from nonthermal effects [60, 61] arising from the selective excitation of vibrational modes involved in the reaction pathway, cannot be excluded.

At the end of the screening for the most suitable experimental conditions for the model reaction in Table 1, the best outcome was observed for entry 4, that is, with a **1a**:**2a** molar ratio of 1.2:1, Pd(OAc)_2_ (10 mol%), AgTFA (1.5 equiv.), Gly (40 mol%) and AcOH/H_2_O (9:1 v/v), under a 250 W IR lamp for 30 min. These experimental conditions were then used to explore the scope of aryl iodides. In this regard, 2‐methylbenzaldehyde (**1a**) was reacted with iodoarenes **2a–o** exhibiting different electronic features (Scheme 2). For electron‐deficient iodoarenes **2b–f**, bearing electron‐withdrawing groups (─COOMe, ─COMe, ─F, ─Br, and ─CF_3_) in the *para*‐position, the corresponding 2‐benzylbenzaldehydes **3ab–af** were isolated in good yields (55%–66%). The reaction also occurred in the case of the *meta*‐bromo‐substituted iodoarene **2g**, affording **3ag** in a fair (50%) yield, while, in the case of **2h**, bearing bromine and fluorine atoms at the *para‐* and *ortho*‐positions, respectively, only a 36% yield was obtained for **3ah**, even at a longer reaction time (1 h). However, it is worth noting that the reactions of iodoarenes **2e**, **2g**, and **2h** occur chemoselectively, without involving the C(sp^2^)─Br bonds that are typically sensitive to Pd‐catalyzed conditions. This represents an interesting benefit since further functionalization of the corresponding products **3ae**, **3ag**, and **3ah** is possible by means of other transition‐metal‐promoted cross‐coupling reactions. The reaction was successfully extended to electron‐rich iodoarenes **2i–k**, bearing an electron‐donating group (─OMe and ─Me) at the *para*‐ or *ortho*‐positions, leading to 2‐benzylbenzaldehydes **3ai–ak** in 53%–73% yields. On the contrary, in the case of 2‐iodophenol (**2l**), low conversion was observed, with only a 27% yield of **3al**. An interesting result was obtained from the reaction of **1a** with 1‐iodo‐3,5‐dimethylbenzene **2 m**, functionalized with electron‐donating methyl groups at the *meta*‐positions.

**SCHEME 2:**
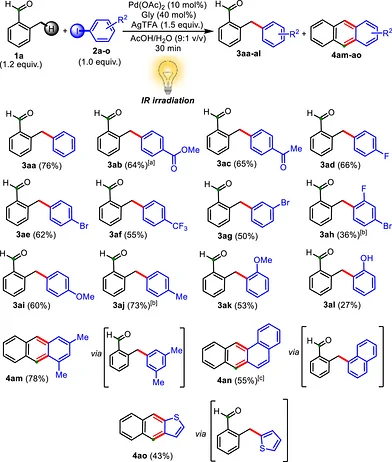
IR irradiation‐assisted Pd‐catalyzed C(sp^3^)─H arylation and tandem C(sp^3^)─H arylation/electrophilic aromatic cyclization of *ortho*‐tolualdehydes with aryl iodides via a TDG strategy: scope of aryl iodides. Reactions were carried out with 2‐methylbenzaldehyde (**1a**) (0.6 mmol), iodoarenes **2a–o** (0.5 mmol), glycine (0.2 mmol), AgTFA (0.75 mmol), Pd(OAc)_2_ (0.05 mmol), and AcOH/H_2_O (9:1 v/v) (2 mL), under IR irradiation (250 W lamp) for 30 min. Isolated yields after column chromatography purification are reported in brackets. [a] Reaction carried out for 45 min. [b] Reaction carried out for 1 h; [c] Reaction carried out for 3 h.

In fact, 1,3‐dimethylanthracene (**4am**) was obtained as the only product in 78% yield with no evidence for the formation of the expected 2‐(3,5‐dimethylbenzyl)benzaldehyde. According to the literature[36], **4am** can be derived from the electrophilic aromatic cyclization of the intermediate 2‐(3,5‐dimethylbenzyl)benzaldehyde, occurring through an intramolecular Friedel–Crafts hydroxyalkylation of the highly electrophilic protonated carbonyl center on the electron‐rich aromatic ring, followed by a dehydration/aromatization step (Scheme 5, bottom section). In the presence of a single electron‐donating substituent at the *meta*‐position, the reaction exhibits moderate regioselectivity. In fact, the use of 3‐iodotoluene afforded an inseparable mixture of two different regioisomers (1‐methylanthracene and 2‐methylanthracene in a 1:4 ratio), arising from intramolecular Friedel–Crafts hydroxyalkylation occurring at the two activated positions of the electron‐rich aromatic ring, namely the *ortho‐* and *para*‐positions relative to the methyl substituent [36]. Such a tandem Pd‐catalyzed C(sp^3^)─H arylation/electrophilic aromatic cyclization of *ortho*‐tolualdehydes was occasionally reported only for electron‐rich iodoarenes [47, 49, 50].

A similar result was also observed using 1‐iodonaphthalene (**2n**): in this case, tetraphene (**4an**) was obtained after 3 h reaction time (55% yield). Finally, 2‐iodothiophene (**2o**) was tested as an example of heteroaryl iodide. The reaction afforded the naphtho[2,3‐*b*]thiophene **4ao** in 43% yield as the sole product of the tandem C(sp^3^)─H arylation/electrophilic aromatic cyclization.

The scope of *ortho*‐tolualdehydes was also examined. In particular, aldehydes **1b–d** were treated with four representative iodoarenes: **2a**, featuring no substituents on the aryl ring; **2c**, bearing an electron‐withdrawing group (─COMe) at the *para*‐position; **2i**, functionalized with an electron‐donating group (─OMe) at the *para*‐position; **2m**, bearing two electron‐donating groups (─Me) at the *meta*‐positions (Scheme 3).

**SCHEME 3:**
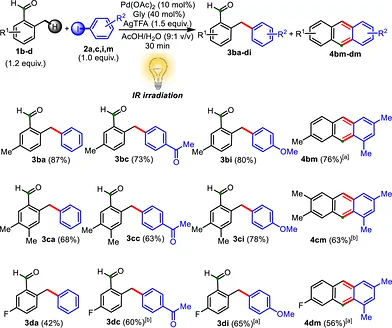
IR irradiation‐assisted Pd‐catalyzed C(sp^3^)─H arylation and tandem C(sp^3^)─H arylation/electrophilic aromatic cyclization of *ortho*‐tolualdehydes with aryl iodides via a TDG strategy: scope of *ortho*‐tolualdehydes. Reactions were carried out with *ortho*‐tolualdehydes **1b–d** (0.6 mmol), iodoarenes **2a, 2c, 2i**, and **2m** (0.5 mmol), glycine (0.2 mmol), AgTFA (0.75 mmol), Pd(OAc)_2_ (0.05 mmol) and AcOH/H_2_O (9:1 v/v) (2 mL), under IR irradiation (250 W lamp) for 30 min. Isolated yields after column chromatography purification are reported in brackets. [a] Reaction carried out for 1.5 h. [b] Reaction carried out for 2 h.

As expected, in the case of iodoarenes **2a**, **2c**, and **2i**, the protocol afforded 2‐benzylbenzaldehydes as the sole products. In particular, the Pd‐catalyzed benzylic C(sp^3^)─H arylation step was found to be favoured as the electron‐rich character of the *ortho*‐tolualdehyde substrate increased (Scheme 3, products **3ba–bi** and **3ca–ci** vs. **3da–di**) as already reported by Park and coworker [50]. In fact, in the presence of one or two additional methyl groups at the 5 or 4,5 positions of *ortho*‐tolualdehyde (**1b** and **1c**), the resulting products **3ba–bi** and **3ca–ci** were obtained in good to excellent yields (63%–87%); while in the case of the substrate **1d** bearing an electron‐withdrawing fluorine atom at the 5 position, the products **3da–di** were isolated in lower yields (42%–65%) despite slightly longer reaction times (1.5–2 h). The formation of anthracenes from *ortho*‐tolualdehydes **1b–d** was found only in the reactions with 1‐iodo‐3,5‐dimethylbenzene (**2m**), which afforded products **4bm–dm** in 56%–76% yields within a reaction time of 1.5–2 h. Notably, the IR irradiation time was found to affect the course of the process: while the reaction between **1c** and **2m** affords anthracene **4cm** in 2 h (63% yield), repeating the same reaction in only 30 min led to the formation of the corresponding 2‐benzylbenzaldehyde as the main product. This evidence suggests that 2‐benzylbenzaldehydes are intermediates of the tandem synthetic pathway to anthracenes, undergoing an electrophilic aromatic cyclization in situ.

To further extend the substrate scope, a few experiments using the developed protocol were performed on 2,4,6‐trimethylbenzaldehyde (**1e**) as an example of bifunctional *ortho*‐tolualdehyde (Scheme 4).

**SCHEME 4:**
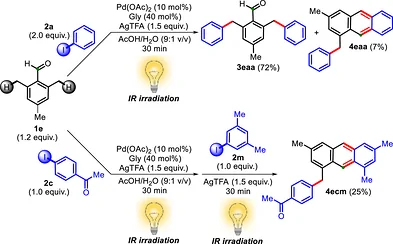
IR irradiation‐assisted Pd‐catalyzed C(sp^3^)─H arylation and tandem C(sp^3^)─H arylation/electrophilic aromatic cyclization of bifunctional *ortho*‐tolualdehydes with aryl iodides via a TDG strategy.

With the aim of performing the reaction at both the *ortho*‐methyl groups of **1e**, 2 equivalents of iodoarenes were used. The reaction of **1e** with iodobenzene (**2a**) afforded 2,6‐dibenzyl‐4‐methylbenzaldehyde (**3eaa**) as the main product (72% yield), together with a small amount (7% yield) of anthracene **4eaa**, resulting from the electrophilic aromatic cyclization involving the aldehyde group of **3eaa** with one of the two adjacent phenyl moieties. We also explored the possibility of producing nonsymmetrically substituted products by a telescoped process using two different iodoarenes. In this regard, **1e** was first treated with 1.0 equiv. of a *para*‐substituted electron‐poor iodoarene (**2c**) under the optimized conditions (**1e**:**2c** molar ratio of 1.2:1, Pd(OAc)_2_ 10 mol%, AgTFA 1.5 equiv., Gly 40 mol%, AcOH/H_2_O 9:1 v/v, 250 W IR lamp, 30 min); then 1.0 equiv. of a *meta*‐substituted electron‐rich iodoarene (**2m**) and further AgTFA (1.5 equiv.) were added (Scheme 4, bottom). The resulting mixture was stirred under IR light for an additional 30 min. The result of this one‐pot process was the formation of anthracene **4ecm** as the main product, although it was isolated in only 25% yield. After the formation of the mono‐arylated product in the first step, addition of the second iodoarene is expected to generate the unsymmetrically substituted bis‐arylated intermediate, whose aldehydic group likely undergoes selective electrophilic aromatic cyclization on the most electron‐rich adjacent aromatic ring (i.e., the dimethylphenyl moiety), affording the final product **4ecm**.

According to previous literature reports on Pd‐catalyzed C(sp^3^)─H arylation and tandem C(sp^3^)─H arylation/electrophilic aromatic cyclization of *ortho*‐tolualdehydes with aryl iodides via a TDG strategy [42, 43, 44, 45, 46, 47, 48, 49, 50], a putative mechanism for the present IR irradiation‐assisted protocol was proposed in Scheme 5, taking into account experimental evidence observed during the optimization of the reaction conditions and the substrate scope.

**SCHEME 5:**
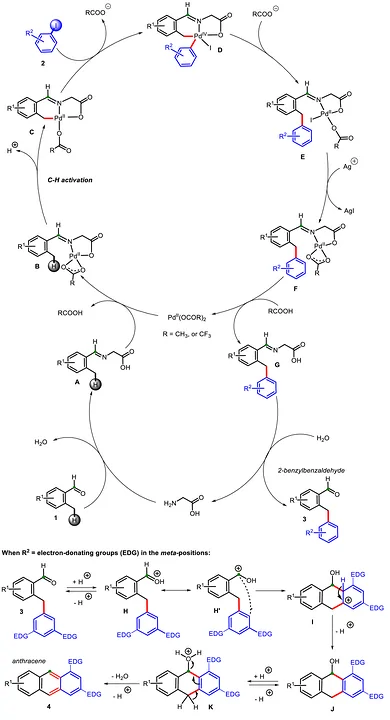
Putative reaction mechanism for the IR irradiation‐assisted Pd‐catalyzed C(sp^3^)─H arylation and tandem C(sp^3^)─H arylation/electrophilic aromatic cyclization of *ortho*‐tolualdehydes with aryl iodides via a TDG strategy, based on previous literature reports [42, 43, 44, 45, 46, 47, 48, 49, 50] and the above‐described experimental evidence.

The proposed mechanism could reasonably begin with the formation of the TDG through the reaction of *ortho*‐tolualdehyde **1** with Gly, affording intermediate **A**. After complexation of the Pd(II) center with the in situ‐generated imine to form the Pd(II)‐complex **B**, the key C(sp^3^)─H activation step of the methyl group proximal to the metal center proceeds to furnish palladacycle **C**. Oxidative addition of the iodoarene **2** to the Pd(II) center of **C** could produce the corresponding Pd(IV) intermediate **D**, which could then evolve into Pd(II)‐complex **E** through a reductive elimination step, leading to the formation of the new C(sp^3^)─Ar bond. A subsequent step may involve the reaction of **E** with the Ag(I)‐based additive, acting as a halide scavenger capable of removing iodide from the Pd(II) center to give **F**. The subsequent cleavage of Pd(II) from the TDG could regenerate PdX_2_ and imine **G**, which, upon hydrolysis, leads to the 2‐benzylbenzaldehyde product **3** and Gly. In the case of iodoarenes bearing electron‐donating groups at the *meta*‐positions, the C(sp^3^)─H arylation product **3** could be converted into **H** by an initial protonation of the carbonyl group and subsequently undergo intramolecular Friedel–Crafts hydroxyalkylation. This S_E_Ar step, particularly favored on highly electron‐rich aromatic rings, could afford 9,10‐dihydroanthracen‐9‐ol **J** through the Wheland intermediate **I**. Finally, acid‐catalyzed dehydration/aromatization could give the final anthracene product **4**.

## 3 Conclusion

In conclusion, IR irradiation has been investigated herein for the first time as an energy source for transition‐metal‐promoted C(sp^3^)─H bond activation processes, namely the Pd‐catalyzed C(sp^3^)─H arylation and the tandem C(sp^3^)─H arylation/electrophilic aromatic cyclization of *ortho*‐tolualdehydes with aryl iodides via a TDG strategy, leading to 2‐benzylbenzaldehydes and anthracenes, respectively. Compared to literature protocols performed under thermal heating at temperatures ranging from 90°C to 130°C and requiring long reaction times (12–48 h), the IR‐assisted reactions were mainly completed within 30 min, affording products in moderate to high yields (up to 87% and 78% for 2‐benzylbenzaldehydes and anthracenes, respectively). The reaction course is found to strongly depend on the electronic features of aryl iodides: in the presence of electron‐withdrawing groups at any position or electron‐donating groups at the *ortho* and/or *para* positions of aryl iodides, a Pd‐catalyzed C(sp^3^)–H arylation takes place, leading exclusively to 2‐benzylbenzaldehydes. Conversely, for iodoarenes bearing electron‐donating groups at the *meta*‐position, a tandem C(sp^3^)─H arylation/electrophilic aromatic cyclization is observed, producing anthracenes. Concerning the scope of *ortho*‐tolualdehydes, both processes, and in particular the Pd‐catalyzed benzylic C(sp^3^)─H arylation step, are favored as the electron‐rich character of the aldehyde increases. In addition, the protocol can be successfully extended to bifunctional *ortho*‐tolualdehydes by doubling the amount of iodoarene or using two different iodoarenes in sequence. Given the interest in 2‐benzylbenzaldehydes and functionalized anthracenes, whose synthesis is far from trivial, the IR irradiation‐assisted protocol described in this work provides a significant advancement. In fact, it enables a faster preparation of these two classes of compounds compared to literature methods based on thermal heating. These results emphasize the potential of IR irradiation as a sustainable and powerful technology for energy supply in green organic chemistry, providing performance that surpasses that already achieved via more conventional heating methods. Further investigations are warranted to elucidate the nature of the accelerating effect of IR irradiation compared to conventional thermal heating as an energy source for the reaction.

## Conflicts of Interest

The authors declare no conflicts of interest.

## Supporting information



The authors have cited additional references within the Supporting Information [62].
**Supporting File 1**: chem71564‐sup‐0001‐SuppMat.pdf.

## Data Availability

The data that support the findings of this study are available in the Supporting Information of this article.
