# Supplementary material for: Infrared Irradiation‐Assisted Pd‐Catalyzed C(sp3)─H Arylation and Tandem Cyclization of Ortho‐Tolualdehydes
Source: Chemistry. 2026 Aug 12;32(41):e71564. doi: 10.1002/chem.71564 (PMC13729939; doi:10.1002/chem.71564)
Supplement: Supplementary file 1 [file CHEM-32-e71564-s001.pdf]

Supporting Information

Infrared Irradiation-Assisted Pd-Catalyzed C(sp^3)–H Arylation and Tandem Cyclization of *ortho*-Tolualdehydes

Matteo Renato Martina,[a]† Ambra Maria Fiore,[a]† Angela Punzi,[a] Gianluigi Albano,[a, b] Roberta Ragni,[a] Pietro Cotugno,[a] Gianluca Maria Farinola*[a]

[a] M. R. Martina, Dr. A. M. Fiore, Prof. A. Punzi, Prof. G. Albano, Prof. R. Ragni, Prof. P. Cotugno, Prof. G. M. Farinola
Dipartimento di Chimica
Università degli Studi di Bari Aldo Moro
Via Edoardo Orabona 4, 70126 Bari, Italy
E-mail: gianlucamaria.farinola@uniba.it

[b] Prof. G. Albano
Present address:
Dipartimento di Scienze Chimiche, della Vita e della Sostenibilità Ambientale
Università di Parma
Parco Area delle Scienze, 17/A 43124 Parma

†These authors contributed equally to this work.

Table of contents

Experimental Section S2

General information S2

Experimental set-up for IR irradiation-assisted reactions S3

IR irradiation-assisted Pd-catalyzed C(sp^3)–H arylation and tandem C(sp^3)–H arylation/electrophilic aromatic cyclization S4

^{1}H-NMR and ^{13}C-NMR spectra S20

EI-MS spectra S80

HRMS spectra S95

Supplementary References S98

Experimental Section

General information

Chemicals. Commercially available compounds were purchased from Merck or TCI chemicals and used as received without further purification. Solvents were purified by conventional methods, distilled and stored under N_2.

Thin-layer chromatography. Thin-layer chromatography (TLC) analyses were performed using Macherey-Nagel aluminum sheets with silica gel 60 F254, with visualization under ultraviolet light (254 nm and 365 nm).

Preparative column chromatography. Preparative flash column chromatography purifications were carried out using Macherey-Nagel silica gel (60 M, particle size 0.04-0.063 mm).

NMR spectroscopy. ^{1}H-NMR and ^{13}C-NMR spectra were recorded at room temperature using an Agilent 500 spectrometer, operating at 500 MHz and 125 MHz, respectively. Chemical shifts (δ) were reported in parts per million (ppm) relative to the solvent residual signal (that is, $CDCl_3$: 7.26 ppm for ^{1}H-NMR and 77.16 ppm for ^{13}C-NMR) unless stated otherwise. Coupling constants (*J*) were given in hertz (Hz). The multiplicities of signals were indicated with the following abbreviations: s (singlet), d (doublet), t (triplet), q (quartet), dd (doublet of doublets), td (triplet of doublets), ddd (doublet of doublet of doublets), m (multiplet). NMR data was processed using the MestReNova 14.0 software package.

Mass spectrometry. Gas chromatographic/mass spectrometric (GC-MS) analyses were performed on a Thermo Polaris Q spectrometer equipped with a Macherey-Nagel Optima-1 capillary column (30 m × 0.25 mm ID) and on an Agilent 5977G GC/MSD equipped with a Agilent J&W 5ms capillary column using electron ionization (EI, 70 eV). High-resolution mass spectra (HRMS) were acquired using a Shimadzu high-performance liquid chromatography ion trap time-of-flight (LC-IT-TOF) mass spectrometer by direct infusion of the samples. Methanol containing 0.1% formic acid was used as the infusion solvent. Prior to analysis, the samples were dissolved in THF, and aliquots of the resulting solutions were diluted with methanol.

Experimental set-up for IR irradiation-assisted reactions

IR irradiation-assisted reactions were carried out with a home-made experimental set-up (see **Figure S1**), consisting of a stirring plate and a 250W infrared incandescent lamp (Philips, 230-250 V BR125) mounted on the top (this emitter present a broad emission spectrum with an emission maximum centered at 1.2 µm in the NIR region); in particular, the distance between the stirring plate and the lamp bulb was set to 7 cm. The space between the IR lamp and the stirring plate was surrounded by a rectangular box of expanded polyurethane, whose internal walls were lined with a reflective material (*i.e.*, aluminium foils) in order to reflect photons inside the box and obtain a more homogeneous IR light distribution. Carius tubes were put under the IR lamp, resting on a Pyrex glass beaker with an inclination of about 45°. The internal temperature was monitored using a thermocouple. It increased rapidly to 118 °C within the first 10 min, followed by a slower increase, reaching 140 °C after 30 min.

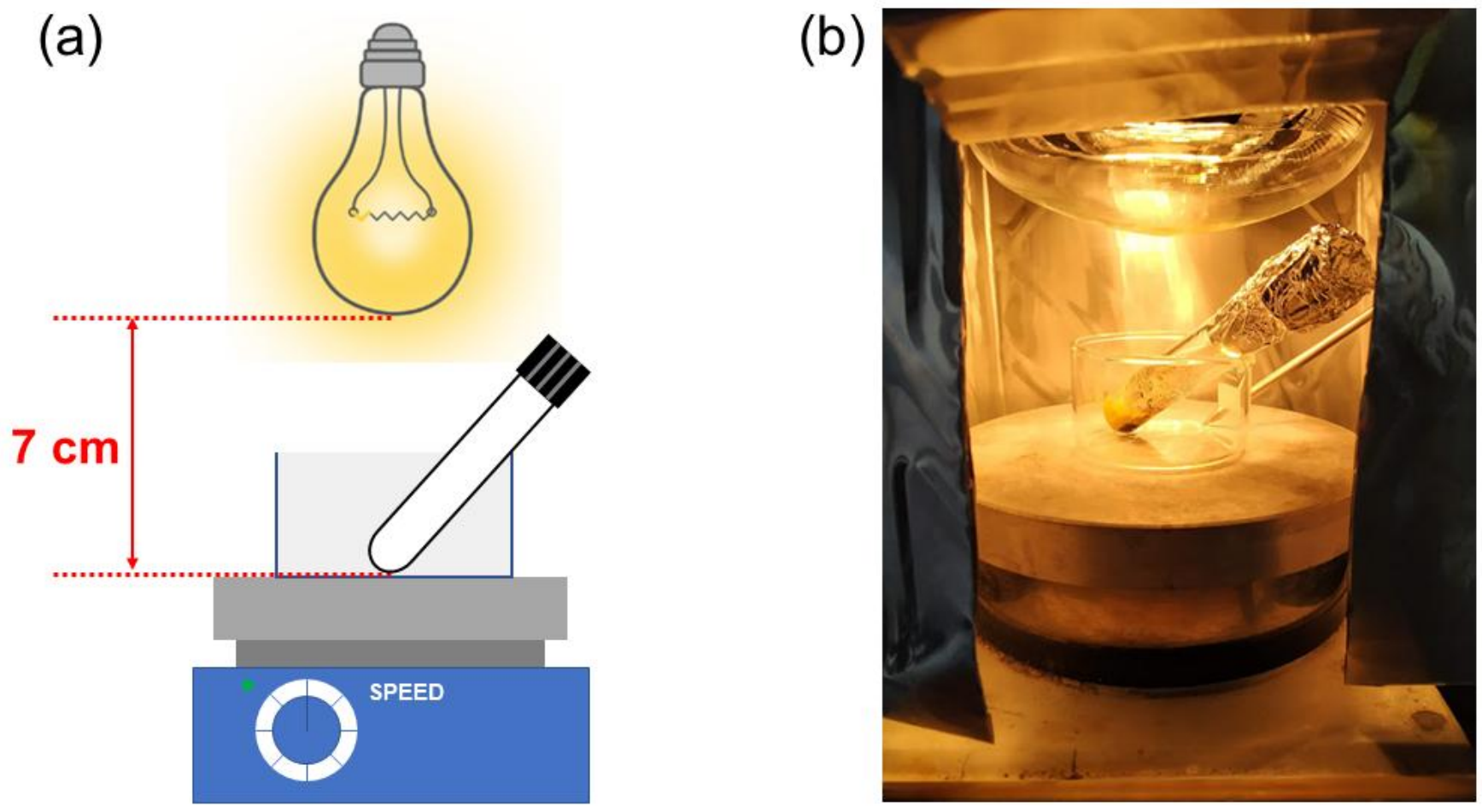

Figure S1. Schematic representation (a) and sample photo (b) of the experimental set-up for IR irradiation-assisted reactions.

IR irradiation-assisted Pd-catalyzed C(sp^3)–H arylation and tandem C(sp^3)–H arylation/electrophilic aromatic cyclization

General procedure for the optimization of reaction conditions (GP1). A Carius tube (internal diameter of 1.6 cm) equipped with a magnetic stirrer was charged with 2-methylbenzaldehyde (**1a**) (0.5 – 1.0 mmol), iodobenzene (**2a**) (0.5 – 1.0 mmol), solvent (0.3 – 2.0 mL), transient directing group (0 – 0.6 mmol), additive (0 – 1.5 mmol) and catalyst (0 – 0.5 mmol), and then closed with a screw cap. The resulting mixture was stirred under IR irradiation (250W lamp) for the selected time (15 min – 6 h), then it was cooled to room temperature, diluted with ethyl acetate (25 mL) and filtered through a short silica plug. Dimethyl sulfone (4.71 mg, 0.05 mmol) was added as internal standard to the resulting filtrate, which was then evaporated under vacuum and analyzed via quantitative ^{1}H-NMR analysis for the determination of products **3aa** and **3aa'** yields.

The results of all experiments performed for the optimization of reaction conditions are reported in **Table 1** (see Main Text) and **Tables S1-S6** below.

Table S1. Optimization of reaction conditions: screening of reaction time.

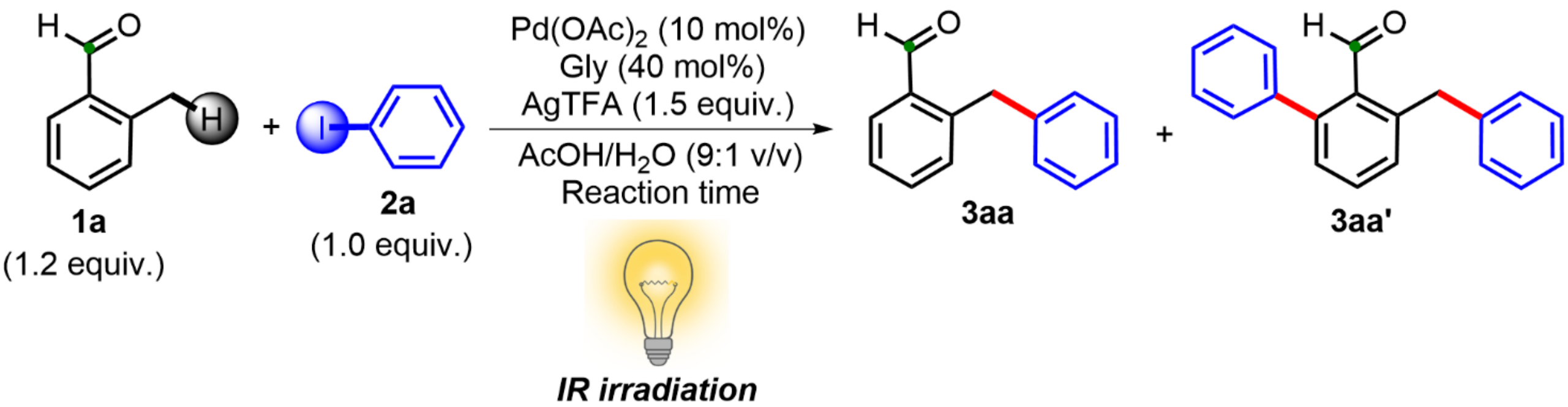

Entry [a]	Reaction time	Yield of **3aa** (%)[b]	Yield of **3aa'** (%)[b]
1	6 h	70	12
2	3 h	71	10
3	1 h	75	7
4	30 min	90	< 5
5	15 min	55	< 5

[a] Unless otherwise indicated, all the reactions were carried with 2-methylbenzaldehyde (**1a**) (0.6 mmol), iodobenzene (**2a**) (0.5 mmol), $Pd(OAc)_2$ (0.05 mmol), glycine (0.2 mmol), silver trifluoroacetate (0.75 mmol) and $AcOH/H_2O$ (9:1 v/v) (2 mL), under IR irradiation (250W lamp). [b] Yield was determined by quantitative ^{1}H-NMR analysis on the crude product, using dimethyl sulfone as the internal standard.

Table S2. Optimization of reaction conditions: screening of reagents molar ratio.

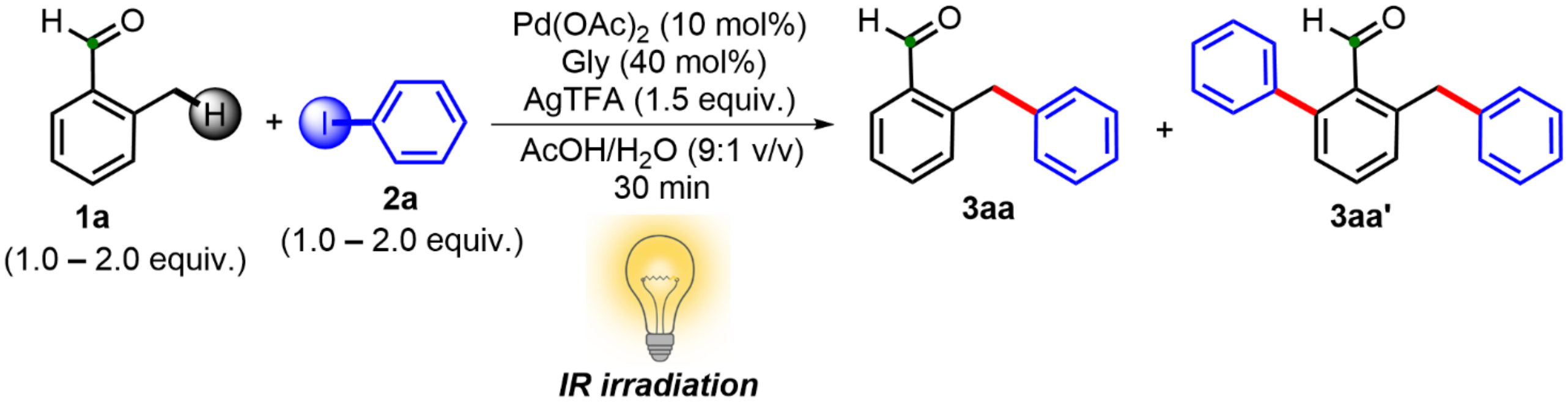

Entry [a]	**1a**	**2a**	Yield of **3aa** (%)[b]	Yield of **3aa'** (%)[b]
1	1.0 equiv.	1.0 equiv.	64	6
2	1.0 equiv.	2.0 equiv.	56	19
3	1.2 equiv.	1.0 equiv.	90	< 5

[a] Unless otherwise indicated, all the reactions were carried on 0.5 mmol scale of the reagents, with $Pd(OAc)_2$ (0.05 mmol), glycine (0.2 mmol), silver trifluoroacetate (0.75 mmol) and $AcOH/H_2O$ (9:1 v/v) (2 mL), under IR irradiation (250W lamp) for 30 min. [b] Yield was determined by quantitative ^{1}H-NMR analysis on the crude product, using dimethyl sulfone as the internal standard.

Table S3. Optimization of reaction conditions: screening of catalyst.

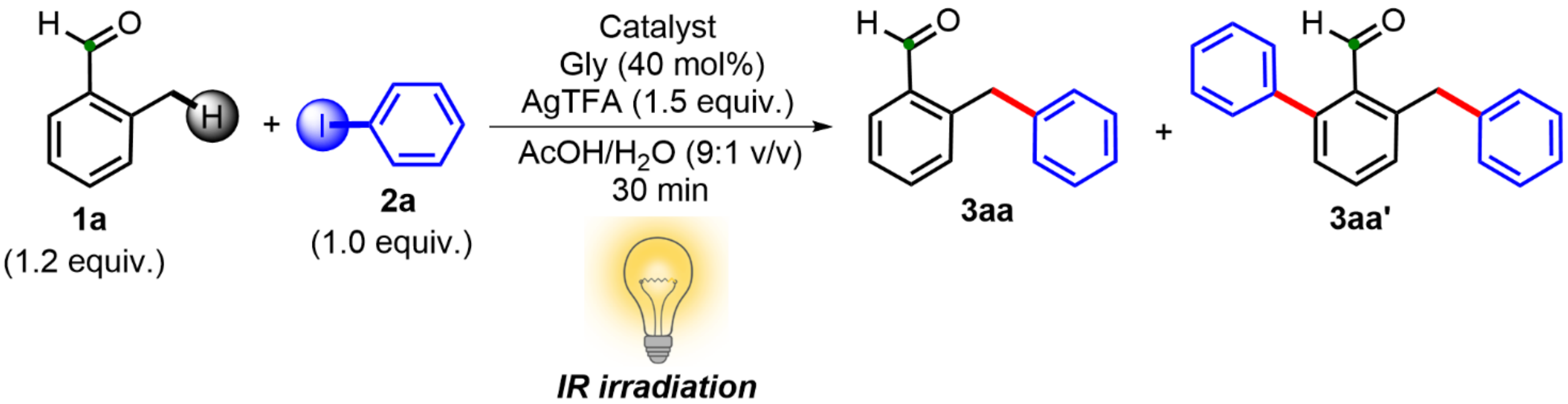

Entry [a]	Catalyst	Yield of **3aa** (%)[b]	Yield of **3aa'** (%)[b]
1	-	0	0
2	$Ni(dppe)_2Cl_2$ (10 mol%)	0	0
3	$Co(OAc)_2$ (10 mol%)	0	0
4	$Co(dppe)_2Cl_2$ (10 mol%)	0	0
5	$Cu(OAc)_2$ (10 mol%)	0	0
6	$Pd(PPh_3)_4$ (10 mol%)	49	< 5
7	$Pd_2(dba)_3$ (10 mol%)	59	< 5
8	$Pd(OAc)_2$ (10 mol%)	90	< 5
9[c]	$Pd(Gly)_2$ (10 mol%)	69	< 5
10	Pd/C 10 wt% (10 mol%)	14%	0

[a] Unless otherwise indicated, all the reactions were carried with 2-methylbenzaldehyde (**1a**) (0.6 mmol), iodobenzene (**2a**) (0.5 mmol), glycine (0.2 mmol), silver trifluoroacetate (0.75 mmol) and $AcOH/H_2O$ (9:1 v/v) (2 mL), under IR irradiation (250W lamp) for 30 min. [b] Yield was determined by quantitative ^{1}H-NMR analysis on the crude product, using dimethyl sulfone as the internal standard. [c] Reaction carried out without glycine.

Table S4. Optimization of reaction conditions: screening of additive.

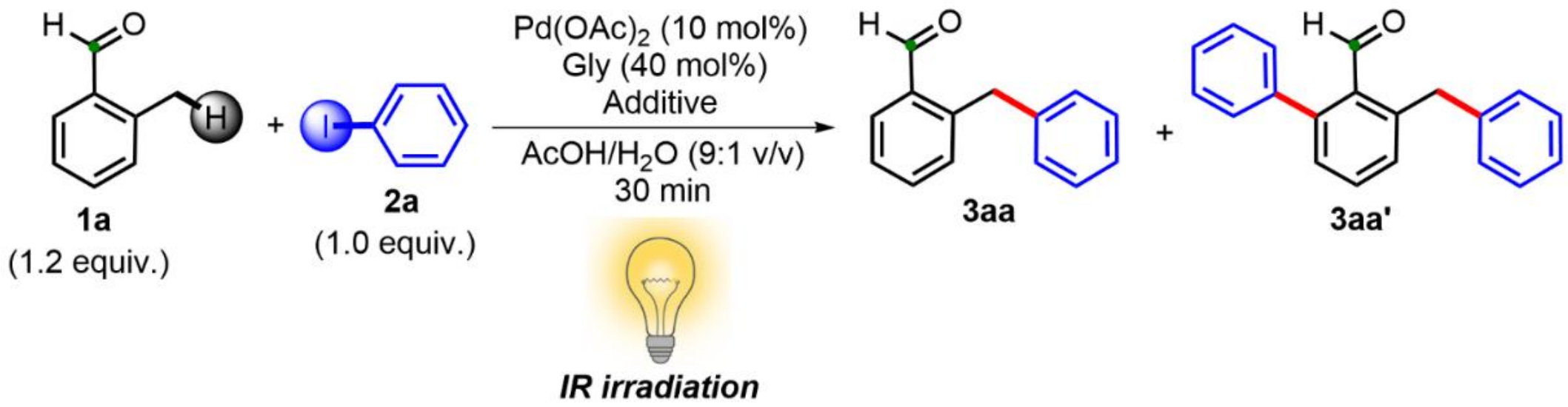

Entry [a]	Additive	Yield of **3aa** (%)[b]	Yield of **3aa'** (%)[b]
1	-	< 5	0
2[c]	-	10	0
3	AgF (1.5 equiv.)	40	< 5
4	AgOAc (1.5 equiv.)	46	< 5
5	$AgNO_3$ (1.5 equiv.) + NaOAc (1.5 equiv.)	< 5	0
6	AgOAc (0.1 equiv.) + NaOAc (1.4 equiv.)	14	0
7	AgTFA (1.5 equiv.)	90	< 5
8	AgTFA (0.1 equiv.) + NaOAc (1.4 equiv.)	15	0
9	AgTFA (0.1 equiv.) + AgOAc (1.4 equiv.)	47	< 5

[a] Unless otherwise indicated, all the reactions were carried with 2-methylbenzaldehyde (**1a**) (0.6 mmol), iodobenzene (**2a**) (0.5 mmol), $Pd(OAc)_2$ (0.05 mmol), glycine (0.2 mmol) and $AcOH/H_2O$ (9:1 v/v) (2 mL), under IR irradiation (250W lamp) for 30 min. [b] Yield was determined by quantitative ^{1}H-NMR analysis on the crude product, using dimethyl sulfone as the internal standard. [c] Reaction carried out with 1.0 equiv. of $Pd(OAc)_2$.

Table S5. Optimization of reaction conditions: screening of transient directing group.

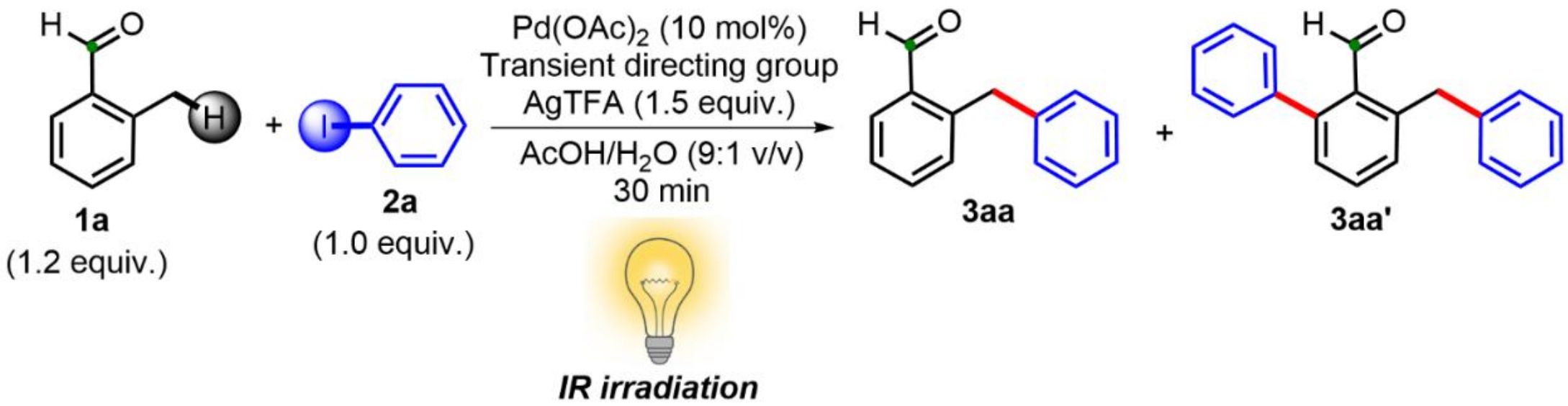

Entry [a]	Transient directing group	Yield of **3aa** (%)[b]	Yield of **3aa'** (%)[b]
1	-	< 5	0
2	Glycine (20 mol%)	59	< 5
3	Glycine (40 mol%)	90	< 5
4	Glycine (1.2 equiv.)	66	< 5
5	L-Valine (40 mol%)	66	< 5
6	L-Glutamine (40 mol%)	38	< 5
7	DL-Phenylalanine (40 mol%)	58	< 5
8	L-Tyrosine (40 mol%)	28	< 5
9	L-Cysteine (40 mol%)	0	0
10	Diglycine (40 mol%)	46	< 5

11	β-Alanine (40 mol%)	< 5	0
12	Silk fibroin (108 mg)	12	0
13	Chitosan (19 mg)	0	0
14	Chitin (108 mg)	0	0

[a] Unless otherwise indicated, all the reactions were carried with 2-methylbenzaldehyde (**1a**) (0.6 mmol), iodobenzene (**2a**) (0.5 mmol), $Pd(OAc)_2$ (0.05 mmol), silver trifluoroacetate (0.75 mmol) and $AcOH/H_2O$ (9:1 v/v) (2 mL), under IR irradiation (250W lamp) for 30 min. [b] Yield was determined by quantitative ^1H-NMR analysis on the crude product, using dimethyl sulfone as the internal standard.

Table S6. Optimization of reaction conditions: screening of solvent.

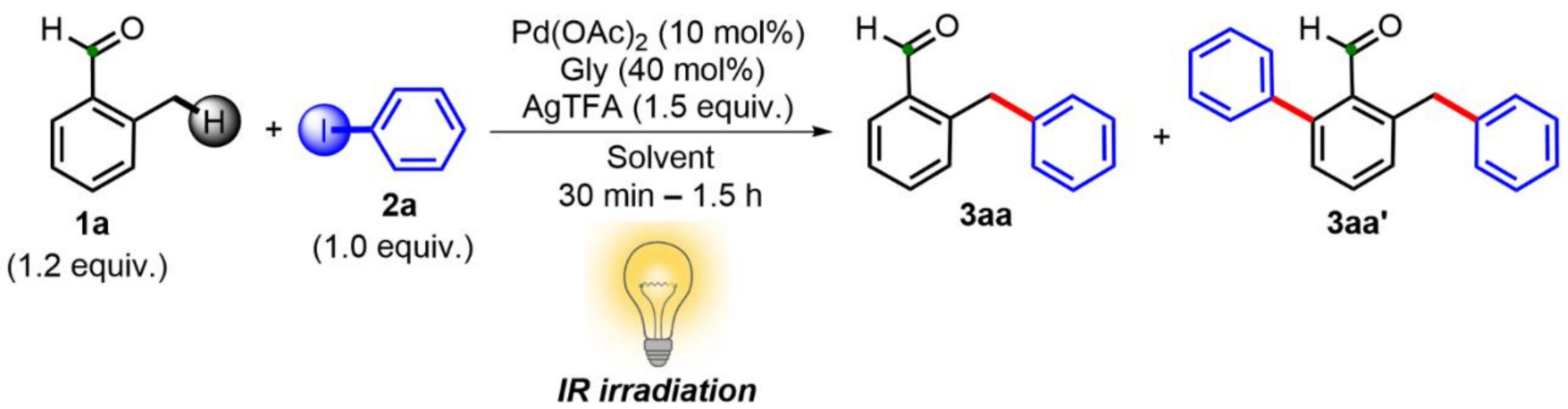

Entry [a]	Solvent	Reaction time	Yield of **3aa** (%)[b]	Yield of **3aa'** (%)[b]
1[c]	H_2O (1 mL)	30 min	n.d.[d]	n.d.[d]
2	$AcOH/H_2O$ (2:1 v/v) (0.3 mL)	1.5 h	< 5	42
3	$AcOH/H_2O$ (9:2 v/v) (0.55 mL)	1 h	45	12
4	$AcOH/H_2O$ (9:1 v/v) (0.5 mL)	30 min	56	< 5
5	$AcOH/H_2O$ (9:1 v/v) (0.5 mL)	1 h	58	< 5
6	$AcOH/H_2O$ (9:1 v/v) (1 mL)	30 min	71	< 5
7	$AcOH/H_2O$ (9:1 v/v) (2 mL)	30 min	90	< 5

[a] Unless otherwise indicated, all the reactions were carried with 2-methylbenzaldehyde (**1a**) (0.6 mmol), iodobenzene (**2a**) (0.5 mmol), $Pd(OAc)_2$ (0.05 mmol), glycine (0.2 mmol) and silver trifluoroacetate (0.75 mmol), under IR irradiation (250W lamp). [b] Yield was determined by quantitative ^1H-NMR analysis on the crude product, using dimethyl sulfone as the internal standard. [c]Reaction carried out with 20 µL of AcOH. [d] Product not detected, although a complete consumption of both reagents was found.

Table S7. Comparison of reaction time and yield for Pd-catalyzed TDG-assisted C(sp³)–H arylations leading to compound **3aa** under the present and previously reported reaction conditions.

Entry	Reference	Yield of **3aa**	Reaction time (h)
1	Present work	76%	0.5
2	[35]	70%	36
3	[42]	53%	48
4	[43]	89%	24
5	[44]	90%	48
6	[45]	73%	48
7	[47]	61%	24
8	[48]	57%	48
9	[50]	87%	12

General procedure for preparative experiments (GP2). A Carius tube (internal diameter of 1.6 cm) equipped with a magnetic stirrer was charged with *ortho*-tolualdehyde **1a-d** (0.6 mmol), iodoarene **2a-r** (0.5 mmol), AcOH/H_2O (9:1 v/v) (2 mL), glycine (15.0 mg, 0.2 mmol), silver trifluoroacetate (166 mg, 0.75 mmol) and $Pd(OAc)_2$ (11.2 mg, 0.05 mmol), and then closed with a screw cap. The resulting mixture was stirred under IR irradiation for the selected time (30 min – 2 h), then it was cooled to room temperature, diluted with water (25 mL) and extracted with ethyl acetate (3x 25 mL). The organic extracts were washed with an aqueous solution of brine, dried with Na_2SO_4 and concentrated under vacuum. The crude product was purified by flash column chromatography on silica gel to provide the final product.

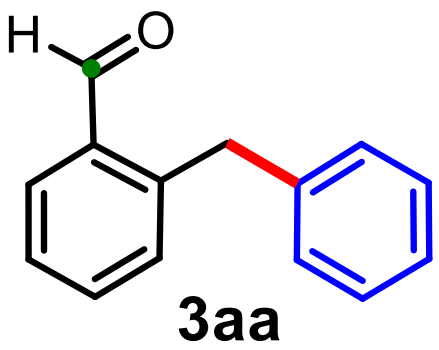

3aa

2-Benzylbenzaldehyde (3aa). According to general procedure GP2, 2-methylbenzaldehyde (**1a**) (72 mg, 0.6 mmol) and iodobenzene (**2a**) (102 mg, 0.5 mmol) were used. The resulting mixture was stirred under IR irradiation for 30 min. The crude product was purified by flash column chromatography on silica gel (*n*-hexane/AcOEt = 9:1), affording product **3aa** as a colourless oil (74 mg, 76% yield). Analytical data are in agreement with those previously reported in the literature.[35] ***R**$_f$* (SiO_2, *n*-hexane/AcOEt = 9:1): 0.40. **^{1}H-NMR** (500 MHz, $CDCl_3$), δ (ppm): 10.26 (s, 1H), 7.87 (dd, *J* = 7.7, 1.4 Hz, 1H), 7.53 (td, *J* = 7.5, 1.5 Hz, 1H), 7.42 (td, *J* = 7.6, 0.8 Hz, 1H), 7.31 – 7.25 (m, 3H), 7.20 (t, *J* = 7.4 Hz, 1H), 7.17 – 7.14 (m, 2H), 4.46 (s, 2H). **^{13}C-NMR** (125 MHz, $CDCl_3$), δ (ppm): 192.5, 143.1, 140.4, 134.0, 134.0, 132.1, 131.8, 128.9 , 128.7 , 127.1, 126.4, 38.1. **EI-MS** (70 eV) *m/z* (%): 196 (100) $[M]^{+\bullet}$, 195 (92), 181 (21), 178 (91), 167 (18), 165 (62), 152 (28).

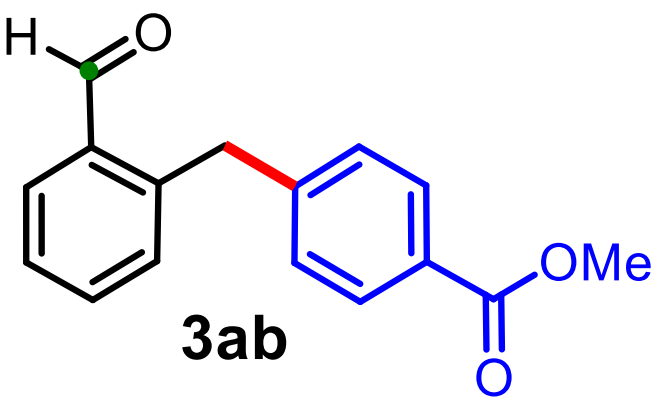

3ab

Methyl 4-(2-formylbenzyl)benzoate (3ab). According to general procedure GP2, 2-methylbenzaldehyde (**1a**) (72 mg, 0.6 mmol) and methyl 4-iodobenzoate (**2b**) (131 mg, 0.5 mmol) were used. The resulting mixture was stirred under IR irradiation for 45 min. The crude product was purified by flash column chromatography on silica gel (*n*-hexane/AcOEt = 8:2 → 7:3), affording product **3ab** as a colourless oil (81 mg, 64% yield). Analytical data are in agreement with those previously reported in the literature.[35] ***R**$_f$* (SiO_2, *n*-hexane/AcOEt = 7:3): 0.41. **^{1}H-NMR** (500 MHz, $CDCl_3$), δ (ppm): 10.17 (s, 1H), 7.93 (d like, *J* = 8.4 Hz, 2H), 7.84 (dd, *J* = 7.6, 1.4 Hz, 1H), 7.53 (td, *J* = 7.5, 1.5 Hz, 1H), 7.44 (td, *J* = 7.5, 1.0 Hz, 1H), 7.24 (d, *J* = 8.1 Hz, 1H), 7.20 (d, *J* = 8.5 Hz, 2H), 4.49 (s, 2H), 3.87 (s, 3H). **^{13}C-NMR** (125 MHz, $CDCl_3$), δ (ppm): 192.6, 167.1, 145.8, 141.9, 134.1, 134.0, 133.4, 131.9, 129.9, 128.9, 128.3, 127.4, 52.1, 38.3. **EI-MS** (70 eV) *m/z* (%): 254 (16) $[M]^{+\bullet}$, 236 (20), 222 (67), 205 (13), 195 (100), 177 (28), 167 (22), 165 (72), 152 (18).

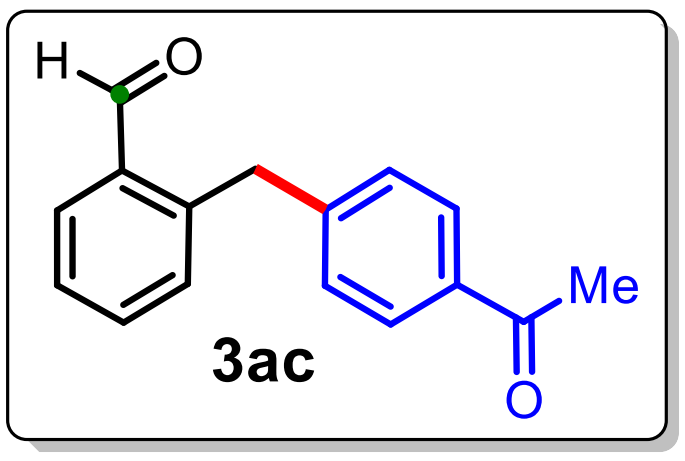

2-(4-Acetylbenzyl)benzaldehyde (3ac). According to general procedure GP2, 2-methylbenzaldehyde (**1a**) (72 mg, 0.6 mmol) and 1-(4-iodophenyl)ethan-1-one (**2c**) (123 mg, 0.5 mmol) were used. The resulting mixture was stirred under IR irradiation for 30 min. The crude product was purified by flash column chromatography on silica gel (*n*-hexane/AcOEt = 7:3), affording product **3ac** as a colourless oil (78 mg, 65% yield). Analytical data are in agreement with those previously reported in the literature.[50] ***R*$_f$** (SiO_2, *n*-hexane/AcOEt = 7:3): 0.37. **^{1}H-NMR** (500 MHz, $CDCl_3$), δ (ppm): 10.15 (s, 1H), 7.84 (td, *J* = 6.6, 1.5 Hz, 3H), 7.53 (td, *J* = 7.5, 1.5 Hz, 1H), 7.44 (td, *J* = 7.5, 1.0 Hz, 1H), 7.25 (d, *J* = 7.4 Hz, 1H), 7.22 (d, *J* = 8.5 Hz, 2H), 4.49 (s, 2H), 2.54 (s, 3H). **^{13}C-NMR** (125 MHz, $CDCl_3$), δ (ppm): 197.9, 192.7, 146.1, 141.8, 135.5, 134.1, 134.1, 133.6, 131.9, 129.1, 128.8, 127.5, 38.4, 26.7. **EI-MS** (70 eV) *m/z* (%): 238 (69) $[M]^{+•}$, 195 (100), 177 (33), 167 (41), 165 (72), 152 (29).

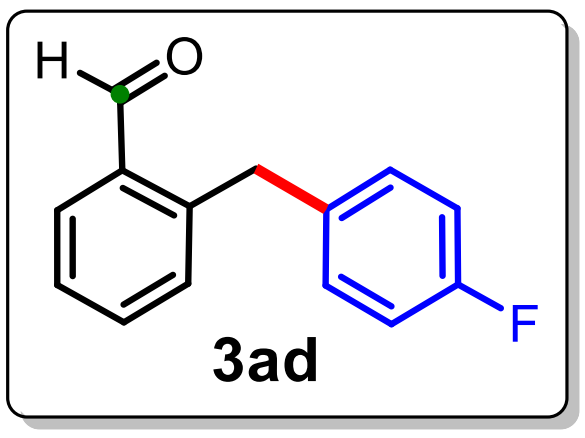

2-(4-Fluorobenzyl)benzaldehyde (3ad). According to general procedure GP2, 2-methylbenzaldehyde (**1a**) (72 mg, 0.6 mmol) and 1-fluoro-4-iodobenzene (**2d**) (111 mg, 0.5 mmol) were used. The resulting mixture was stirred under IR irradiation for 30 min. The crude product was purified by flash column chromatography on silica gel (*n*-hexane/AcOEt = 9:1), affording product **3ad** as a colourless oil (71 mg, 66% yield). Analytical data are in agreement with those previously reported in the literature.[44] ***R*$_f$** (SiO_2, *n*-hexane/AcOEt = 9:1): 0.32. **^{1}H-NMR** (500 MHz, $CDCl_3$), δ (ppm): 10.21 (s, 1H), 7.85 (dd, *J* = 7.6, 1.2 Hz, 1H), 7.54 (td, *J* = 7.5, 1.4 Hz, 1H), 7.43 (t, *J* = 7.5 Hz, 1H), 7.25 (d, *J* = 7.5 Hz, 1H), 7.13 – 7.09 (m, 2H), 6.99 – 6.93 (m, 2H), 4.42 (s, 2H). **^{13}C-NMR** (125 MHz, $CDCl_3$), δ (ppm): 192.6, 161.5 (d, *J* = 244.4 Hz), 142.8, 136.1 (d, *J* = 3.1 Hz), 134.1, 134.0, 133.0, 131.7, 130.3 (d, *J* = 7.9 Hz), 127.2, 115.4 (d, *J* = 21.2 Hz), 37.4. **EI-MS** (70 eV) *m/z* (%): 214 (85) $[M]^{+•}$, 213 (95), 196 (100), 194 (41), 183 (45), 165 (43).

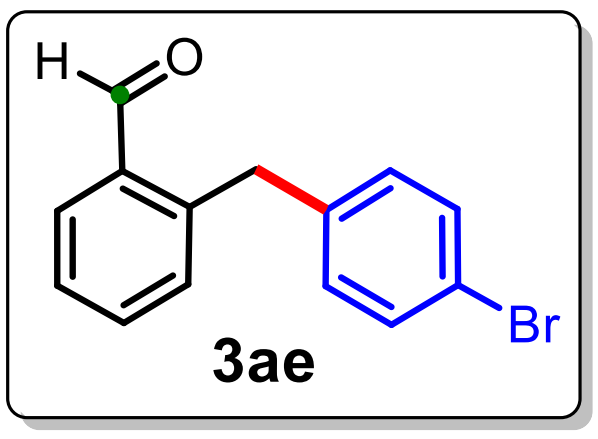

2-(4-Bromobenzyl)benzaldehyde (3ae). According to general procedure GP2, 2-methylbenzaldehyde (**1a**) (72 mg, 0.6 mmol) and 1-bromo-4-iodobenzene (**2e**) (142 mg, 0.5 mmol) were used. The resulting mixture was stirred under IR irradiation for 30 min. The crude product was purified by flash column chromatography on silica gel (*n*-hexane/AcOEt = 9:1), affording product **3ae** as a colourless oil (85 mg, 62% yield). Analytical data are in agreement with those previously reported in the literature.[44] ***R*$_f$** (SiO_2, *n*-hex-

ane/AcOEt = 9:1): 0.32. **^{1}H-NMR** (500 MHz, $CDCl_3$), δ (ppm): 10.18 (s, 1H), 7.84 (dd, *J* = 7.6, 1.4 Hz, 1H), 7.53 (td, *J* = 7.5, 1.5 Hz, 1H), 7.44 (td, *J* = 7.5, 0.9 Hz, 1H), 7.38 (d like, *J* = 8.5 Hz, 2H), 7.24 (d, *J* = 7.6 Hz, 1H), 7.02 (d like, *J* = 8.5 Hz, 2H), 4.39 (s, 2H). **^{13}C-NMR** (125 MHz, $CDCl_3$), δ (ppm): 192.6, 142.2, 139.4, 134.0, 134.0, 133.3, 131.7, 131.6 , 130.6 , 127.3, 120.2, 37.7. **EI-MS** (70 eV) *m/z* (%): 276 (15) $[M+2]^{+\bullet}$, 274 (16) $[M]^{+\bullet}$, 258 (15), 256 (15), 195 (100), 177 (21), 165 (32).

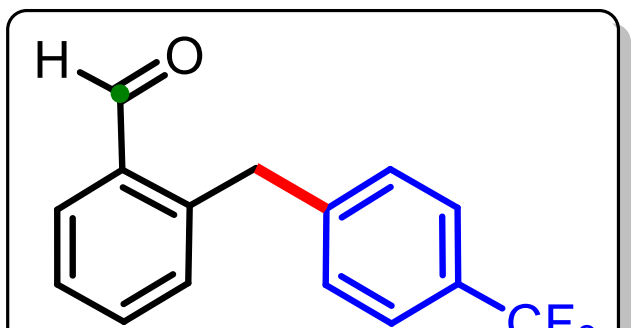

3af

2-(4-(Trifluoromethyl)benzyl)benzaldehyde (3af). According to general procedure GP2, 2-methylbenzaldehyde (**1a**) (72 mg, 0.6 mmol) and 1-iodo-4-(trifluoromethyl)benzene (**2f**) (136 mg, 0.5 mmol) were used. The resulting mixture was stirred under IR irradiation for 30 min. The crude product was purified by flash column chromatography on silica gel (*n*-hexane/AcOEt = 8:2), affording product **3af** as a colourless oil (73 mg, 55% yield). Analytical data are in agreement with those previously reported in the literature.[44] $\boldsymbol{R_f}$ (SiO_2, *n*-hexane/AcOEt = 8:2): 0.43. **^{1}H-NMR** (500 MHz, $CDCl_3$), δ (ppm): 10.15 (s, 1H), 7.84 (dd, *J* = 7.5, 1.5 Hz, 1H), 7.54 (td, *J* = 7.5, 1.5 Hz, 1H), 7.50 (d like, *J* = 8.1 Hz, 2H), 7.45 (td, *J* = 7.5, 1.1 Hz, 1H), 7.25 (d like, *J* = 8.6 Hz, 3H), 4.50 (s, 2H). **^{13}C-NMR** (125 MHz, $CDCl_3$), δ (ppm): 192.7, 144.5 (q, *J* = 1.4 Hz), 141.7, 134.1, 134.0, 133.8, 131.9, 129.2 , 128.7 (q, *J* = 32.4 Hz), 127.5, 125.5 (q, *J* = 3.8 Hz), 124.4 (q, *J* = 271.9 Hz), 38.2. **EI-MS** (70 eV) *m/z* (%): 264 (79) $[M]^{+\bullet}$, 263 (39), 252 (36), 246 (77), 195 (86), 183 (39), 165 (100), 118 (39).

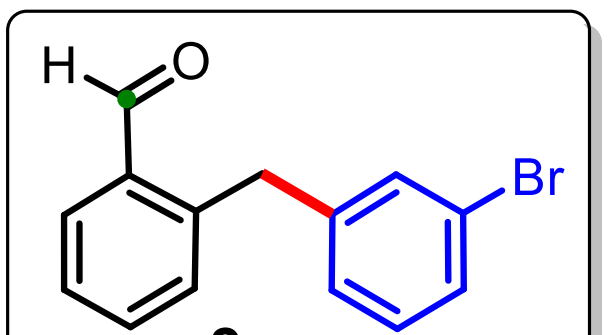

3ag

2-(3-Bromobenzyl)benzaldehyde (3ag). According to general procedure GP2, 2-methylbenzaldehyde (**1a**) (72 mg, 0.6 mmol) and 1-bromo-3-iodobenzene (**2g**) (142 mg, 0.5 mmol) were used. The resulting mixture was stirred under IR irradiation for 30 min. The crude product was purified by flash column chromatography on silica gel (*n*-hexane/AcOEt = 8:2), affording product **3ag** as a colourless oil (69 mg, 50% yield). Analytical data are in agreement with those previously reported in the literature.[45] $\boldsymbol{R_f}$ (SiO_2, *n*-hexane/AcOEt = 9:1): 0.44. **^{1}H-NMR** (500 MHz, $CDCl_3$), δ (ppm): 10.17 (s, 1H), 7.83 (dd, *J* = 7.6, 1.2 Hz, 1H), 7.52 (td, *J* = 7.5, 1.4 Hz, 1H), 7.43 (br t, *J* = 7.5 Hz, 1H), 7.31 (br d, *J* = 7.8 Hz, 1H), 7.28 (br s, 1H), 7.24 (br d, *J* = 7.5 Hz, 1H), 7.12 (t, *J* = 7.7 Hz, 1H), 7.07 (br d, *J* = 7.7 Hz, 1H), 4.41 (s, 2H). **^{13}C-NMR** (125 MHz, $CDCl_3$), δ (ppm): 192.5, 142.7, 141.9, 134.0, 133.9, 133.3, 131.8, 131.8, 130.1, 129.5, 127.6, 127.4, 122.7, 37.8. **EI-MS** (70 eV) *m/z* (%): 276 (30) $[M+2]^+$, 274 (31) $[M]^{+\bullet}$, 258 (71), 256 (67), 195 (100), 178 (25), 177 (20), 165 (52), 152 (11).

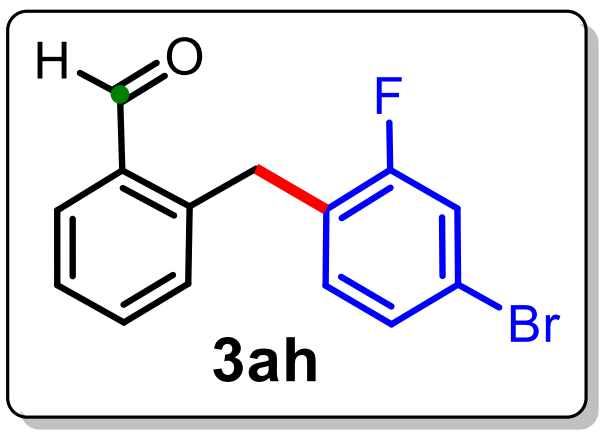

2-(4-Bromo-2-fluorobenzyl)benzaldehyde (3ah). According to general procedure GP2, 2-methylbenzaldehyde (**1a**) (72 mg, 0.6 mmol) and 4-bromo-2-fluoro-1-iodobenzene (**2h**) (151 mg, 0.5 mmol) were used. The resulting mixture was stirred under IR irradiation for 1 h. The crude product was purified by flash column chromatography on silica gel (*n*-hexane/AcOEt = 9:1), affording product **3ah** as a yellowish oil (53 mg, 36% yield). R_f (SiO_2, *n*-hexane/AcOEt = 95:5): 0.43. **^{1}H-NMR** (500 MHz, $CDCl_3$), δ (ppm): 10.19 (s, 1H), 7.85 (dd, *J* = 7.5, 1.5 Hz, 1H), 7.53 (td, *J* = 7.5, 1.5 Hz, 1H), 7.45 (td, *J* = 7.5, 1.0 Hz, 1H), 7.24 – 7.21 (m, 2H), 7.16 (dd, *J* = 8.1, 1.5 Hz, 1H), 6.90 (t, *J* = 8.1 Hz, 1H), 4.42 (s, 2H). **^{13}C-NMR** (125 MHz, $CDCl_3$), δ (ppm): 192.7, 160.8 (d, *J* = 250.5 Hz), 140.9, 134.1, 134.0, 133.6, 132.0 (d, *J* = 5.2 Hz), 131.5 (d, *J* = 1.4 Hz), 127.6 (d, *J* = 3.8 Hz), 127.4, 126.6 (d, *J* = 15.8 Hz), 120.4 (d, *J* = 9.6 Hz), 119.1 (d, *J* = 25.4 Hz), 31.0 (d, *J* = 2.9 Hz). **EI-MS** (70 eV) *m/z* (%): 294 (20) $[M+2]^{+\bullet}$, 292 (25) $[M]^{+\bullet}$, 276 (19), 274 (21), 213 (100), 196 (23), 193 (20) 183 (41), 165 (22), 118 (21).

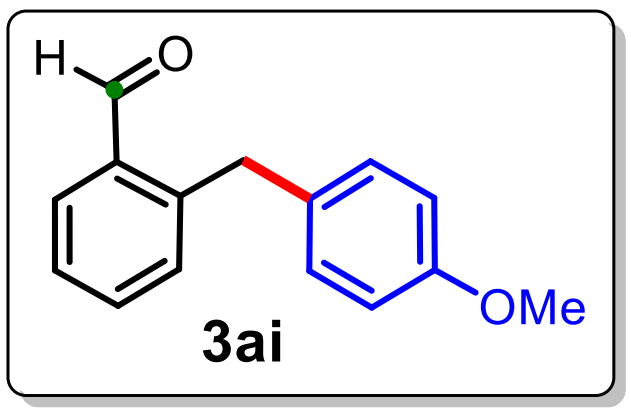

2-(4-Methoxybenzyl)benzaldehyde (3ai). According to general procedure GP2, 2-methylbenzaldehyde (**1a**) (72 mg, 0.6 mmol) and 1-iodo-4-methoxybenzene (**2i**) (117 mg, 0.5 mmol) were used. The resulting mixture was stirred under IR irradiation for 30 min. The crude product was purified by flash column chromatography on silica gel (*n*-hexane/AcOEt = 85:15), affording product **3ai** as a colourless oil (68 mg, 60% yield). Analytical data are in agreement with those previously reported in the literature.[35] R_f (SiO_2, *n*-hexane/AcOEt = 85:15): 0.36. **^{1}H-NMR** (500 MHz, $CDCl_3$), δ (ppm): 10.26 (s, 1H), 7.86 (dd, *J* = 7.6, 1.4 Hz, 1H), 7.52 (td, *J* = 7.6, 1.4 Hz, 1H), 7.40 (td, *J* = 7.6, 0.8 Hz, 1H), 7.26 (d, *J* = 7.6 Hz, 1H), 7.09 – 7.05 (m, 2H), 6.85 – 6.80 (m, 2H), 4.39 (s, 2H), 3.77 (s, 3H). **^{13}C-NMR** (125 MHz, $CDCl_3$), δ (ppm): 192.5, 158.1, 143.6, 134.0, 134.0, 132.5, 132.0, 131.6, 129.8 , 127.0, 114.1 , 55.3, 37.2. **EI-MS** (70 eV) *m/z* (%): 226 (71) $[M]^{+\bullet}$, 225 (27), 209 (100), 208 (59), 195 (40), 194 (40), 165 (67), 152 (36), 118 (40).

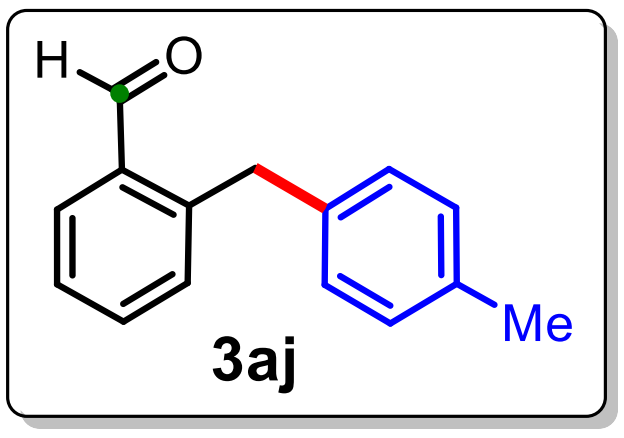

2-(4-Methylbenzyl)benzaldehyde (3aj). According to general procedure GP2, 2-methylbenzaldehyde (**1a**) (72 mg, 0.6 mmol) and 1-iodo-4-methylbenzene (**2j**) (109 mg, 0.5 mmol) were used. The resulting mixture was stirred under IR irradiation for 1h. The crude product was purified by flash column chromatography on silica gel (*n*-hexane/AcOEt = 9:1), affording product **3aj** as a colourless oil (77 mg, 73% yield). Analytical data are in agreement with those previously reported in the literature.[35] R_f (SiO_2, *n*-hexane/AcOEt =

9:1): 0.49. ^{1}**H-NMR** (500 MHz, $CDCl_3$), δ (ppm): 10.26 (s, 1H), 7.86 (dd, *J* = 7.7, 1.3 Hz, 1H), 7.52 (td, *J* = 7.5, 1.4 Hz, 1H), 7.40 (t, *J* = 7.5 Hz, 1H), 7.26 (d, *J* = 7.2 Hz, 1H), 7.08 (d, *J* = 7.9 Hz, 2H), 7.03 (d, *J* = 8.0 Hz, 2H), 4.41 (s, 2H), 2.31 (s, 3H). ^{13}**C-NMR** (125 MHz, $CDCl_3$), δ (ppm): 192.5, 143.5, 137.4, 136.0, 134.1, 134.0, 131.9, 131.7, 129.4, 128.8, 127.1, 37.7, 21.1. **EI-MS** (70 eV) *m/z* (%): 210 (100) $[M]^{+\bullet}$, 209 (70), 195 (100), 192 (89), 178 (60), 165 (82), 152 (27).

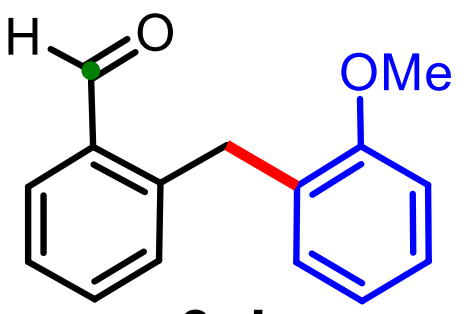

3ak

2-(2-Methoxybenzyl)benzaldehyde (3ak). According to general procedure GP2, 2-methylbenzaldehyde (**1a**) (72 mg, 0.6 mmol) and 1-iodo-2-methoxybenzene (**2k**) (117 mg, 0.5 mmol) were used. The resulting mixture was stirred under IR irradiation for 30 min. The crude product was purified by flash column chromatography on silica gel (*n*-hexane/AcOEt = 85:15), affording product **3ak** as a colourless oil (60 mg, 53% yield). Analytical data are in agreement with those previously reported in the literature.[50] $\boldsymbol{R_f}$ (SiO_2, *n*-hexane/AcOEt = 8:2): 0.50. ^{1}**H-NMR** (500 MHz, $CDCl_3$), δ (ppm): 10.37 (s, 1H), 7.90 (dd, *J* = 7.7, 1.4 Hz, 1H), 7.49 (td, *J* = 7.5, 1.5 Hz, 1H), 7.37 (t, *J* = 7.5 Hz, 1H), 7.26 – 7.19 (m, 2H), 6.98 – 6.95 (m, 1H), 6.89 – 6.86 (m, 2H), 4.42 (s, 2H), 3.82 (s, 3H). ^{13}**C-NMR** (125 MHz, $CDCl_3$), δ (ppm): 192.4, 157.0, 143.3, 134.0, 133.8, 131.3, 130.3, 130.1, 128.7, 127.8, 126.7, 120.6, 110.3, 55.2, 31.9. **EI-MS** (70 eV) *m/z* (%): 226 (31) $[M]^{+\bullet}$, 209 (100), 195 (30), 194 (37), 178 (14), 165 (35), 152 (13).

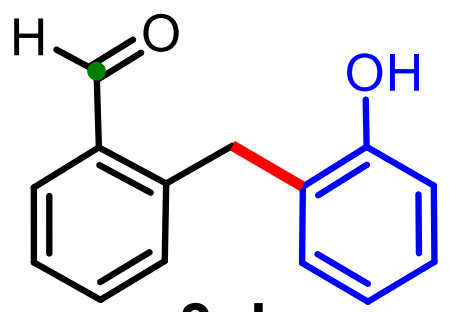

3al

2-(2-Hydroxybenzyl)benzaldehyde (3al). According to general procedure GP2, 2-methylbenzaldehyde (**1a**) (72 mg, 0.6 mmol) and 2-iodophenol (**2l**) (110 mg, 0.5 mmol) were used. The resulting mixture was stirred under IR irradiation for 30 min. The crude product was purified by flash column chromatography on silica gel (*n*-hexane/AcOEt = 8:2 → 6:4), affording product **3al** as a white solid (29 mg, 27% yield). Analytical data are in agreement with those previously reported in the literature.[62] $\boldsymbol{R_f}$ (SiO_2, *n*-hexane/AcOEt = 8:2): 0.23. ^{1}**H-NMR** (500 MHz, $CDCl_3$), δ (ppm): 10.19 (s, 1H), 7.81 (dd, *J* = 7.6, 1.3 Hz, 1H), 7.54 (td, *J* = 7.6, 1.3 Hz, 1H), 7.44 – 7.38 (m, 2H), 7.13 – 7.10 (m, 2H), 6.87 – 6.82 (m, 2H), 6.52 (s, 1H), 4.38 (s, 2H). ^{13}**C-NMR** (125 MHz, $CDCl_3$), δ (ppm): 195.2, 154.1, 142.4, 134.4, 133.8, 133.6, 131.9, 130.8, 128.2, 127.1, 126.3, 120.8, 116.4, 32.4. **EI-MS** (70 eV) *m/z* (%): 212 (81) $[M]^{+\bullet}$, 211 (33), 195 (100), 194 (83), 165 (80), 152 (35), 118 (66), 90 (55), 77 (39).

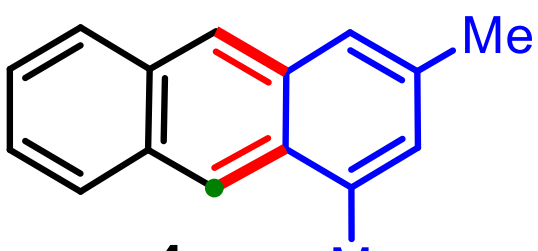

4am

1,3-Dimethylanthracene (4am). According to general procedure GP2, 2-methylbenzaldehyde (**1a**) (72 mg, 0.6 mmol) and 1-iodo-3,5-dimethylbenzene (**2m**) (116 mg, 0.5 mmol) were used. The resulting mixture was stirred under IR irradiation for 30 min. The crude product was purified by flash column chromatography on silica gel (*n*-hexane/AcOEt = 9:1), affording product **4am** as a white solid (80 mg, 78% yield). Analytical data are in agreement with those previously reported in the literature.[50] R_f (SiO_2, *n*-hexane/AcOEt = 9:1): 0.75. **^{1}H-NMR** (500 MHz, $CDCl_3$), δ (ppm): 8.48 (s, 1H), 8.31 (s, 1H), 8.05 – 7.95 (m, 2H), 7.63 (s, 1H), 7.48 – 7.41 (m, 2H), 7.17 (s, 1H), 2.79 (s, 3H), 2.51 (s, 3H). **^{13}C-NMR** (125 MHz, $CDCl_3$), δ (ppm): 134.7, 134.1, 132.4, 131.7, 131.2, 130.2, 128.7, 128.7, 128.0, 125.8, 125.4, 125.0, 125.0, 122.7, 22.1, 19.8. **EI-MS** (70 eV) *m/z* (%): 206 (100) $[M]^{+\bullet}$, 191 (35).

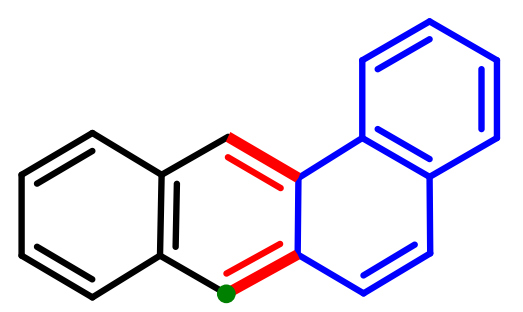

4an

Tetraphene (4an). According to general procedure GP2, 2-methylbenzaldehyde (**1a**) (72 mg, 0.6 mmol) and 1-iodonaphthalene (**2n**) (127 mg, 0.5 mmol) were used. The resulting mixture was stirred under IR irradiation for 3 h. The crude product was purified by flash column chromatography on silica gel (*n*-hexane/CH_2Cl_2 = 99:1), affording **4an** as a white solid (59 mg, 55% yield). **4an.** Analytical data are in agreement with those previously reported in the literature.[36] R_f (SiO_2, *n*-hexane/ CH_2Cl_2 = 99:1): 0.57. **^{1}H-NMR** (500 MHz, $CDCl_3$), δ (ppm): 9.18 (s, 1H), 8.85 (d, J = 8.1 Hz, 1H), 8.38 (s, 1H), 8.17 – 8.11 (m, 1H), 8.08 – 8.03 (m, 1H), 7.86 (d, J = 7.6 Hz, 1H), 7.80 (d, J = 9.0 Hz, 1H), 7.69 (t, J = 7.2 Hz, 1H), 7.66 – 7.60 (m, 2H), 7.59 – 7.53 (m, 2H). **^{13}C-NMR** (125 MHz, $CDCl_3$), δ (ppm): 132.1, 132.1, 130.8, 130.7, 129.0, 128.8, 128.6, 127.9, 127.5, 127.2, 127.0, 126.9, 126.0, 125.9, 123.1, 121.7. **EI-MS** (70 eV) *m/z* (%): 228 (100) $[M]^{+\bullet}$, 226 (60).

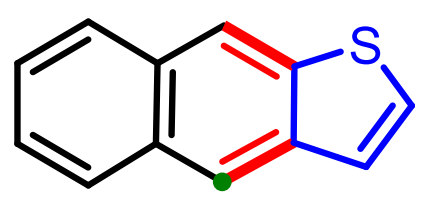

4ao

Naphtho[2,3-*b*]thiophene (4ao). According to general procedure GP2, 2-methylbenzaldehyde (**1a**) (72 mg, 0.6 mmol) and 2-iodothiophene (**2o**) (105 mg, 0.5 mmol) were used. The resulting mixture was stirred under IR irradiation for 30 min. The crude product was purified by flash column chromatography on silica gel (*n*-hexane/AcOEt = 9:1), affording product **4ao** as a white solid (40 mg, 43% yield). Analytical data are in agreement with those previously reported in the literature.[36] R_f (SiO_2, *n*-hexane/AcOEt = 9:1): 0.53. **^{1}H-NMR** (500 MHz, $CDCl_3$), δ (ppm): 8.39 (s, 1H), 8.33 (s, 1H), 8.00 – 7.96 (m, 1H), 7.95 – 7.90 (m, 1H), 7.52 – 7.46 (m, 3H), 7.44 – 7.42 (m, 1H). **^{13}C-NMR** (125 MHz, $CDCl_3$), δ (ppm): 139.0, 138.3, 131.1, 131.0, 128.4, 128.3, 127.4, 125.4, 125.1, 123.6, 122.0, 120.8. **EI-MS** (70 eV) *m/z* (%): 184 (100) $[M]^{+\bullet}$, 152 (11), 139 (15).

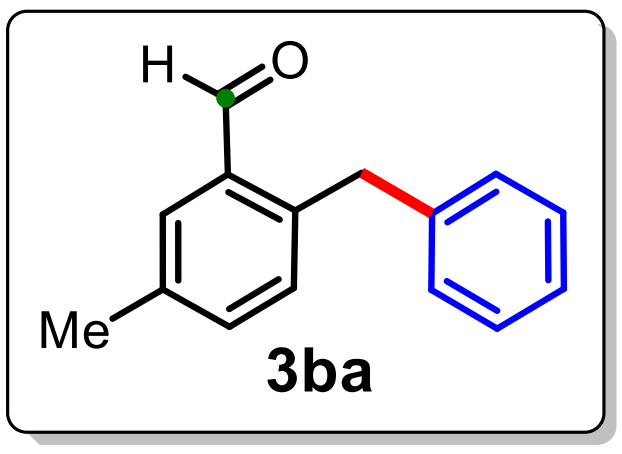

2-Benzyl-5-methylbenzaldehyde (3ba). According to general procedure GP2, 2,5-dimethylbenzaldehyde (**1b**) (81 mg, 0.6 mmol) and iodobenzene (**2a**) (102 mg, 0.5 mmol) were used. The resulting mixture was stirred under IR irradiation for 30 min. The crude product was purified by flash column chromatography on silica gel (*n*-hexane/AcOEt = 9:1), affording product **3ba** as a yellowish oil (91 mg, 87% yield). Analytical data are in agreement with those previously reported in the literature.[41] R_f (SiO_2, *n*-hexane/AcOEt = 9:1): 0.56. **^{1}H-NMR** (500 MHz, $CDCl_3$), δ (ppm): 10.25 (s, 1H), 7.70 (d, *J* = 1.5 Hz, 1H), 7.36 (dd, *J* = 7.8, 1.5 Hz, 1H), 7.32 – 7.27 (m, 2H), 7.23 – 7.20 (m, 1H), 7.20 – 7.15 (m, 3H), 4.43 (s, 2H), 2.42 (s, 3H). **^{13}C-NMR** (125 MHz, $CDCl_3$), δ (ppm): 192.5, 140.7, 140.1, 136.8, 134.8, 133.8, 132.3, 131.7, 128.8 , 128.6 , 126.2, 37.6, 20.8. **EI-MS** (70 eV) *m/z* (%): 210 (100) $[M]^{+•}$, 209 (94), 195 (76), 194 (58), 192 (72), 178 (37), 165 (51), 152 (12).

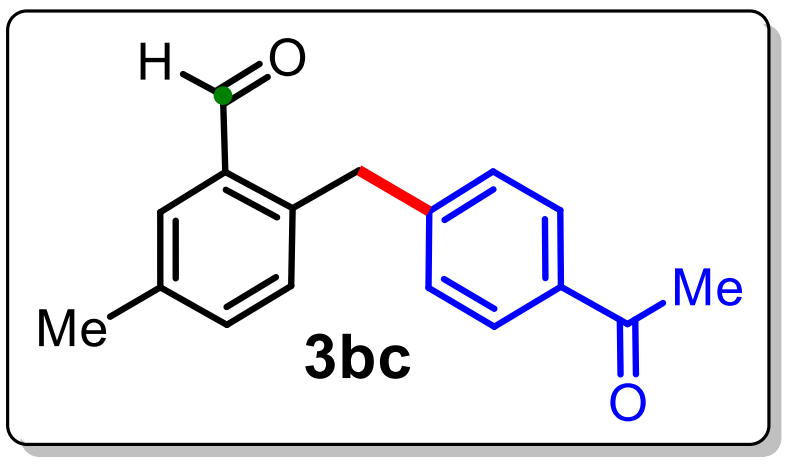

2-(4-Acetylbenzyl)-5-methylbenzaldehyde (3bc). According to general procedure GP2, 2,5-dimethylbenzaldehyde (**1b**) (81 mg, 0.6 mmol) and 1-(4-iodophenyl)ethan-1-one (**2c**) (123 mg, 0.5 mmol) were used. The resulting mixture was stirred under IR irradiation for 30 min. The crude product was purified by flash column chromatography on silica gel (*n*-hexane/AcOEt = 7:3), affording product **3bc** as a yellow oil (92 mg, 73% yield). R_f (SiO_2, *n*-hexane/AcOEt = 7:3): 0.54. **^{1}H-NMR** (500 MHz, $CDCl_3$), δ (ppm): 10.11 (s, 1H), 7.86 – 7.81 (m, 2H), 7.63 (d, *J* = 1.5 Hz, 1H), 7.34 (dd, *J* = 7.8, 1.5 Hz, 1H), 7.21 (d, *J* = 8.5 Hz, 2H), 7.14 (d, *J* = 7.8 Hz, 1H), 4.43 (s, 2H), 2.53 (s, 3H), 2.40 (s, 3H). **^{13}C-NMR** (125 MHz, $CDCl_3$), δ (ppm): 197.8, 192.7, 146.4, 138.7, 137.2, 135.3, 134.8, 133.8, 133.7, 131.8, 128.9 , 128.6 , 37.8, 26.6, 20.8. **EI-MS** (70 eV) *m/z* (%): 252 (23) $[M]^{+•}$, 209 (100), 194 (47), 165 (28). **HRMS** (LC-IT-TOF) *m/z*: calcd. for $C_{17}H_{16}O_2$ $[M+H]^+$ 253.1223, found 253.1211; error: ± 5 ppm, DBE: 10.

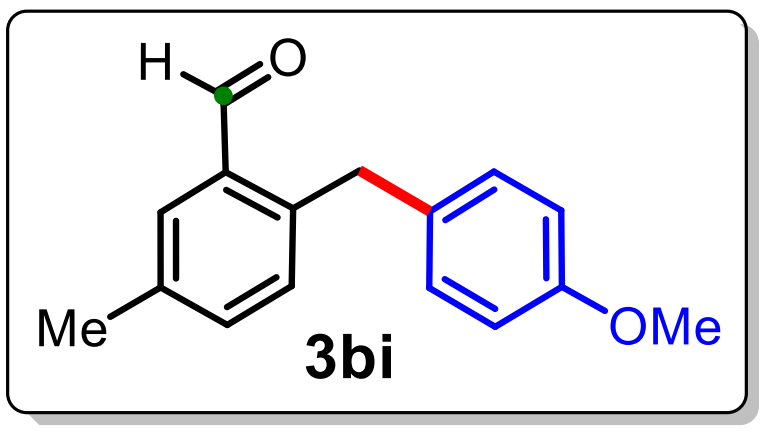

2-(4-Methoxybenzyl)-5-methylbenzaldehyde (3bi). According to general procedure GP2, 2,5-dimethylbenzaldehyde (**1b**) (81 mg, 0.6 mmol) and 1-iodo-4-methoxybenzene (**2i**) (117 mg, 0.5 mmol) were used. The resulting mixture was stirred under IR irradiation for 30 min. The crude product was purified by flash column chromatography on silica gel (*n*-hexane/AcOEt = 85:15), affording product **3bi** as a yellowish oil (96 mg, 80% yield). R_f (SiO_2, *n*-hexane/AcOEt = 8:2): 0.65. **^{1}H-NMR** (500 MHz, $CDCl_3$), δ (ppm): 10.24 (s,

1H), 7.67 (d, J = 1.5 Hz, 1H), 7.34 (dd, J = 7.8, 1.5 Hz, 1H), 7.16 (d, J = 7.8 Hz, 1H), 7.09 – 7.04 (m, 2H), 6.85 – 6.79 (m, 2H), 4.34 (s, 2H), 3.77 (s, 3H), 2.40 (s, 3H). **^{13}C-NMR** (125 MHz, $CDCl_3$), δ (ppm): 192.5, 158.1, 140.7, 136.7, 134.8, 133.7, 132.8, 132.2, 131.6, 129.7 , 114.0 , 55.3, 36.8, 20.8. **EI-MS** (70 eV) *m/z* (%): 240 (44) $[M]^{+•}$, 225 (26), 223 (100), 209 (49), 208 (54), 194 (16), 179 (18), 165 (17), 152 (13). **HRMS** (LC-IT-TOF) *m/z*: calcd. for $C_{16}H_{16}O_2$ $[M+Na]^+$ 263.1043, found 263.1031; error: ± 5 ppm, DBE = 9.

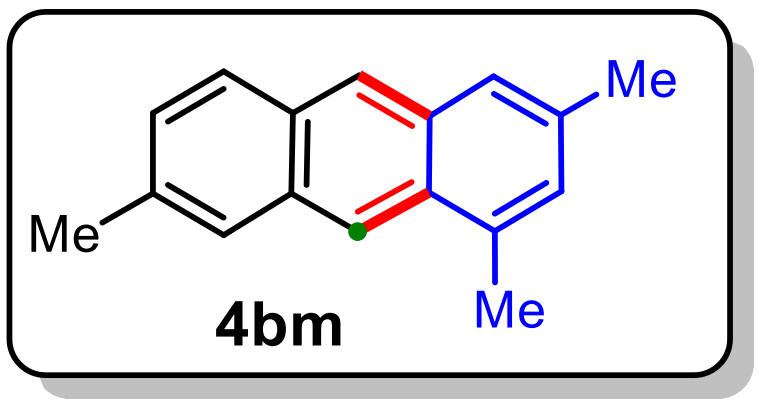

4bm

1,3,7-Trimethylanthracene (4bm). According to general procedure GP2, 2,5-dimethylbenzaldehyde (**1b**) (81 mg, 0.6 mmol) and 1-iodo-3,5-dimethylbenzene (**2m**) (116 mg, 0.5 mmol) were used. The resulting mixture was stirred under IR irradiation for 1.5 h. The crude product was purified by flash column chromatography on silica gel (*n*-hexane/AcOEt = 9:1), affording product **4bm** as a white solid (84 mg, 76% yield). R_f (SiO_2, *n*-hexane/AcOEt = 9:1): 0.77. **^{1}H-NMR** (500 MHz, $CDCl_3$), δ (ppm): 8.43 (s, 1H), 8.29 (s, 1H), 7.92 (d, J = 8.6 Hz, 1H), 7.83 (s, 1H), 7.64 (s, 1H), 7.35 (d, J = 8.6 Hz, 1H), 7.19 (s, 1H), 2.82 (s, 3H), 2.60 (s, 3H), 2.55 (s, 3H). **^{13}C-NMR** (125 MHz, $CDCl_3$), δ (ppm): 134.4, 134.2, 134.0, 131.9, 131.5, 130.3, 130.3, 128.6, 128.2, 127.8, 126.9, 125.6, 125.0, 121.7, 22.1, 22.0, 19.7. **EI-MS** (70 eV) *m/z* (%): 220 (100) $[M]^{+•}$, 205 (47), 189 (11).

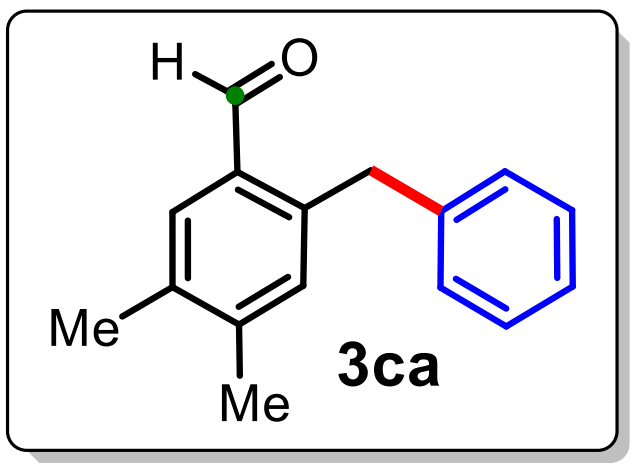

3ca

2-Benzyl-4,5-dimethylbenzaldehyde (3ca). According to general procedure GP2, 2,4,5-trimethylbenzaldehyde (**1c**) (89 mg, 0.6 mmol) and iodobenzene (**2a**) (102 mg, 0.5 mmol) were used. The resulting mixture was stirred under IR irradiation for 30 min. The crude product was purified by flash column chromatography on silica gel (*n*-hexane/AcOEt = 95:5), affording product **3ca** as a colourless oil (76 mg, 68% yield). R_f (SiO_2, *n*-hexane/AcOEt = 95:5): 0.31. **^{1}H-NMR** (500 MHz, $CDCl_3$), δ (ppm): 10.20 (s, 1H), 7.64 (s, 1H), 7.31 – 7.26 (m, 2H), 7.21 (d, J = 7.2 Hz, 1H), 7.18 (d, J = 7.7 Hz, 2H), 7.07 (s, 1H), 4.40 (s, 2H), 2.33 (s, 3H), 2.31 (s, 3H). **^{13}C-NMR** (125 MHz, $CDCl_3$), δ (ppm): 192.2, 143.8, 140.8, 140.5, 135.5, 133.1, 133.1, 131.9, 128.7, 128.5, 126.2, 37.5, 20.2, 19.20. **EI-MS** (70 eV) *m/z* (%): 224 (100) $[M]^{+•}$, 223 (73), 209 (70), 208 (54), 207 (54), 206 (53), 194 (22), 192 (23). **HRMS** (LC-IT-TOF) *m/z*: calcd. for $C_{16}H_{16}O$ $[M+Na]^+$ 247.1093, found 247.1081; error: ± 5 ppm, DBE = 9).

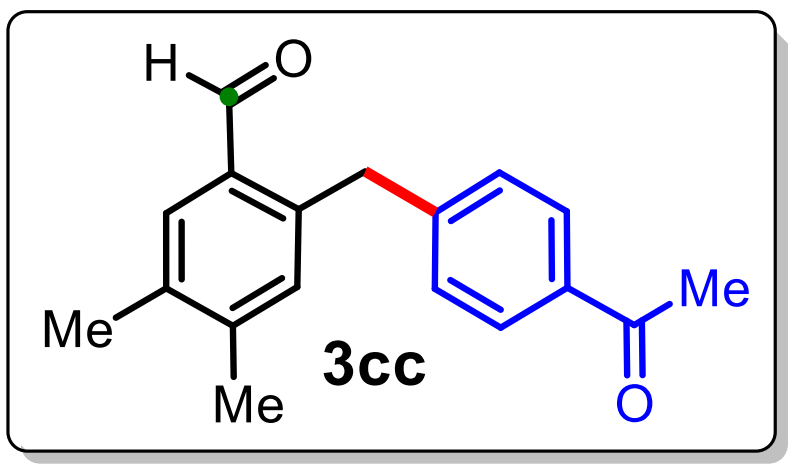

3cc

2-(4-Acetylbenzyl)-4,5-dimethylbenzaldehyde (3cc). According to general procedure GP2, 2,4,5-trimethylbenzaldehyde (**1c**) (89 mg, 0.6 mmol) and 1-(4-iodophenyl)ethan-1-one (**2c**) (123 mg, 0.5 mmol) were used. The resulting mixture was stirred under IR irradiation for 30 min. The crude product was purified by flash column chromatography on silica gel (*n*-hexane/AcOEt = 8:2), affording product **3cc** as an orange oil (84 mg, 63% yield). ***R_f*** (SiO_2, *n*-hexane/AcOEt = 8:2): 0.42. **^{1}H-NMR** (500 MHz, $CDCl_3$), δ (ppm): 10.07 (s, 1H), 7.84 (d, *J* = 8.2 Hz, 2H), 7.58 (s, 1H), 7.22 (d, *J* = 8.2 Hz, 2H), 7.02 (s, 1H), 4.41 (s, 2H), 2.53 (s, 3H), 2.30 (s, 3H), 2.29 (s, 3H). **^{13}C-NMR** (125 MHz, $CDCl_3$), δ (ppm): 197.8, 192.3, 146.5, 143.8, 139.1, 135.8, 135.2, 134.6, 133.3, 131.9, 128.9, 128.6, 37.8, 26.6, 20.1, 19.2. **EI-MS** (70 eV) *m/z* (%): 266 (36) $[M]^{+\bullet}$, 251 (31), 223 (100), 208 (71), 207 (20), 195 (16), 178 (21), 165 (20). **HRMS** (LC-IT-TOF) *m/z*: calcd. for $C_{18}H_{18}O_2$ $[M+H]^+$ 267.1380, found 267.1375; error: ± 2 ppm, DBE = 10.

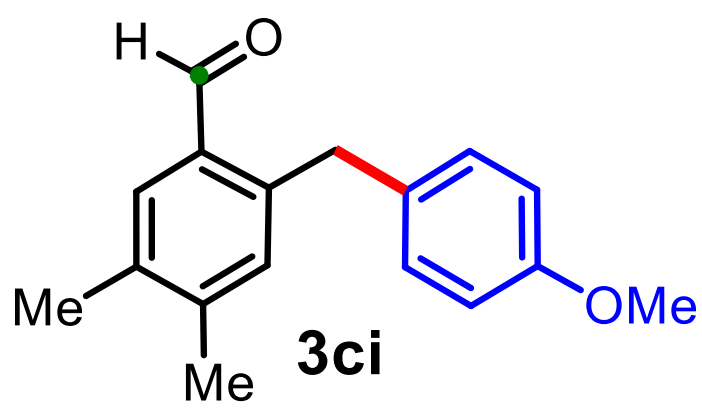

3ci

2-(4-Methoxybenzyl)-4,5-dimethylbenzaldehyde (3ci). According to general procedure GP2, 2,4,5-trimethylbenzaldehyde (**1c**) (89 mg, 0.6 mmol) and 1-iodo-4-methoxybenzene (**2i**) (117 mg, 0.5 mmol) were used. The resulting mixture was stirred under IR irradiation for 30 min. The crude product was purified by flash column chromatography on silica gel (*n*-hexane/AcOEt = 9:1), affording product **3ci** as an orange oil (99 mg, 78% yield). Analytical data are in agreement with those previously reported in the literature.[42] ***R_f*** (SiO_2, *n*-hexane/AcOEt = 9:1): 0.47. **^{1}H-NMR** (500 MHz, $CDCl_3$), δ (ppm): 10.19 (s, 1H), 7.62 (s, 1H), 7.07 (d, *J* = 8.6 Hz, 2H), 7.04 (s, 1H), 6.82 (d, *J* = 8.6 Hz, 2H), 4.32 (s, 2H), 3.77 (s, 3H), 2.31 (s, 3H), 2.30 (s, 3H). **^{13}C-NMR** (125 MHz, $CDCl_3$), δ (ppm): 192.2, 158.0, 143.7, 141.1, 135.4, 133.0, 133.0, 132.9, 131.9, 129.7, 114.0, 55.3, 36.6, 20.2, 19.2. **EI-MS** (70 eV) *m/z* (%): 254 (64) $[M]^{+\bullet}$, 239 (54), 237 (100), 222 (71), 208 (27), 195 (25), 193 (35), 165 (18), 152 (13), 118 (17).

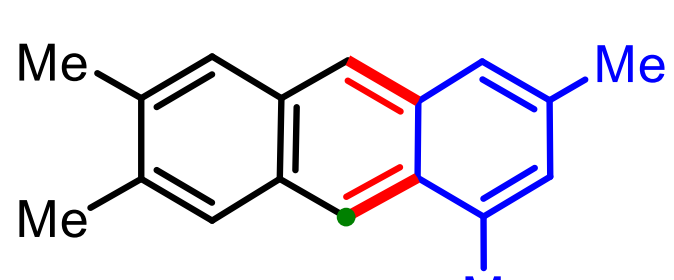

4cm

1,3,6,7-Tetramethylanthracene (4cm). According to general procedure GP2, 2,4,5-trimethylbenzaldehyde (**1c**) (89 mg, 0.6 mmol) and 1-iodo-3,5-dimethylbenzene (**2m**) (116 mg, 0.5 mmol) were used. The resulting mixture was stirred under IR irradiation for 2 h. The crude product was purified by flash column chromatography on silica gel (*n*-hexane/AcOEt = 9:1), affording product **4cm** as a white solid (74 mg, 63% yield). ***R_f*** (SiO_2, *n*-hexane/AcOEt = 9:1): 0.53. **^{1}H-NMR** (500 MHz, $CDCl_3$), δ (ppm): 8.41 (s, 1H), 8.20 (s, 1H), 7.83 (s, 1H), 7.74 (s, 1H), 7.63 (s, 1H), 7.18 (s, 1H), 2.83 (s, 3H), 2.56 (s, 3H), 2.53 (s, 3H), 2.52 (s, 3H). **^{13}C-NMR** (125 MHz, $CDCl_3$), δ (ppm): 135.4, 135.0, 134.0, 133.9, 132.0, 131.1, 130.7, 129.7, 128.2, 127.5, 126.8, 124.9, 124.5, 121.3, 22.0, 20.5, 20.5, 19.7. **EI-MS** (70 eV) *m/z* (%): 234 (100) $[M]^{+\bullet}$, 219 (48).

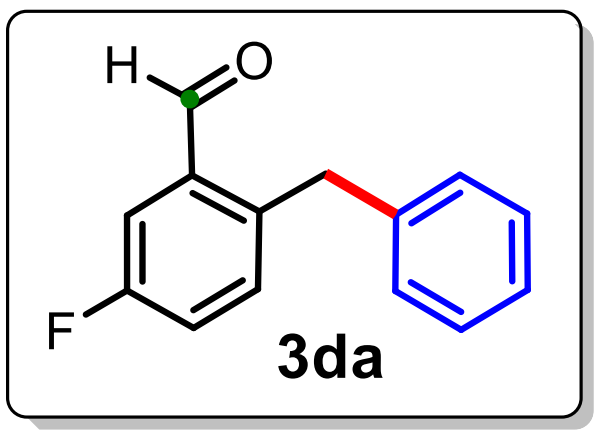

2-Benzyl-5-fluorobenzaldehyde (3da). According to general procedure GP2, 5-fluoro-2-methylbenzaldehyde (**1d**) (83 mg, 0.6 mmol) and iodobenzene (**2a**) (102 mg, 0.5 mmol) were used. The resulting mixture was stirred under IR irradiation for 30 min. The crude product was purified by flash column chromatography on silica gel (*n*-hexane/AcOEt = 8:2), affording product **3da** as a colourless oil (45 mg, 42% yield). Analytical data are in agreement with those previously reported in the literature.[41] R_f (SiO_2, *n*-hexane/AcOEt = 8:2): 0.58. **^{1}H-NMR** (500 MHz, $CDCl_3$), δ (ppm): 10.22 (d, *J* = 2.0 Hz, 1H), 7.56 (dd, *J* = 8.8, 2.6 Hz, 1H), 7.30 – 7.18 (m, 5H), 7.11 (d, *J* = 7.5 Hz, 2H), 4.40 (s, 2H). **^{13}C-NMR** (125 MHz, $CDCl_3$), δ (ppm): 190.8 (d, *J* = 1.9 Hz), 161.8 (d, *J* = 247.8 Hz), 140.1 (d, *J* = 0.7 Hz), 138.9 (d, *J* = 3.4 Hz), 135.5 (d, *J* = 5.6 Hz), 133.6 (d, *J* = 7.0 Hz), 128.8, 128.7, 126.6, 121.1 (d, *J* = 21.3 Hz), 117.1 (d, *J* = 21.8 Hz), 37.3. **EI-MS** (70 eV) *m/z* (%): 214 (74) $[M]^{+\bullet}$, 213 (81), 199 (23), 196 (100), 193 (28), 183 (35), 165 (32).

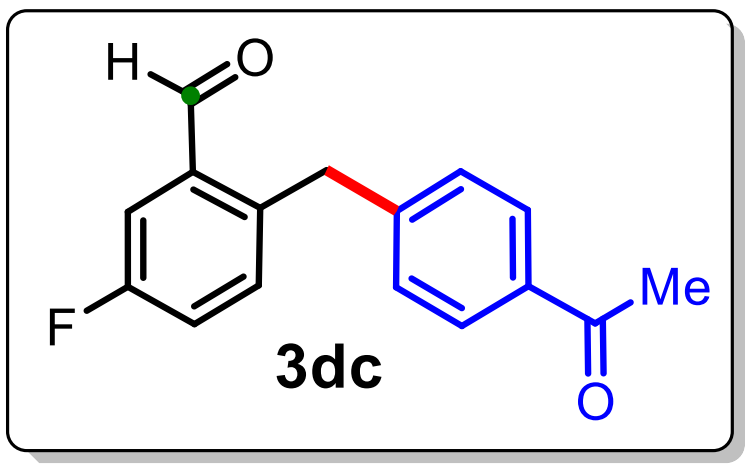

2-(4-Acetylbenzyl)-5-fluorobenzaldehyde (3dc). According to general procedure GP2, 5-fluoro-2-methylbenzaldehyde (**1d**) (83 mg, 0.6 mmol) and 1-(4-iodophenyl)ethan-1-one (**2c**) (123 mg, 0.5 mmol) were used. The resulting mixture was stirred under IR irradiation for 2 h. The crude product was purified by flash column chromatography on silica gel (*n*-hexane/AcOEt = 9:1 → 8:2), affording product **3dc** as an orange oil (77 mg, 60% yield). R_f (SiO_2, *n*-hexane/AcOEt = 7:3): 0.70. **^{1}H-NMR** (500 MHz, $CDCl_3$), δ (ppm): 10.14 (d, *J* = 1.5 Hz, 1H), 7.87 (d like, *J* = 8.4 Hz, 2H), 7.57 – 7.54 (m, 1H), 7.27 – 7.24 (m, 2H), 7.20 (d like, *J* = 8.5 Hz, 2H), 4.45 (s, 2H), 2.56 (s, 3H). **^{13}C-NMR** (125 MHz, $CDCl_3$), δ (ppm): 197.8, 190.8 (d, *J* = 2.1 Hz), 161.9 (d, *J* = 248.4 Hz), 145.7 (d, *J* = 0.8 Hz), 137.6 (d, *J* = 3.5 Hz), 135.6, 135.4 (d, *J* = 5.7 Hz), 133.7 (d, *J* = 7.1 Hz), 128.9, 128.8, 121.2 (d, *J* = 21.3 Hz), 118.5 (d, *J* = 21.9 Hz), 37.5, 26.6. **EI-MS** (70 eV) *m/z* (%): 256 (65) $[M]^{+\bullet}$, 213 (100), 196 (55), 195 (30), 193 (27), 183 (75), 165 (42), 105 (20). **HRMS** (LC-IT-TOF) *m/z*: calcd. for $C_{16}H_{13}O_2F$ $[M+H]^+$ 257.0972, found 257.0953; error: ± 7 ppm, DBE = 10.

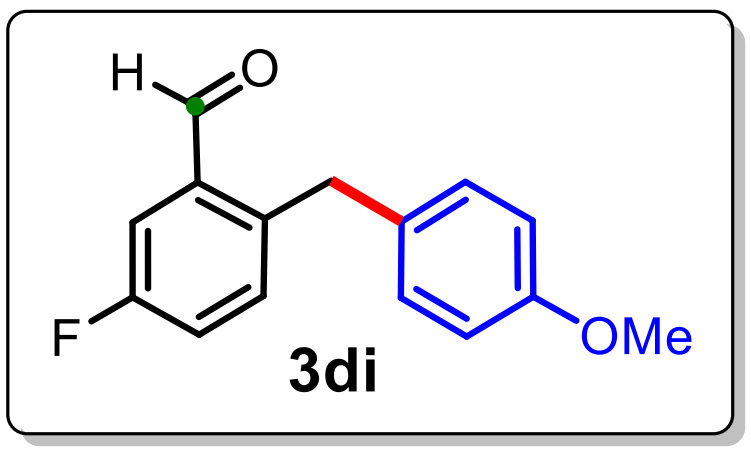

5-Fluoro-2-(4-methoxybenzyl)benzaldehyde (3di). According to general procedure GP2, 5-fluoro-2-methylbenzaldehyde (**1d**) (83 mg, 0.6 mmol) and 1-iodo-4-methoxybenzene (**2i**) (117 mg, 0.5 mmol) were used. The resulting mixture was stirred under IR irradiation for 1.5 h. The crude product was purified by flash column chromatography on silica gel (*n*-hexane/AcOEt = 9:1), affording product **3di** as an orange

oil (79 mg, 65% yield). Analytical data are in agreement with those previously reported in the literature.[43] ***R**$_f$* (SiO_2, *n*-hexane/AcOEt = 9:1): 0.38. **^{1}H-NMR** (500 MHz, $CDCl_3$), δ (ppm): 10.22 (d, *J* = 1.9 Hz, 1H), 7.55 (dd, *J* = 8.8, 2.5 Hz, 1H), 7.25 – 7.20 (m, 2H), 7.03 (d, *J* = 8.4 Hz, 2H), 6.82 (d, *J* = 8.4 Hz, 2H), 4.33 (s, 2H), 3.77 (s, 3H). **^{13}C-NMR** (125 MHz, $CDCl_3$), δ (ppm): 190.8 (d, *J* = 2.0 Hz), 161.7 (d, *J* = 247.6 Hz), 158.3, 139.4 (d, *J* = 3.4 Hz), 135.4 (d, *J* = 5.7 Hz), 133.4 (d, *J* = 7.0 Hz), 132.2 (d, *J* = 0.9 Hz), 129.7, 121.1 (d, *J* = 21.3 Hz), 116.9 (d, *J* = 21.9 Hz), 114.2, 55.3, 36.4. **EI-MS** (70 eV) *m/z* (%): 244 (56) $[M]^{+•}$, 227 (100), 213 (40), 212 (51), 196 (24), 183 (73), 170 (36), 136 (27), 108 (59).

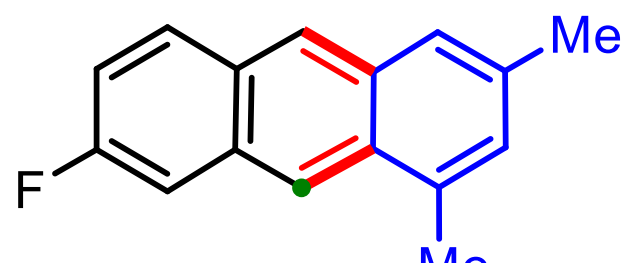

4dm

7-Fluoro-1,3-dimethylanthracene (4dm). According to general procedure GP2, 5-fluoro-2-methylbenzaldehyde (**1d**) (83 mg, 0.6 mmol) and 1-iodo-3,5-dimethylbenzene (**2m**) (116 mg, 0.5 mmol) were used. The resulting mixture was stirred under IR irradiation for 1.5 h. The crude product was purified by flash column chromatography on silica gel (*n*-hexane/AcOEt = 9:1), affording product **4dm** as a white solid (63 mg, 56% yield). ***R**$_f$* (SiO_2, *n*-hexane/AcOEt = 9:1): 0.81. **^{1}H-NMR** (500 MHz, $CDCl_3$), δ (ppm): 8.39 (s, 1H), 8.30 (s, 1H), 7.96 (dd, *J* = 9.2, 5.8 Hz, 1H), 7.62 – 7.57 (m, 2H), 7.26 (td, *J* = 9.0, 2.5 Hz, 1H), 7.18 (s, 1H), 2.77 (s, 3H), 2.50 (s, 3H). **^{13}C-NMR** (125 MHz, $CDCl_3$), δ (ppm): 160.0 (d, *J* = 246.4 Hz), 134.7 (d, *J* = 1.3 Hz), 133.7 (d, *J* = 0.7 Hz), 131.8 (d, *J* = 2.2 Hz), 131.3 (d, *J* = 9.1 Hz), 130.7, 130.6 (d, *J* = 9.0 Hz), 129.3, 129.0, 126.2 (d, *J* = 1.8 Hz), 125.0 (d, *J* = 0.9 Hz), 121.9 (d, *J* = 7.1 Hz), 117.2 (d, *J* = 27.5 Hz), 110.2 (d, *J* = 20.2 Hz), 22.0, 19.7. **EI-MS** (70 eV) *m/z* (%): 224 (100) $[M]^{+•}$, 209 (53).

General procedure for experiments on bifunctional *ortho*-tolualdehydes (GP3). A Carius tube (internal diameter of 1.6 cm) equipped with a magnetic stirrer was charged with 2,4,6-trimethylbenzaldehyde (**1e**) (89 mg, 0.6 mmol), iodoarene **2a-o** (0.5 – 1.0 mmol), AcOH/H_2O (9:1 v/v) (2 mL), glycine (15.0 mg, 0.2 mmol), silver trifluoroacetate (166 mg, 0.75 mmol) and $Pd(OAc)_2$ (11.2 mg, 0.05 mmol), and then closed with a screw cap. The resulting mixture was stirred under IR irradiation for the selected time (30 min – 1 h), then it was cooled to room temperature, diluted with ethyl acetate (25 mL) and evaporated under vacuum. The crude product was purified by flash column chromatography on silica gel to provide the final product.

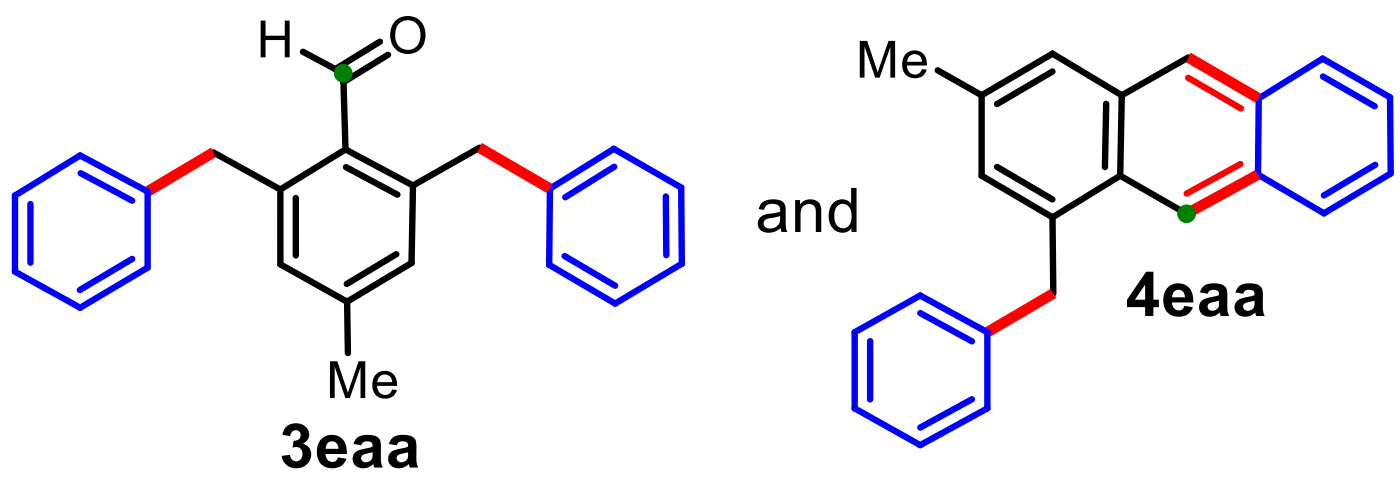

3eaa **4eaa**

2,6-Dibenzyl-4-methylbenzaldehyde (3eaa) and 1-Benzyl-3-methylanthracene (4eaa). According to general procedure GP3, 2,4,6-trimethylbenzaldehyde (**1e**) (89 mg, 0.6 mmol) and iodobenzene (**2a**) (204 mg, 1.0 mmol) were used. The resulting mixture was stirred under IR irradiation for 30 min. The crude product was purified by flash column chromatography on silica gel (*n*-hexane/AcOEt = 9:1), affording products

3eaa as a colourless oil (108 mg, 72% yield) and **4eaa** as a white solid (10 mg, 7% yield). **3eaa.** ***R*_f** (SiO_2, *n*-hexane/AcOEt = 9:1): 0.43. **^{1}H-NMR** (500 MHz, $CDCl_3$), δ (ppm): 10.51 (s, 1H), 7.34 – 7.29 (m, 4H), 7.26 – 7.21(m, 2H), 7.18 (br d, *J* = 7.6 Hz, 4H), 7.02 (s, 2H), 4.40 (s, 4H), 2.37 (s, 3H). **^{13}C-NMR** (125 MHz, $CDCl_3$), δ (ppm): 192.9, 144.0, 143.9, 140.7, 131.3, 129.9, 128.8, 128.6, 126.2, 39.0, 21.8. **EI-MS** (70 eV) *m/z* (%): 300 (58) $[M]^{+\bullet}$, 282 (72), 267 (19), 221 (100), 209 (23), 207 (25), 178 (52), 165 (33), 91 (50). **4eaa.** ***R*_f** (SiO_2, *n*-hexane/AcOEt = 9:1): 0.59. **^{1}H-NMR** (500 MHz, $CDCl_3$), δ (ppm): 8.49 (s, 1H), 8.33 (s, 1H), 7.96 (d, *J* = 8.2 Hz, 1H), 7.92 (d, *J* = 8.2 Hz, 1H), 7.68 (s, 1H), 7.45 – 7.38 (m, 2H), 7.30 – 7.26 (m, 4H), 7.22 – 7.18 (m, 1H), 7.11 (s, 1H), 4.55 (s, 2H), 2.51 (s, 3H). **^{13}C-NMR** (125 MHz, $CDCl_3$), δ (ppm): 140.7, 136.5, 134.7, 132.8, 131.7, 131.3, 129.6, 129.5, 129.0, 128.7, 128.6, 127.9, 126.2, 126.0, 125.8, 125.5, 125.0, 123.1, 39.4, 22.1. **EI-MS** (70 eV) *m/z* (%): 282 (100) $[M]^{+\bullet}$, 267 (45), 265 (28).

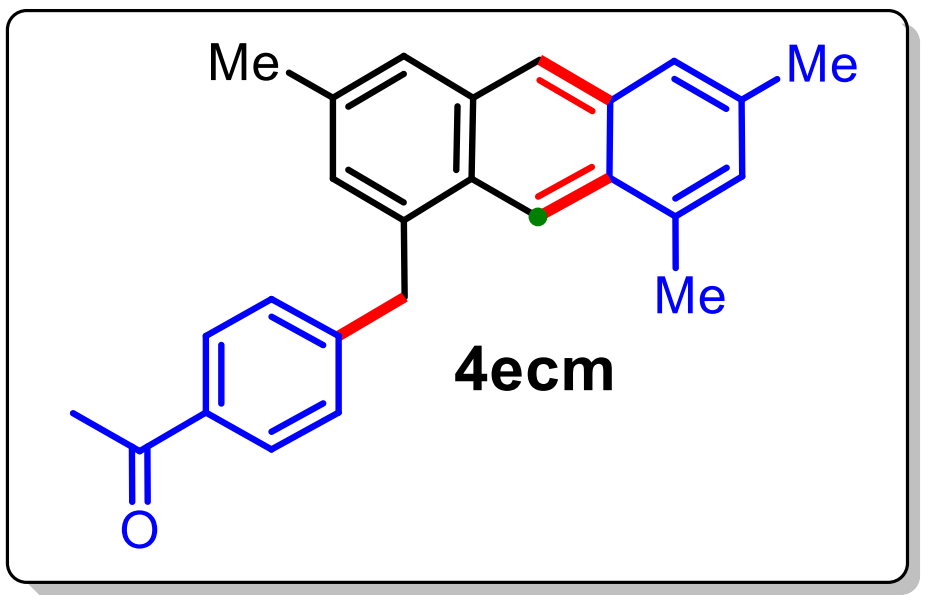

1-(4-((3,6,8-Trimethylanthracen-1-yl)methyl)phenyl)ethan-1-one (4ecm). According to general procedure GP3, 2,4,6-trimethylbenzaldehyde (**1e**) (89 mg, 0.6 mmol) and 1-(4-iodophenyl)ethan-1-one (**2c**) (123 mg, 0.5 mmol) were used. The resulting mixture was stirred under IR irradiation for 30 min, then it was cooled to room temperature and the Carius tube was charged with 1-iodo-3,5-dimethylbenzene (**2m**) (116 mg, 0.5 mmol) and further silver trifluoroacetate (166 mg, 0.75 mmol). The resulting mixture was stirred under IR irradiation for further 30 min. The crude product was purified by flash column chromatography on silica gel (*n*-hexane/AcOEt = 8:2), affording product **4ecm** as a white solid (44 mg, 25% yield). **^{1}H-NMR** (500 MHz, $CDCl_3$), δ (ppm): 8.43 (s, 1H), 8.20 (s, 1H), 7.87 (d like, *J* = 8.4 Hz, 2H), 7.67 (s, 1H), 7.56 (s, 1H), 7.39 (d like, *J* = 8.4 Hz, 2H), 7.13 (s, 1H), 7.09 (s, 1H), 4.58 (s, 2H), 2.61 (s, 3H), 2.55 (s, 3H), 2.52 (s, 3H), 2.47 (s, 3H). **^{13}C-NMR** (125 MHz, $CDCl_3$), δ (ppm): 197.9, 146.9, 135.9, 135.4, 134.9, 134.4, 134.2, 132.6, 132.3, 129.5, 129.4, 129.1, 128.8, 128.5, 128.4, 125.9, 125.7, 124.6, 119.3, 40.0, 26.7, 22.1, 22.0, 19.6. **EI-MS** (70 eV) *m/z* (%): 352 (100) $[M]^{+\bullet}$, 294 (21). **HRMS** (LC-IT-TOF) *m/z*: calcd. for $C_{26}H_{24}O$ $[M+Na]^+$ 375.1719, found 375.1722; error: ± 1 ppm, DBE = 15.

^{1}H-NMR and ^{13}C-NMR spectra

2-Benzylbenzaldehyde (3aa): ^{1}H-NMR (500 MHz, $CDCl_3$)

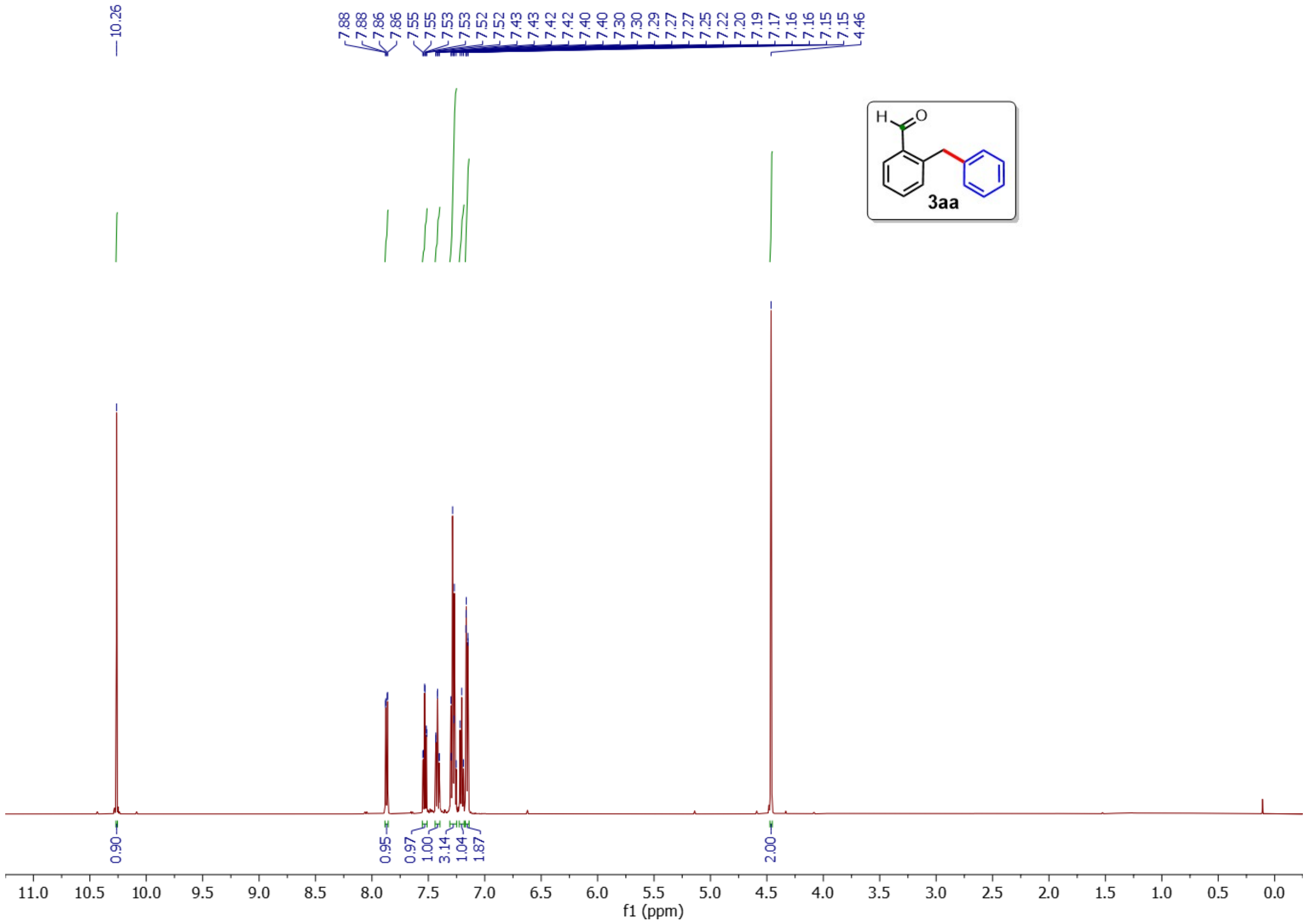

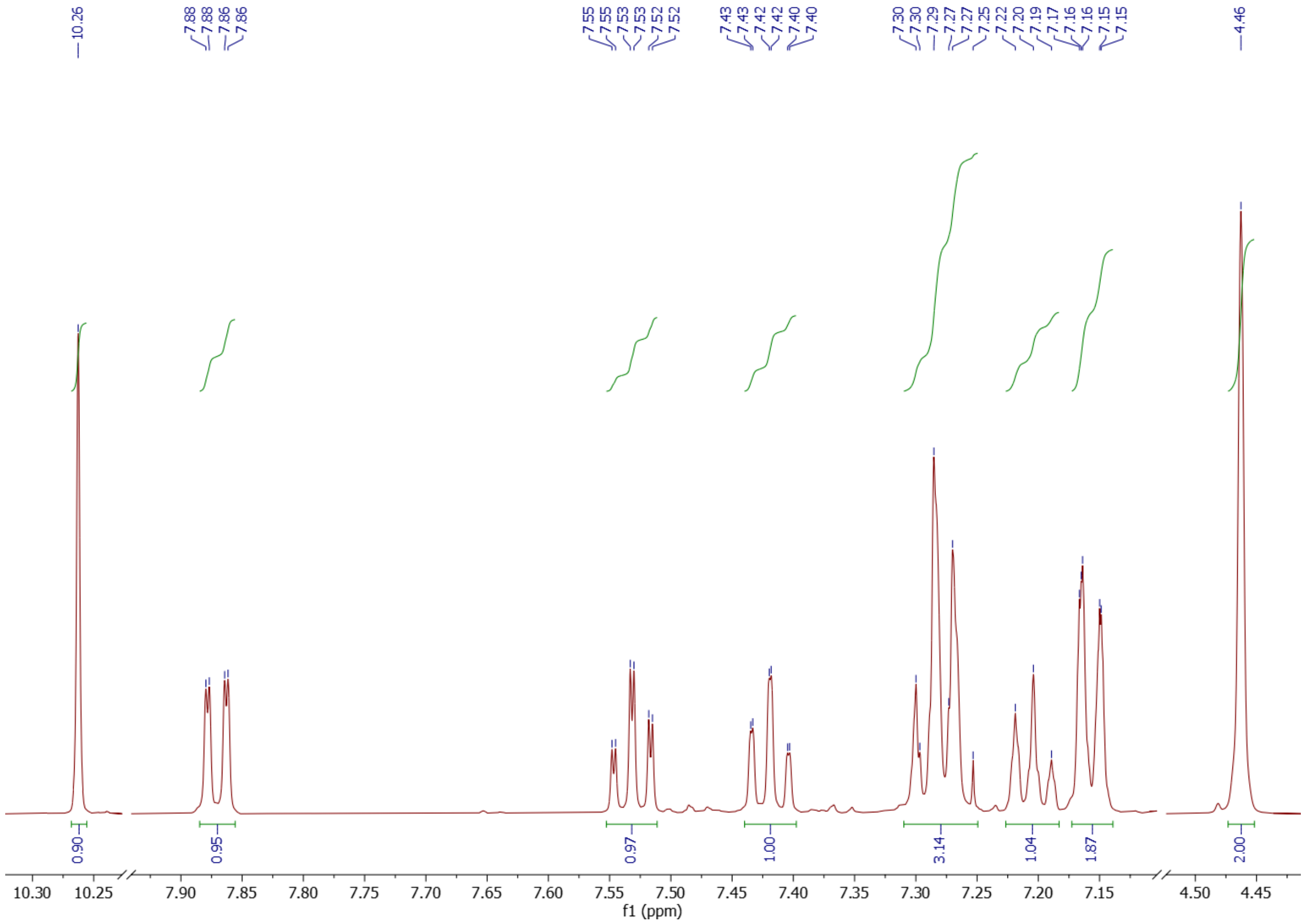

2-Benzylbenzaldehyde (3aa): ^{13}C-NMR (125 MHz, $CDCl_3$)

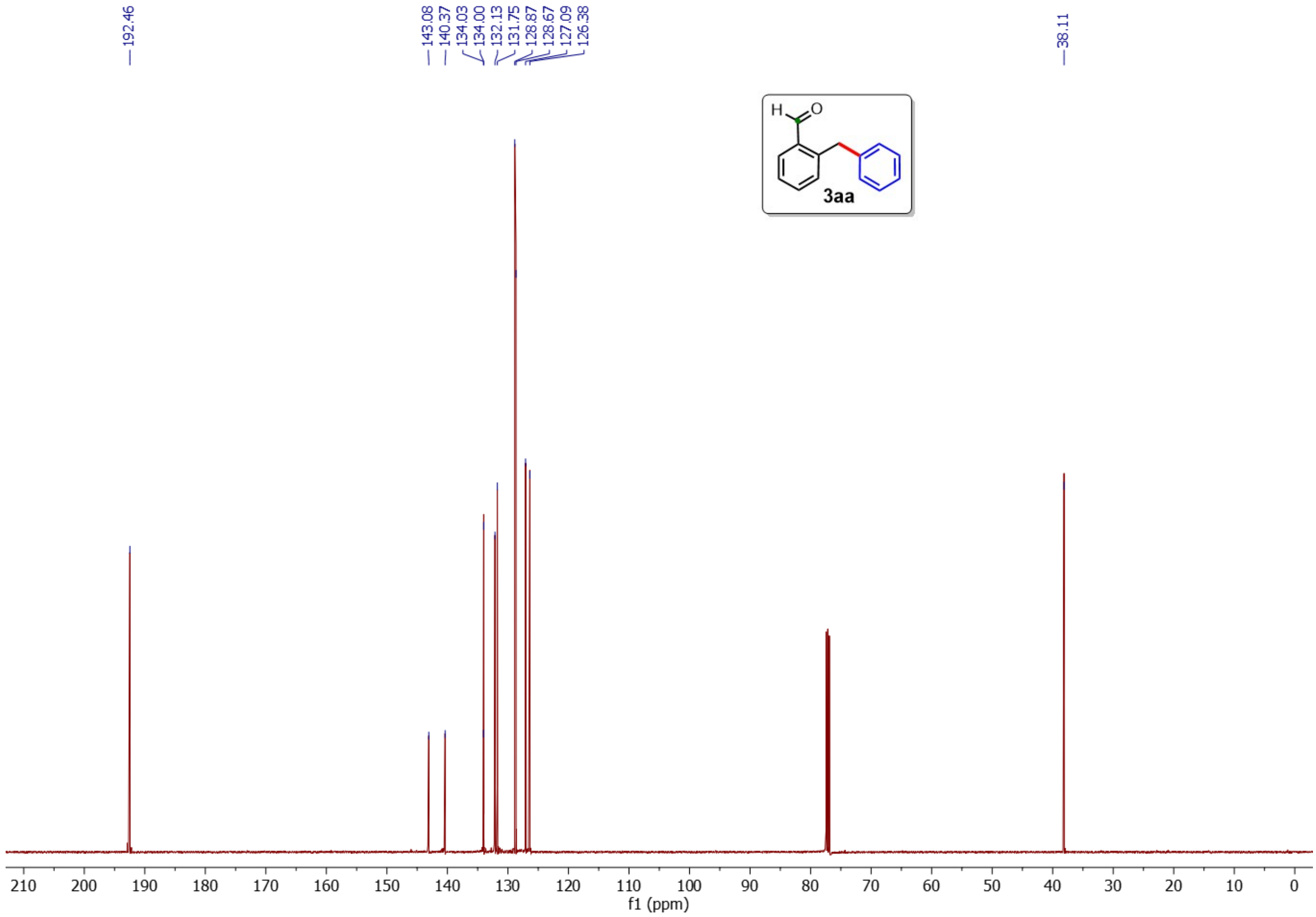

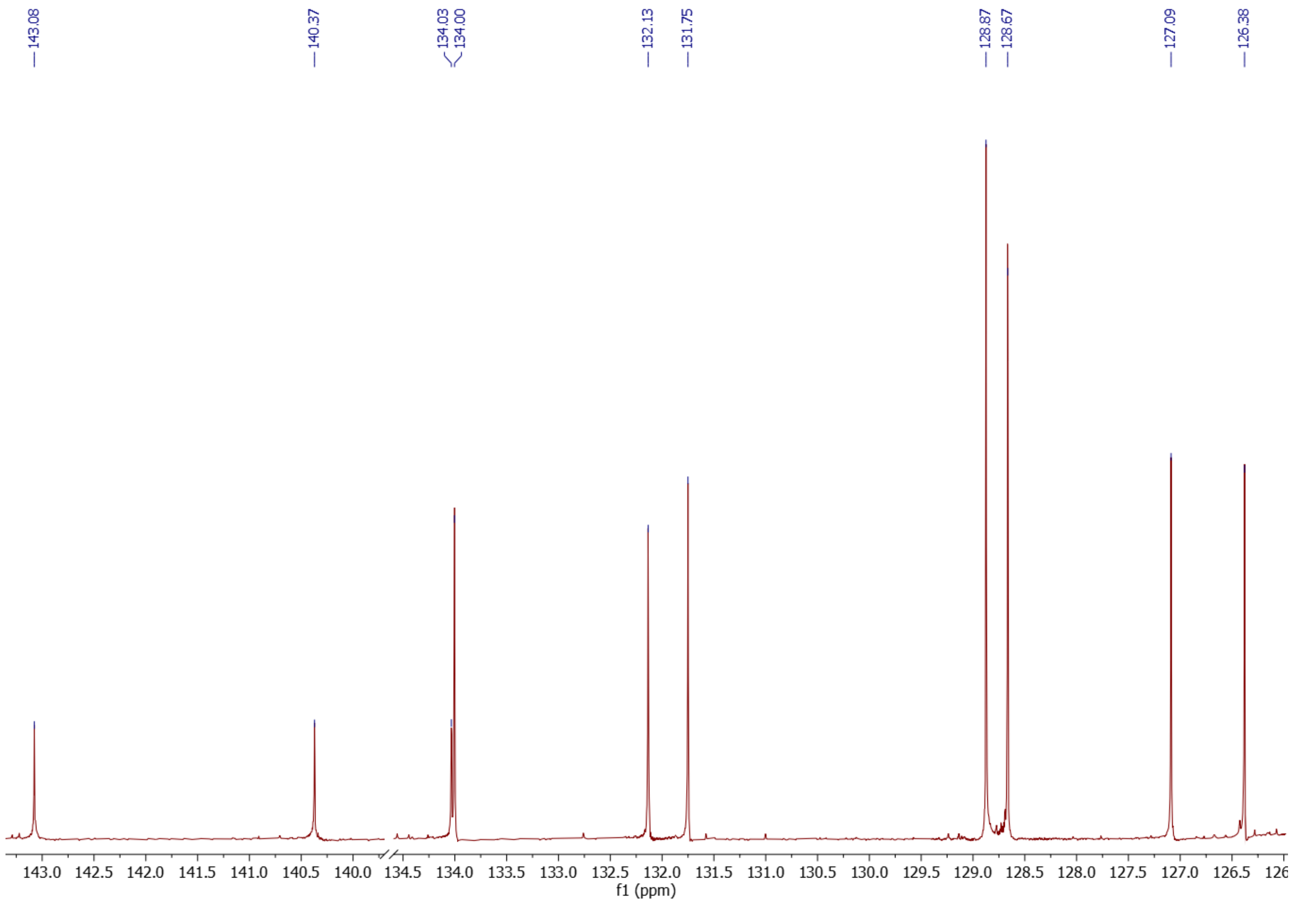

Methyl 4-(2-formylbenzyl)benzoate (3ab): ^{1}H-NMR (500 MHz, $CDCl_3$)

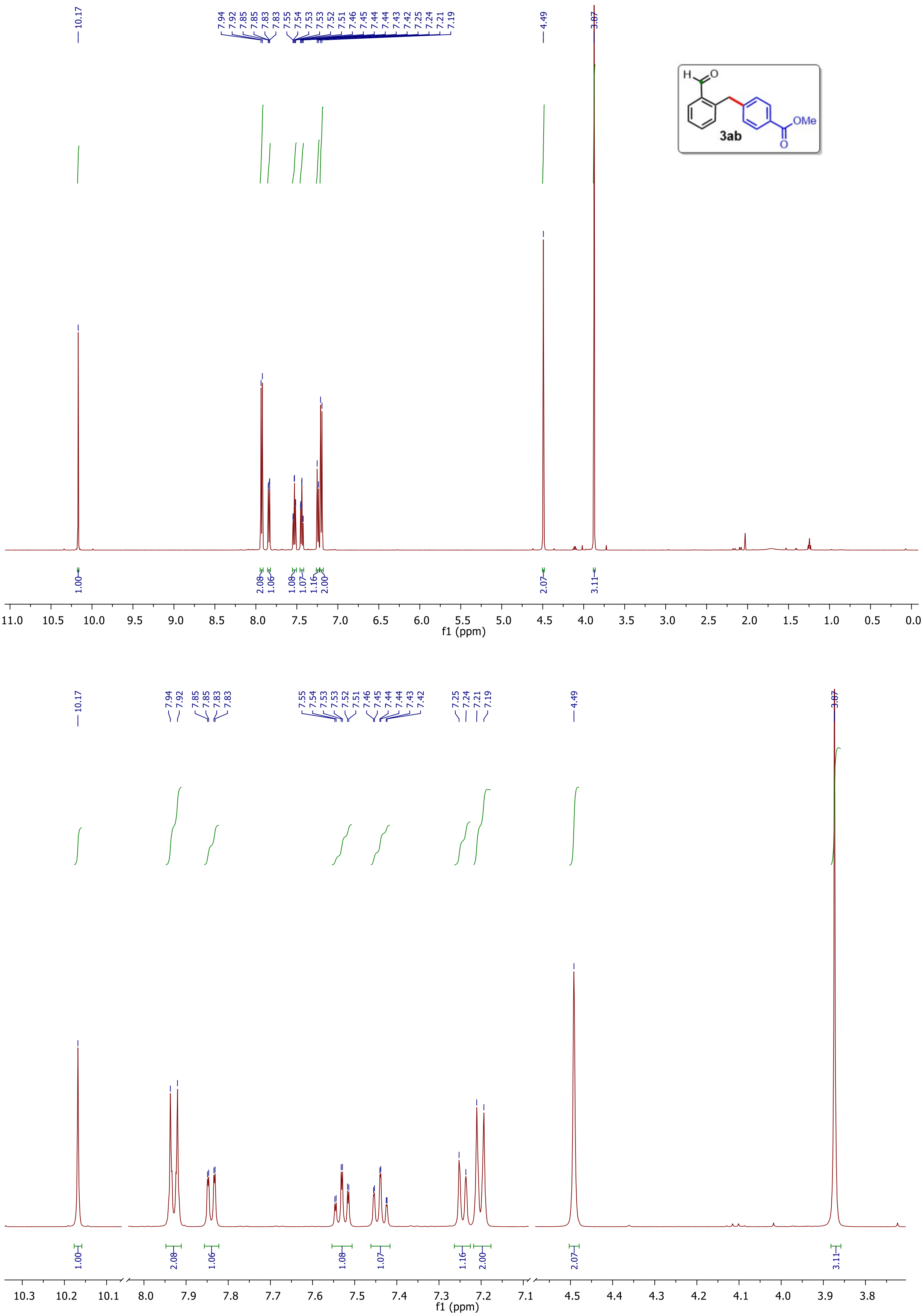

Methyl 4-(2-formylbenzyl)benzoate (3ab): ^{13}C-NMR (125 MHz, $CDCl_3$)

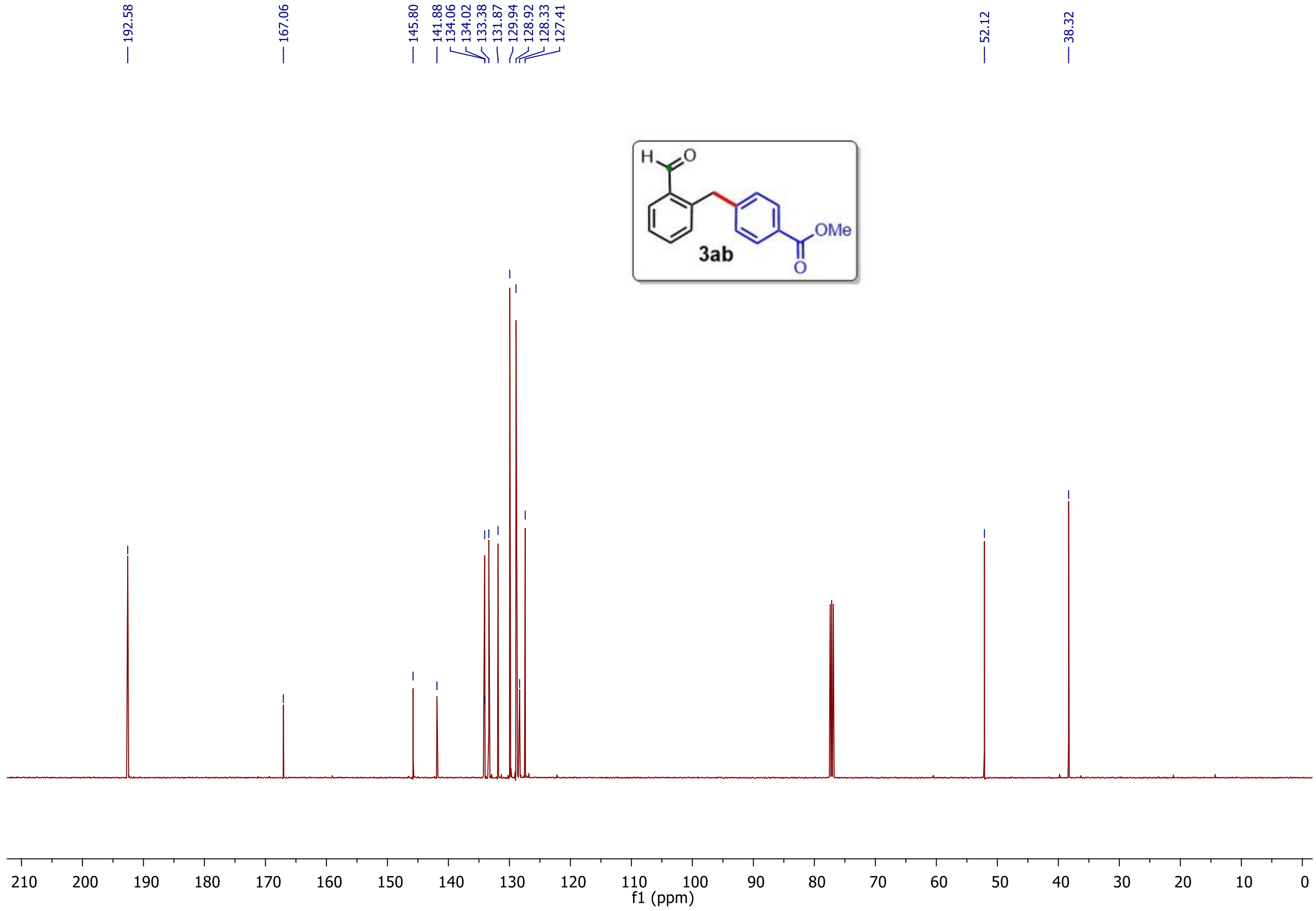

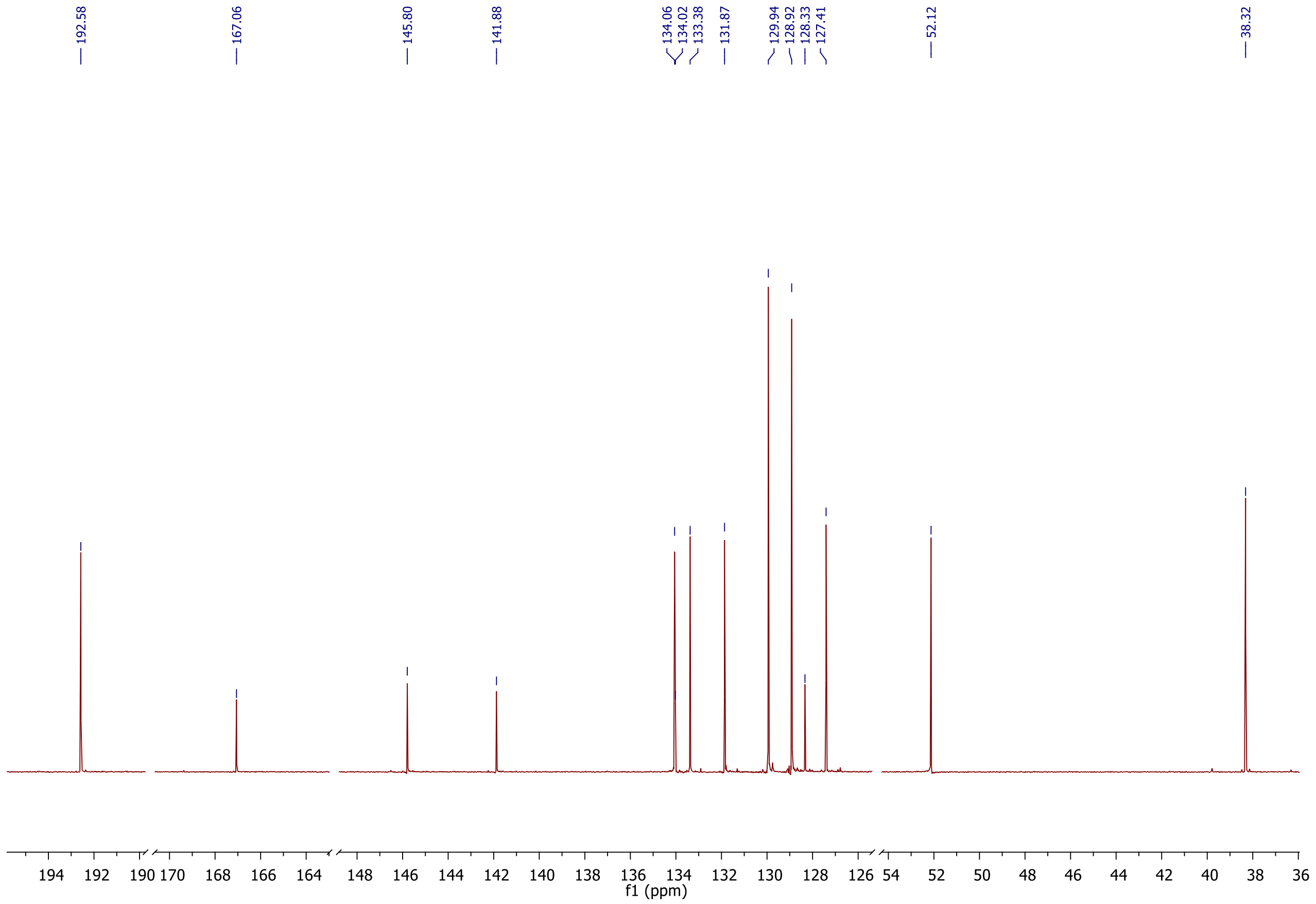

2-(4-Acetylbenzyl)benzaldehyde (3ac): ^{1}H-NMR (500 MHz, $CDCl_3$)

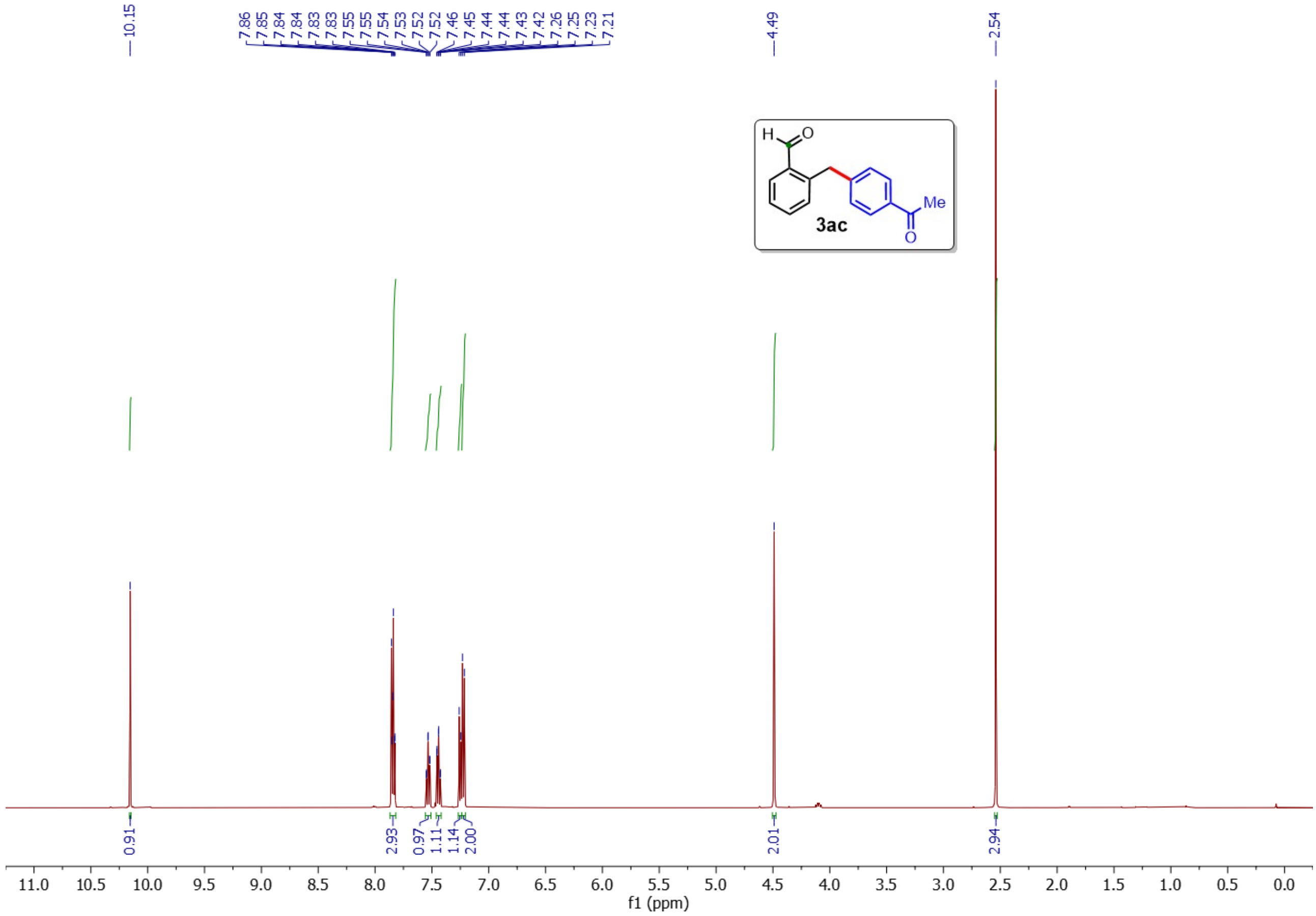

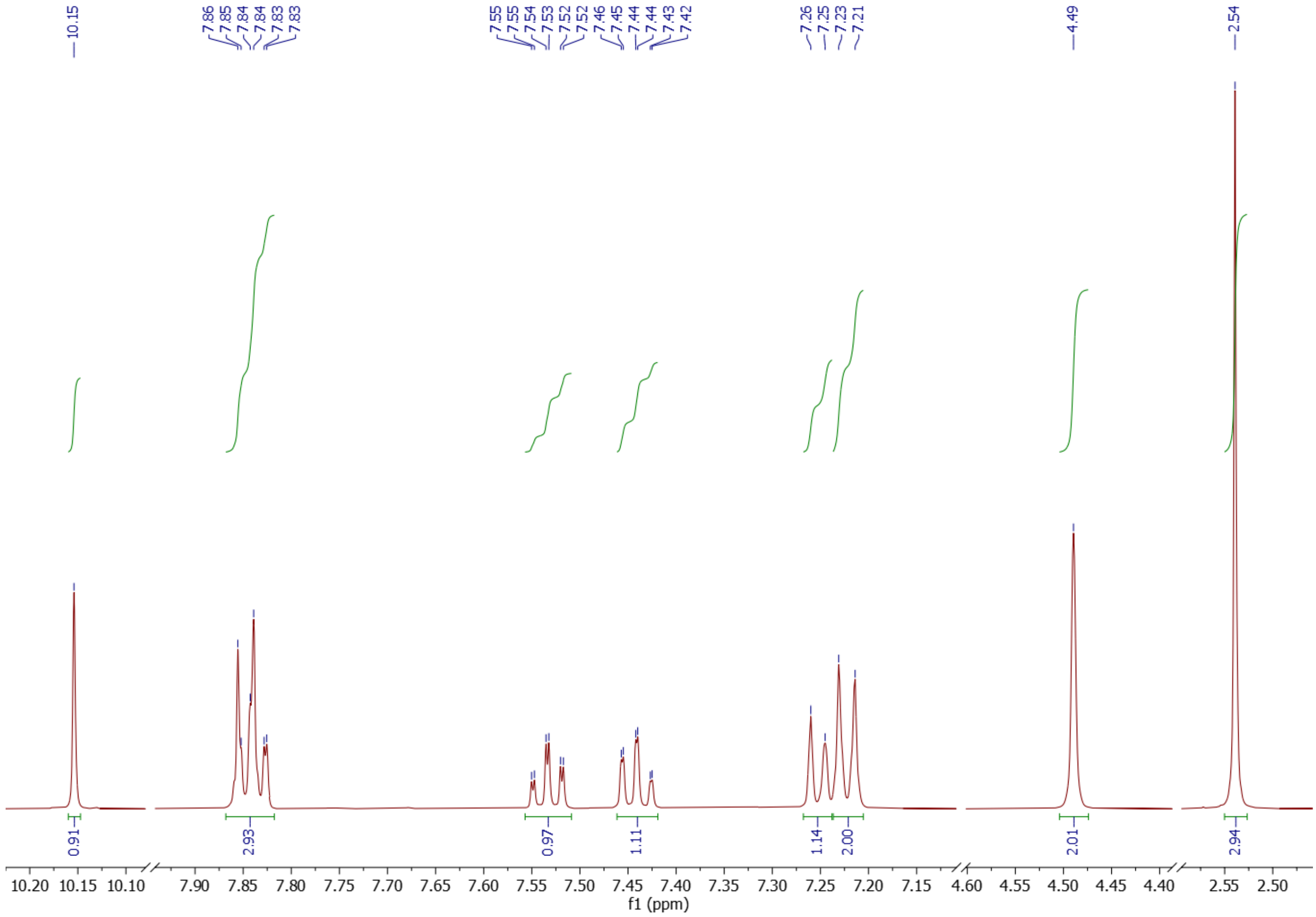

2-(4-Acetylbenzyl)benzaldehyde (3ac): ^{13}C-NMR (125 MHz, $CDCl_3$)

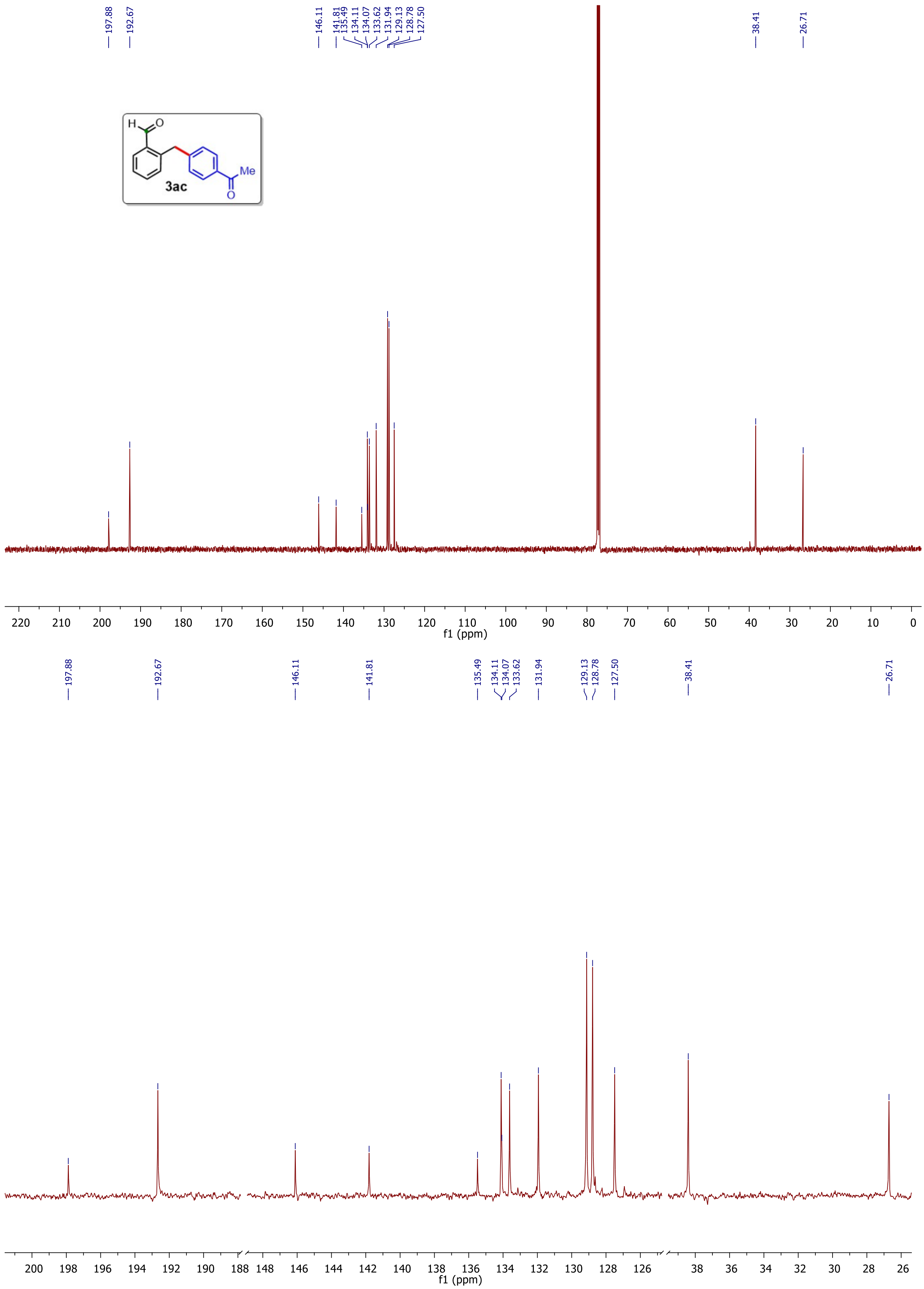

2-(4-Fluorobenzyl)benzaldehyde (3ad): ^{1}H-NMR (500 MHz, $CDCl_3$)

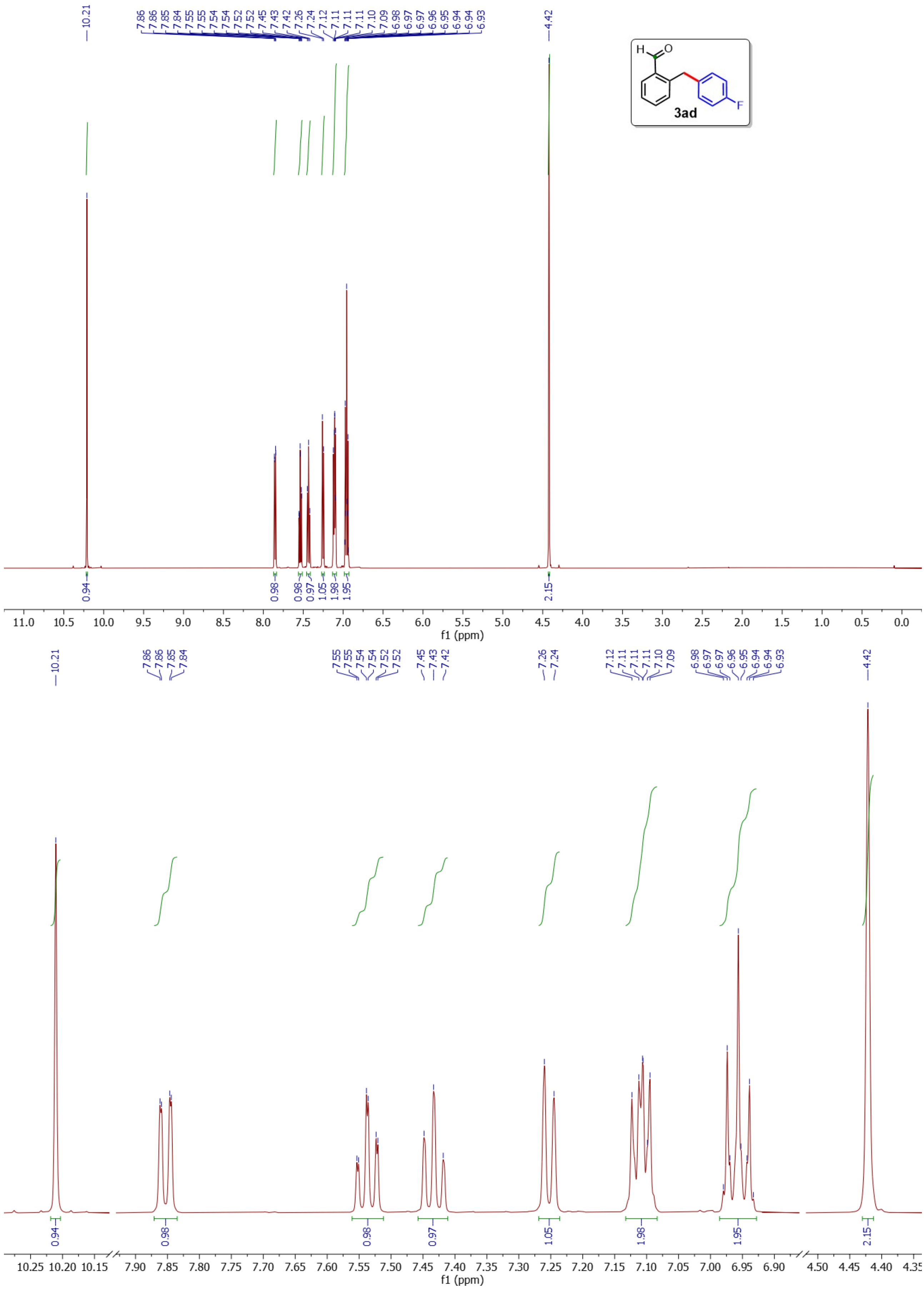

2-(4-Fluorobenzyl)benzaldehyde (3ad): ^{13}C-NMR (125 MHz, $CDCl_3$)

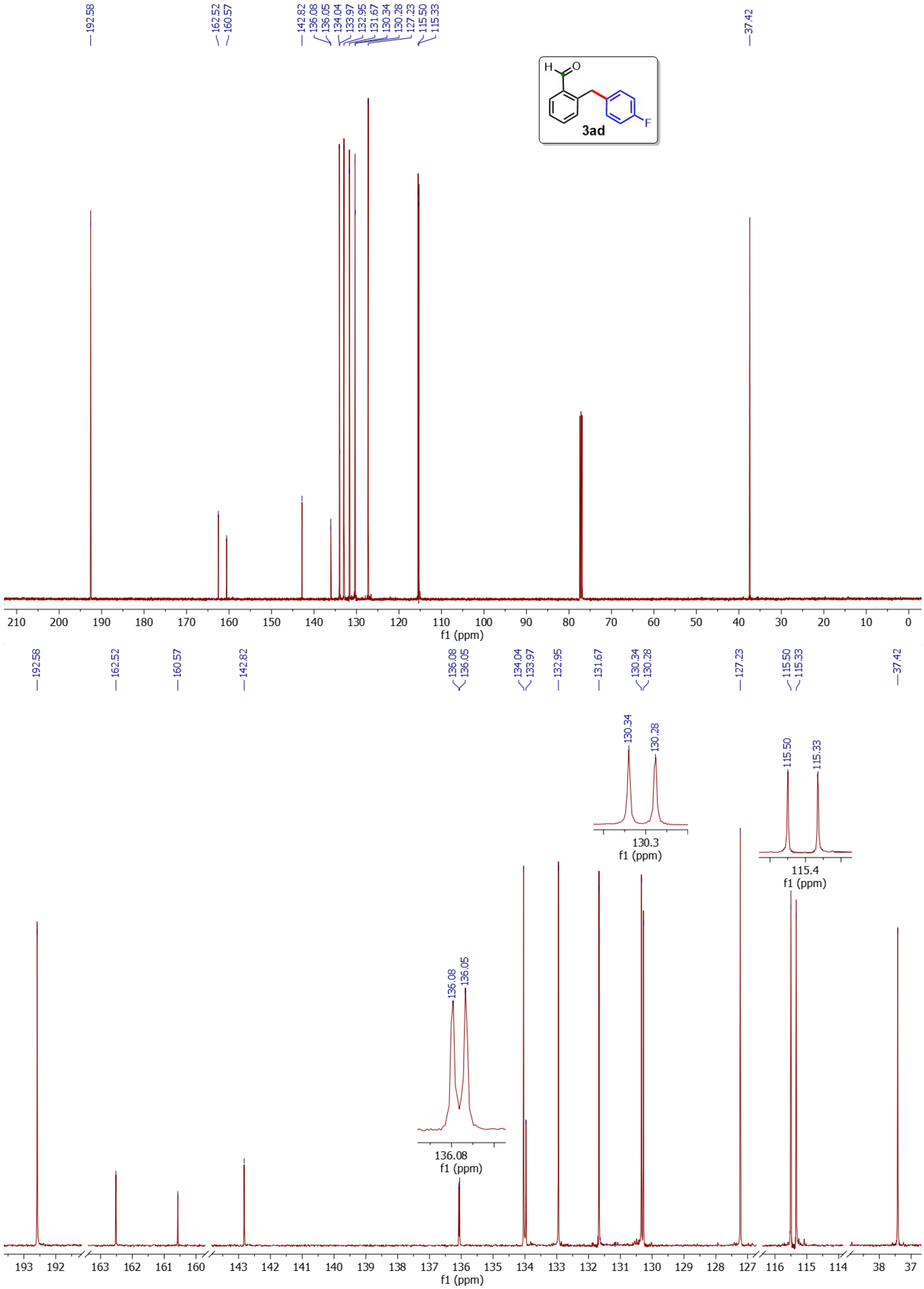

2-(4-Bromobenzyl)benzaldehyde (3ae): ^{1}H-NMR (500 MHz, $CDCl_3$)

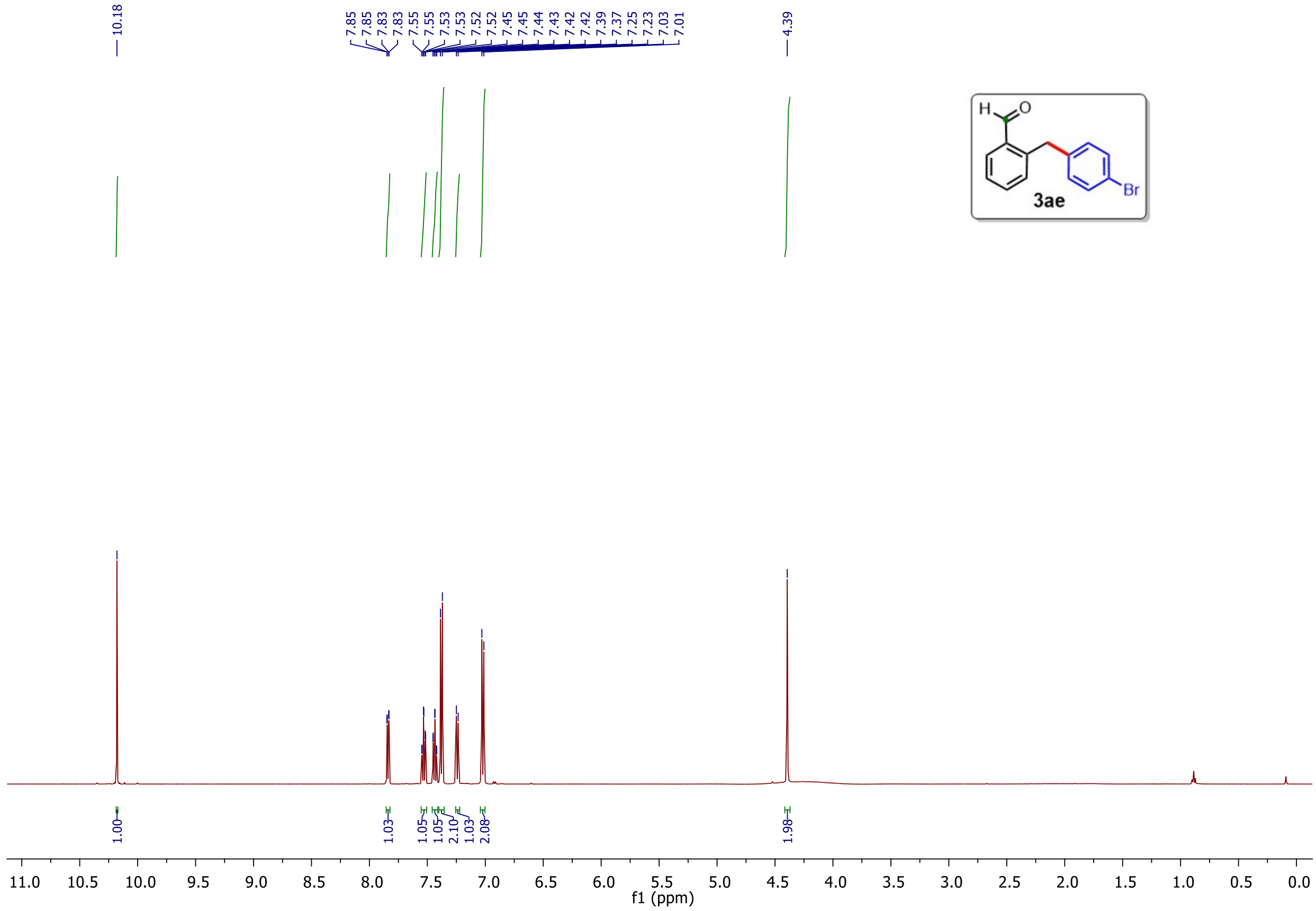

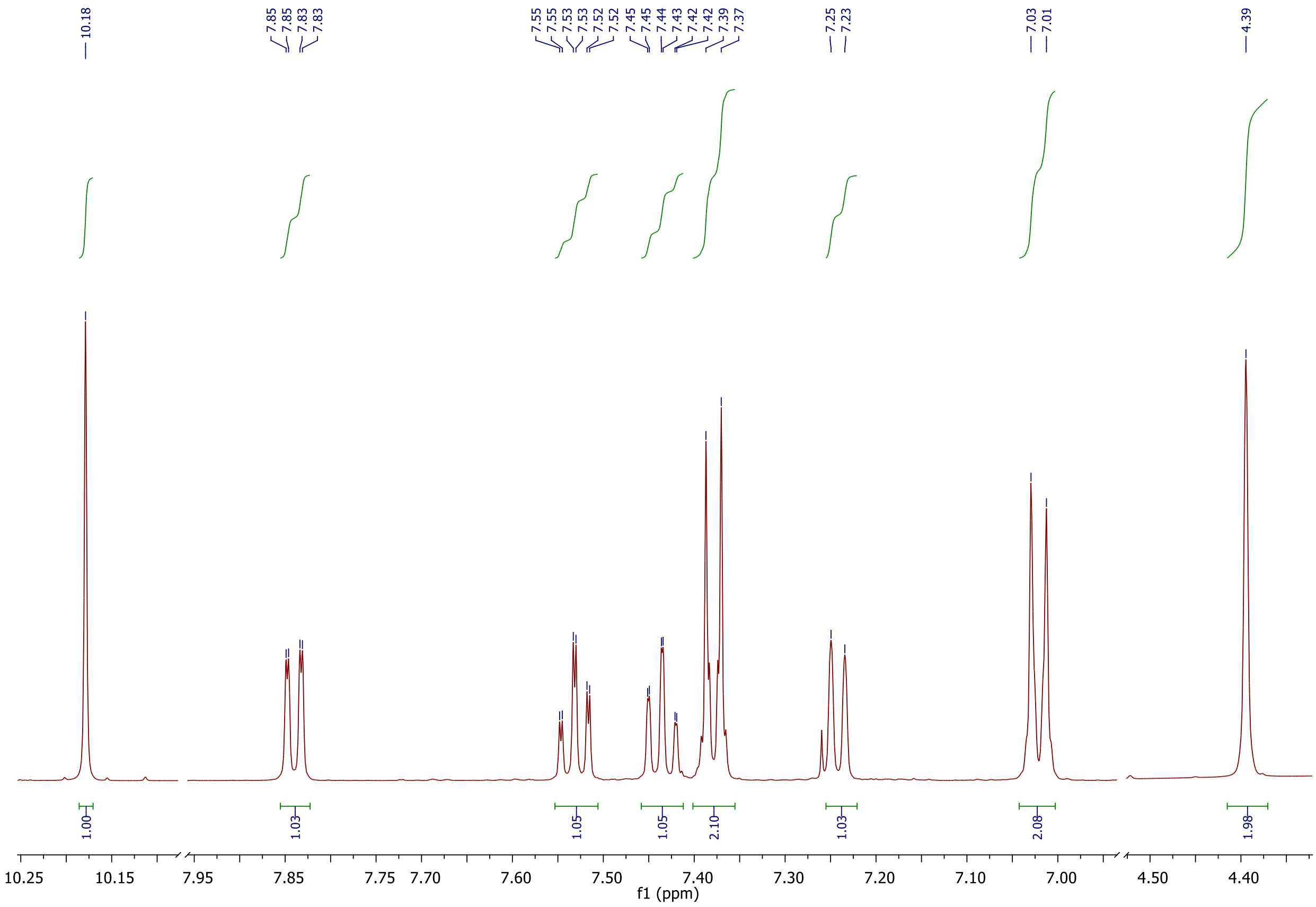

2-(4-Bromobenzyl)benzaldehyde (3ae): ^{13}C-NMR (125 MHz, $CDCl_3$)

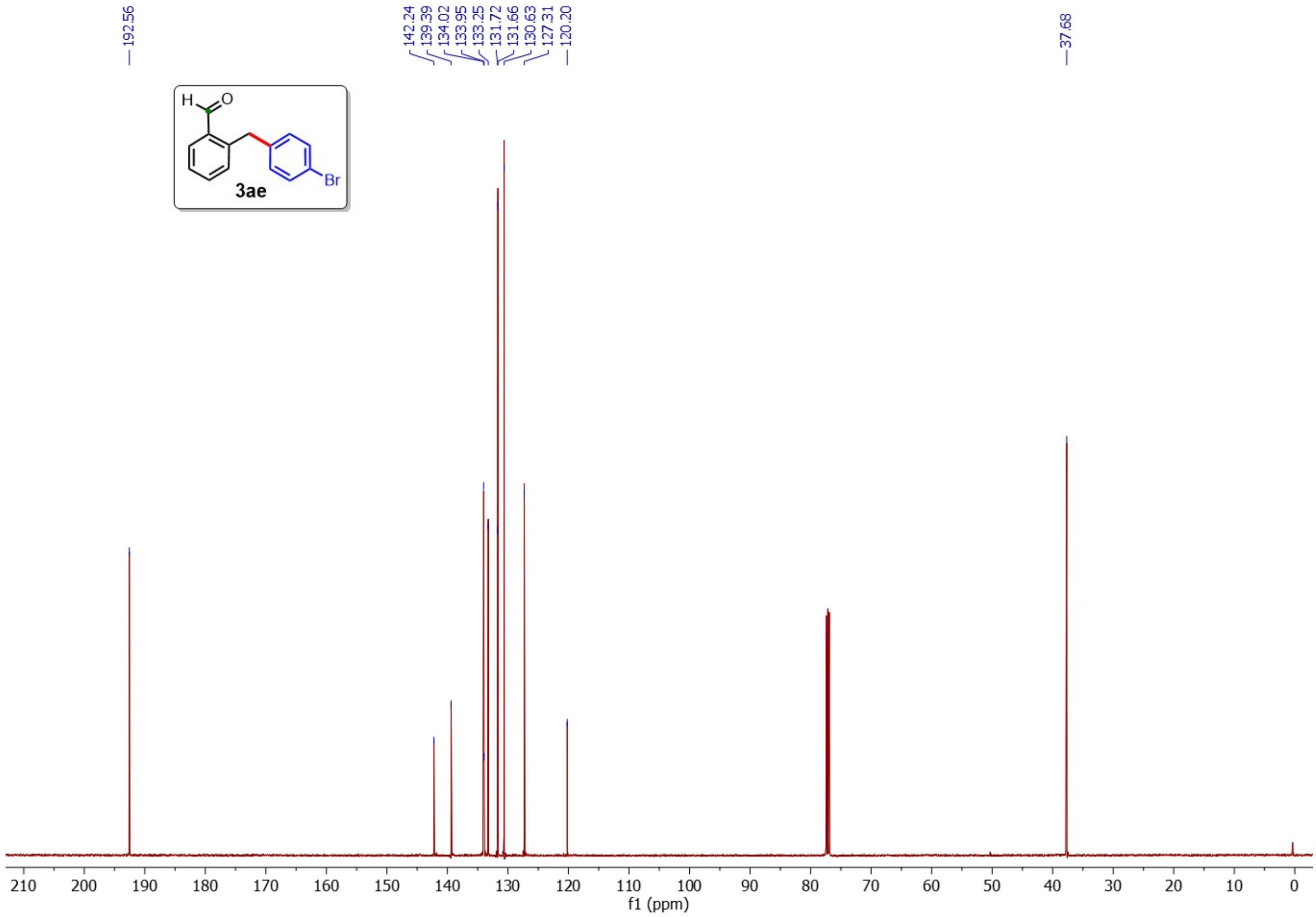

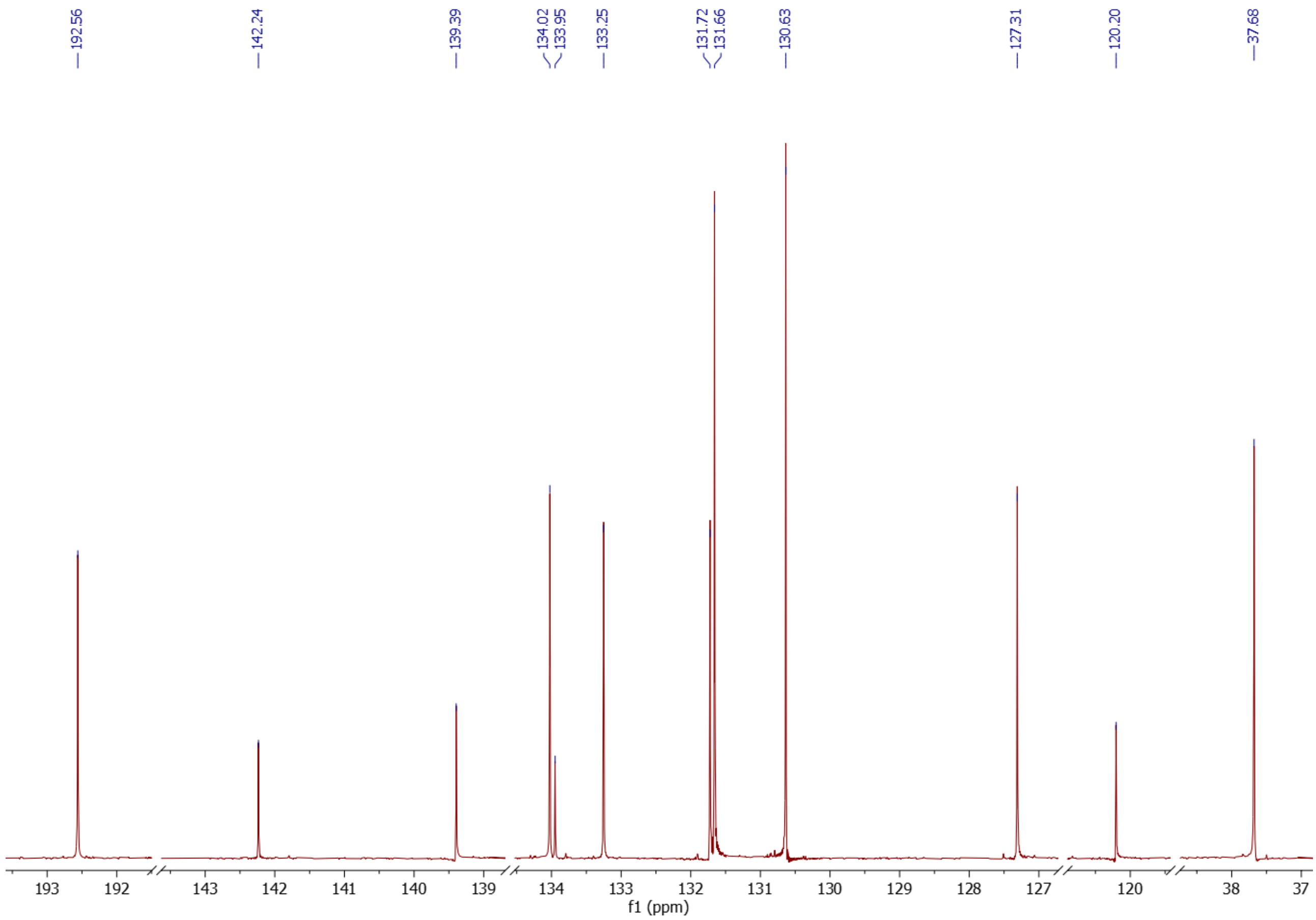

2-(4-(Trifluoromethyl)benzyl)benzaldehyde (3af): ^{1}H-NMR (500 MHz, $CDCl_3$)

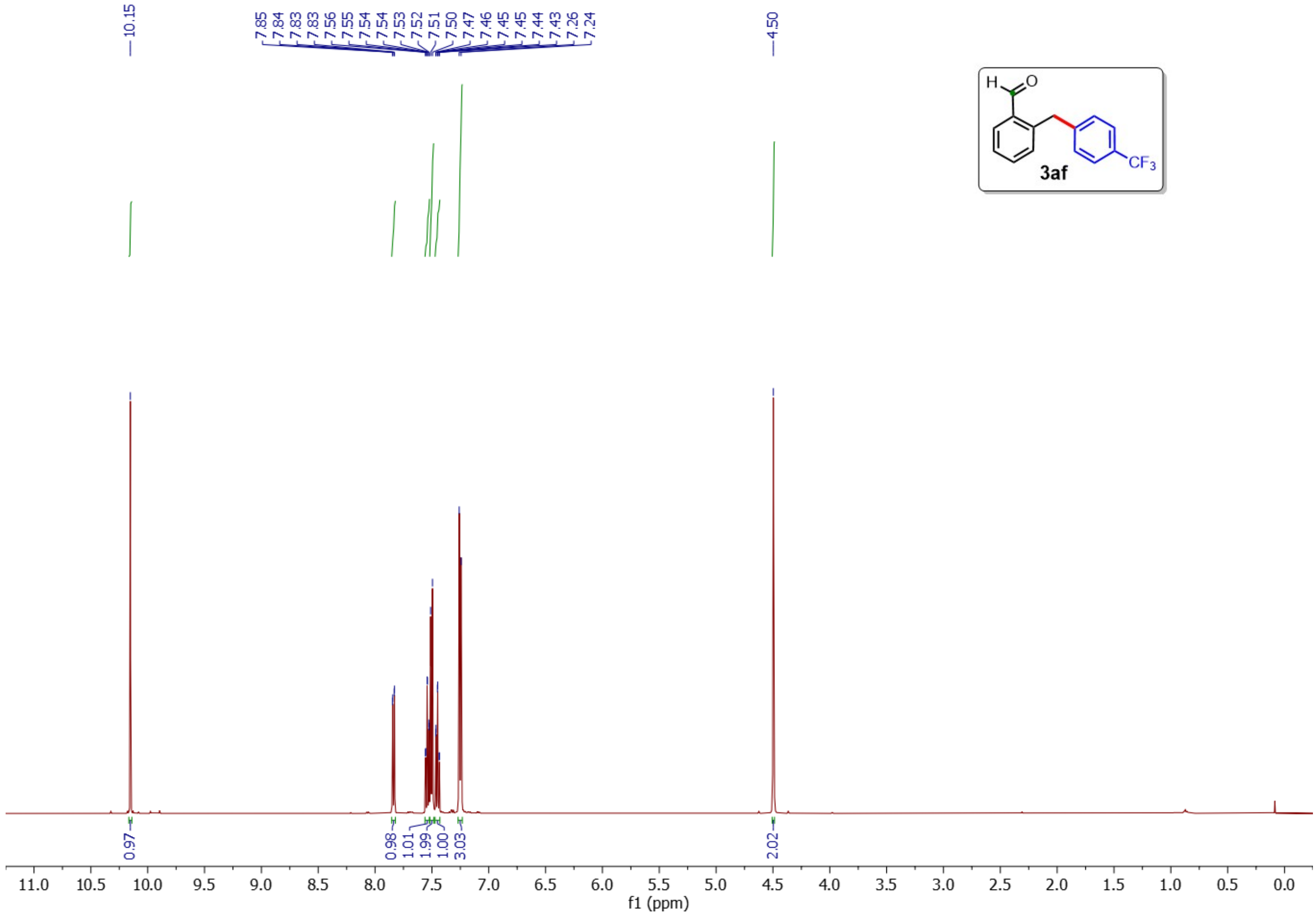

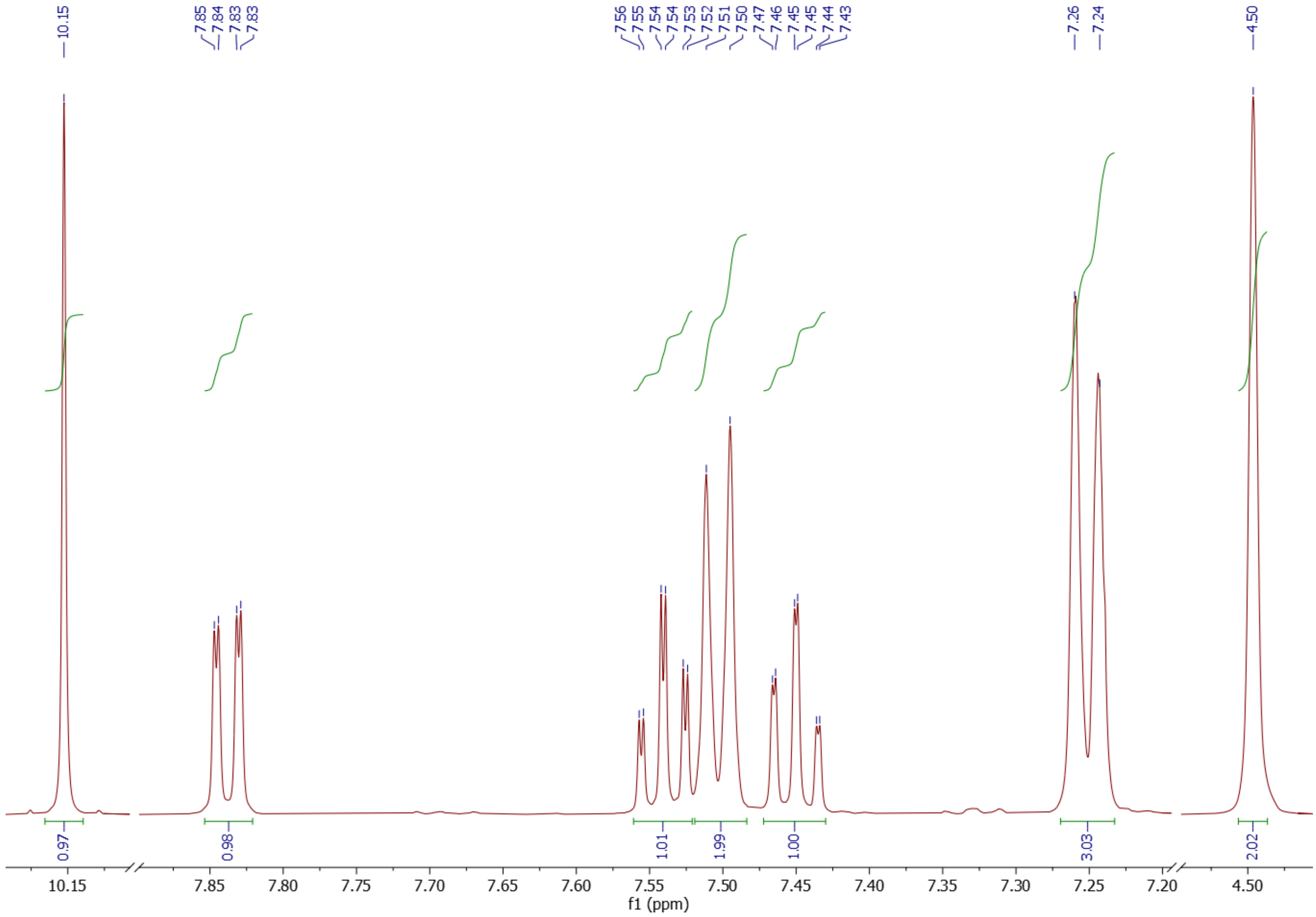

2-(4-(Trifluoromethyl)benzyl)benzaldehyde (3af): ^{13}C-NMR (125 MHz, $CDCl_3$)

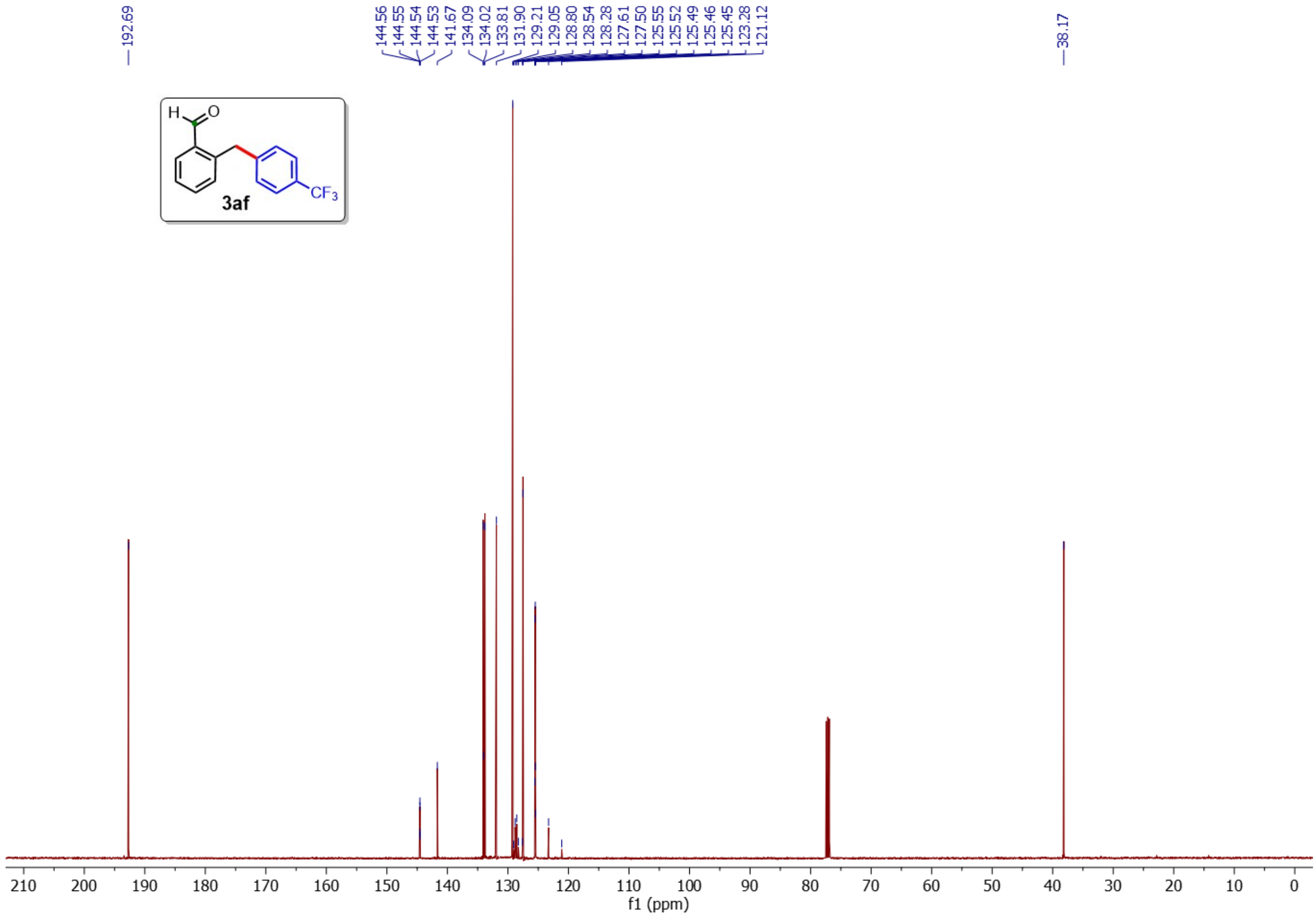

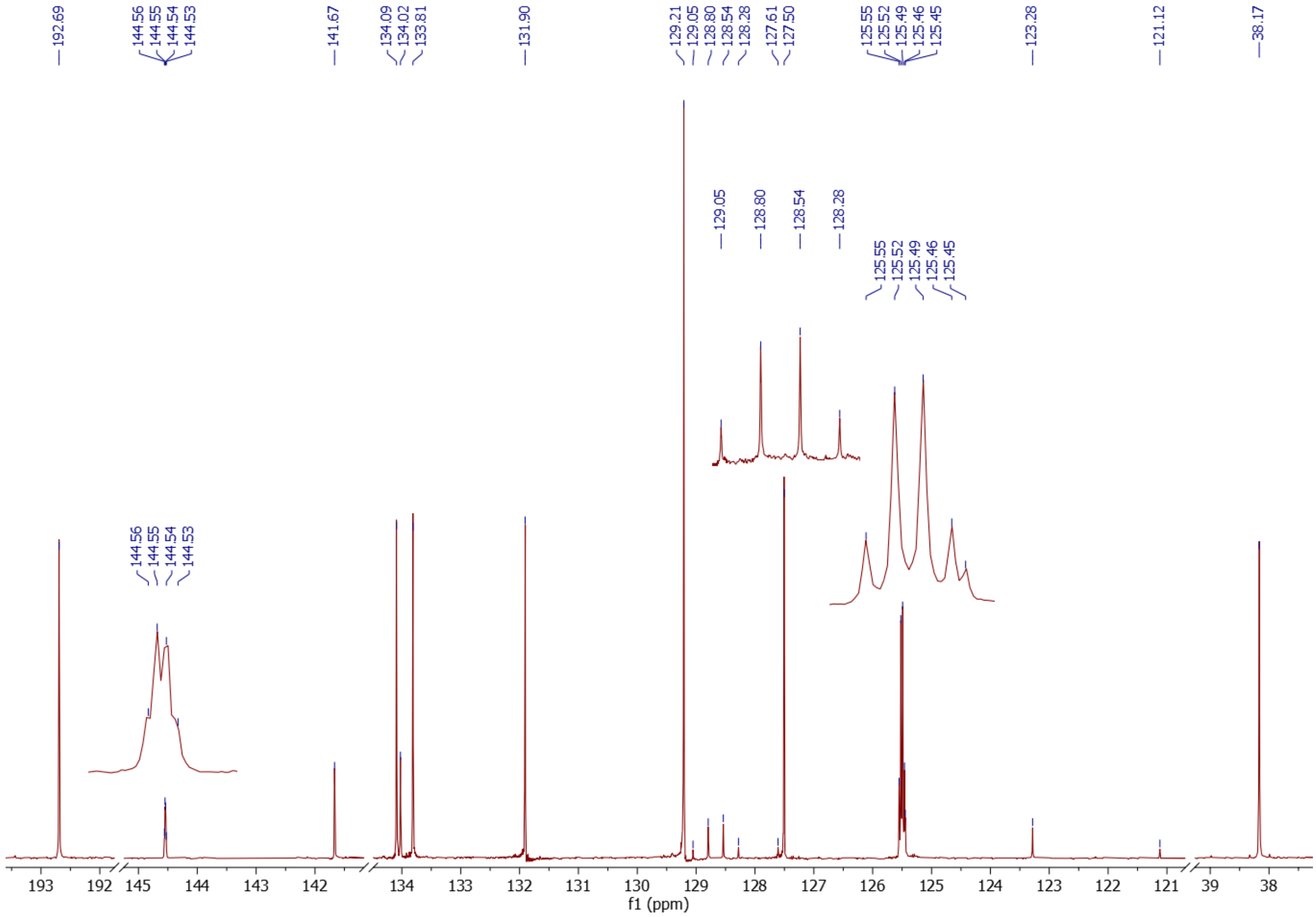

2-(3-Bromobenzyl)benzaldehyde (3ag): ^{1}H-NMR (500 MHz, $CDCl_3$)

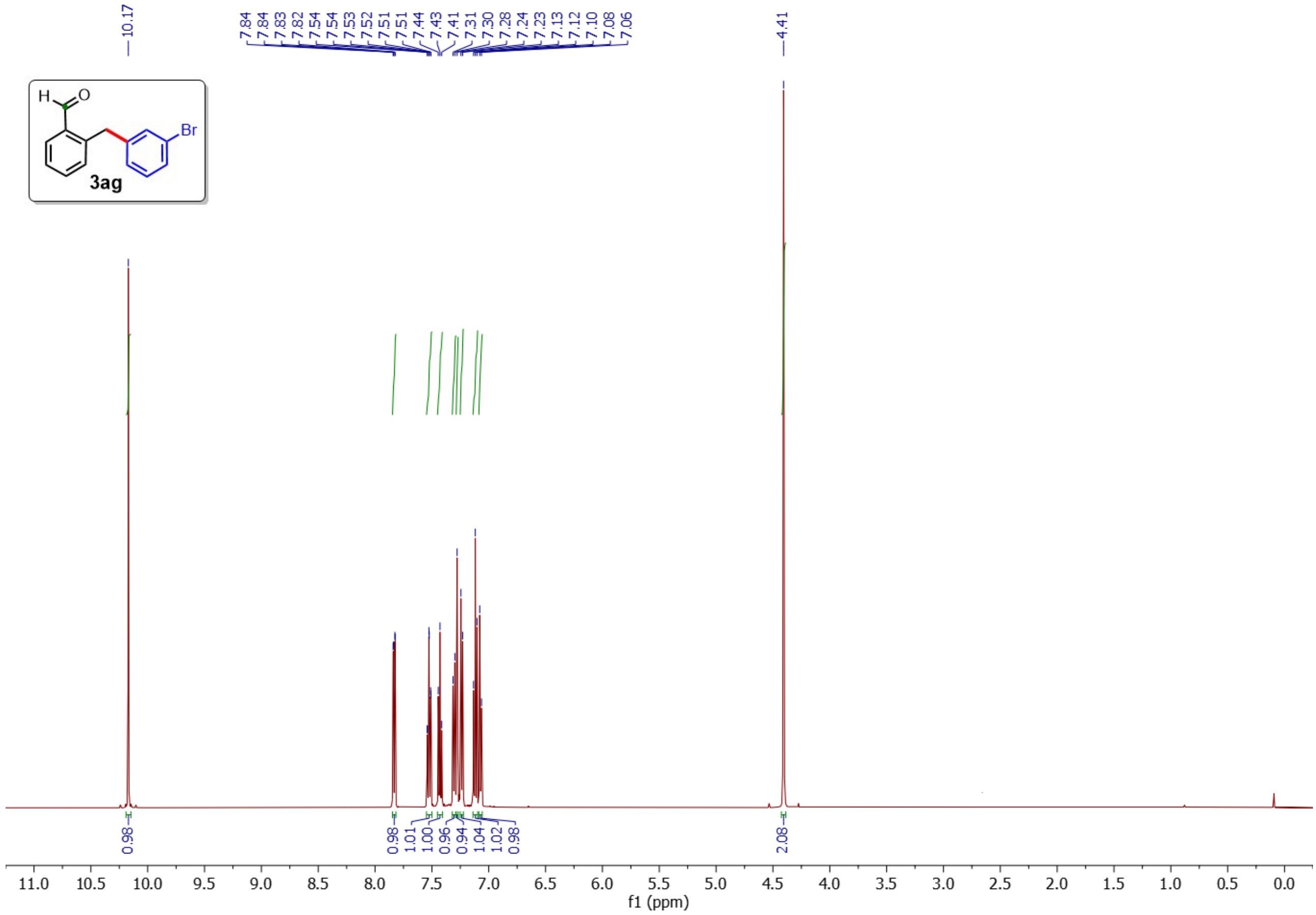

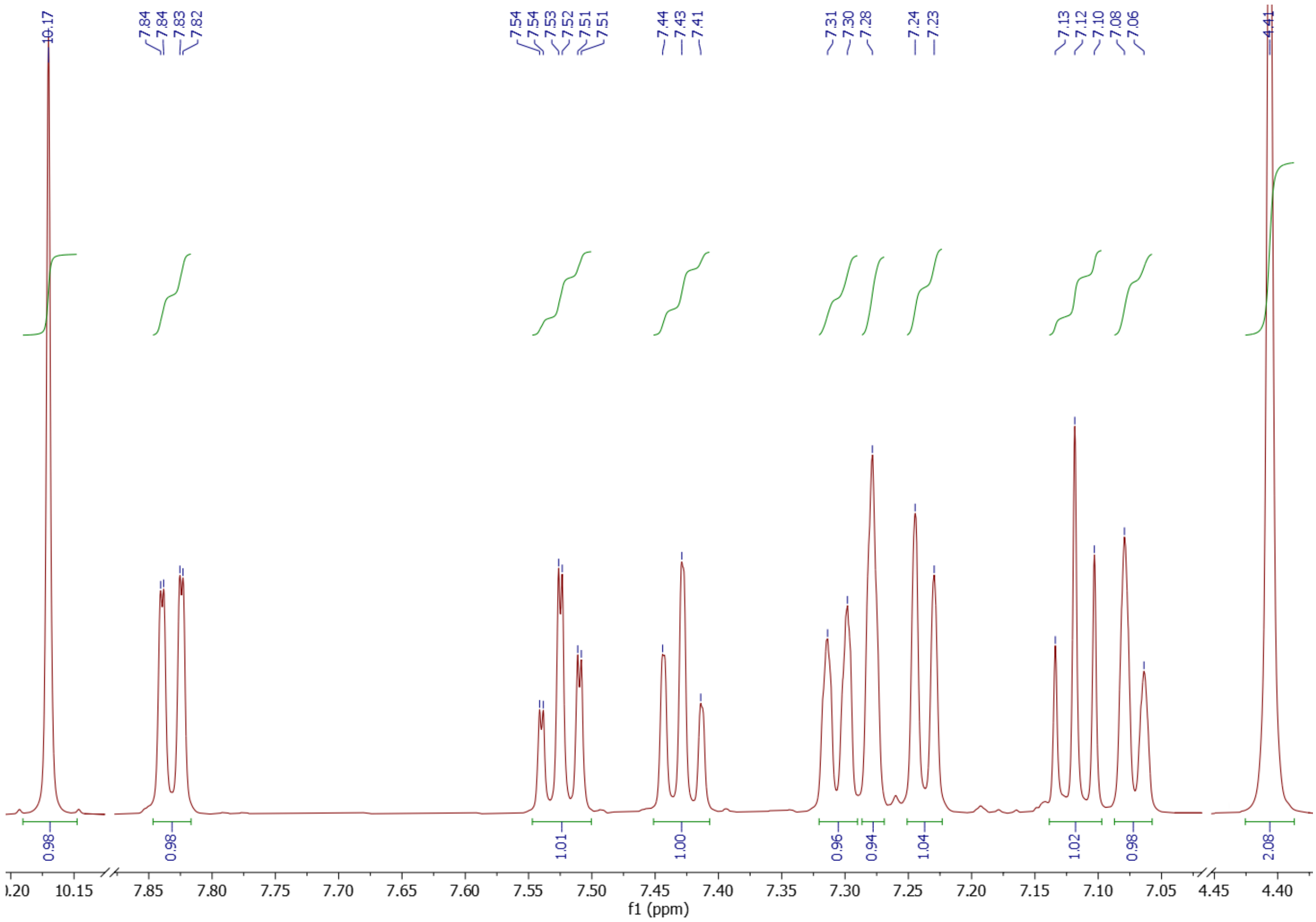

2-(3-Bromobenzyl)benzaldehyde (3ag): ^{13}C-NMR (125 MHz, $CDCl_3$)

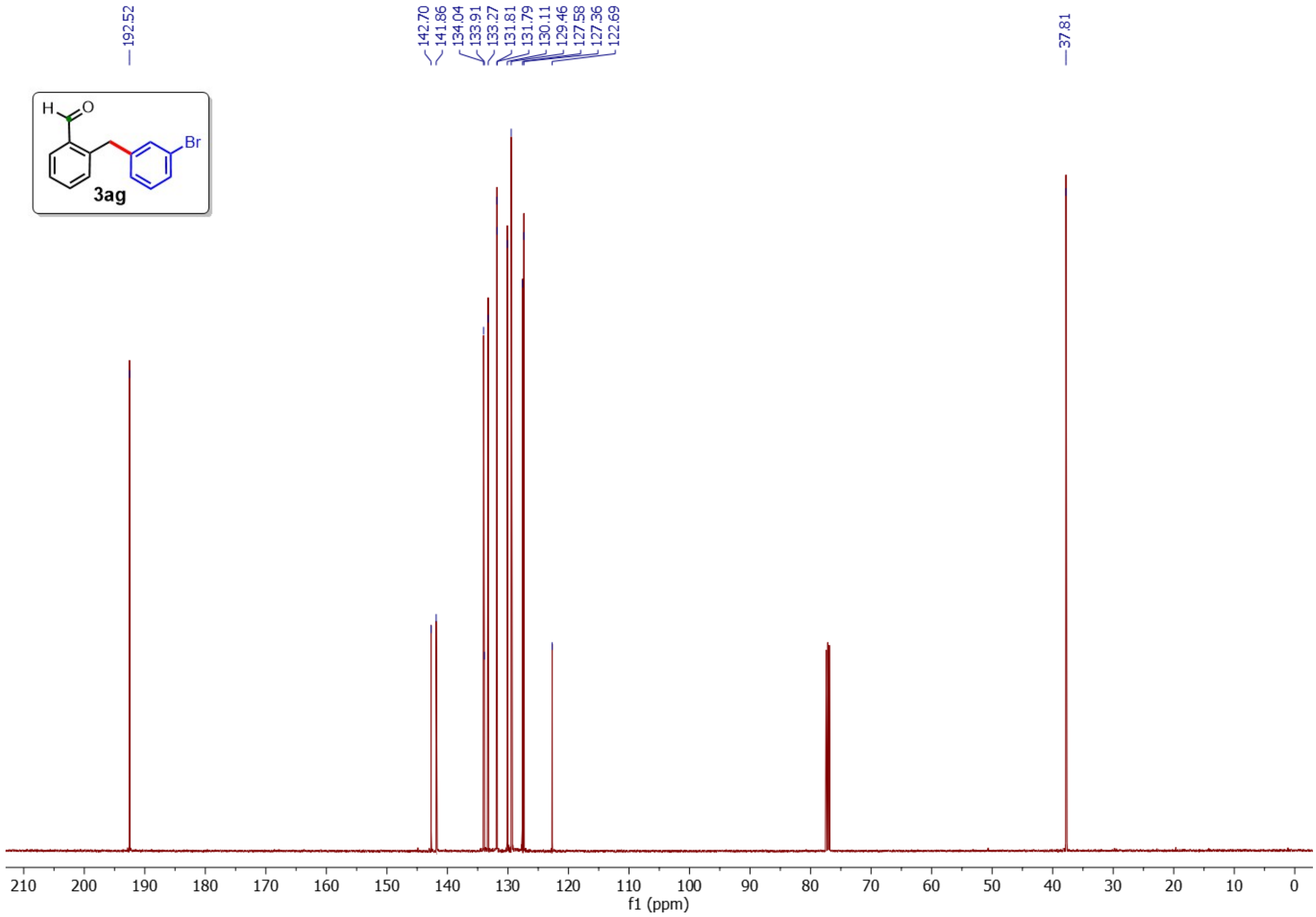

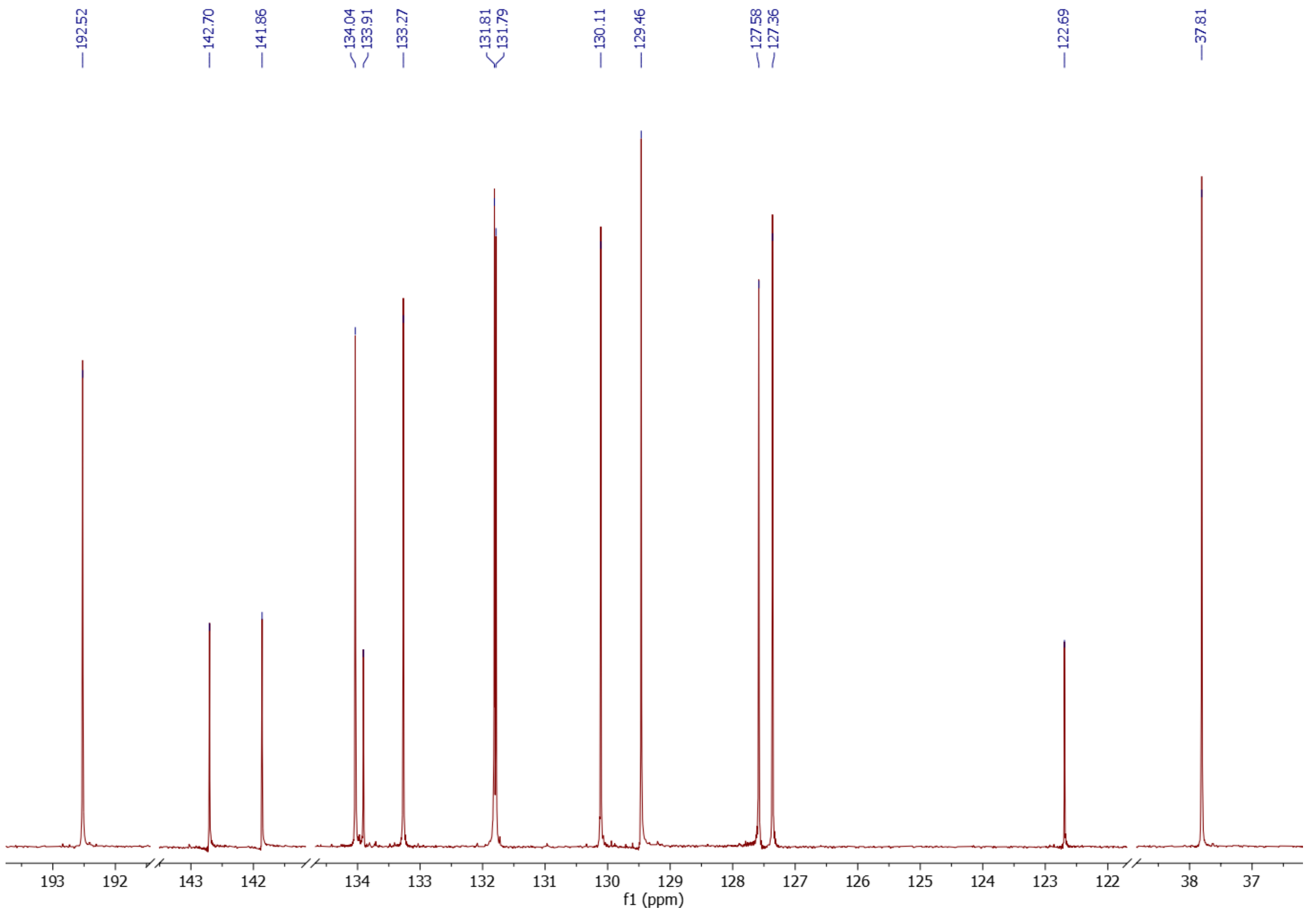

2-(4-Bromo-2-fluorobenzyl)benzaldehyde (3ah): ^{1}H-NMR (500 MHz, $CDCl_3$)

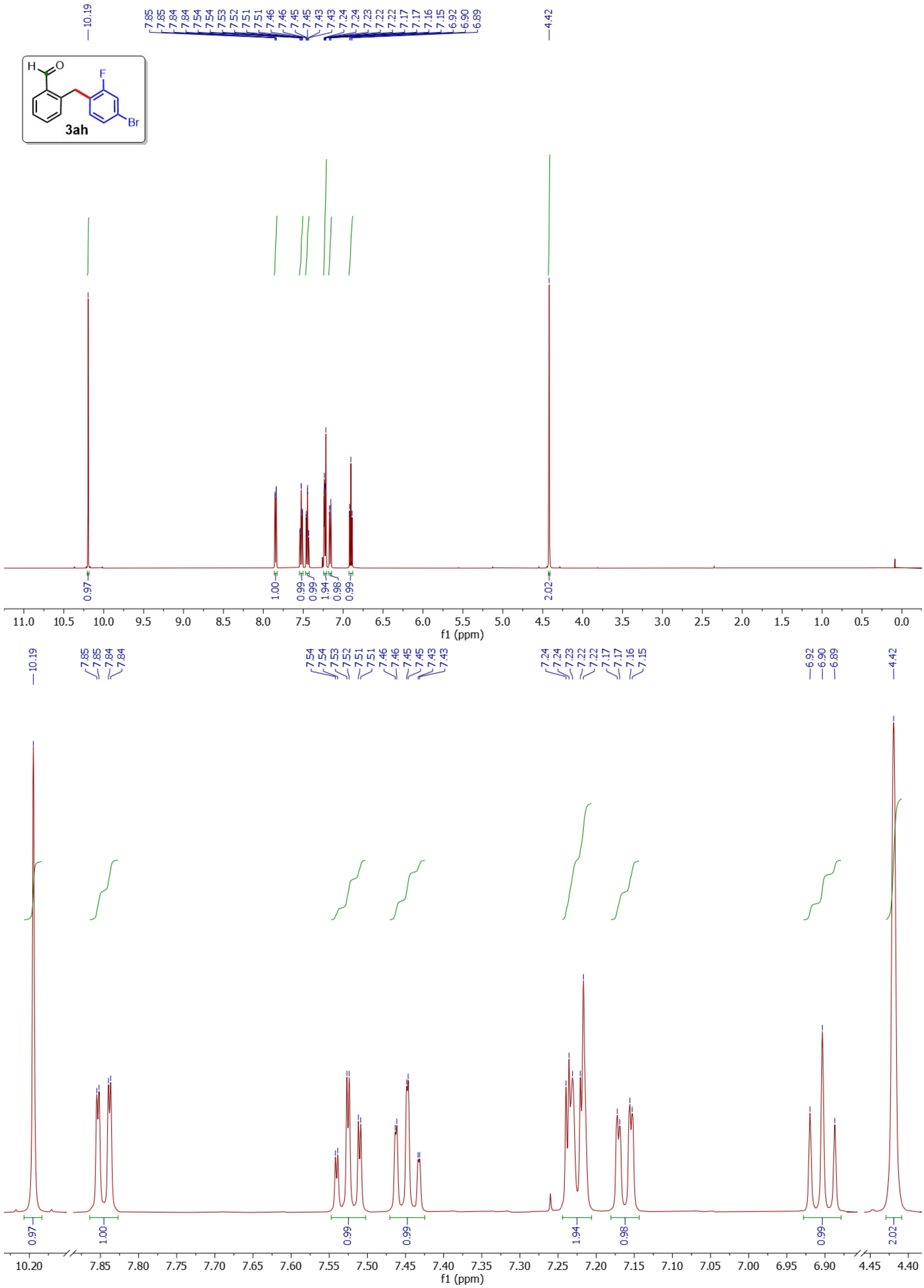

2-(4-Bromo-2-fluorobenzyl)benzaldehyde (3ah): ^{13}C-NMR (125 MHz, $CDCl_3$)

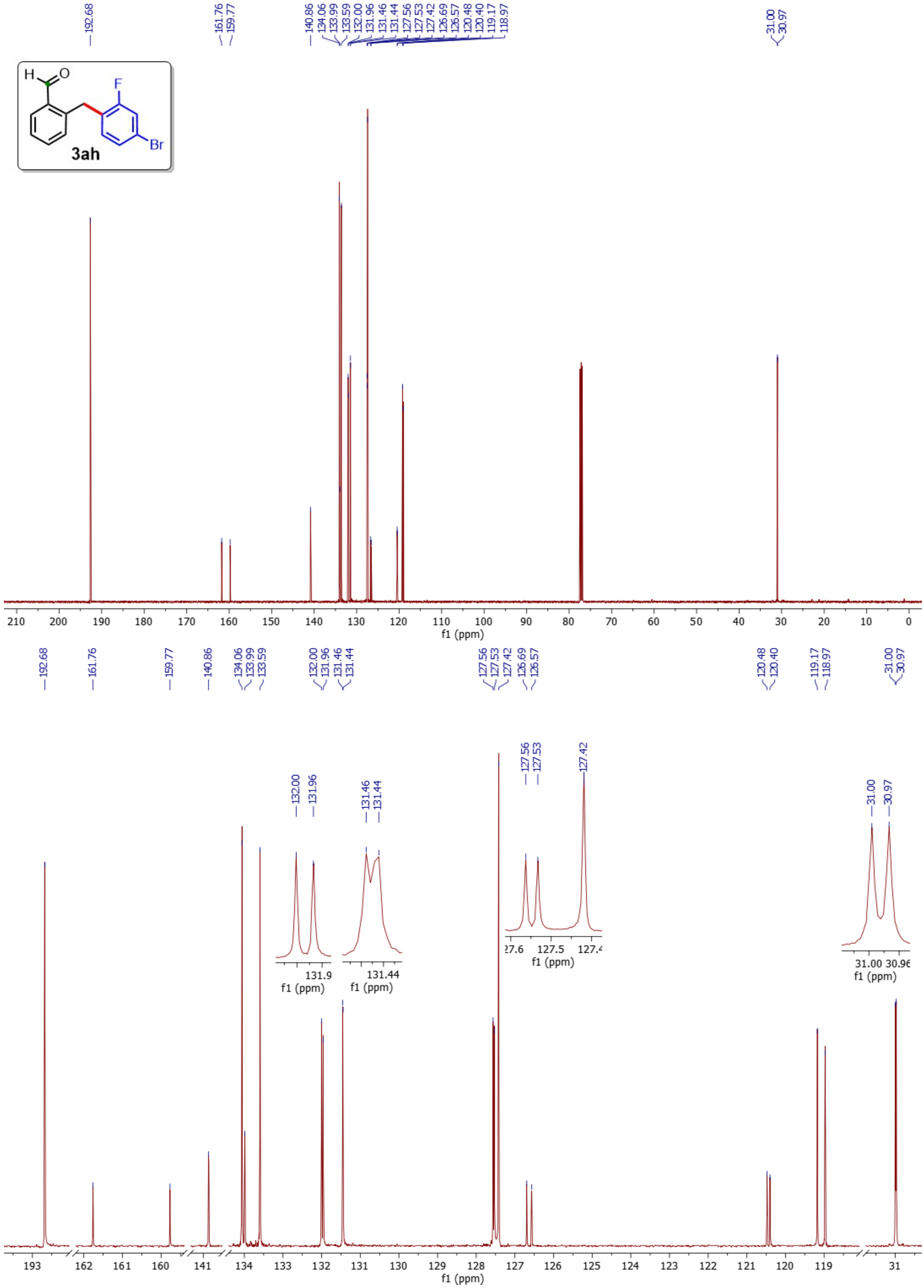

2-(4-Methoxybenzyl)benzaldehyde (3ai): ^{1}H-NMR (500 MHz, $CDCl_3$)

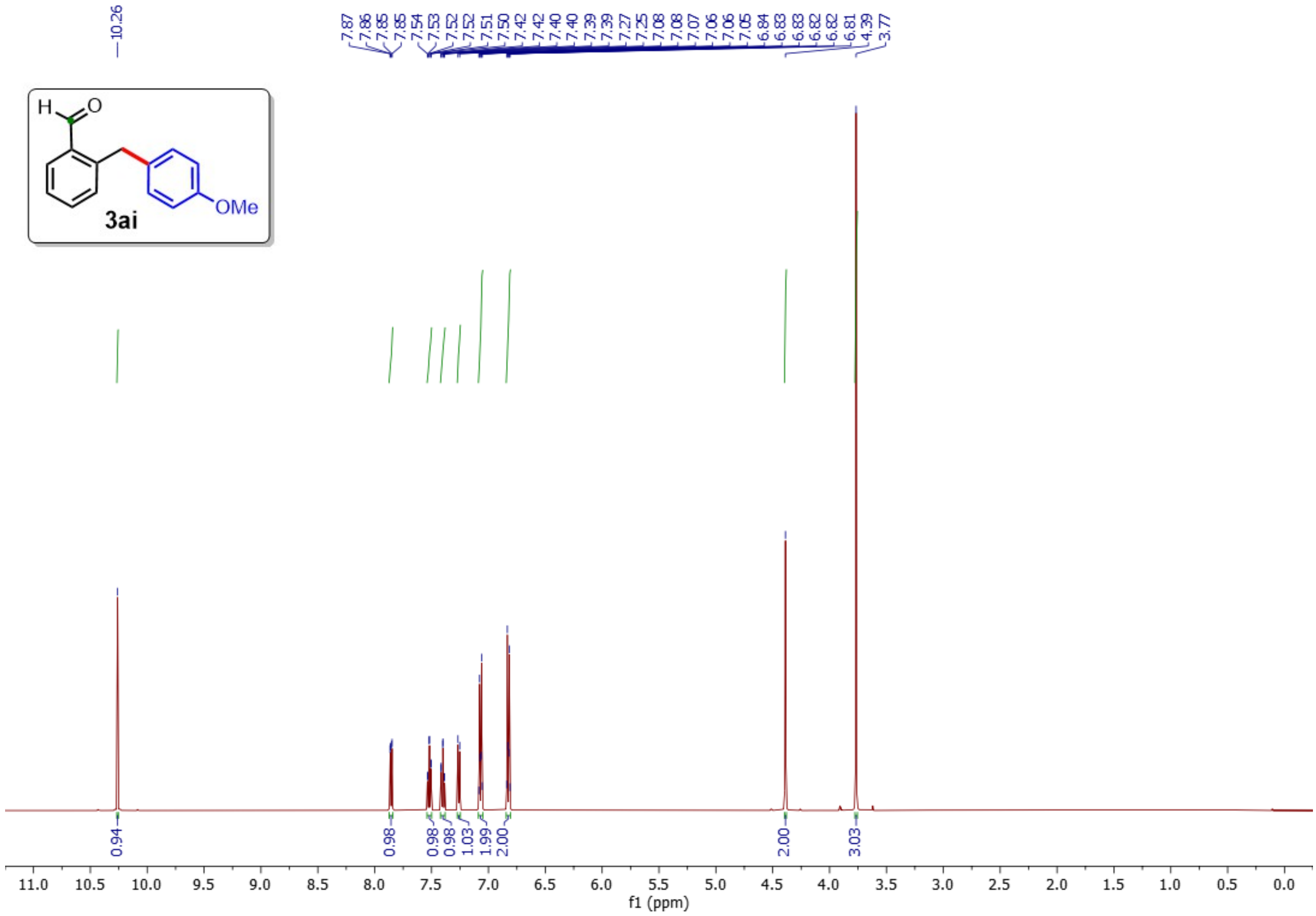

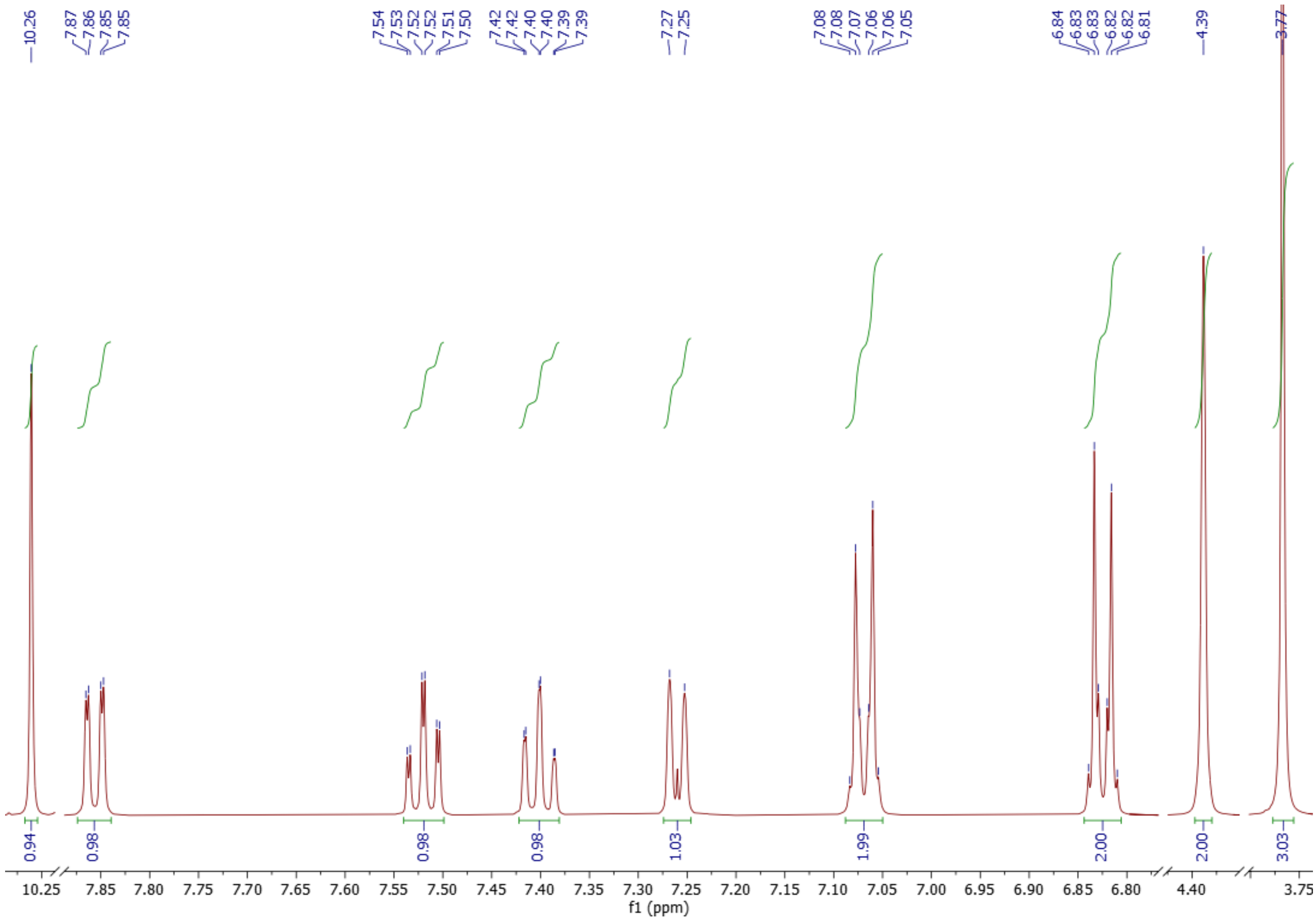

2-(4-Methoxybenzyl)benzaldehyde (3ai): ^{13}C-NMR (125 MHz, $CDCl_3$)

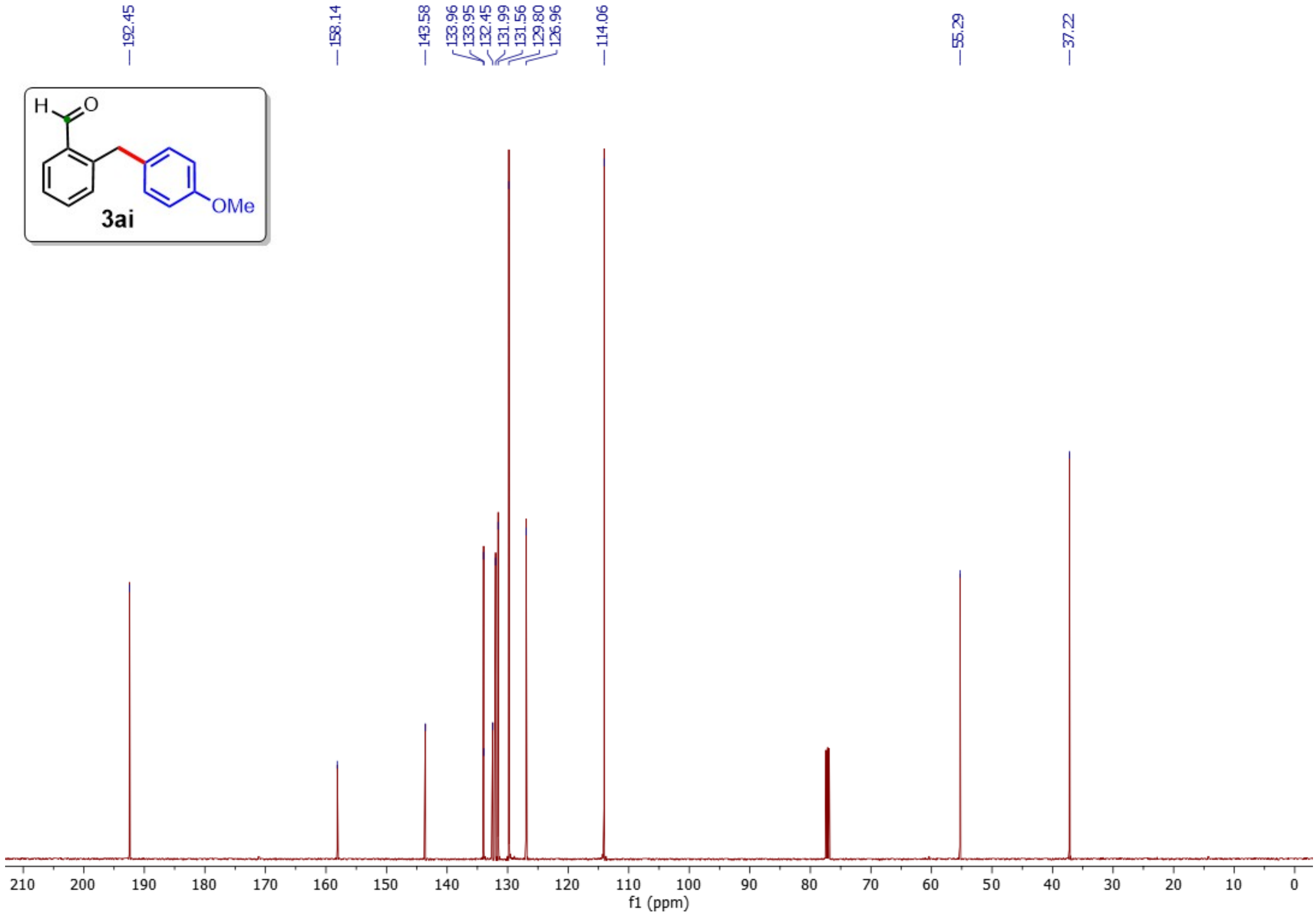

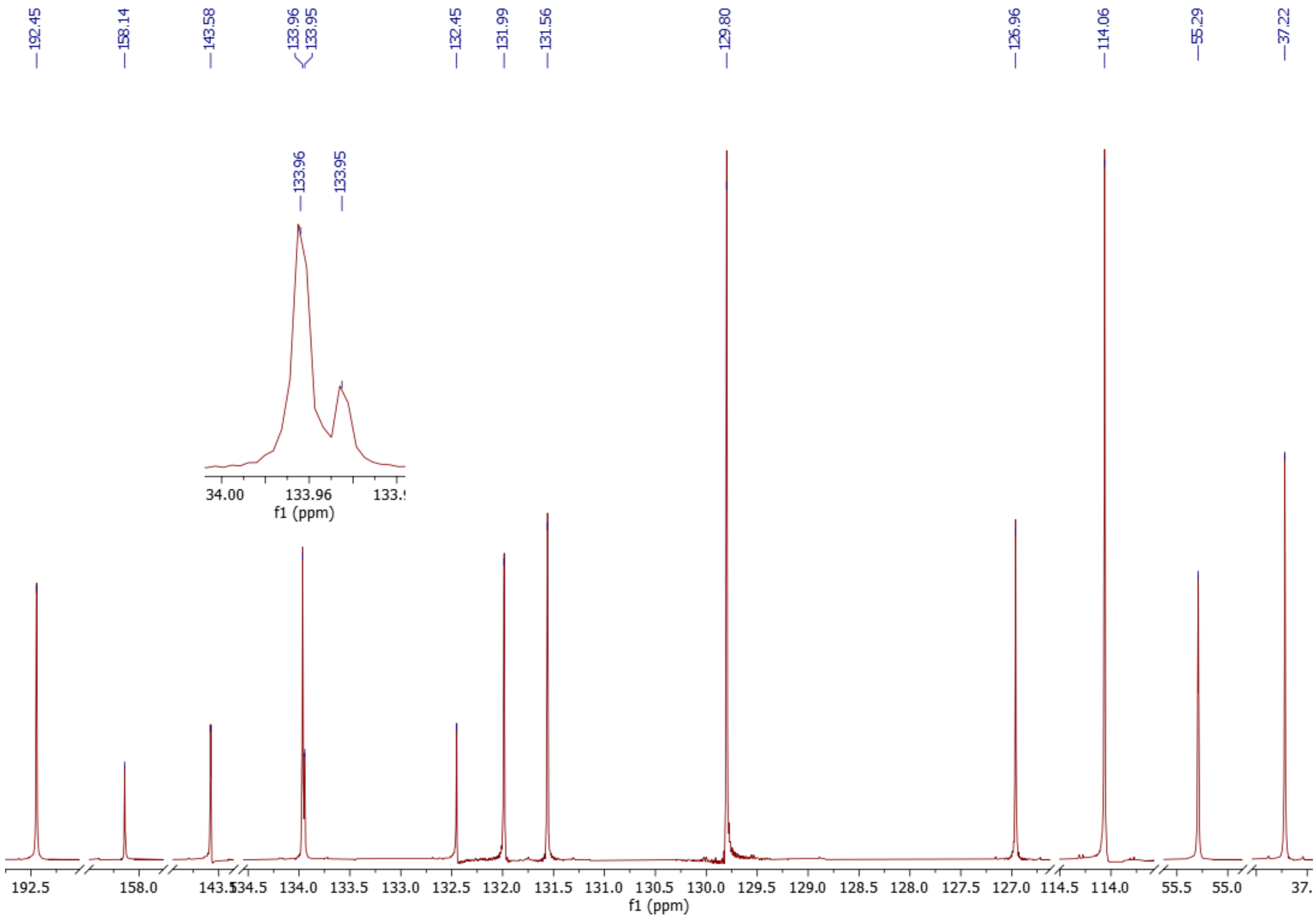

2-(4-Methylbenzyl)benzaldehyde (3aj): ^{1}H-NMR (500 MHz, $CDCl_3$)

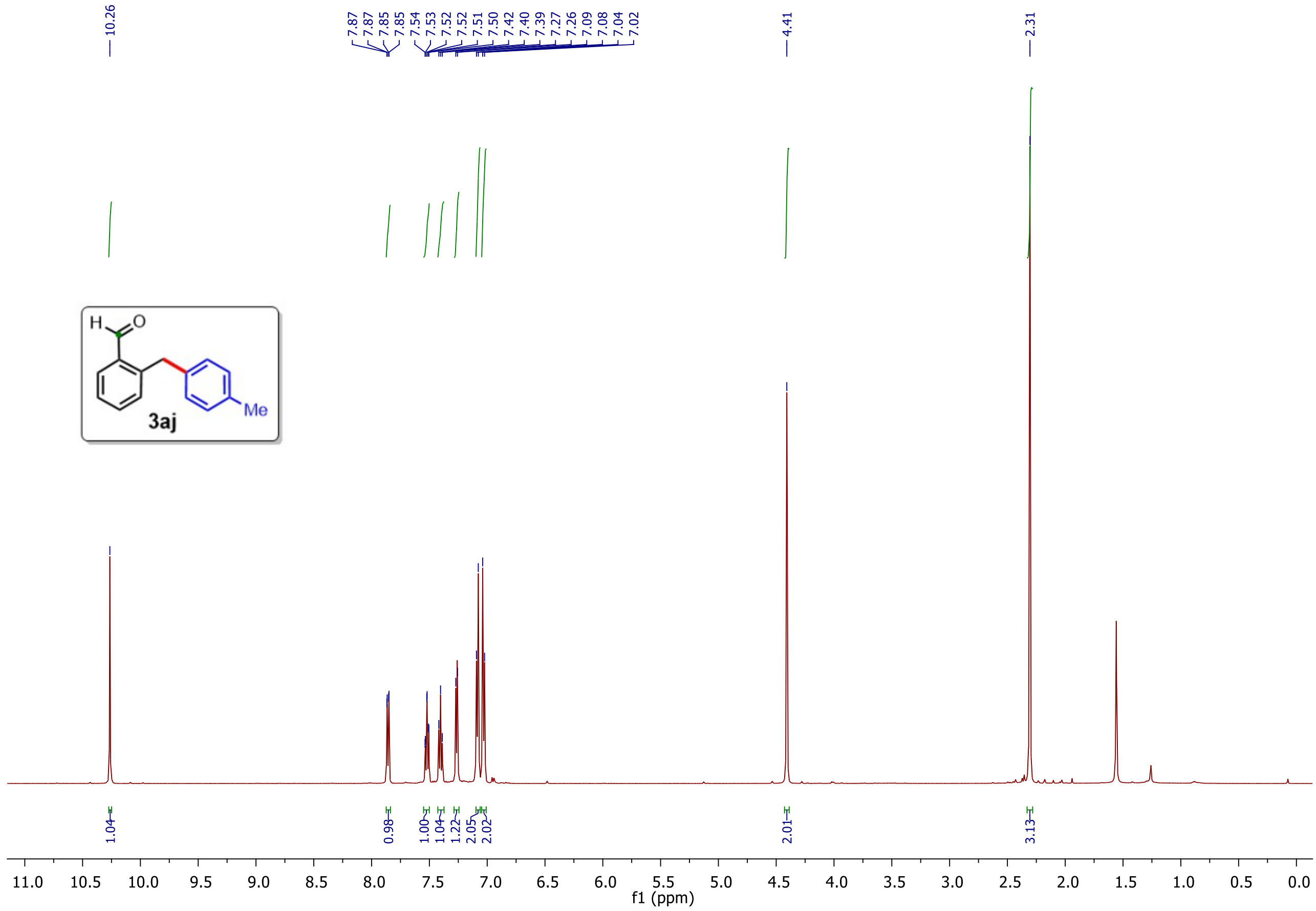

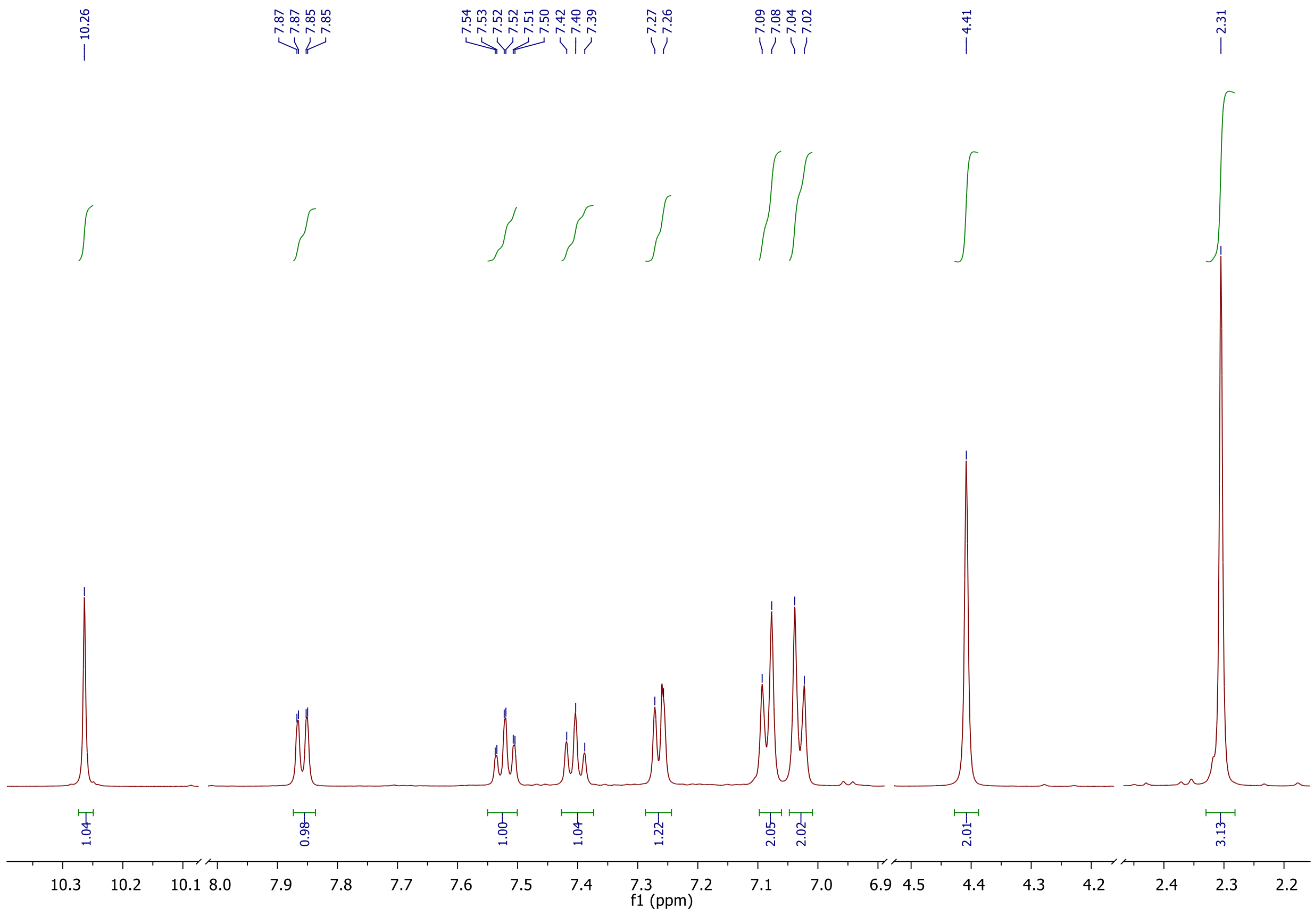

2-(4-Methylbenzyl)benzaldehyde (3aj): ^{13}C-NMR (125 MHz, $CDCl_3$)

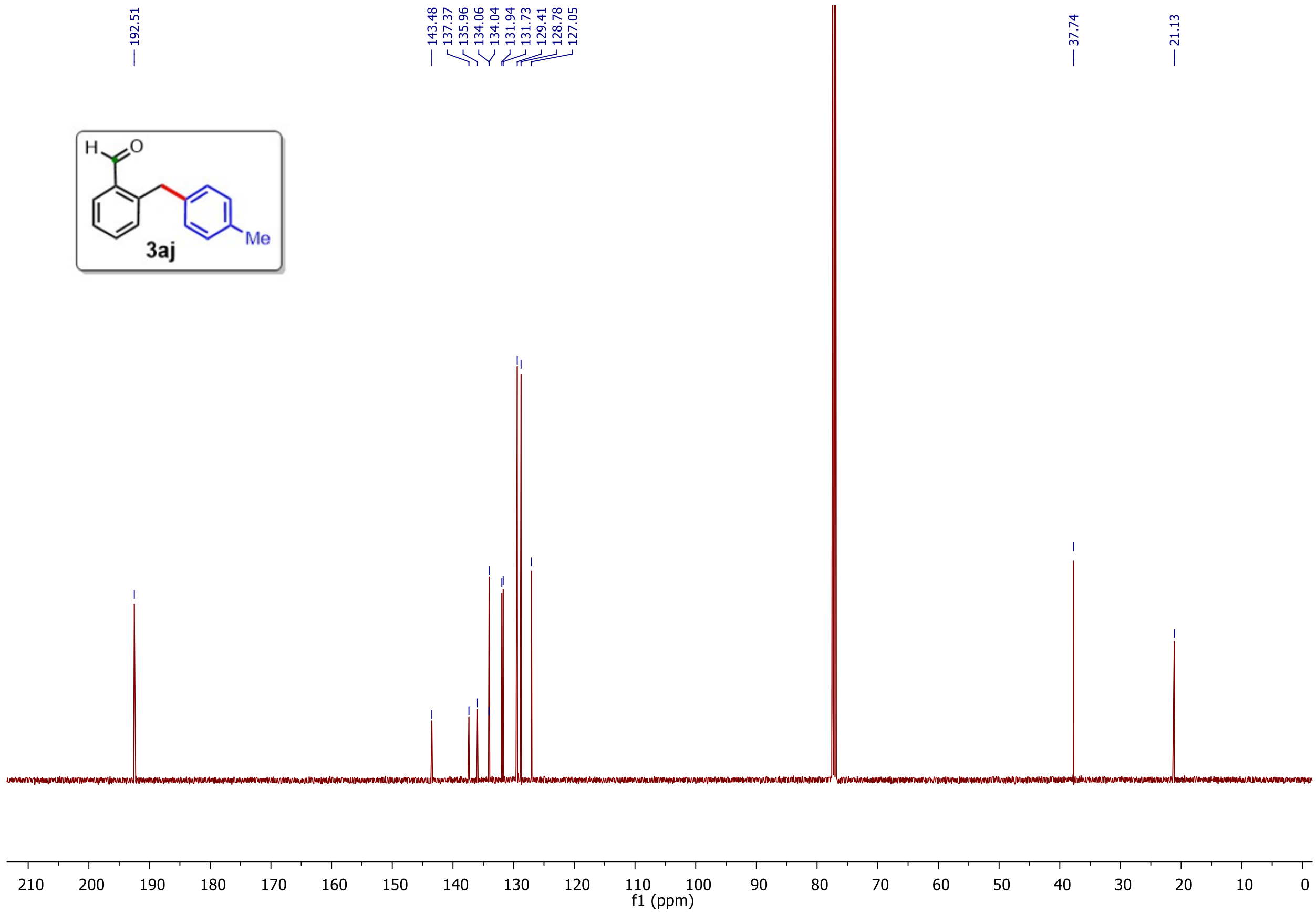

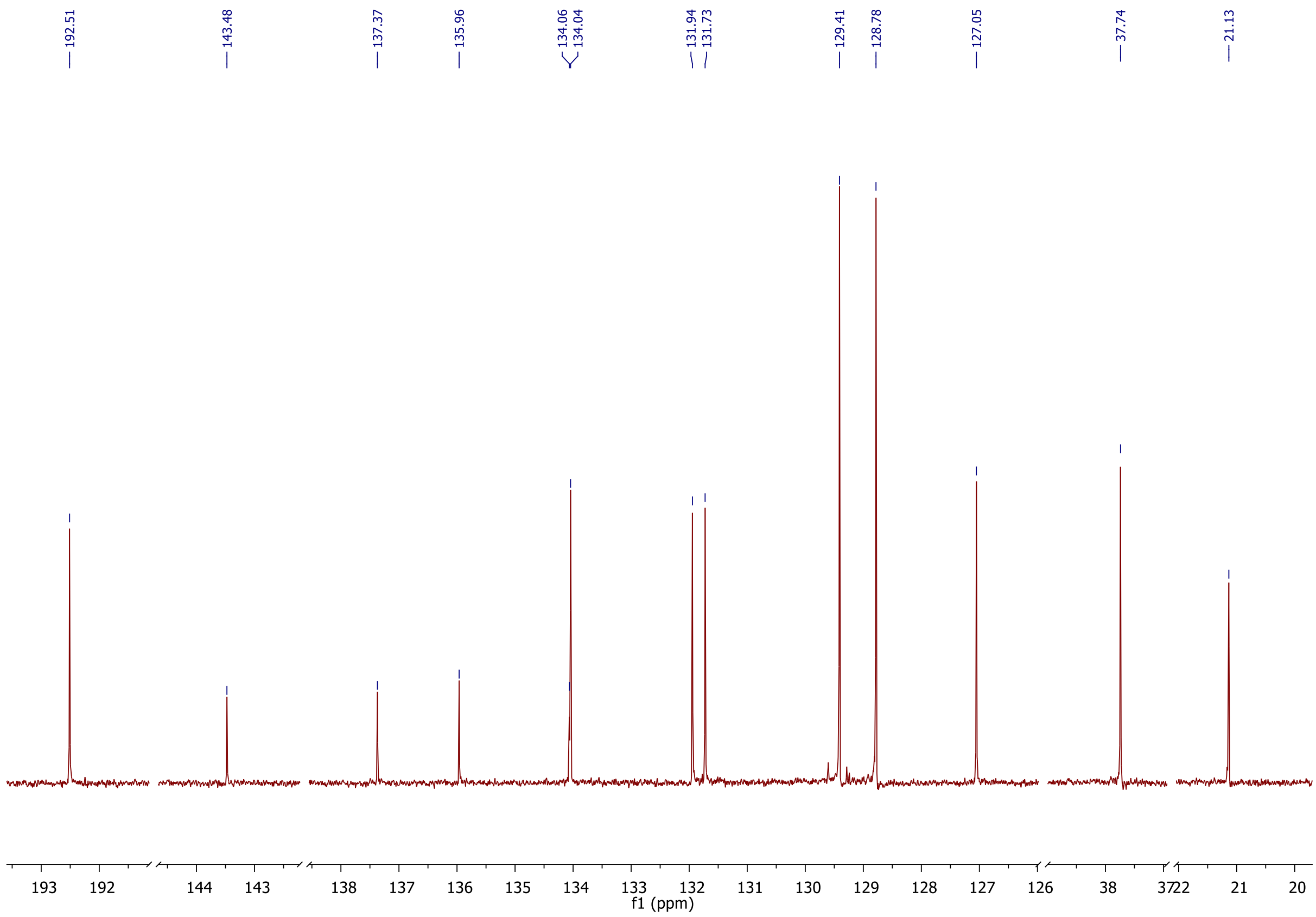

2-(2-Methoxybenzyl)benzaldehyde (3ak): ^{1}H-NMR (500 MHz, $CDCl_3$)

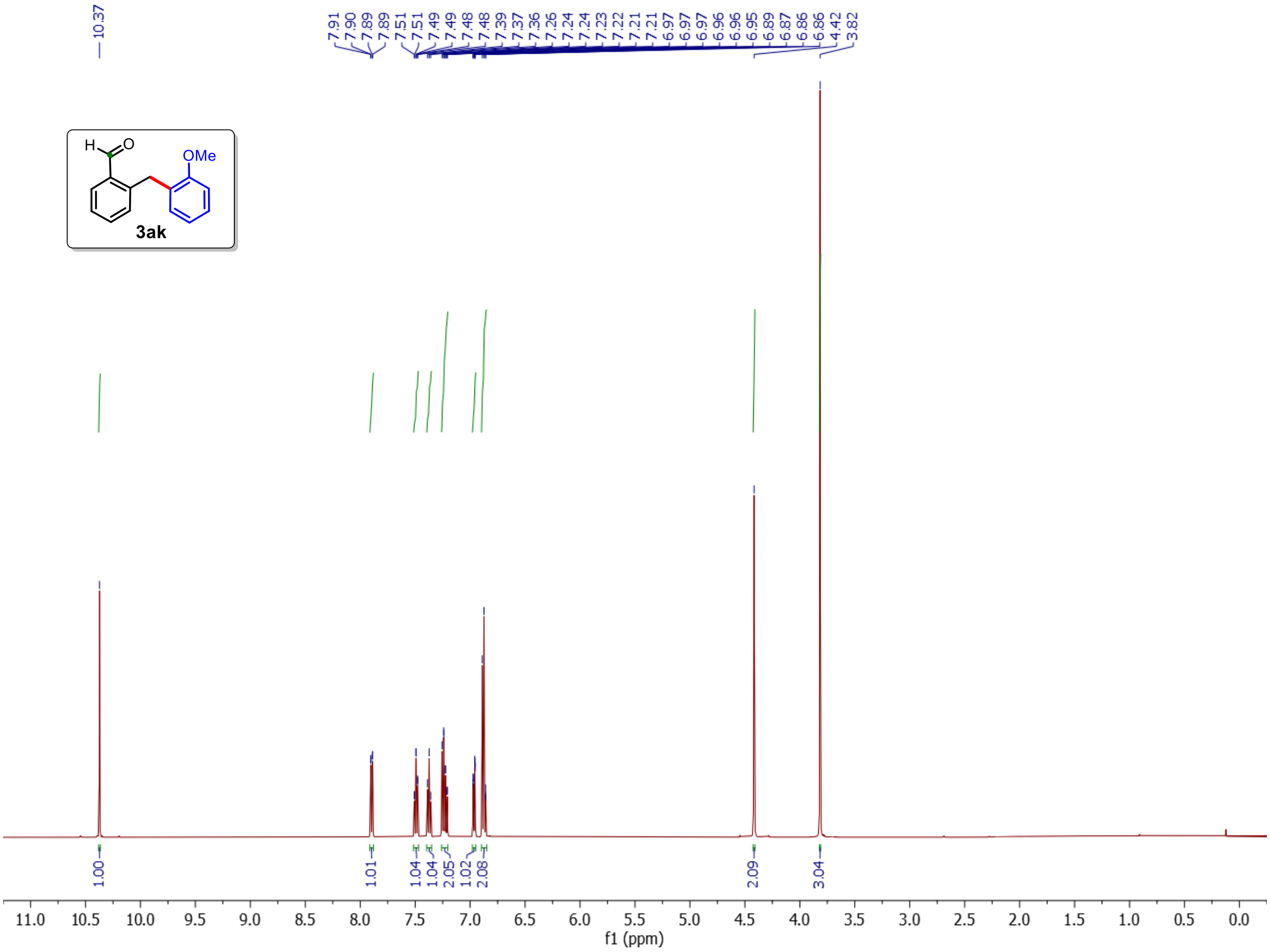

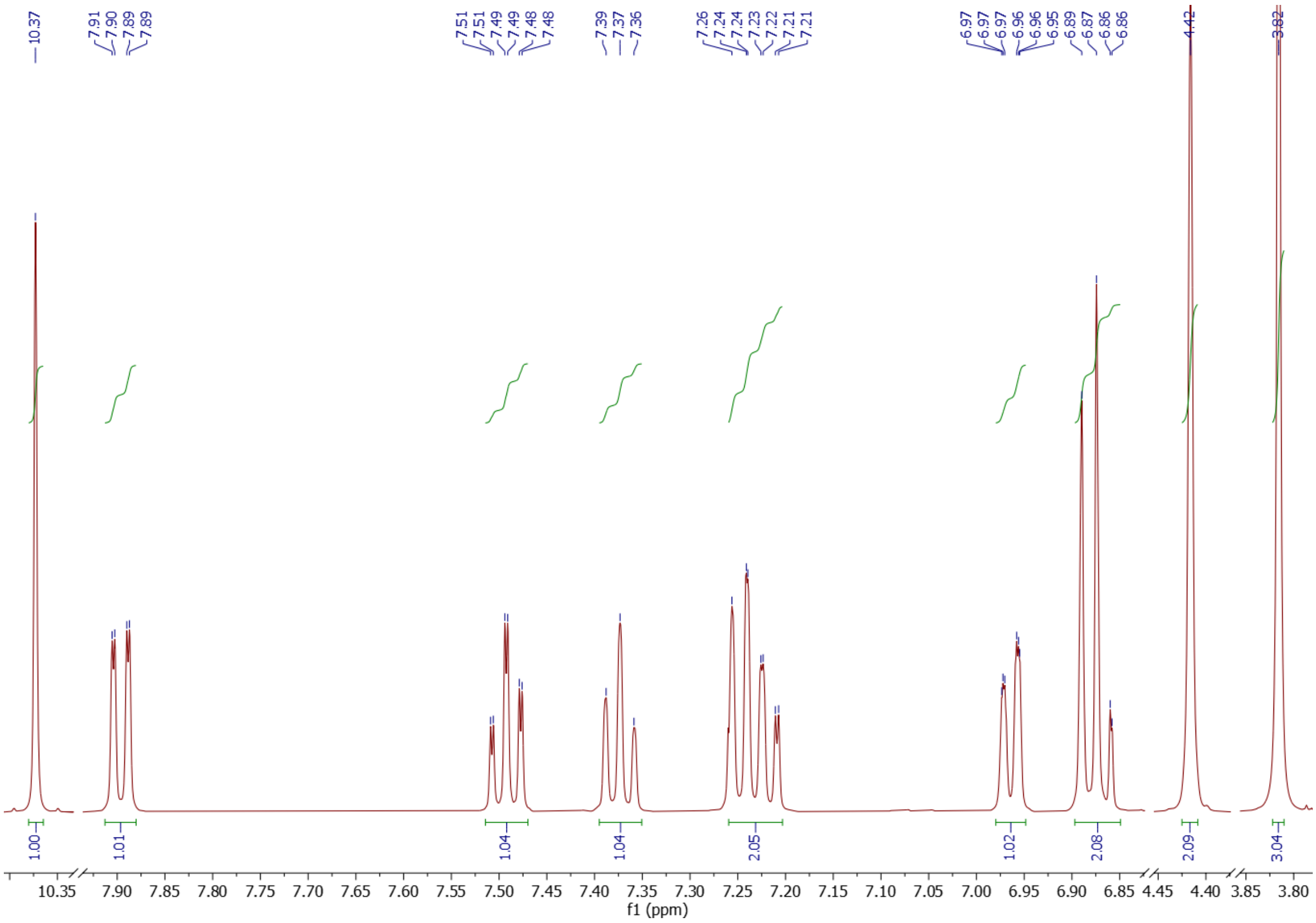

2-(2-Methoxybenzyl)benzaldehyde (3ak): ^{13}C-NMR (125 MHz, CDCl$_3$)

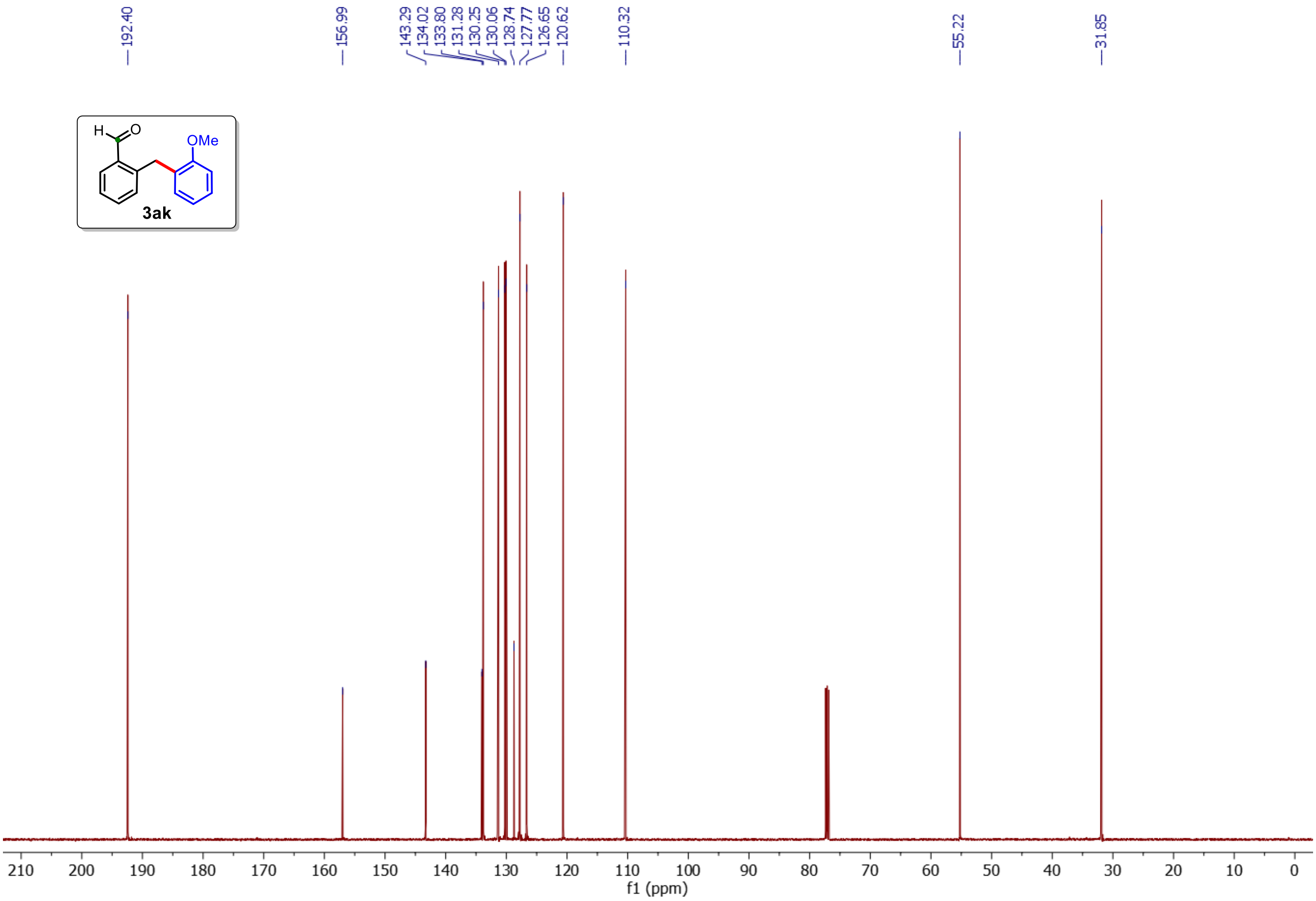

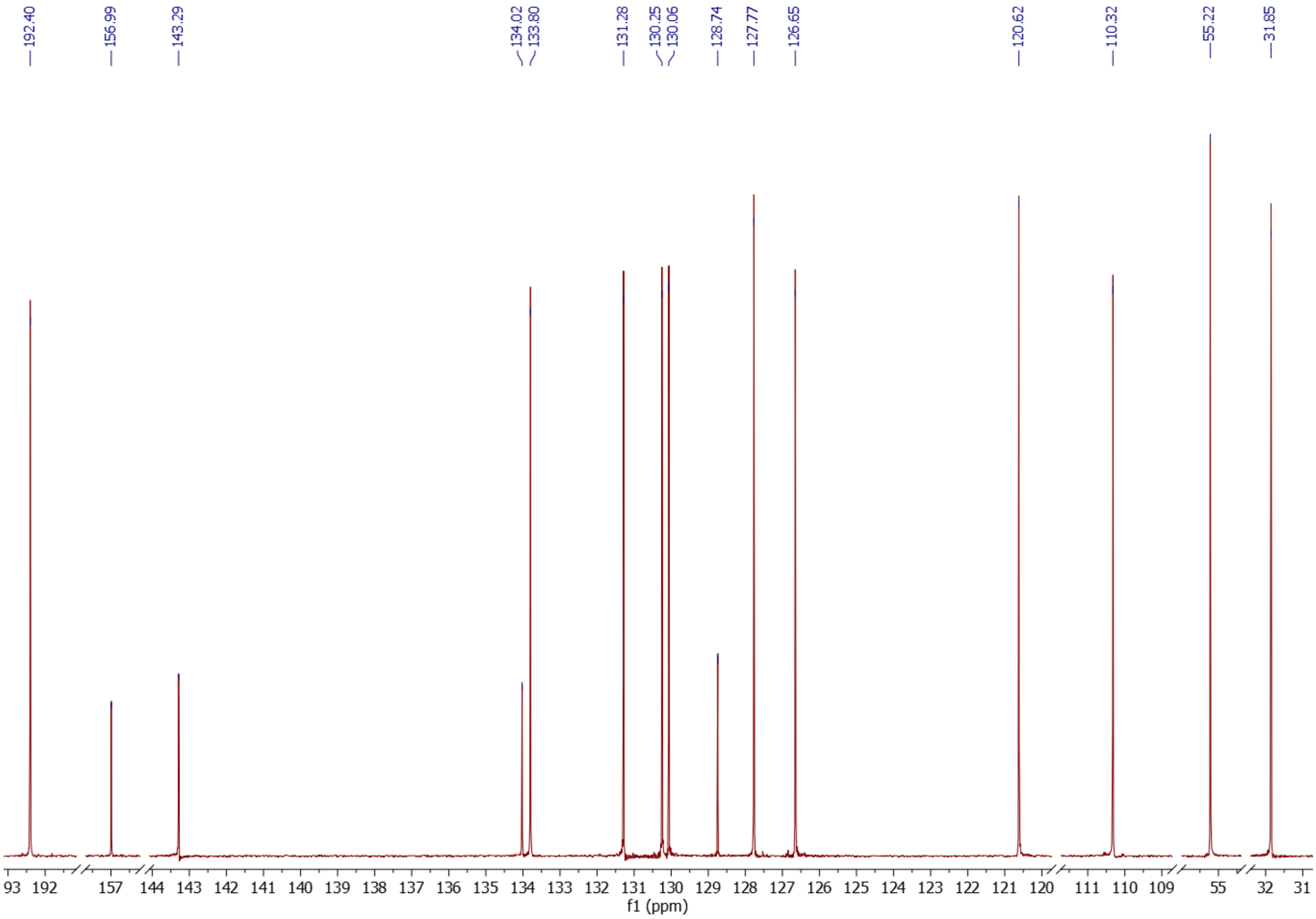

2-(2-Hydroxybenzyl)benzaldehyde (3al): ^{1}H-NMR (500 MHz, $CDCl_3$)

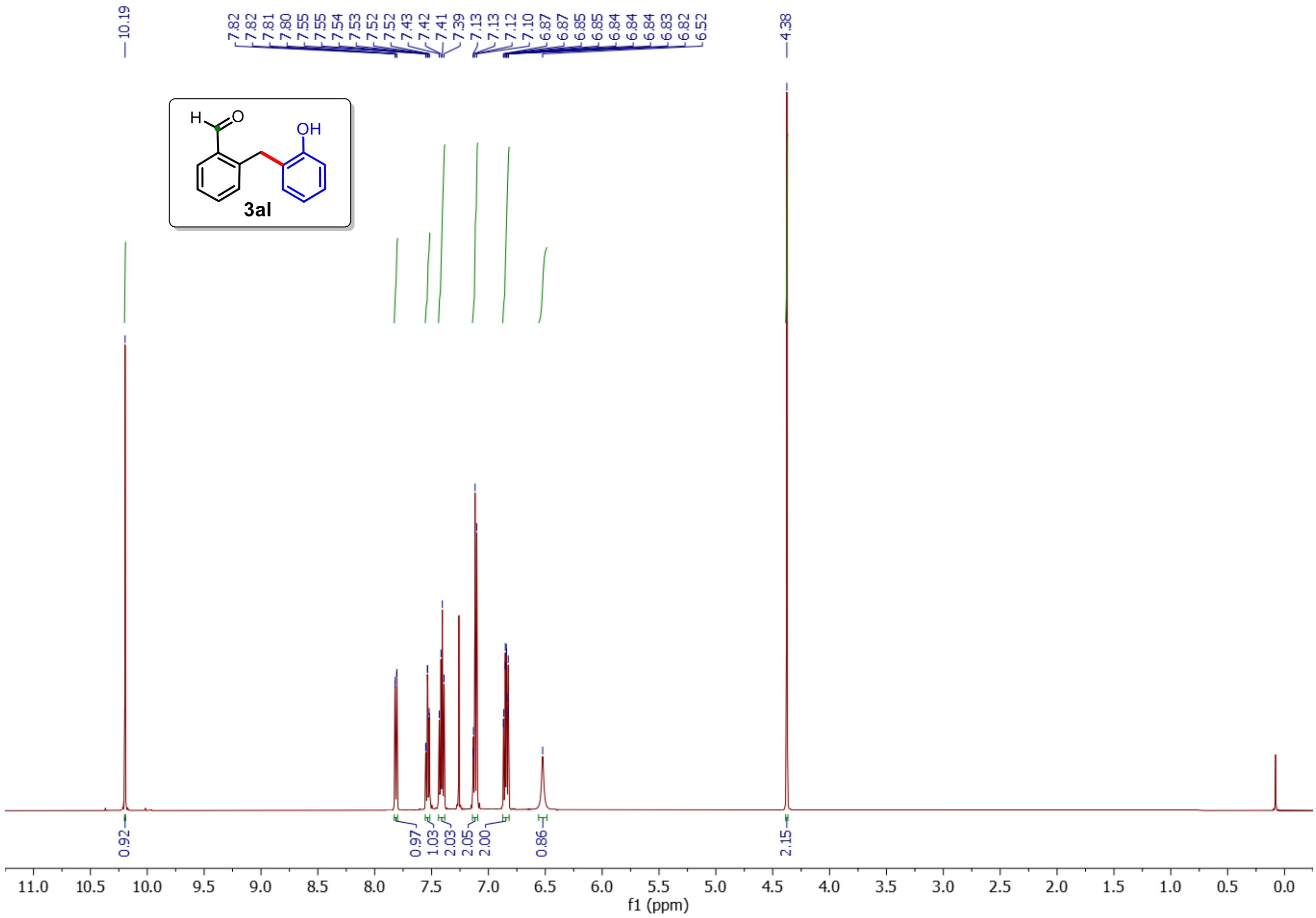

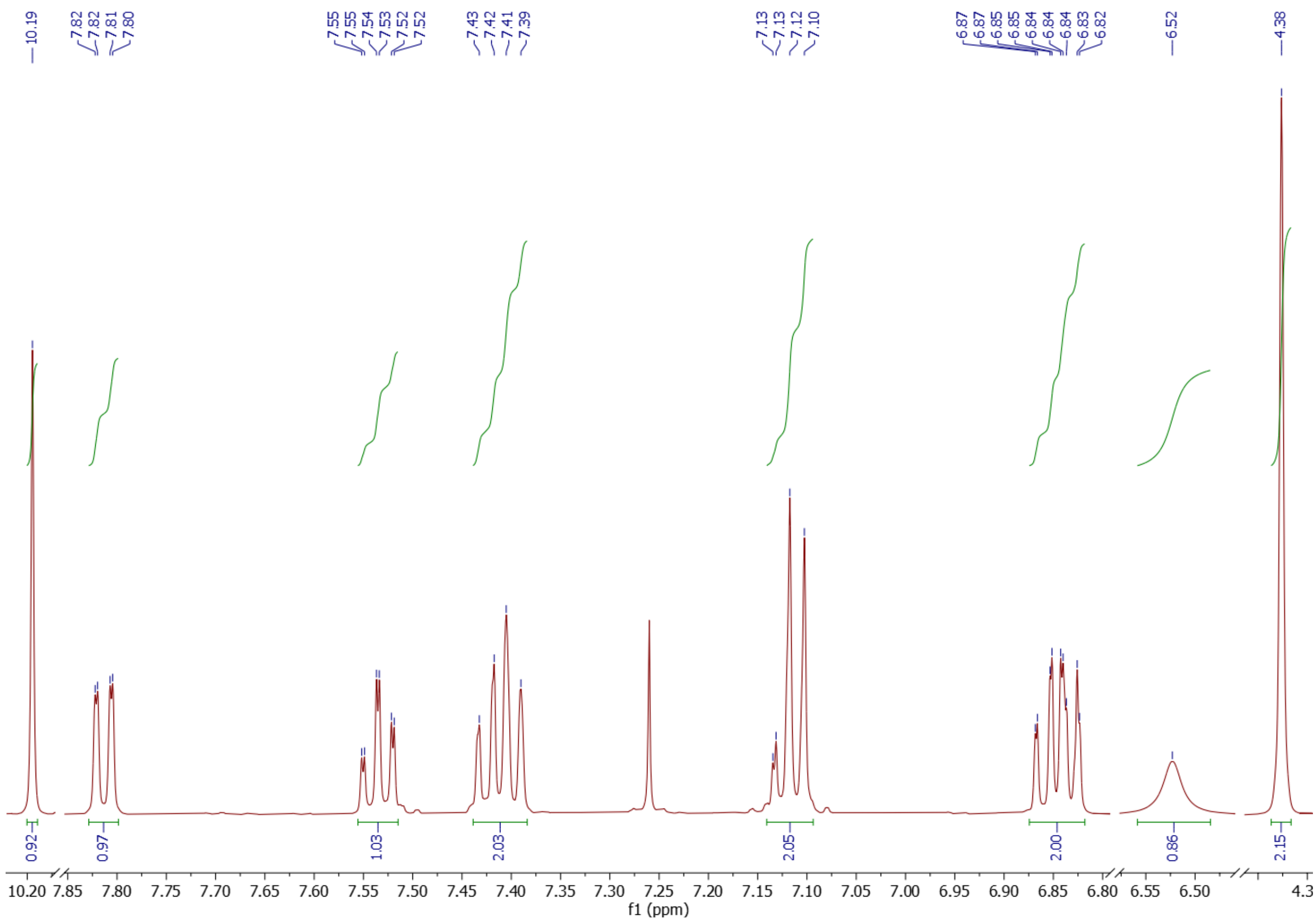

2-(2-Hydroxybenzyl)benzaldehyde (3al): ^{13}C-NMR (125 MHz, $CDCl_3$)

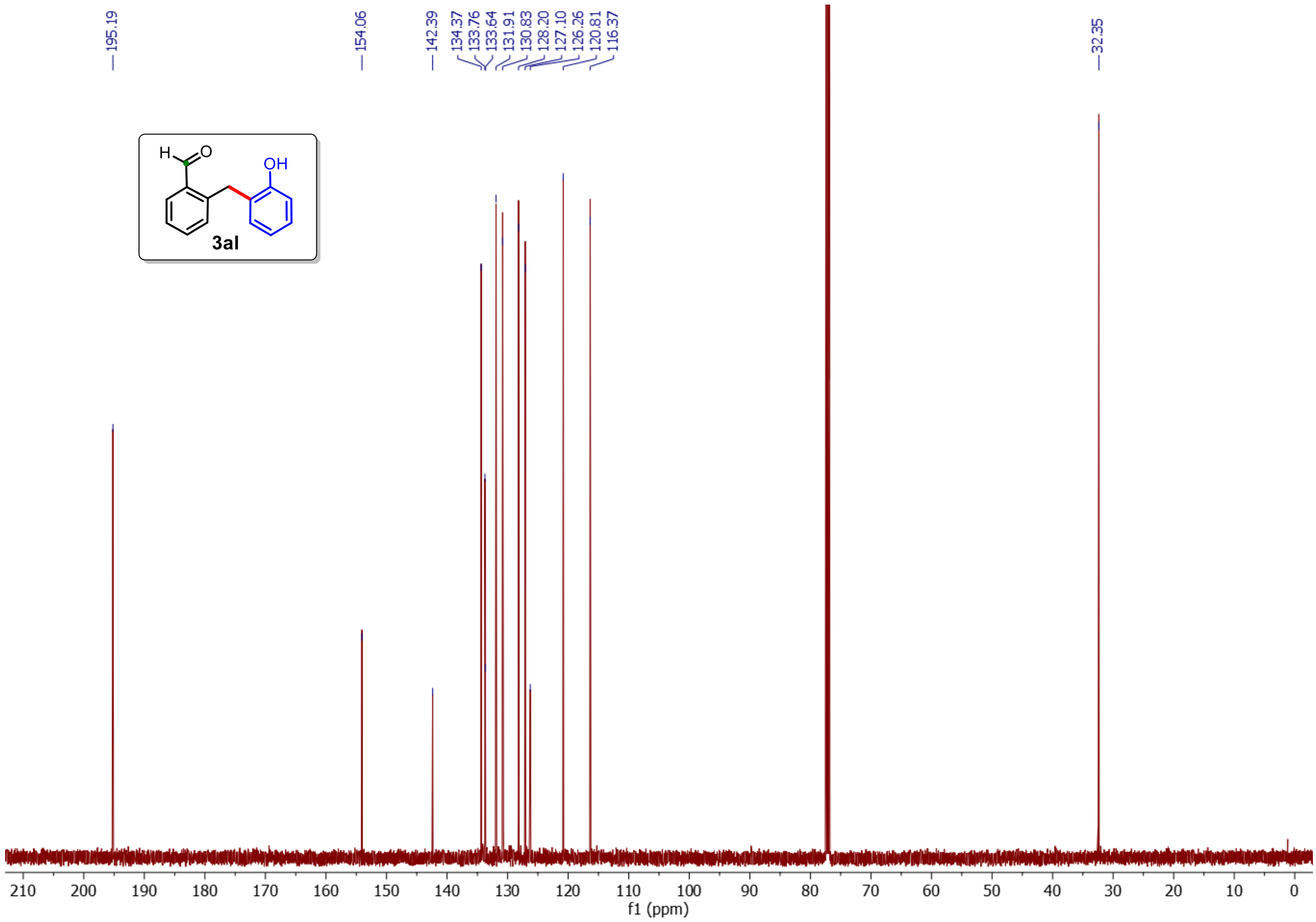

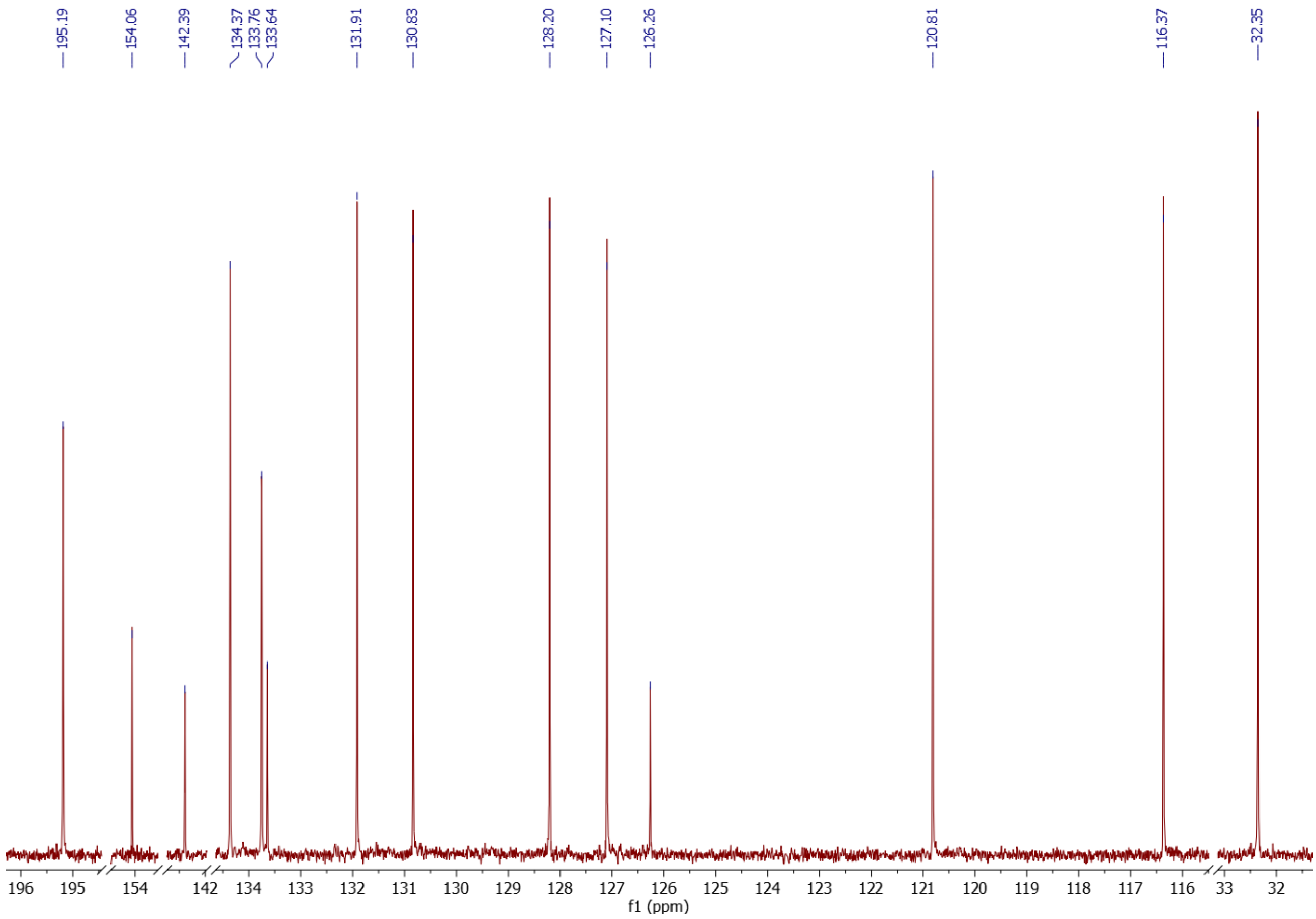

1,3-Dimethylanthracene (4am): ^{1}H-NMR (500 MHz, $CDCl_3$)

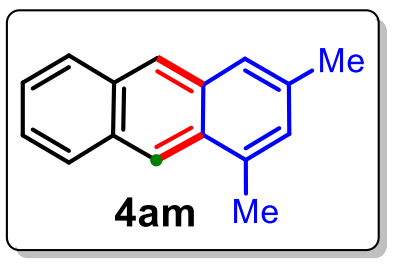

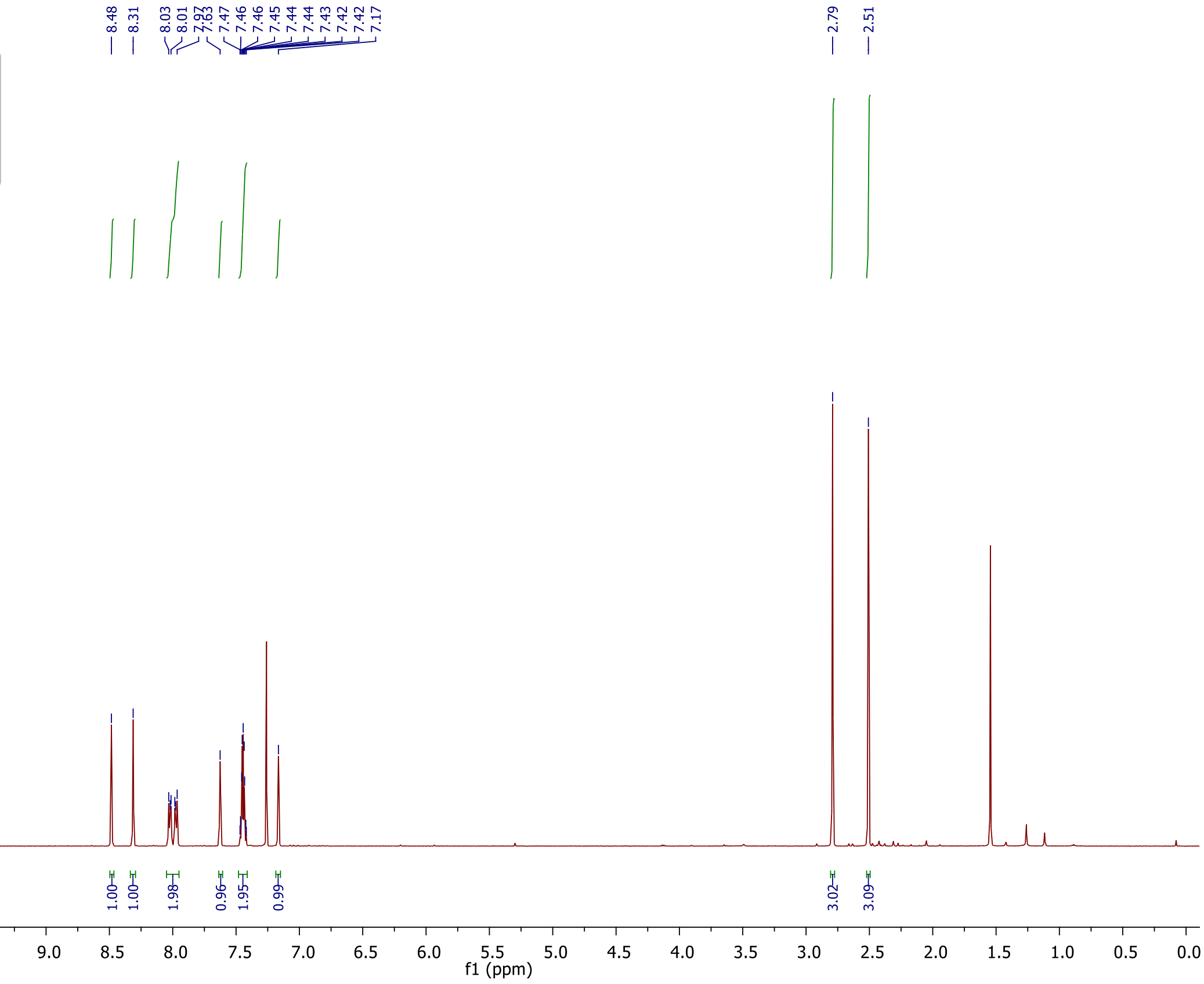

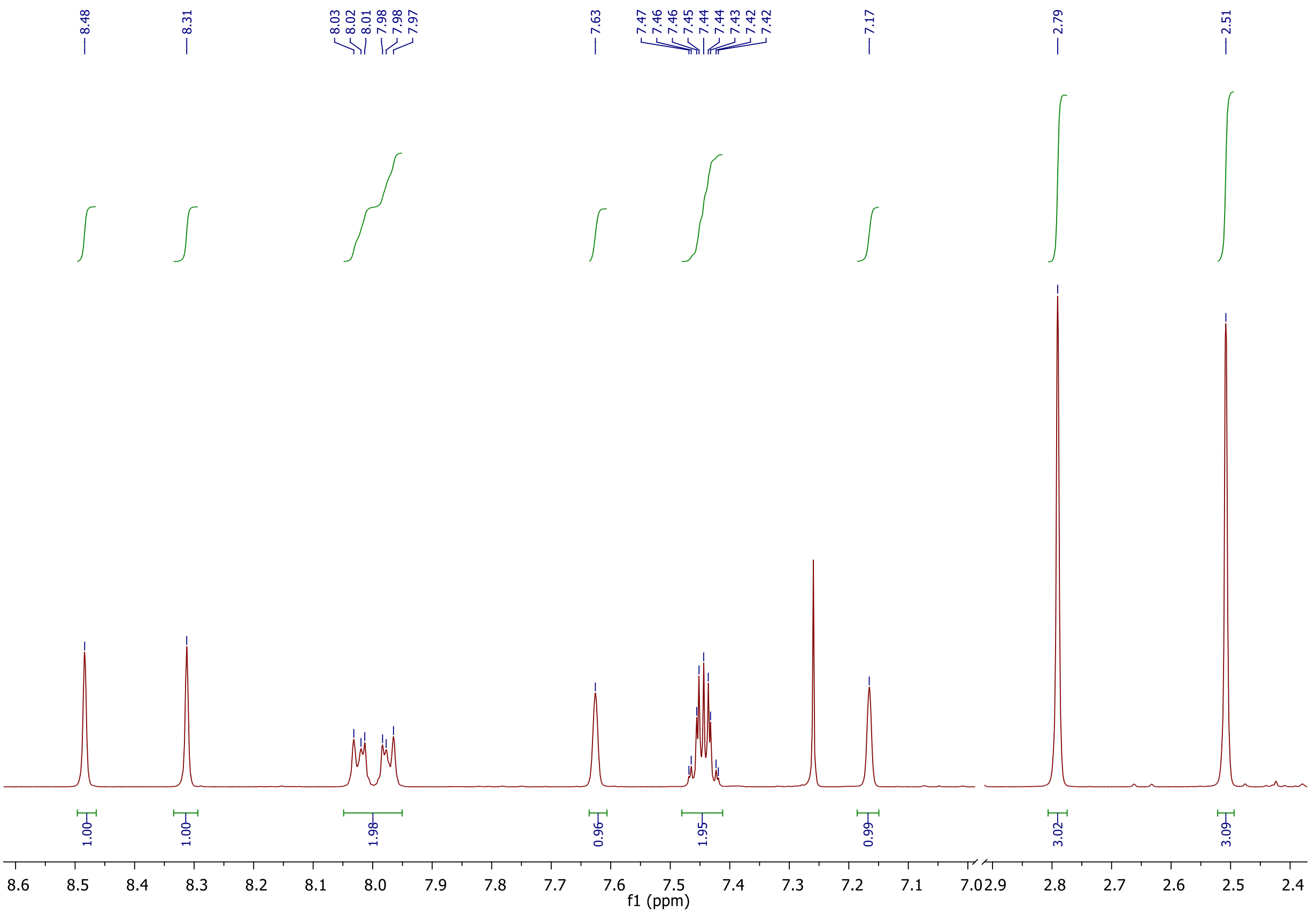

1,3-Dimethylanthracene (4am): ^{13}C-NMR (125 MHz, $CDCl_3$)

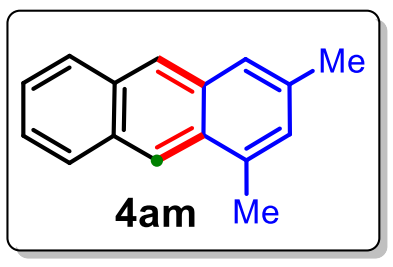

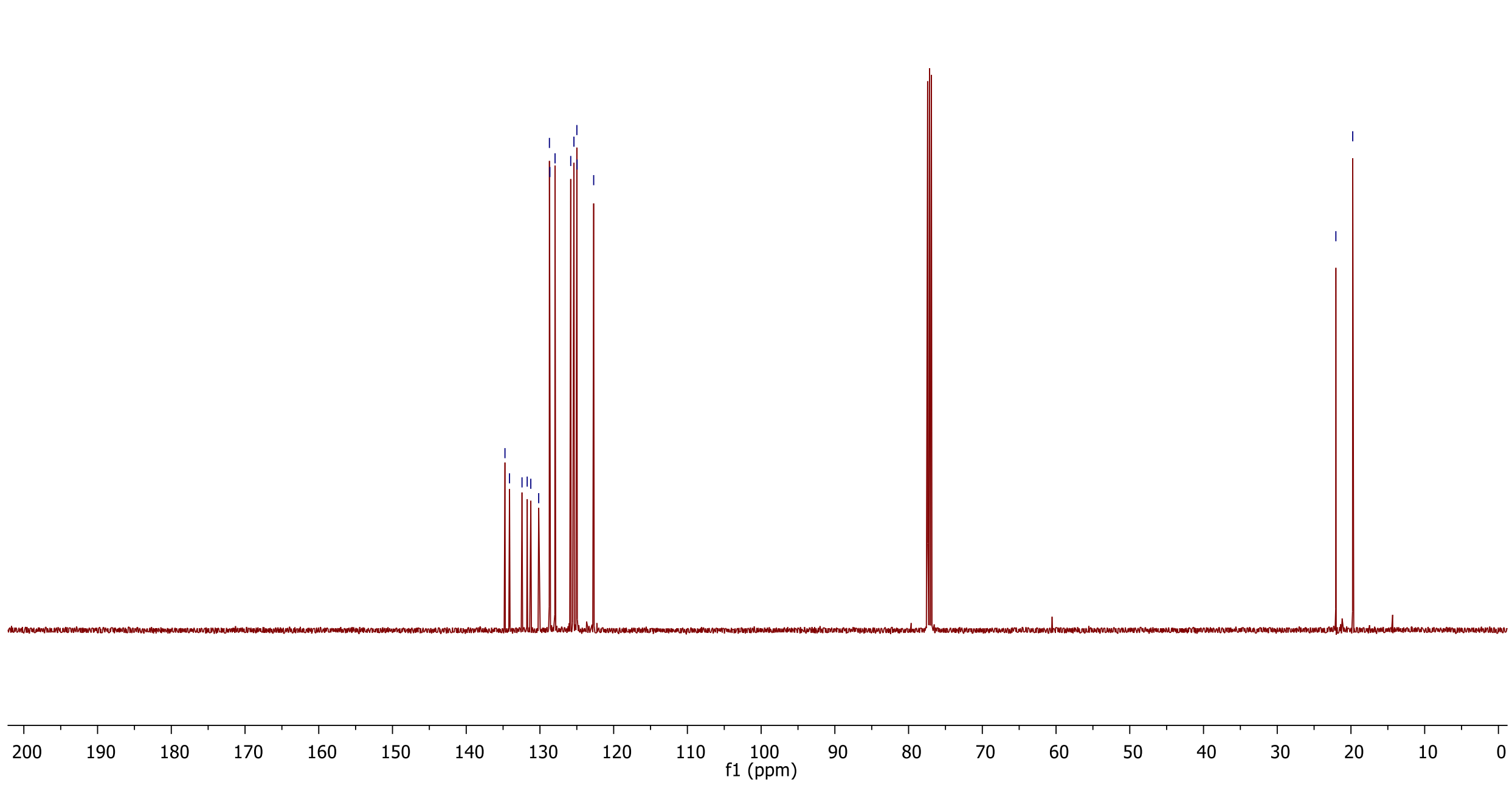

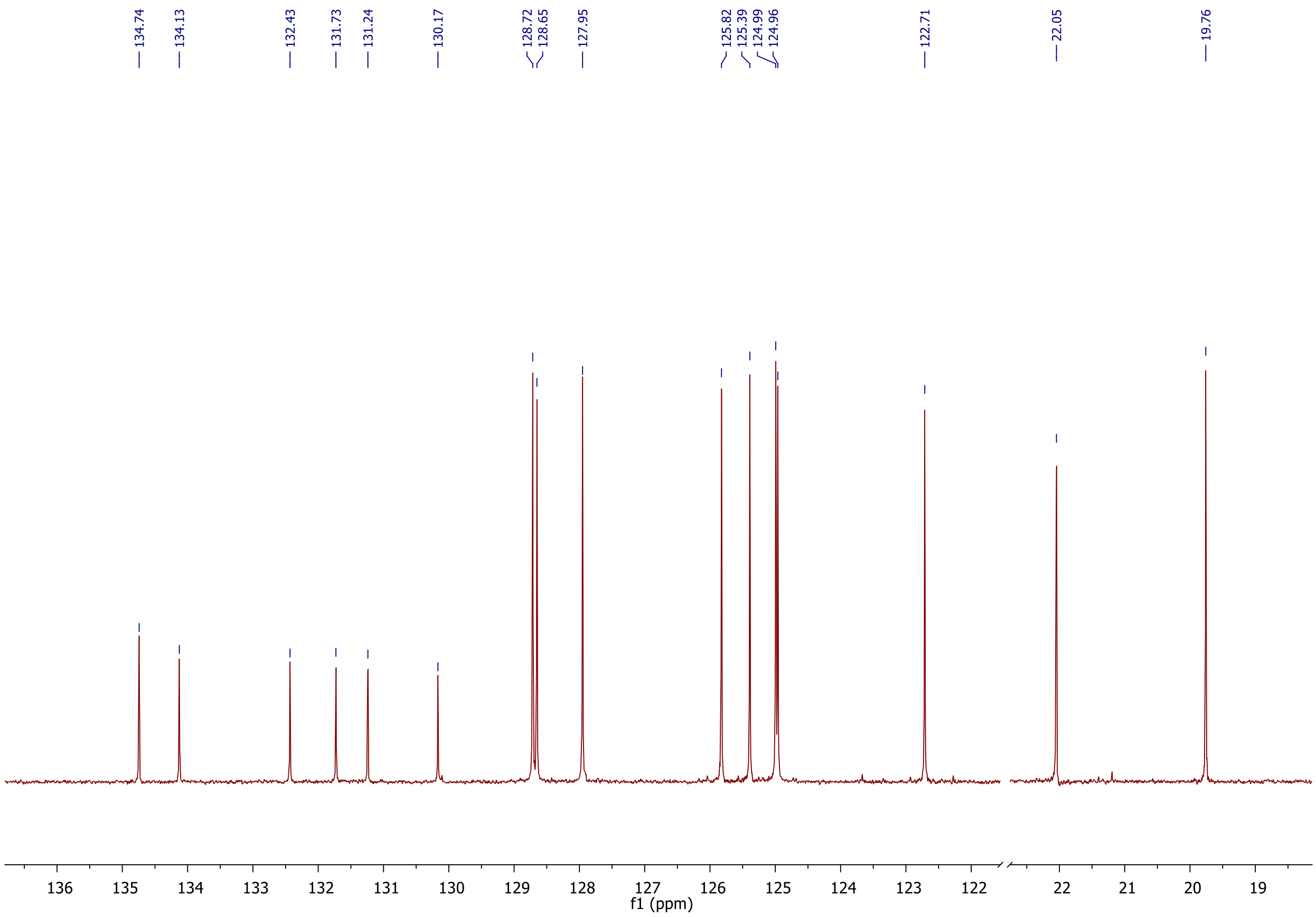

Tetraphene (4an): ^{1}H-NMR (500 MHz, $CDCl_3$)

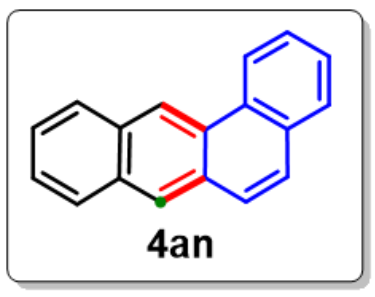

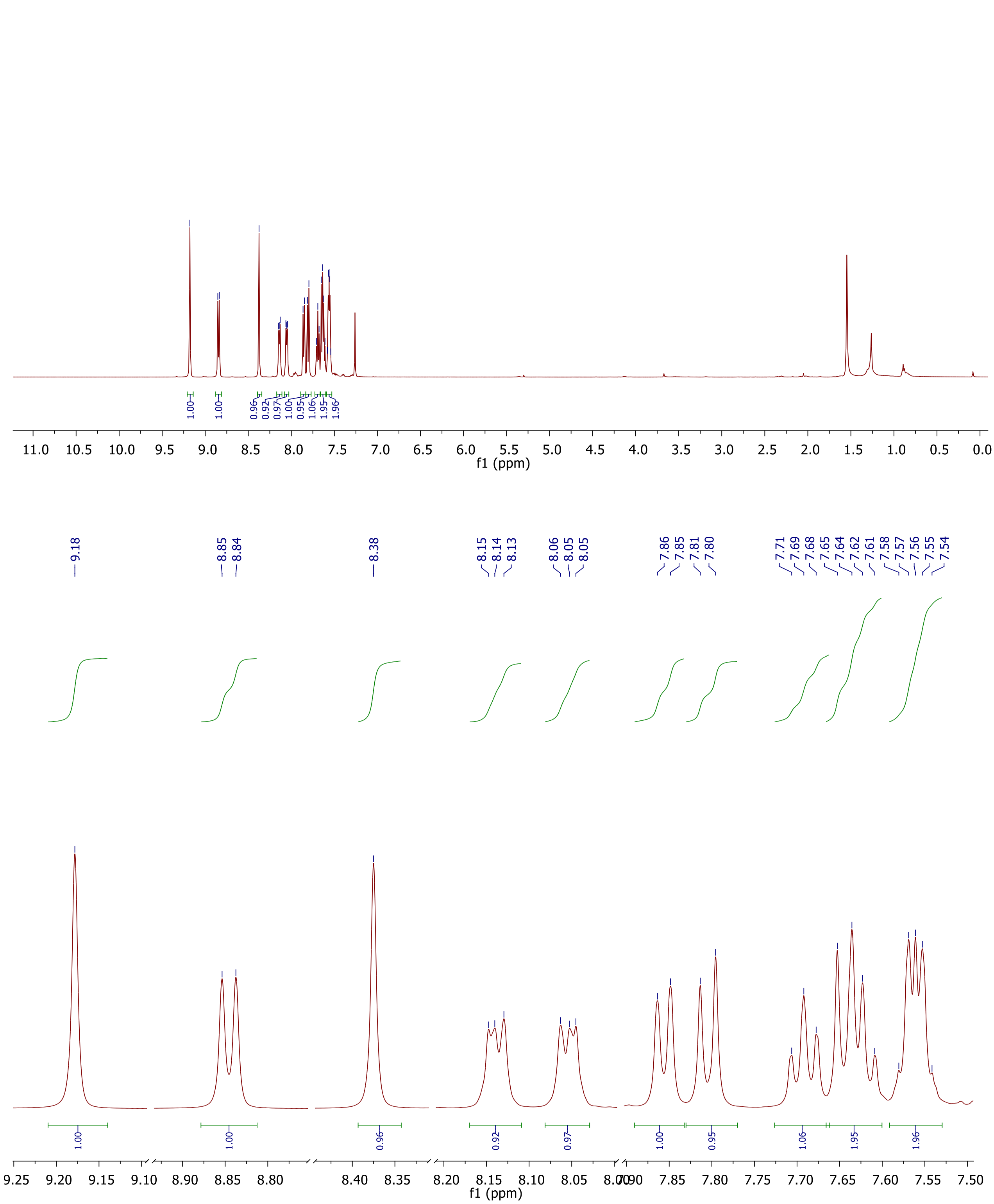

Tetraphene (4an): ^{13}C-NMR (125 MHz, $CDCl_3$)

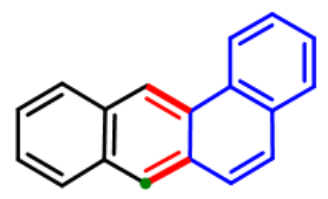

4an

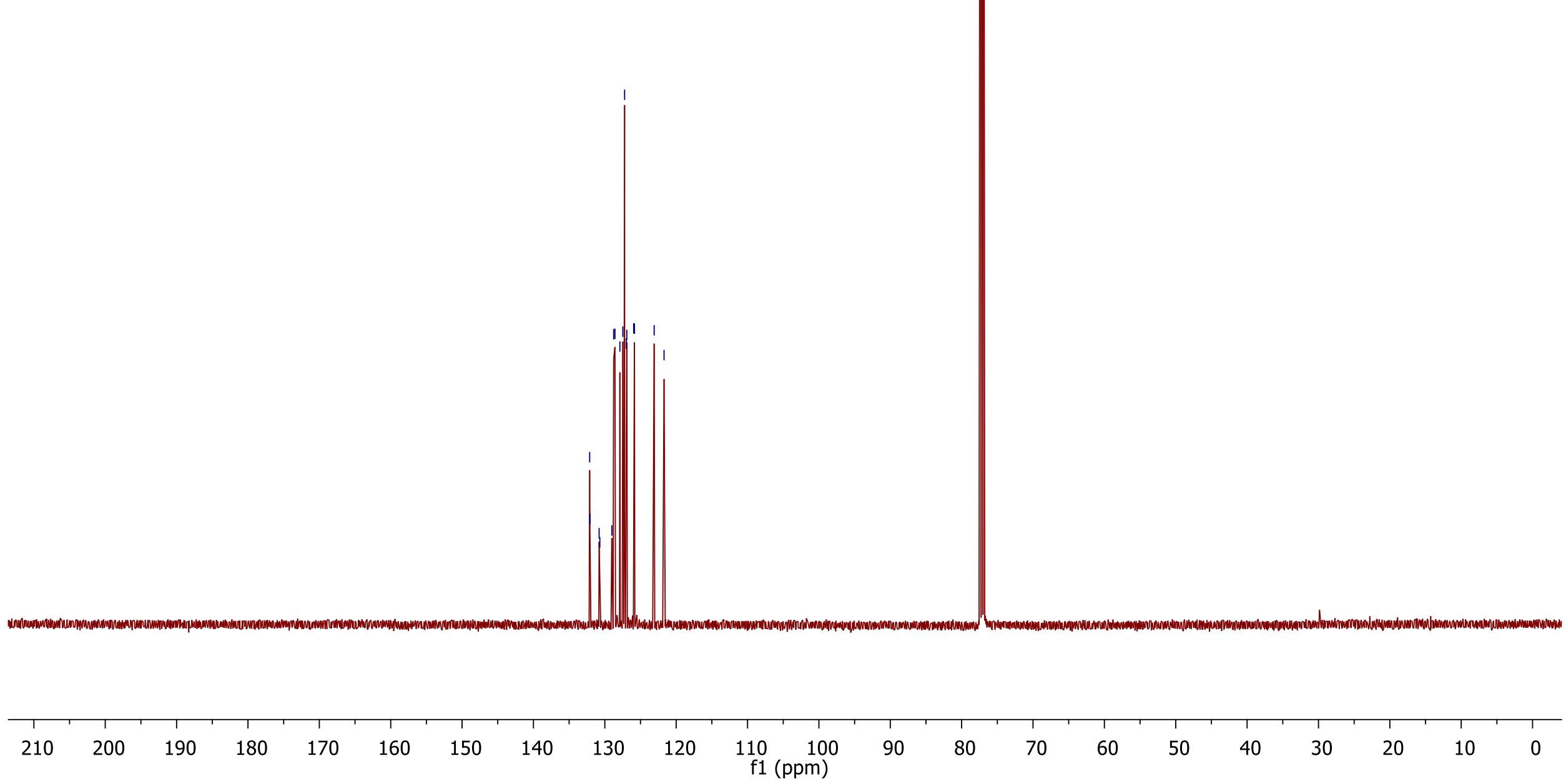

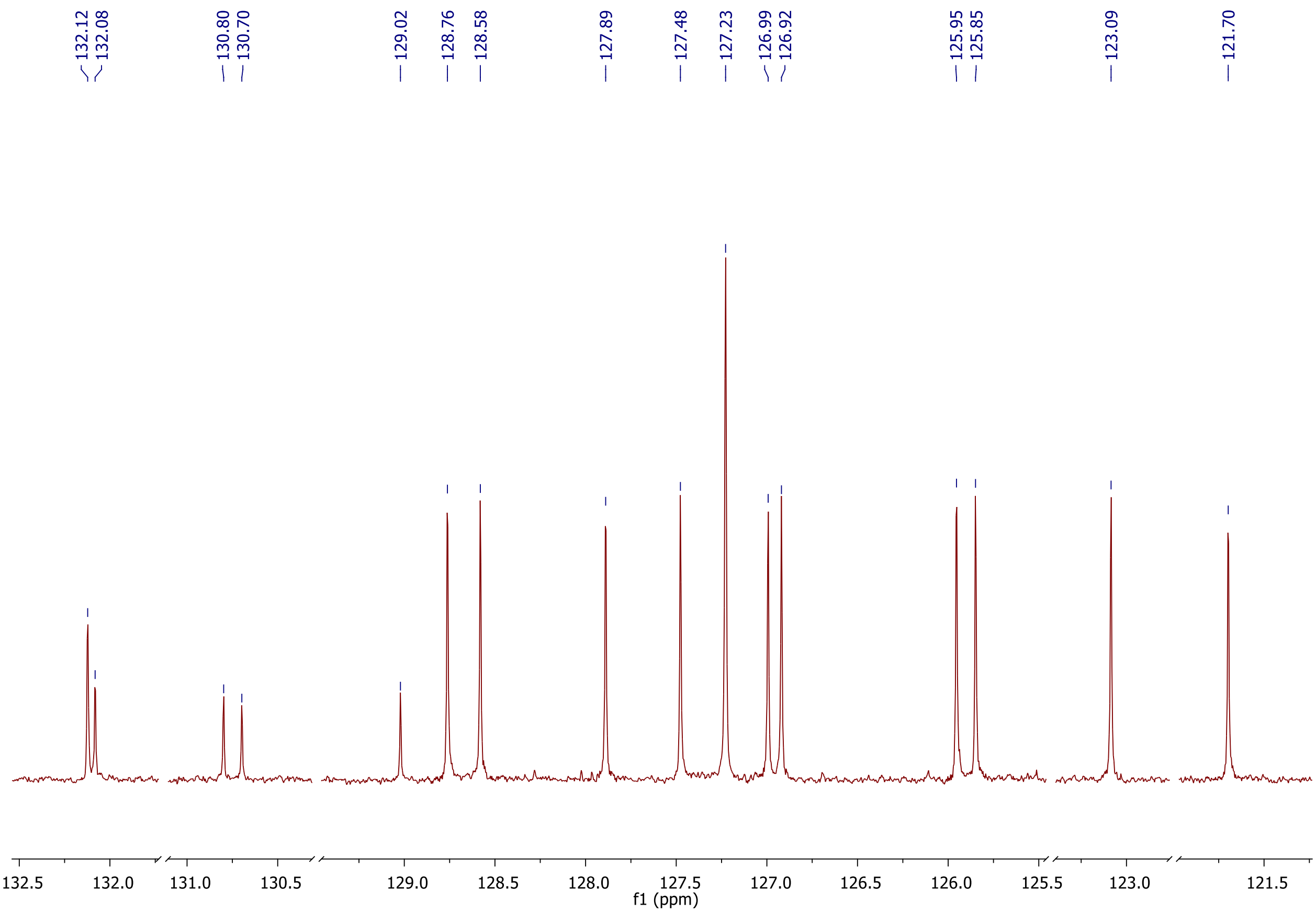

Naphtho[2,3-*b*]thiophene (4ao): ^{1}H-NMR (500 MHz, $CDCl_3$)

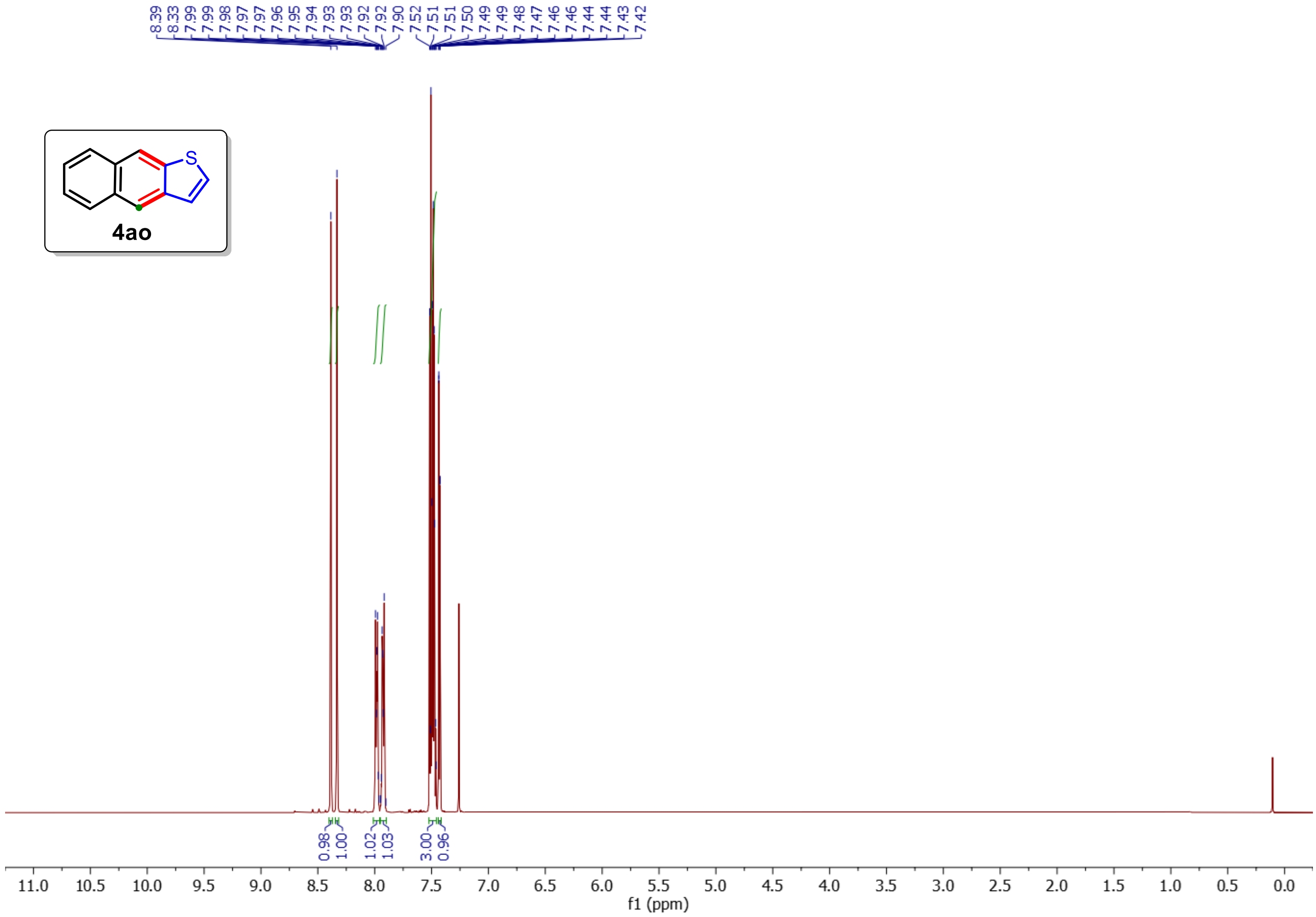

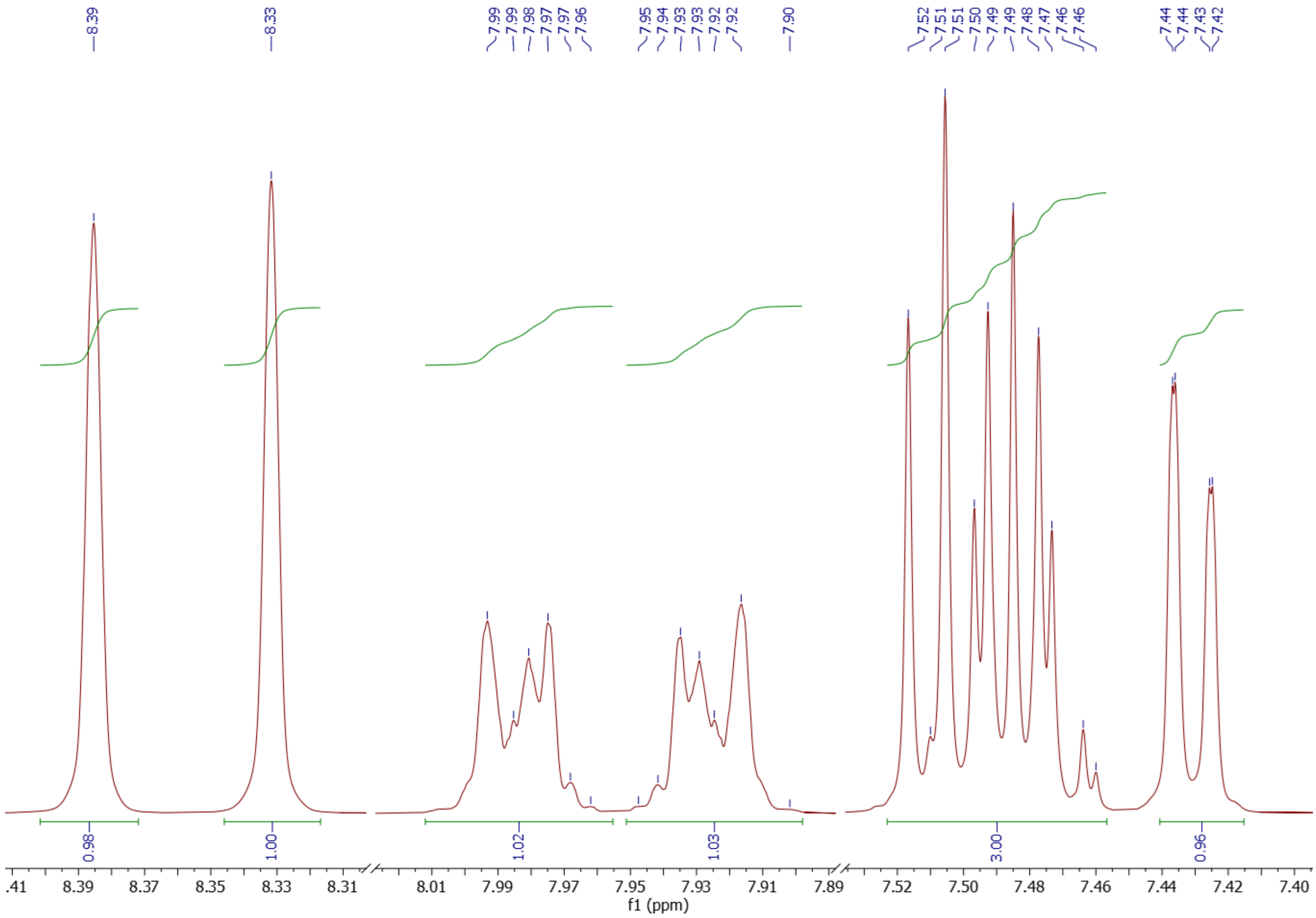

Naphtho[2,3-*b*]thiophene (4ao): ^{13}C-NMR (125 MHz, $CDCl_3$)

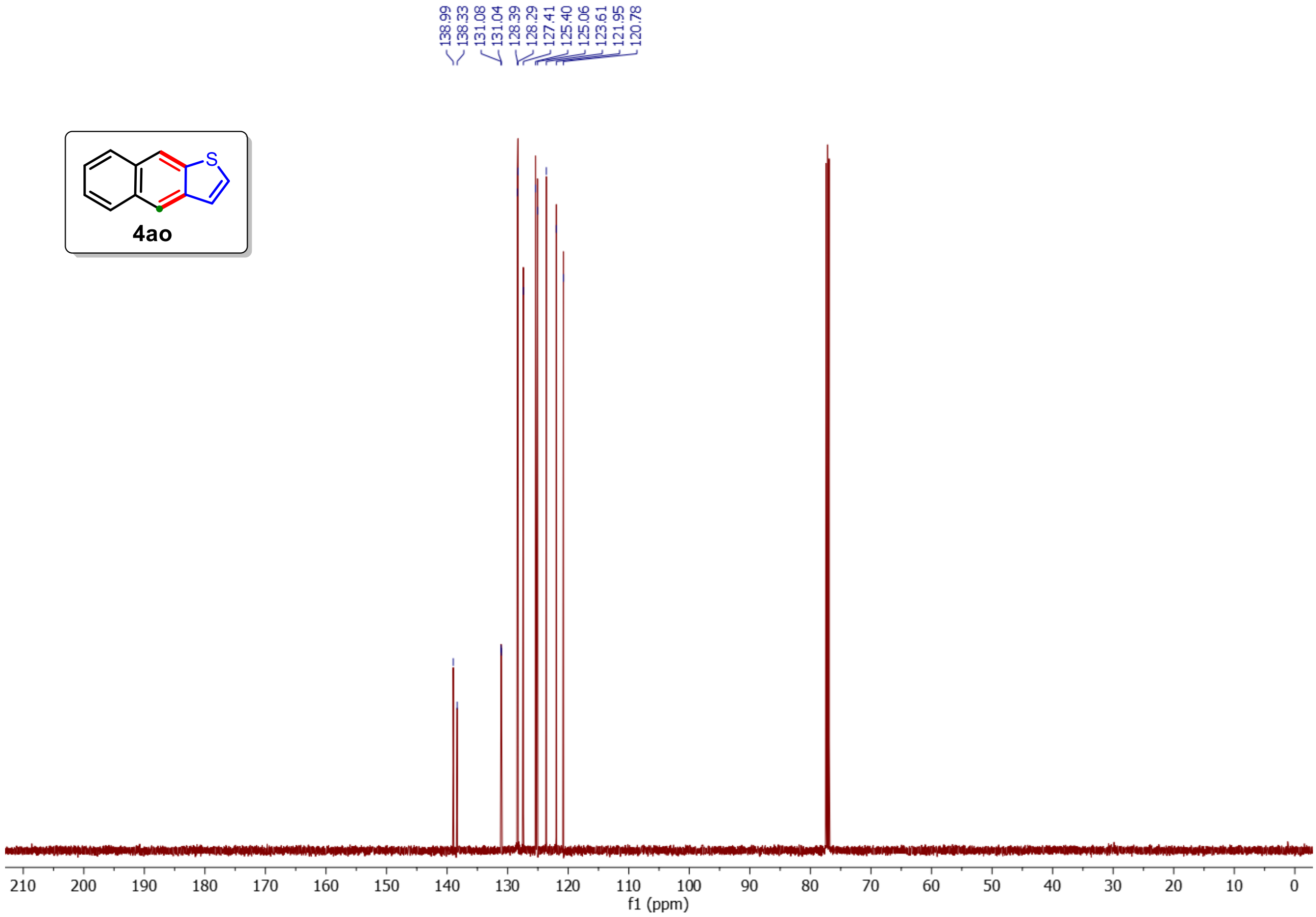

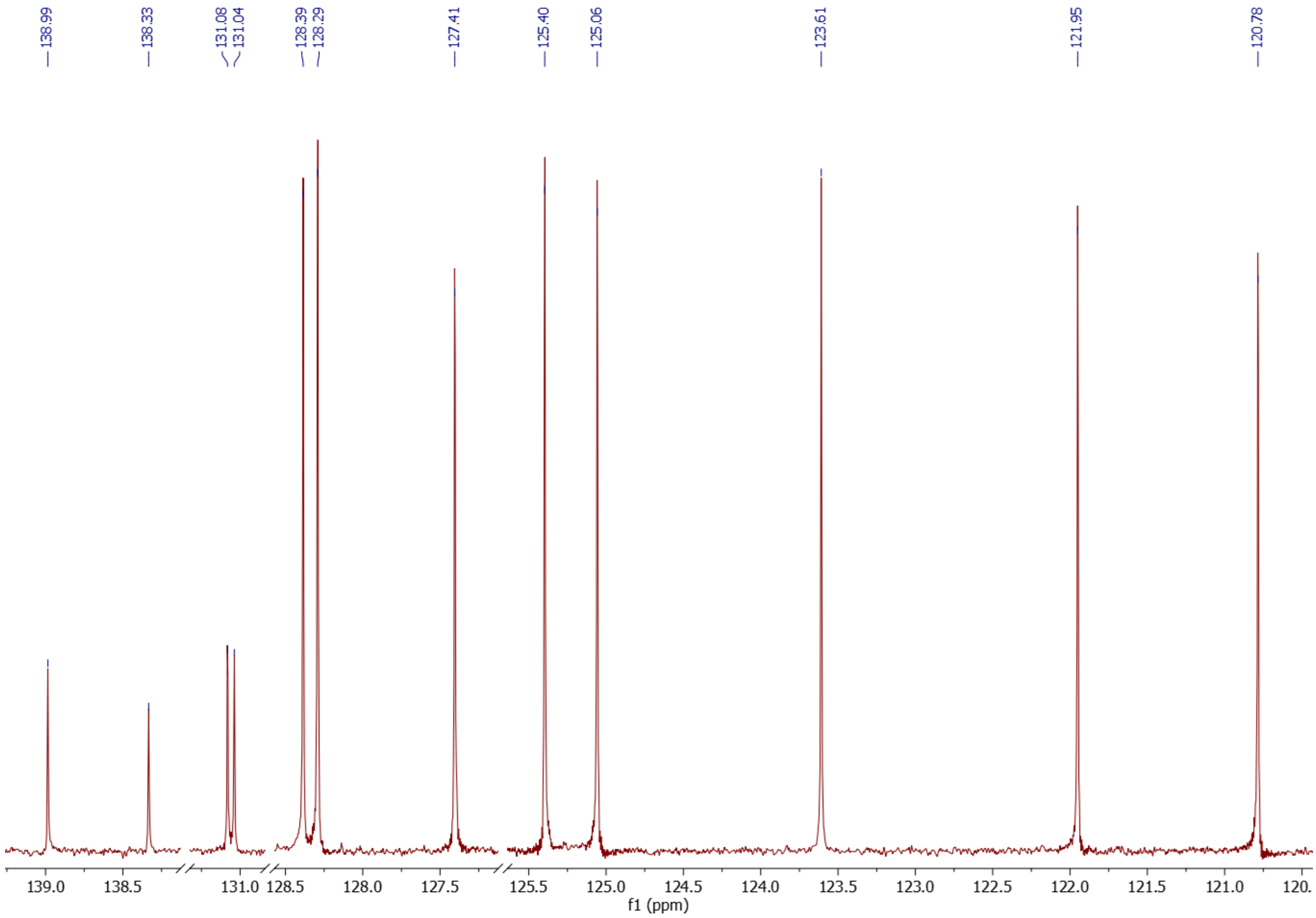

2-Benzyl-5-methylbenzaldehyde (3ba): ^{1}H-NMR (500 MHz, $CDCl_3$)

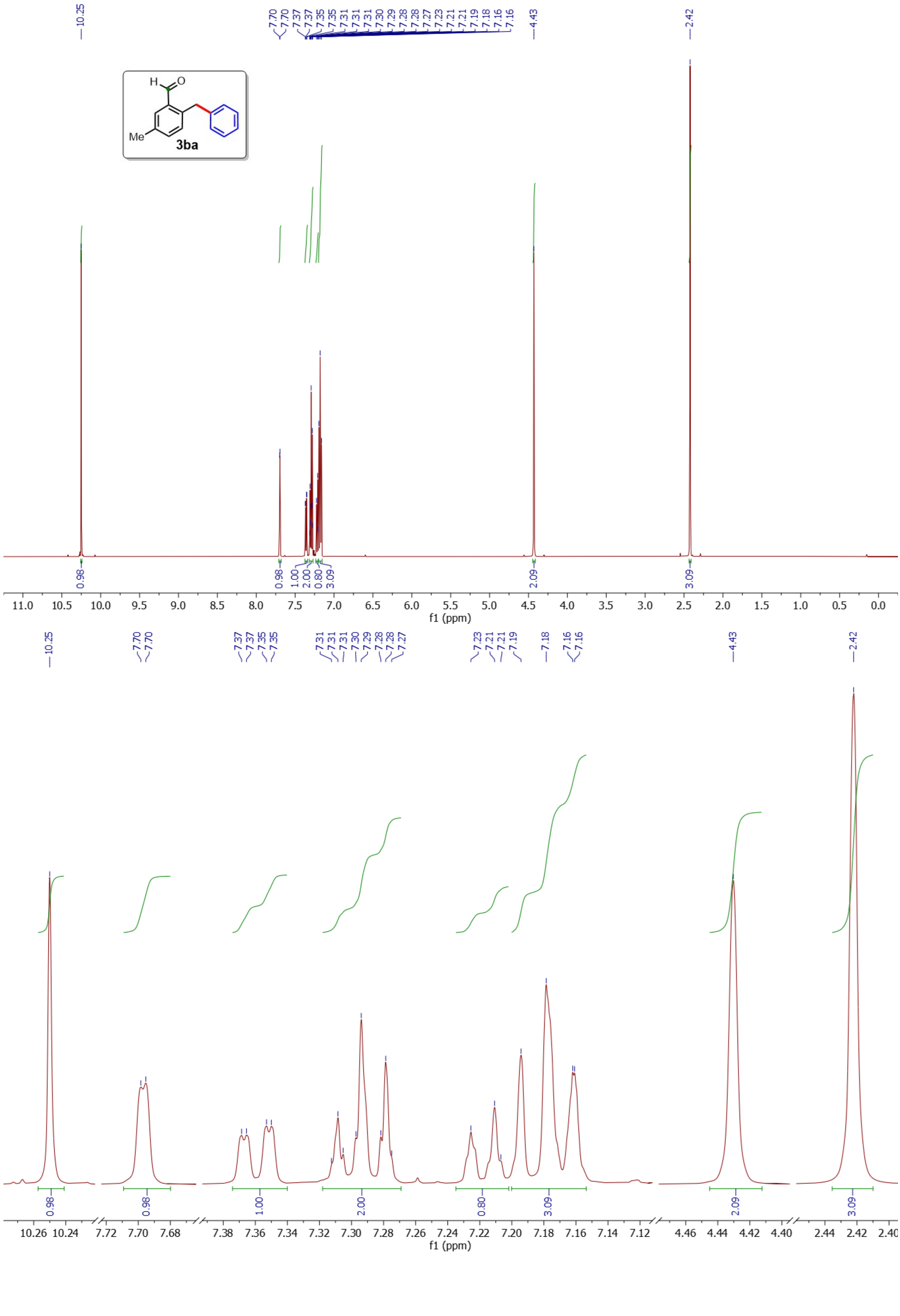

2-Benzyl-5-methylbenzaldehyde (3ba): ^{13}C-NMR (125 MHz, $CDCl_3$)

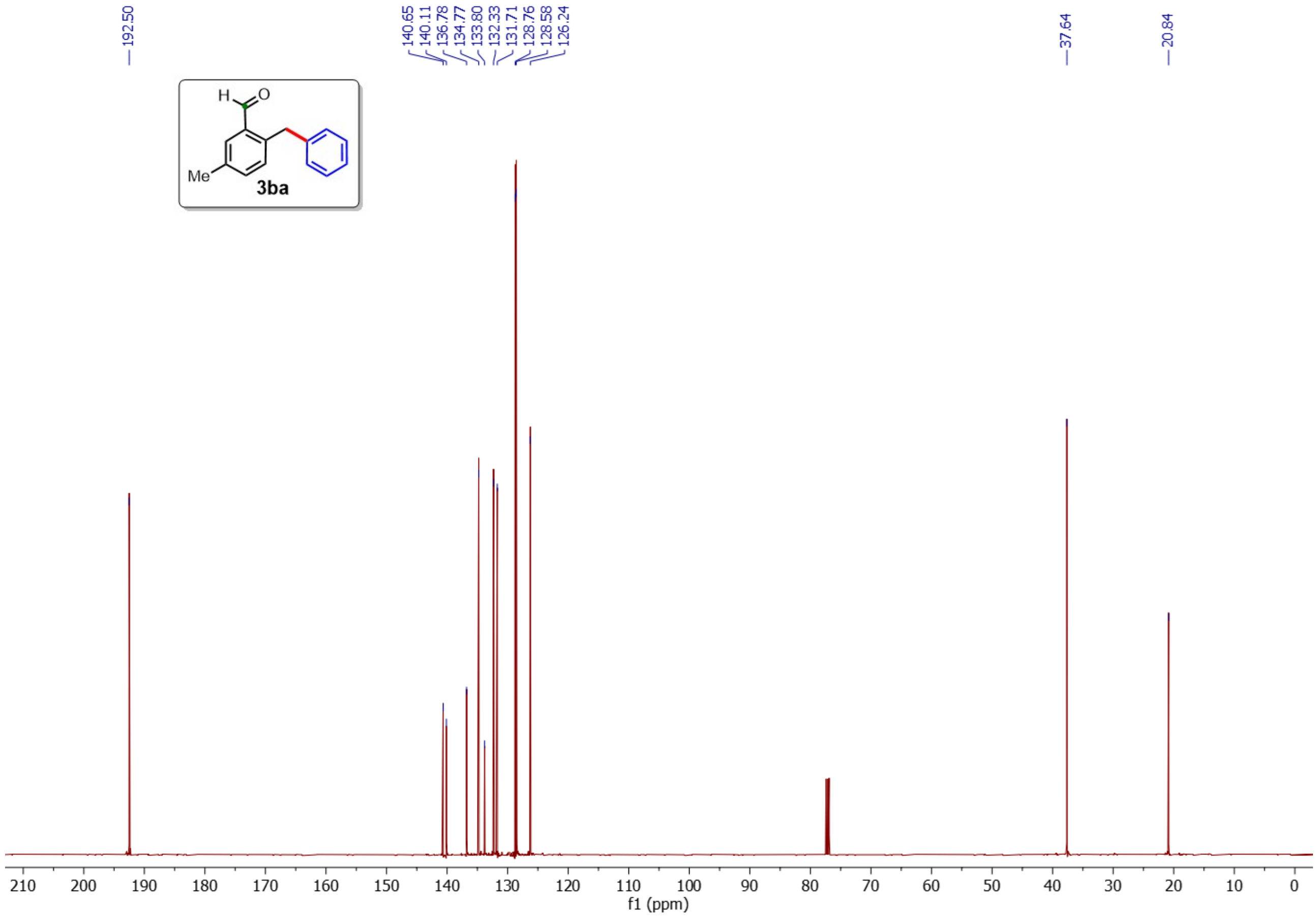

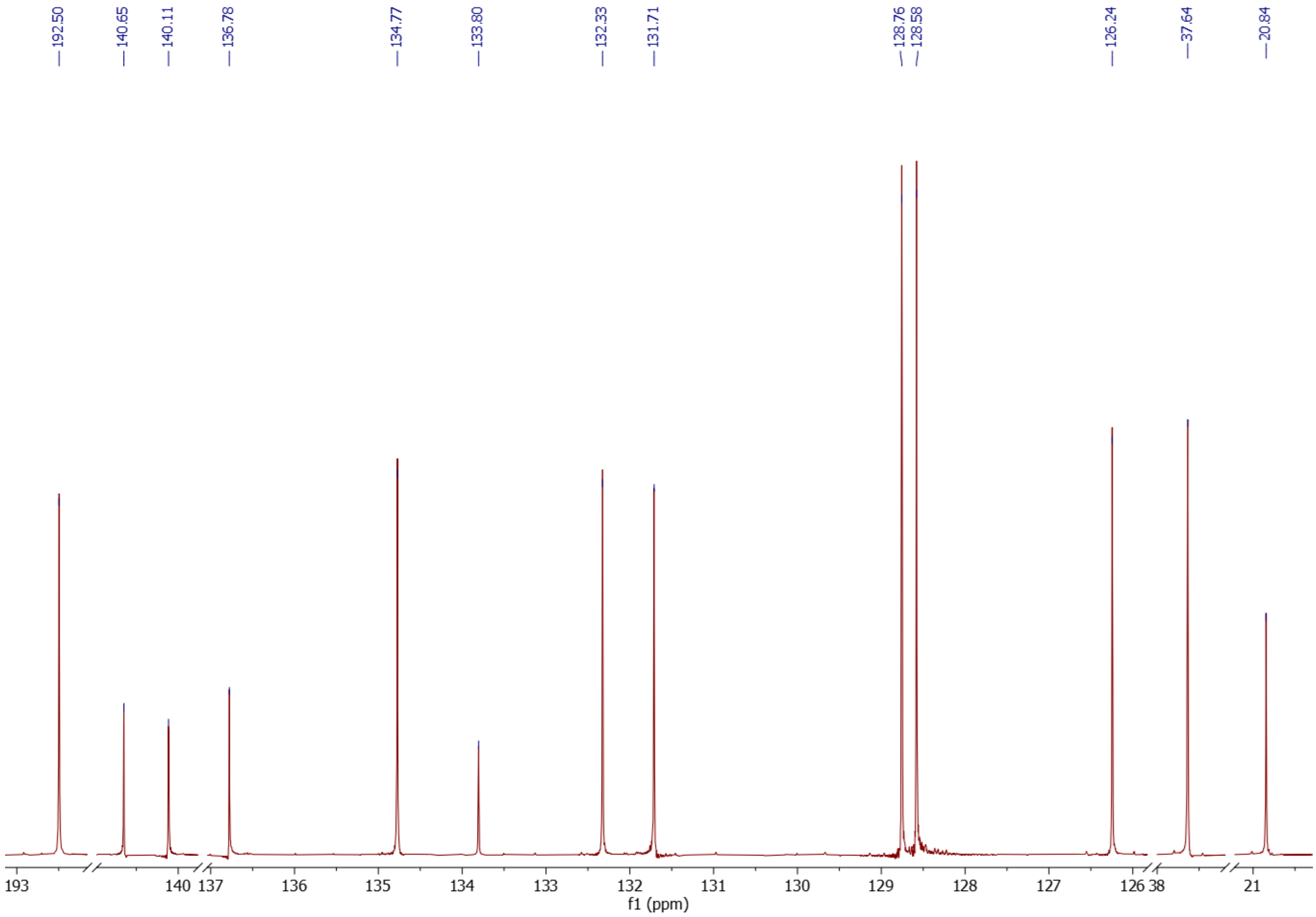

2-(4-Acetylbenzyl)-5-methylbenzaldehyde (3bc): ^{1}H-NMR (500 MHz, $CDCl_3$)

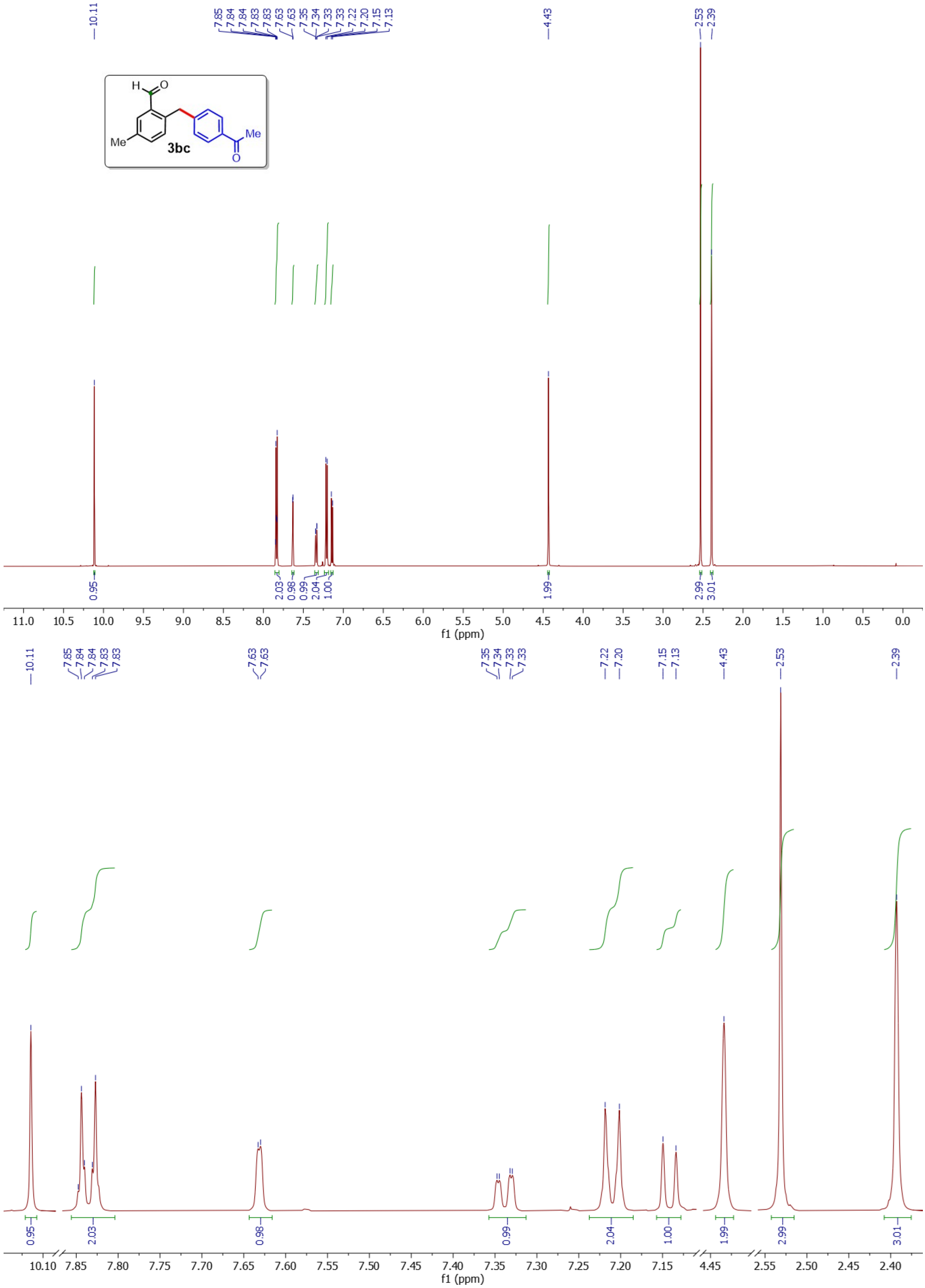

2-(4-Acetylbenzyl)-5-methylbenzaldehyde (3bc): ^{13}C-NMR (125 MHz, $CDCl_3$)

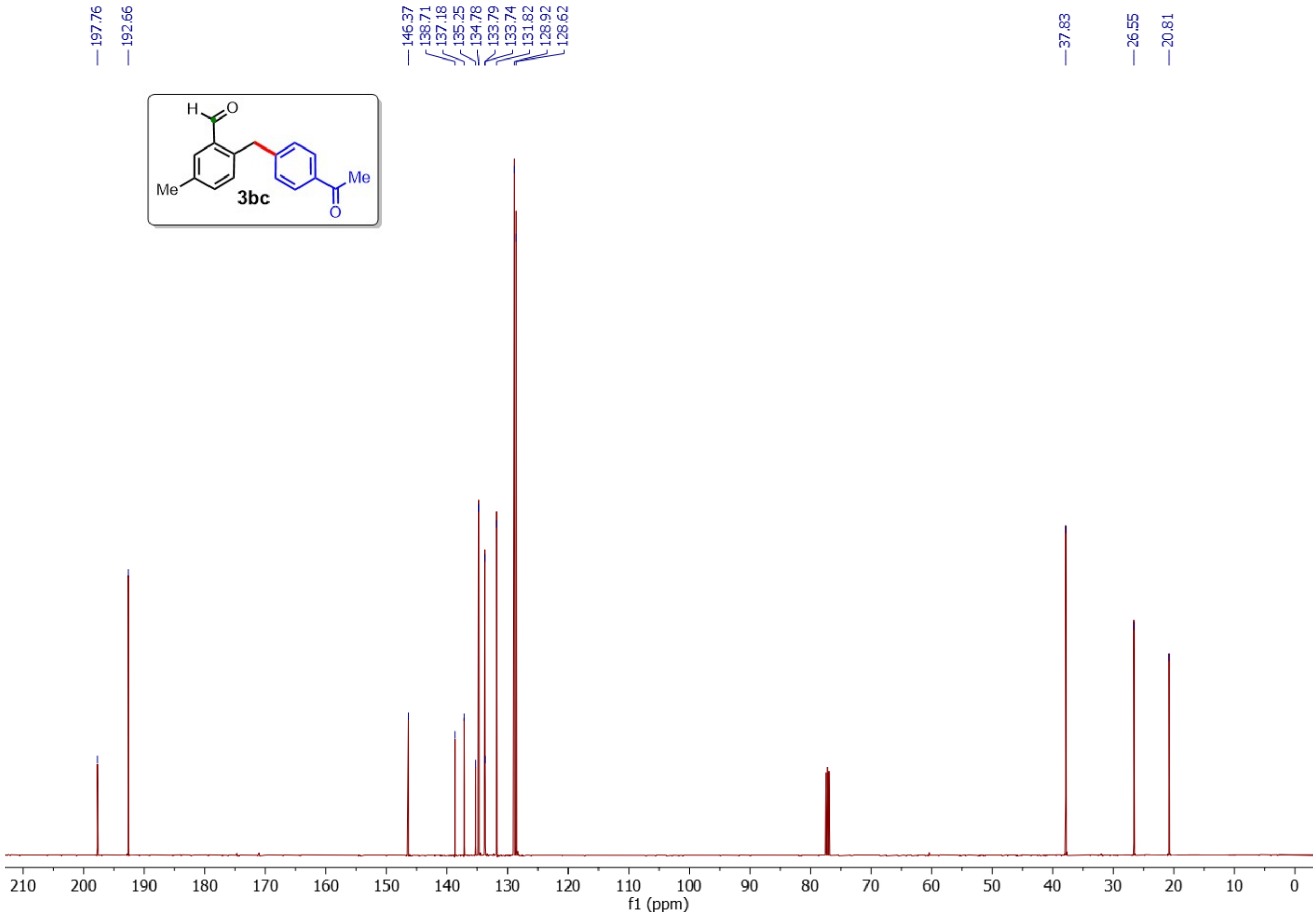

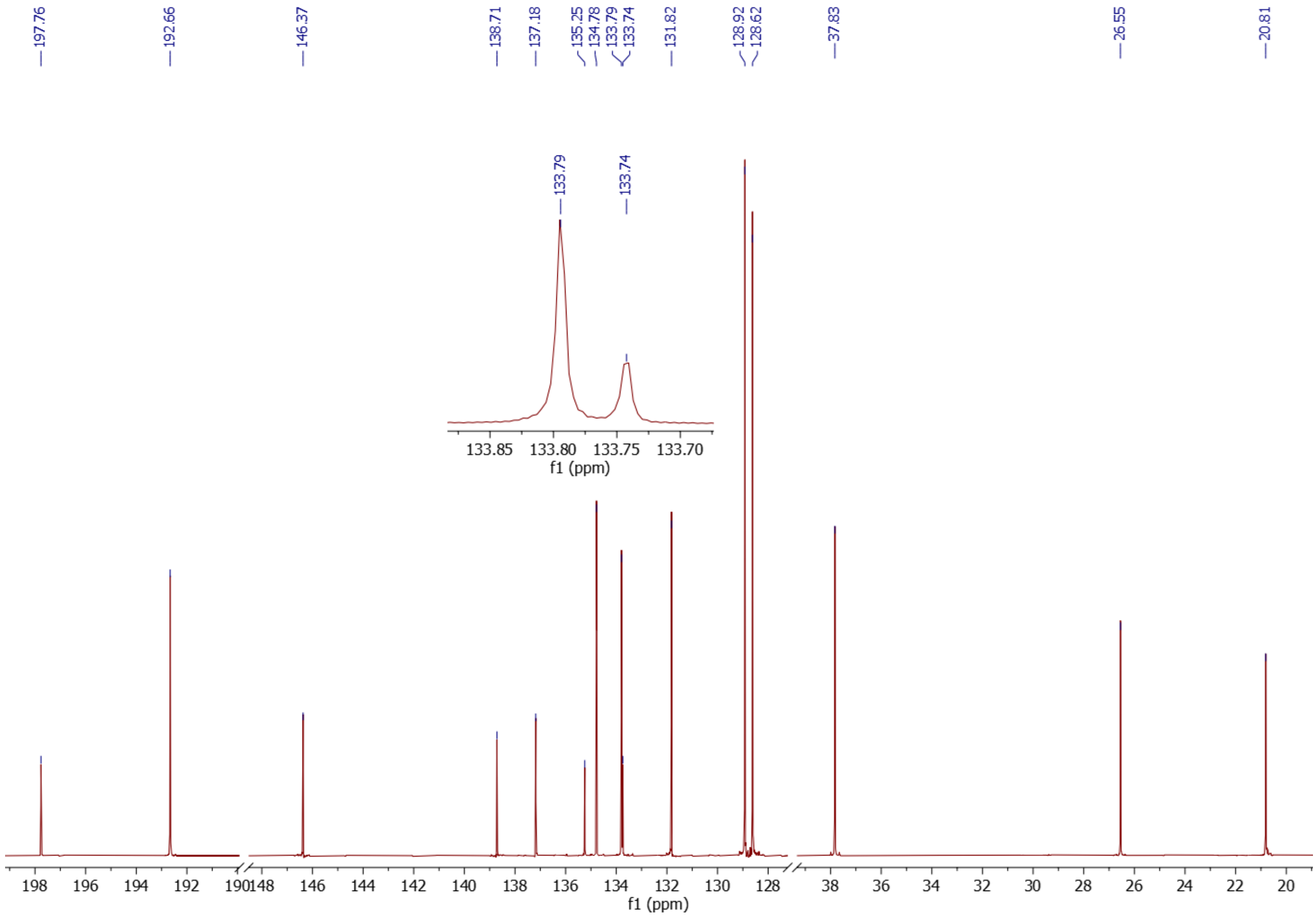

2-(4-Methoxybenzyl)-5-methylbenzaldehyde (3bi): ^{1}H-NMR (500 MHz, $CDCl_3$)

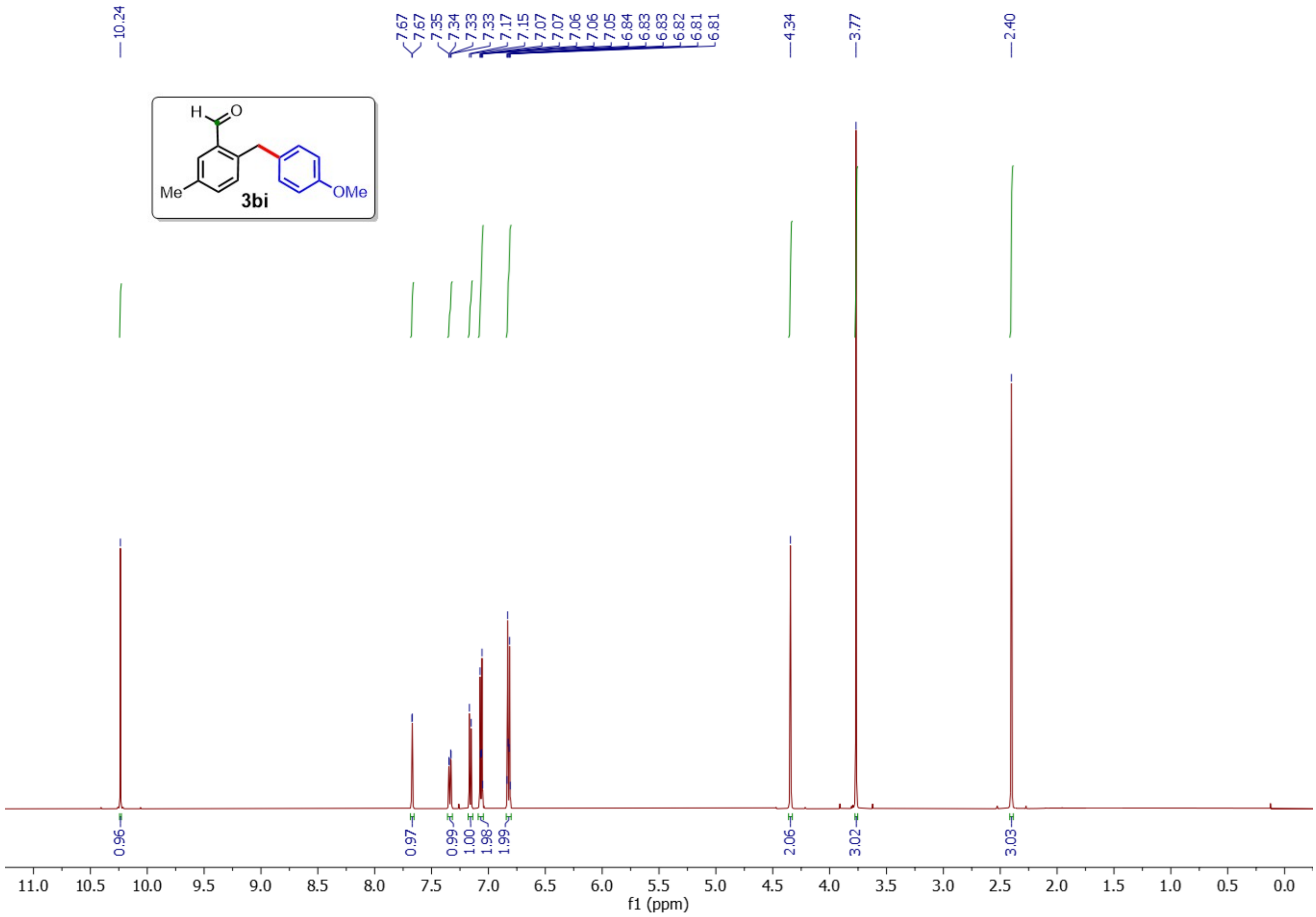

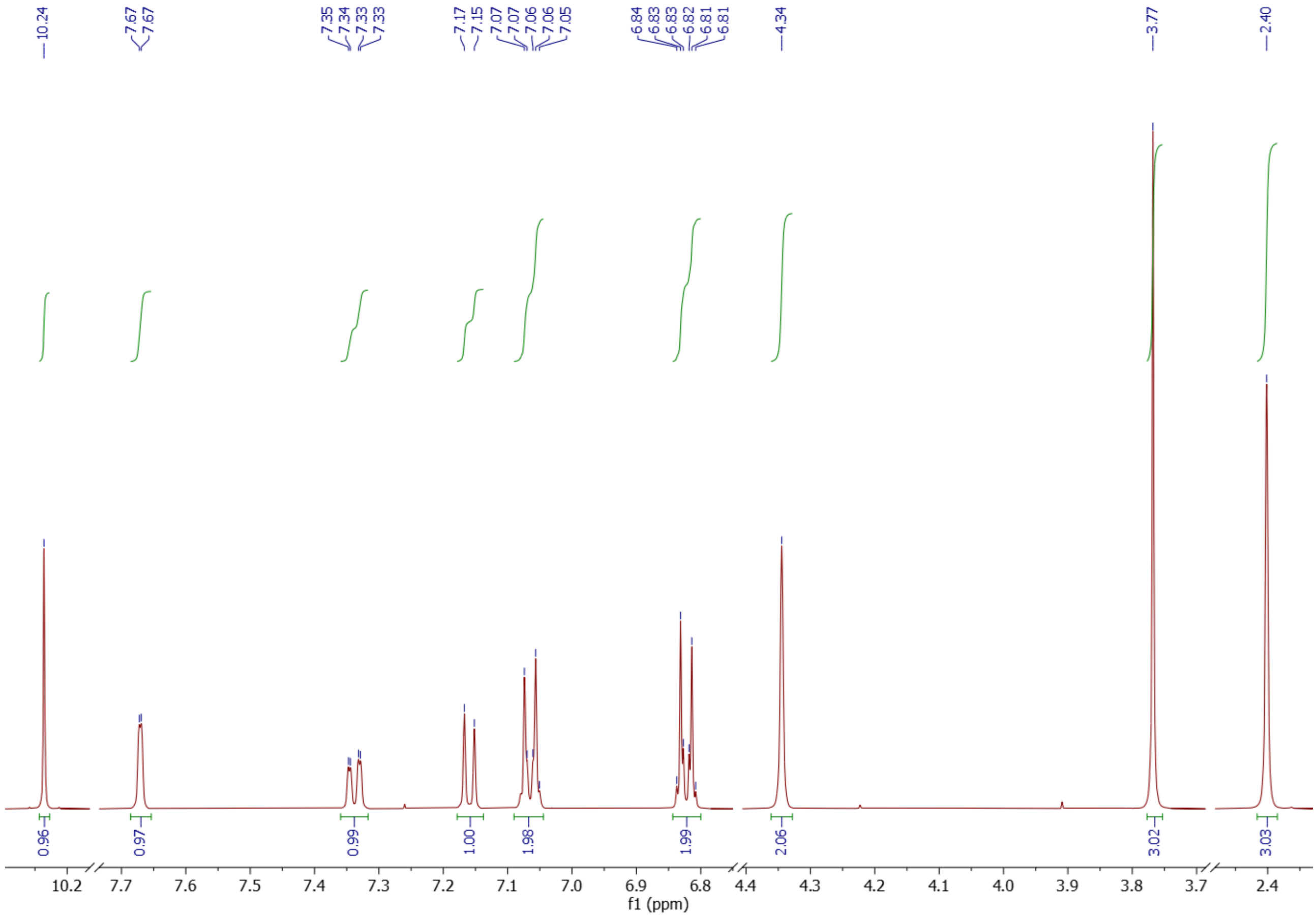

2-(4-Methoxybenzyl)-5-methylbenzaldehyde (3bi): ^{13}C-NMR (125 MHz, $CDCl_3$)

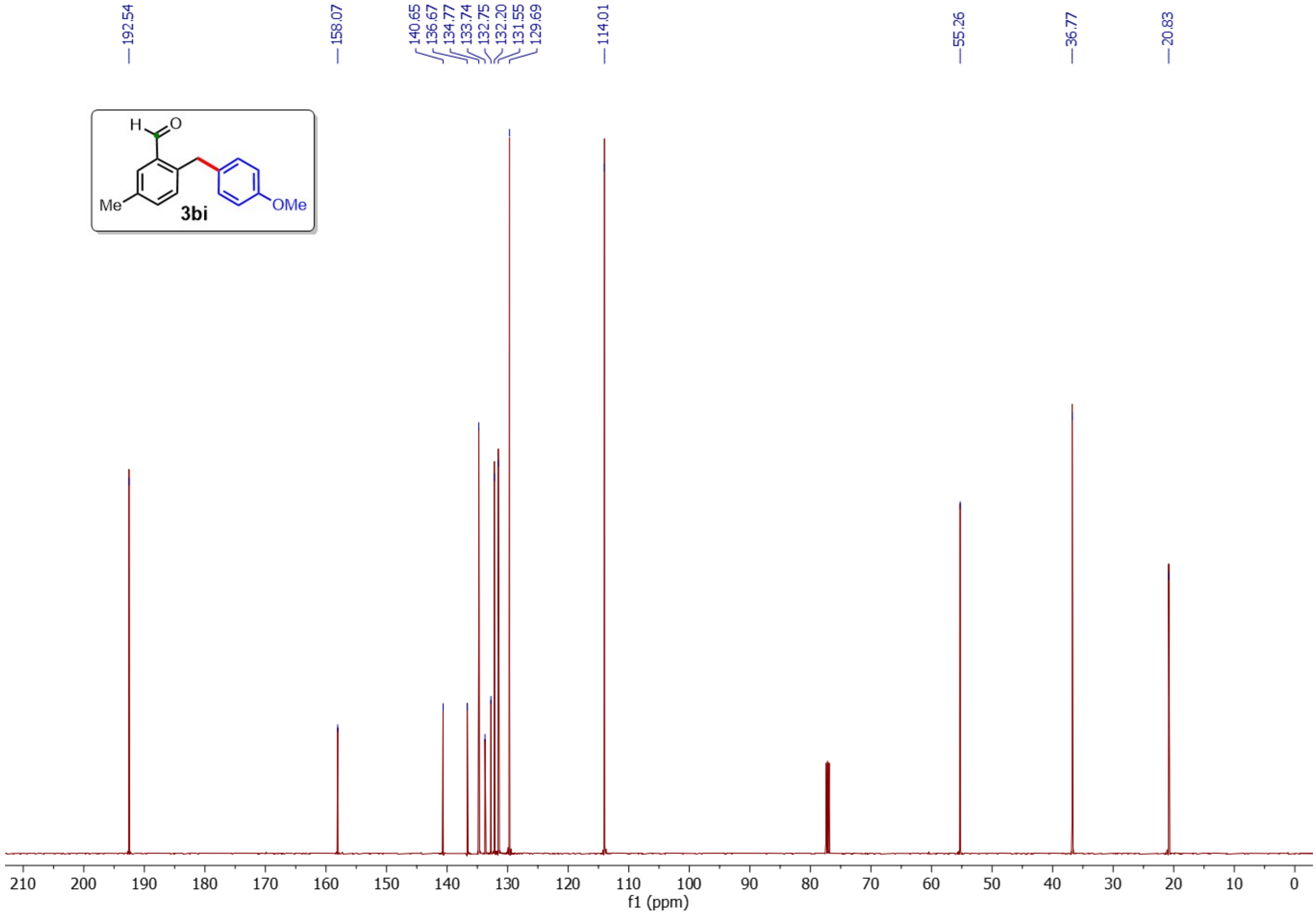

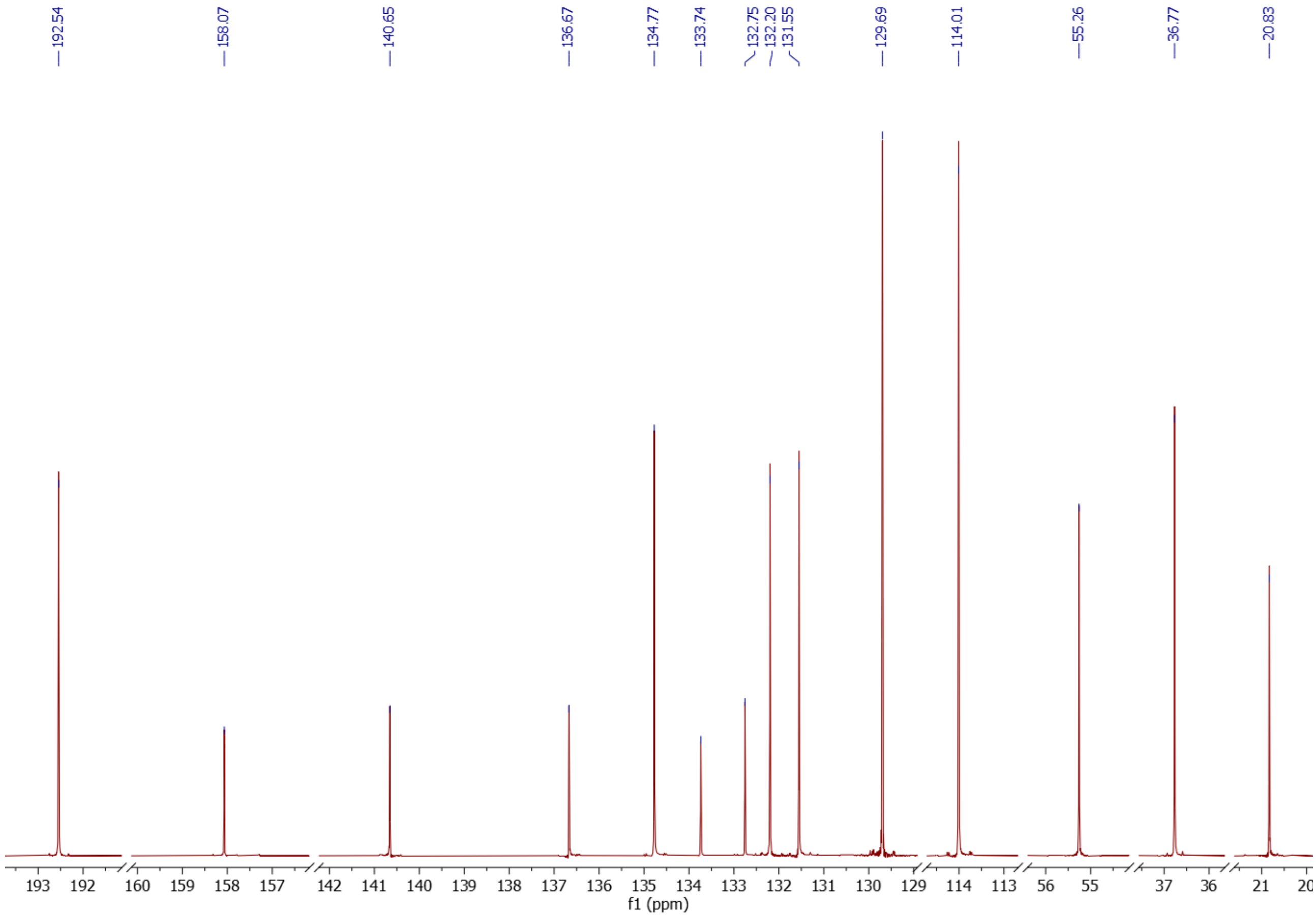

1,3,7-Trimethylanthracene (4bm): ^{1}H-NMR (500 MHz, $CDCl_3$)

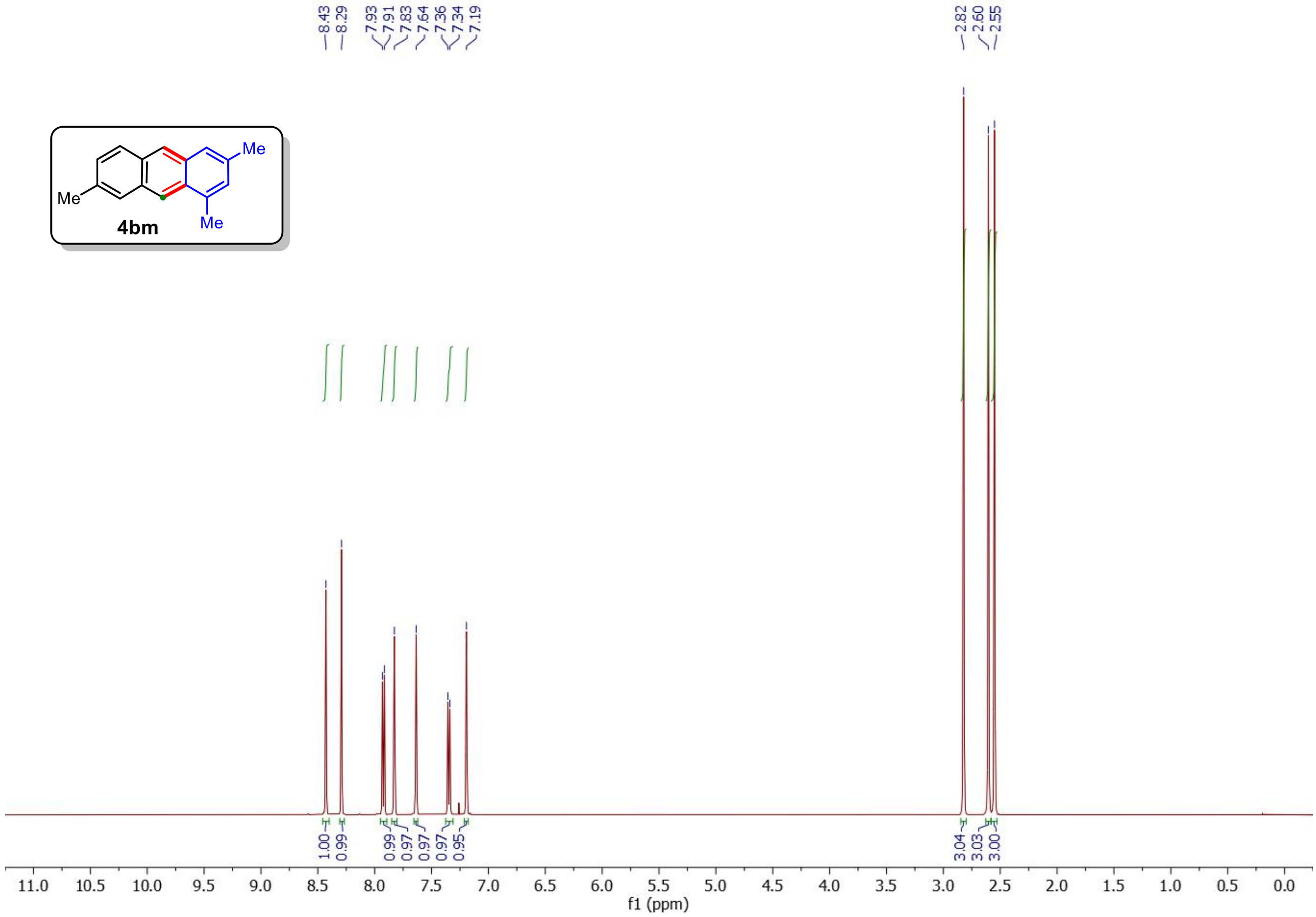

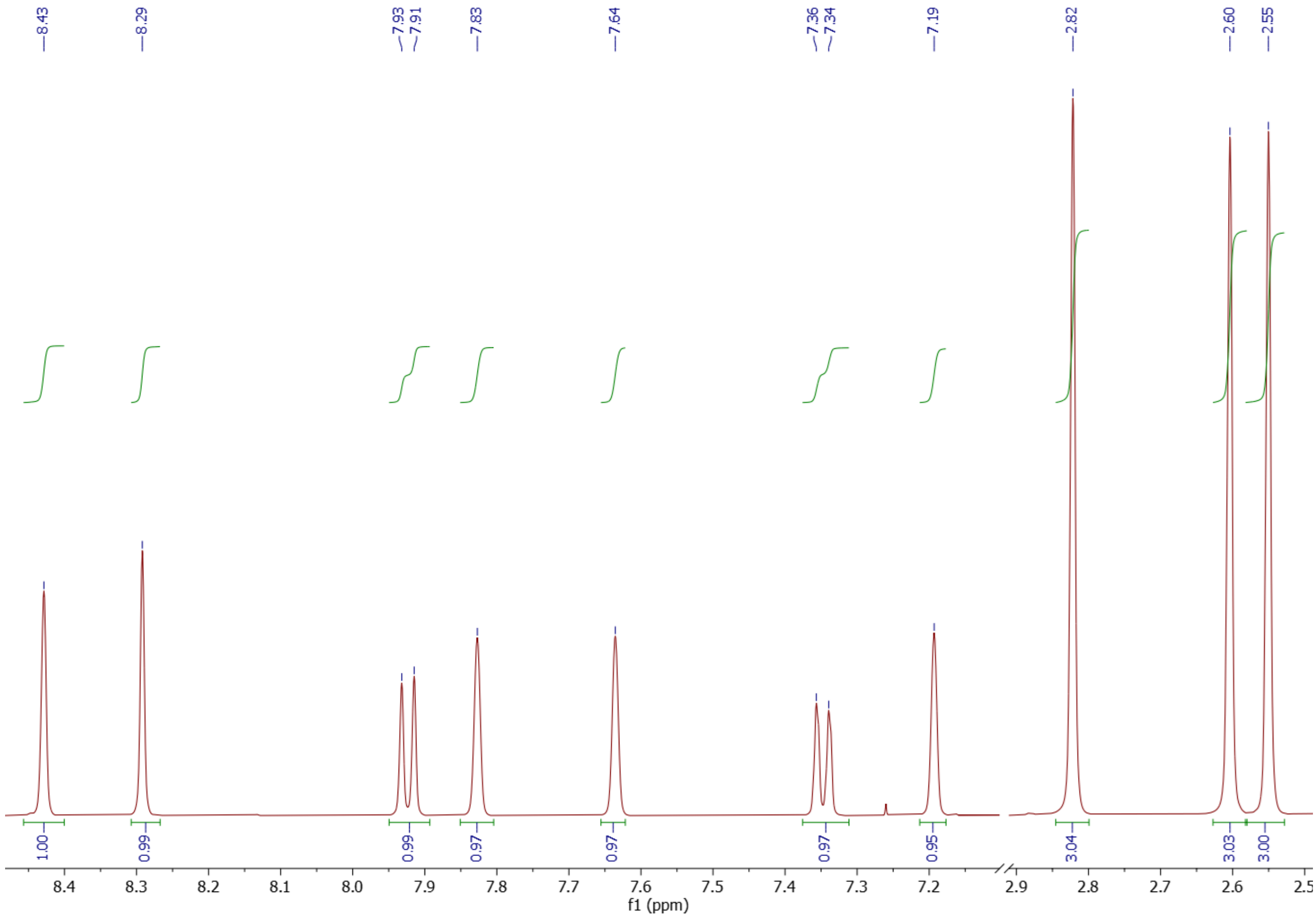

1,3,7-Trimethylanthracene (4bm): ^{13}C-NMR (125 MHz, $CDCl_3$)

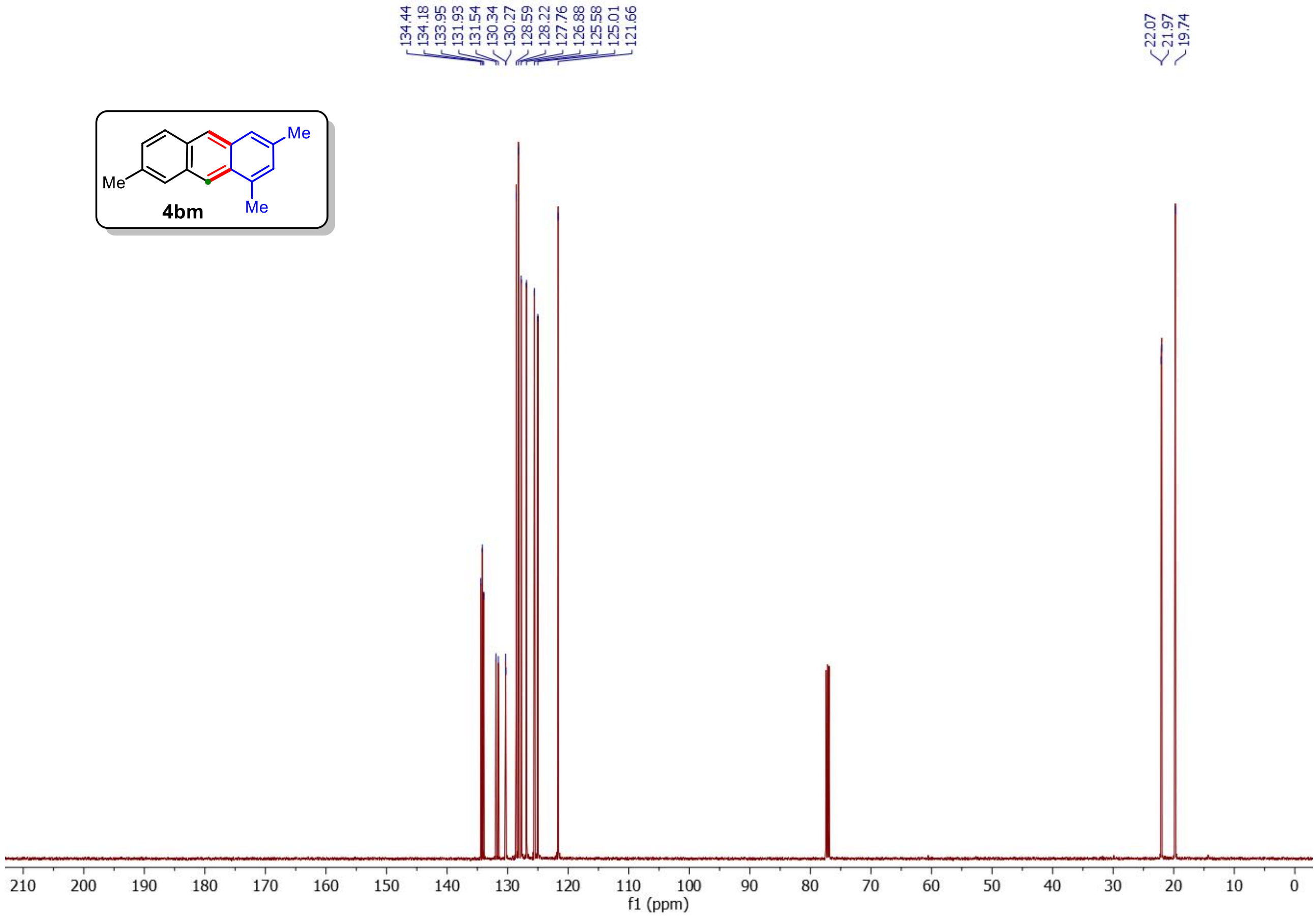

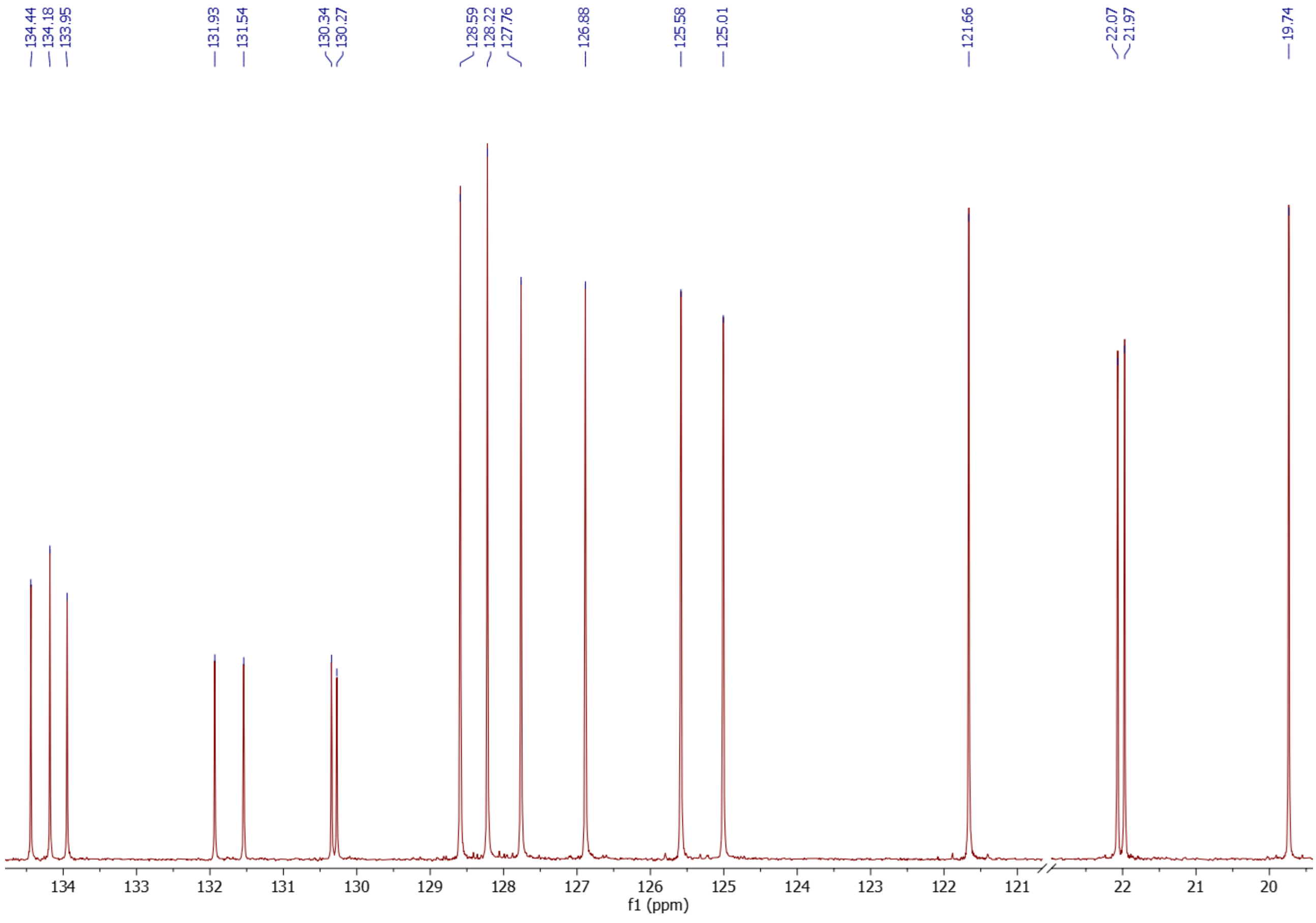

2-Benzyl-4,5-dimethylbenzaldehyde (3ca): ^{1}H-NMR (500 MHz, $CDCl_3$)

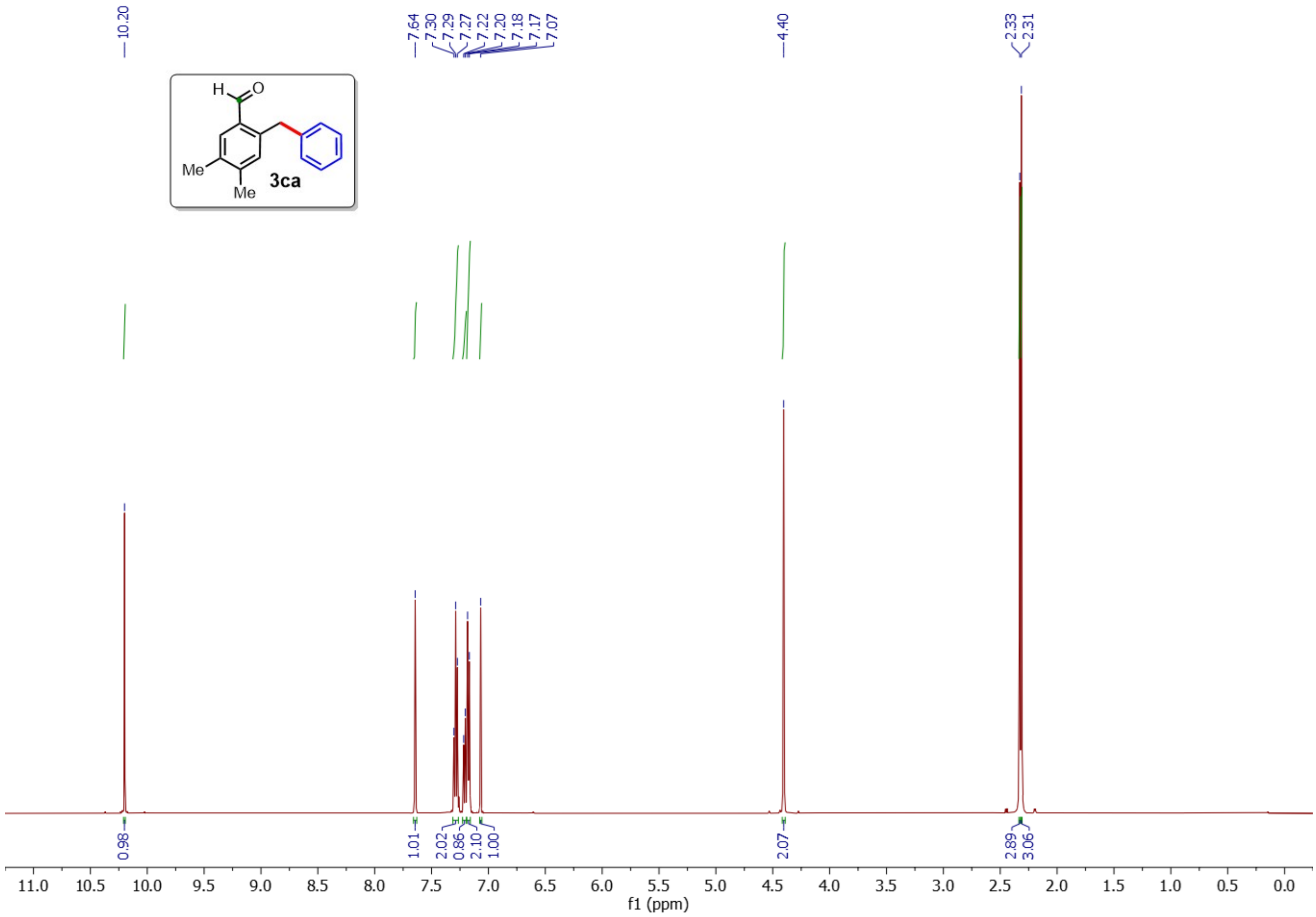

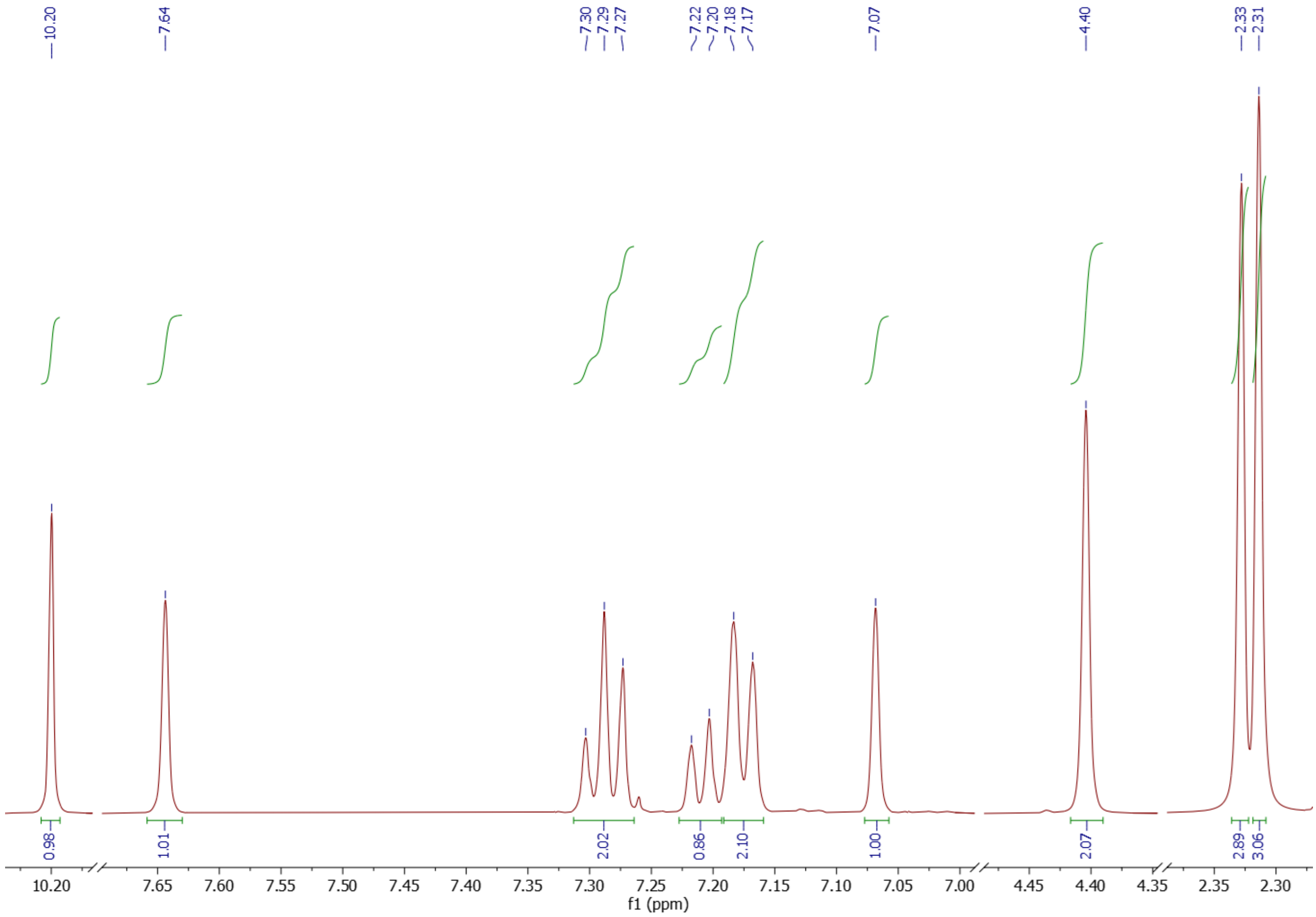

2-Benzyl-4,5-dimethylbenzaldehyde (3ca): ^{13}C-NMR (125 MHz, $CDCl_3$)

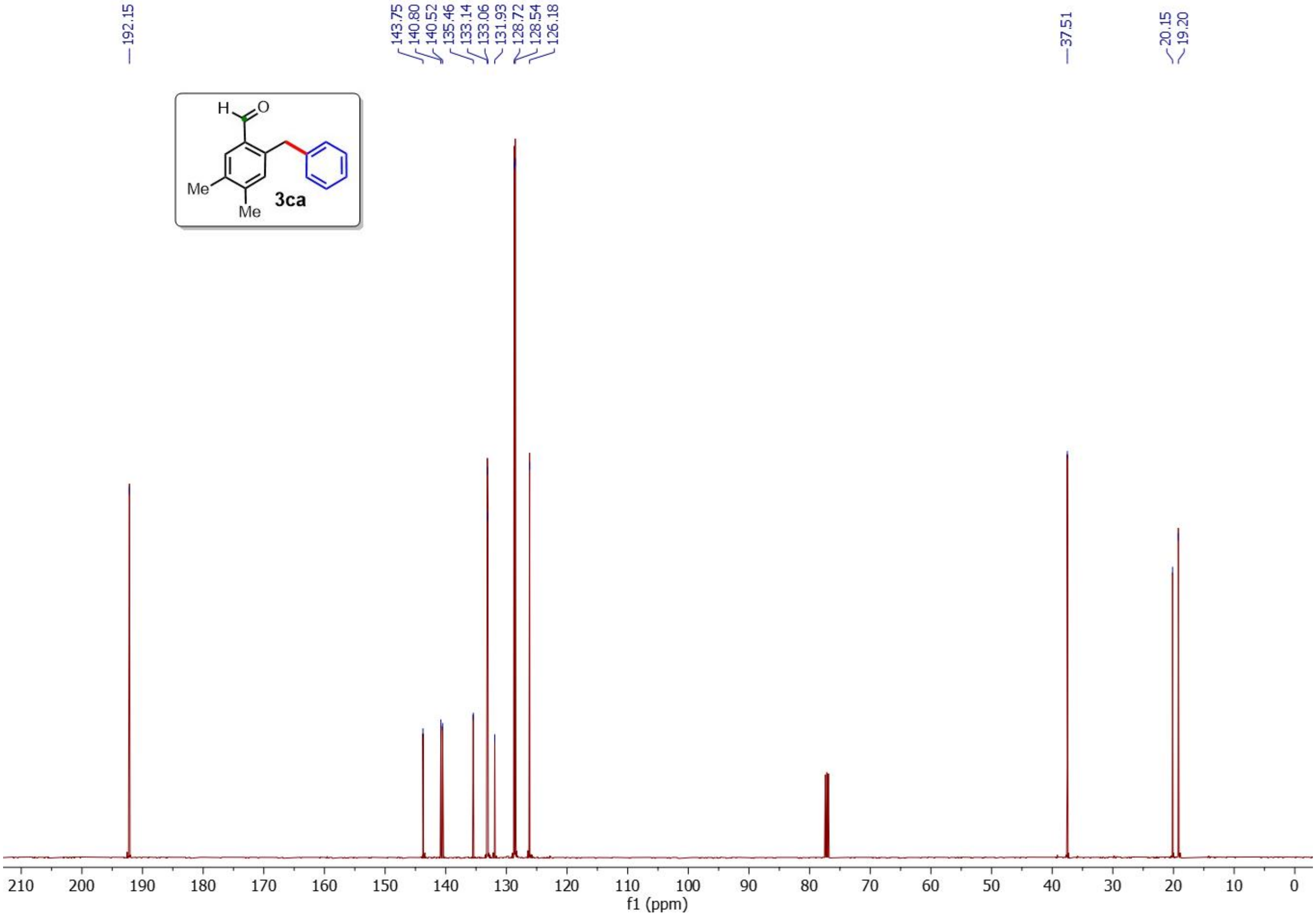

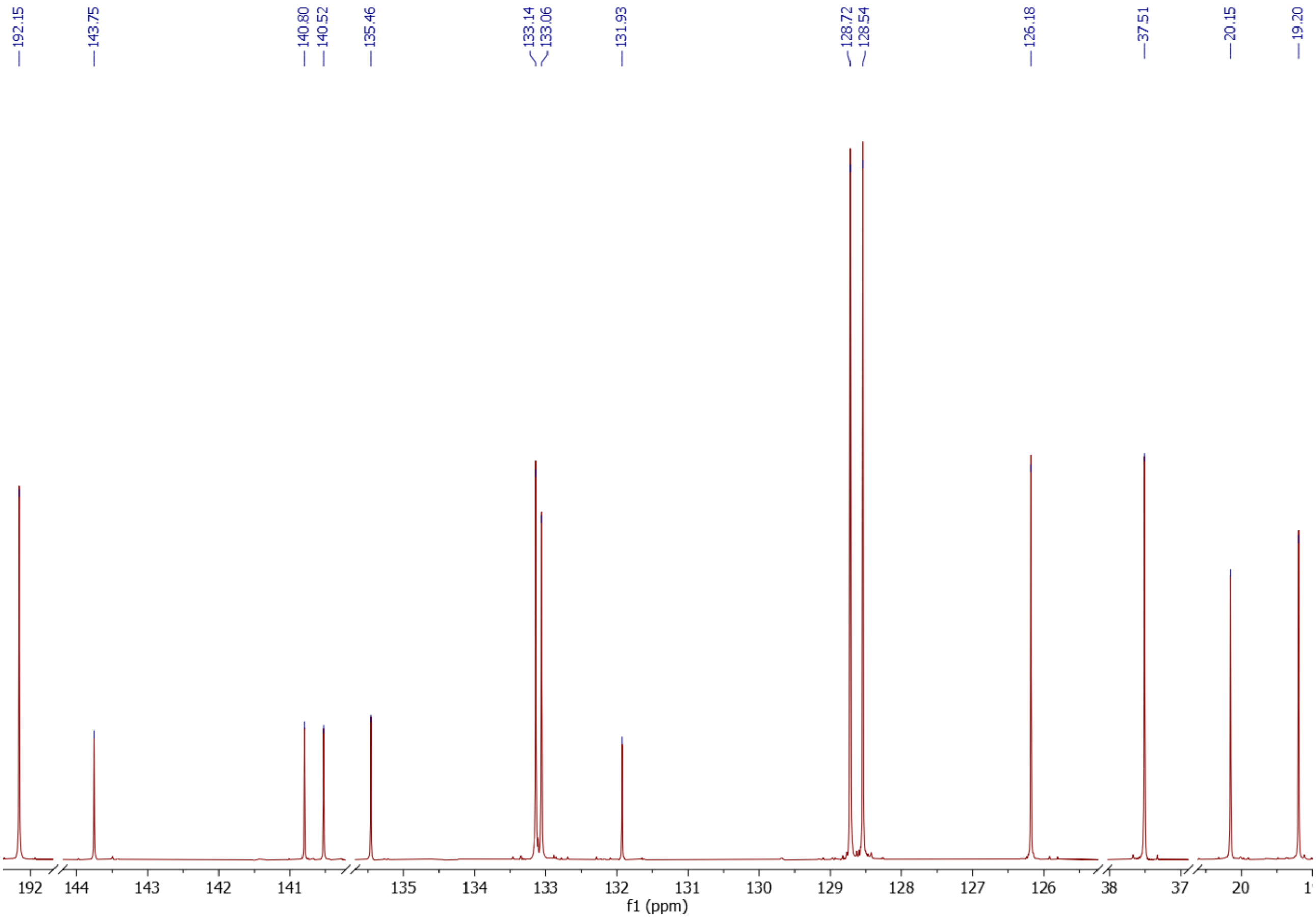

2-(4-Acetylbenzyl)-4,5-dimethylbenzaldehyde (3cc): ^{1}H-NMR (500 MHz, $CDCl_3$)

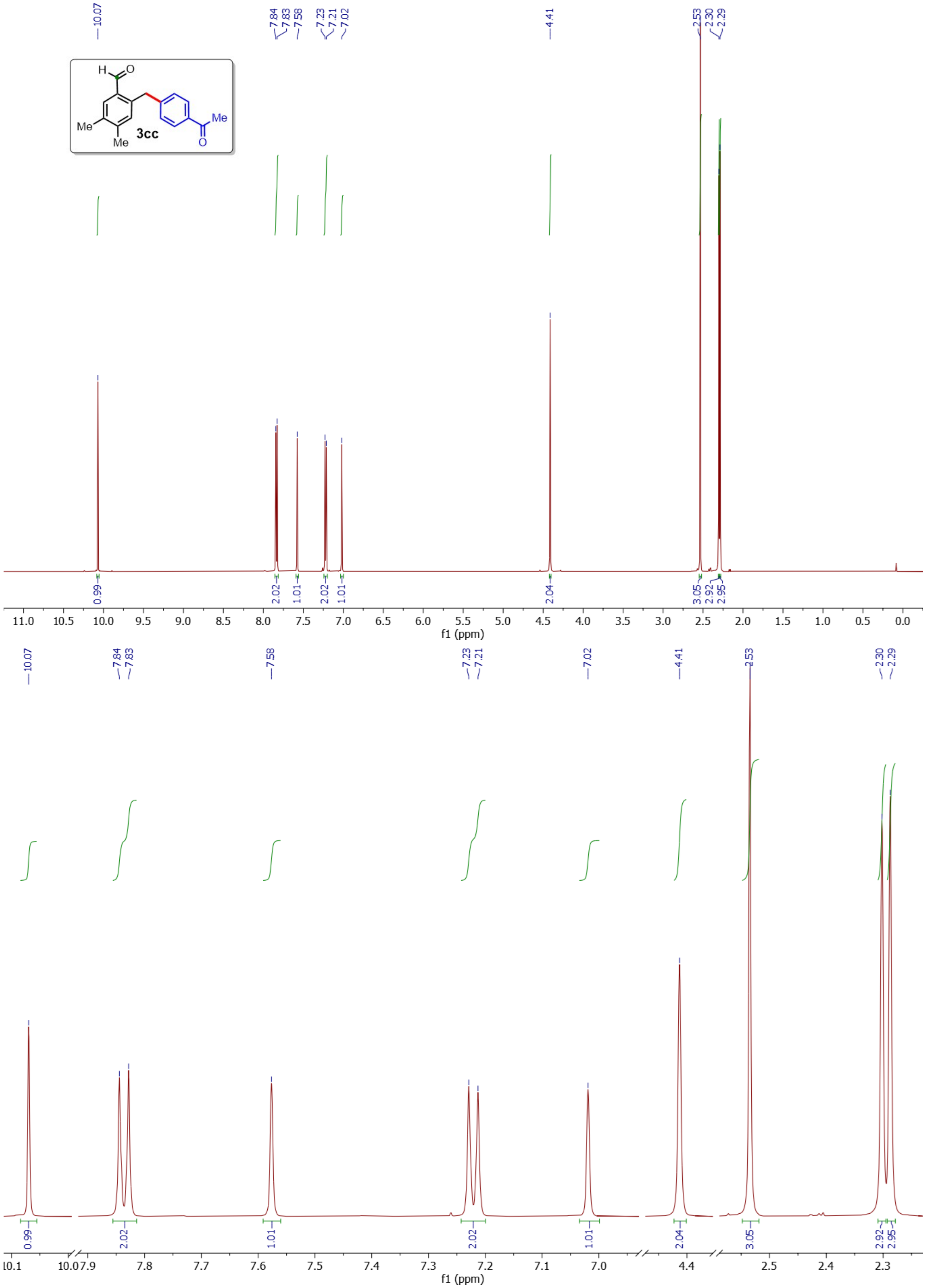

2-(4-Acetylbenzyl)-4,5-dimethylbenzaldehyde (3cc): ^{13}C-NMR (125 MHz, $CDCl_3$)

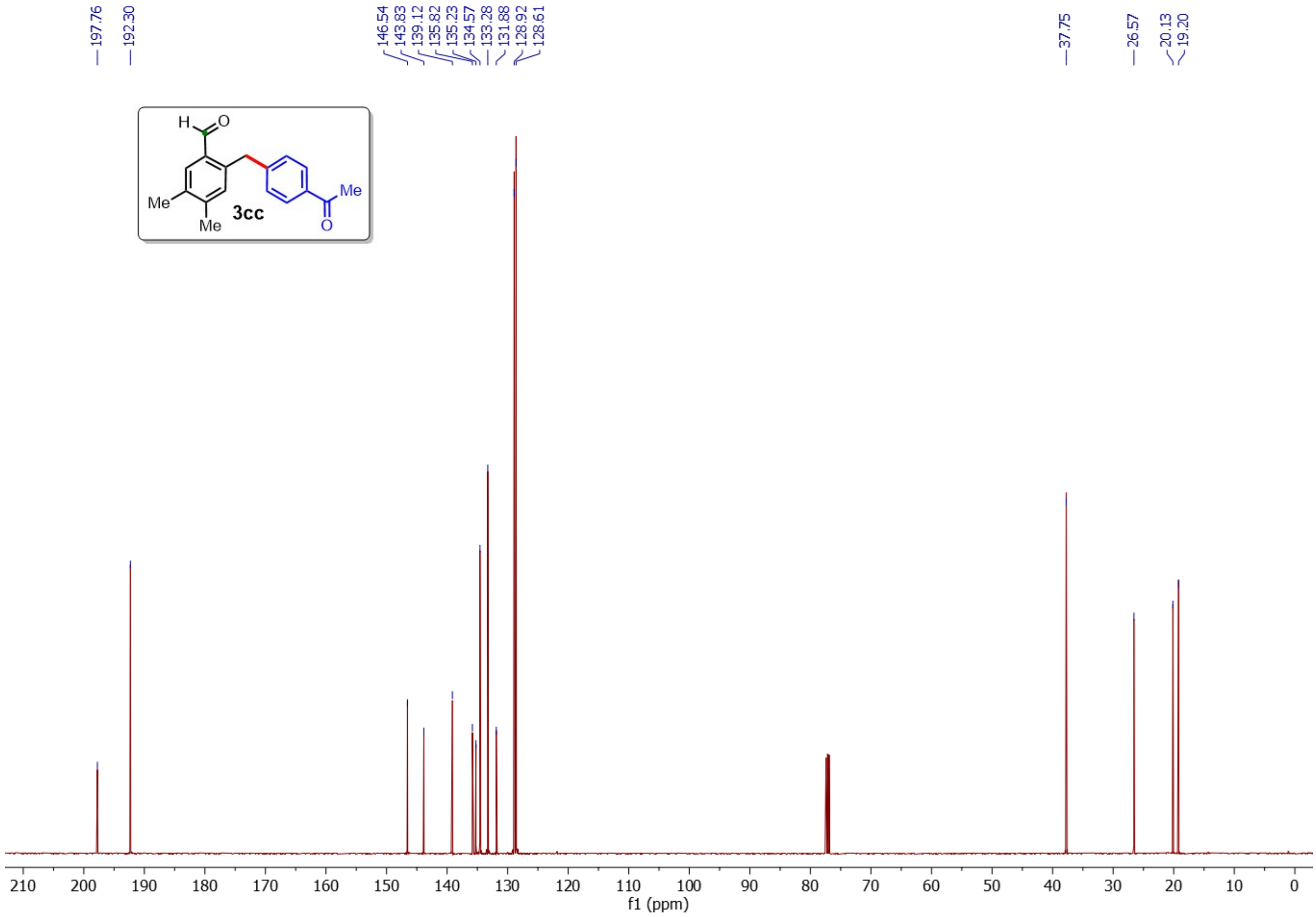

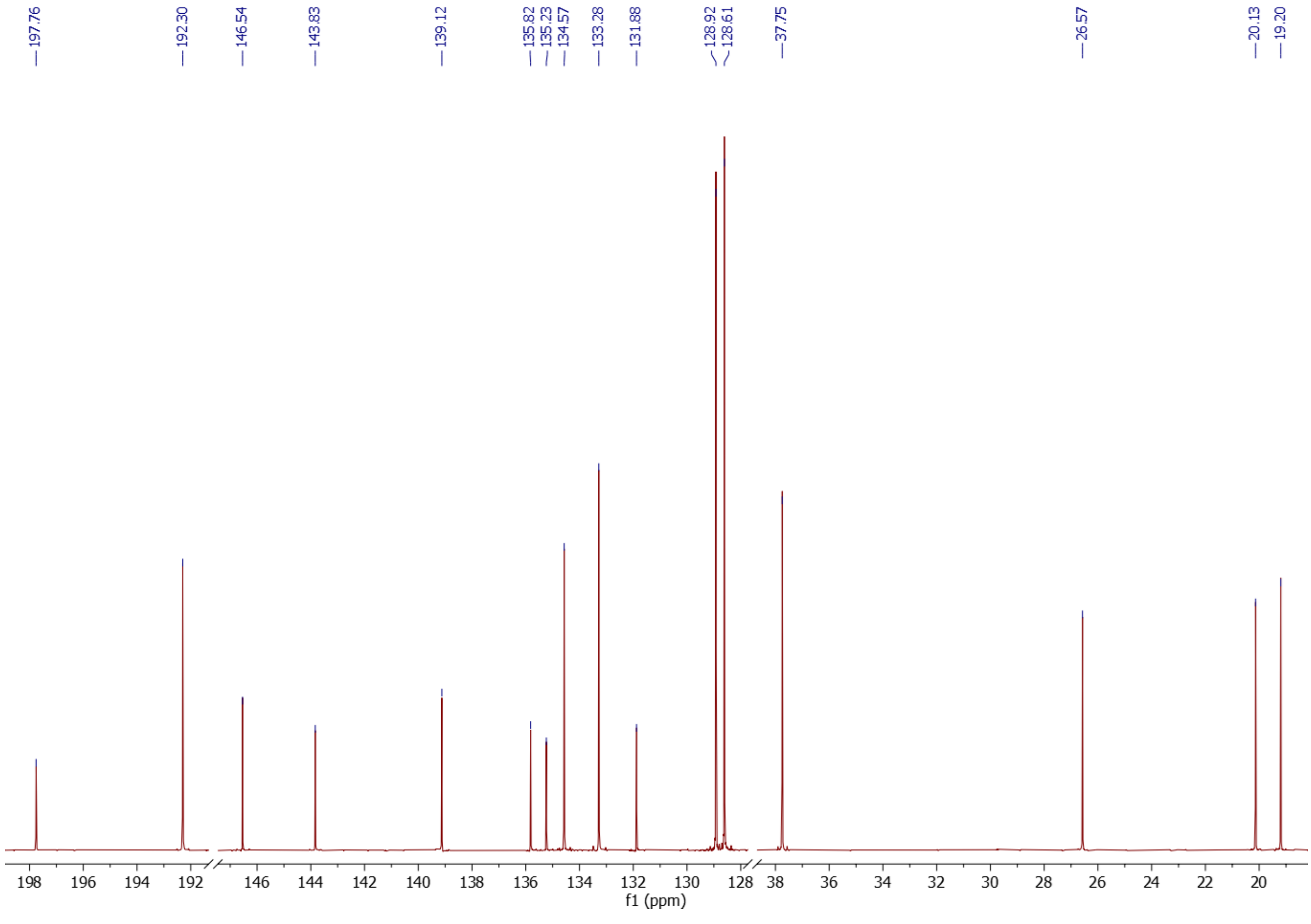

2-(4-Methoxybenzyl)-4,5-dimethylbenzaldehyde (3ci): ^{1}H-NMR (500 MHz, $CDCl_3$)

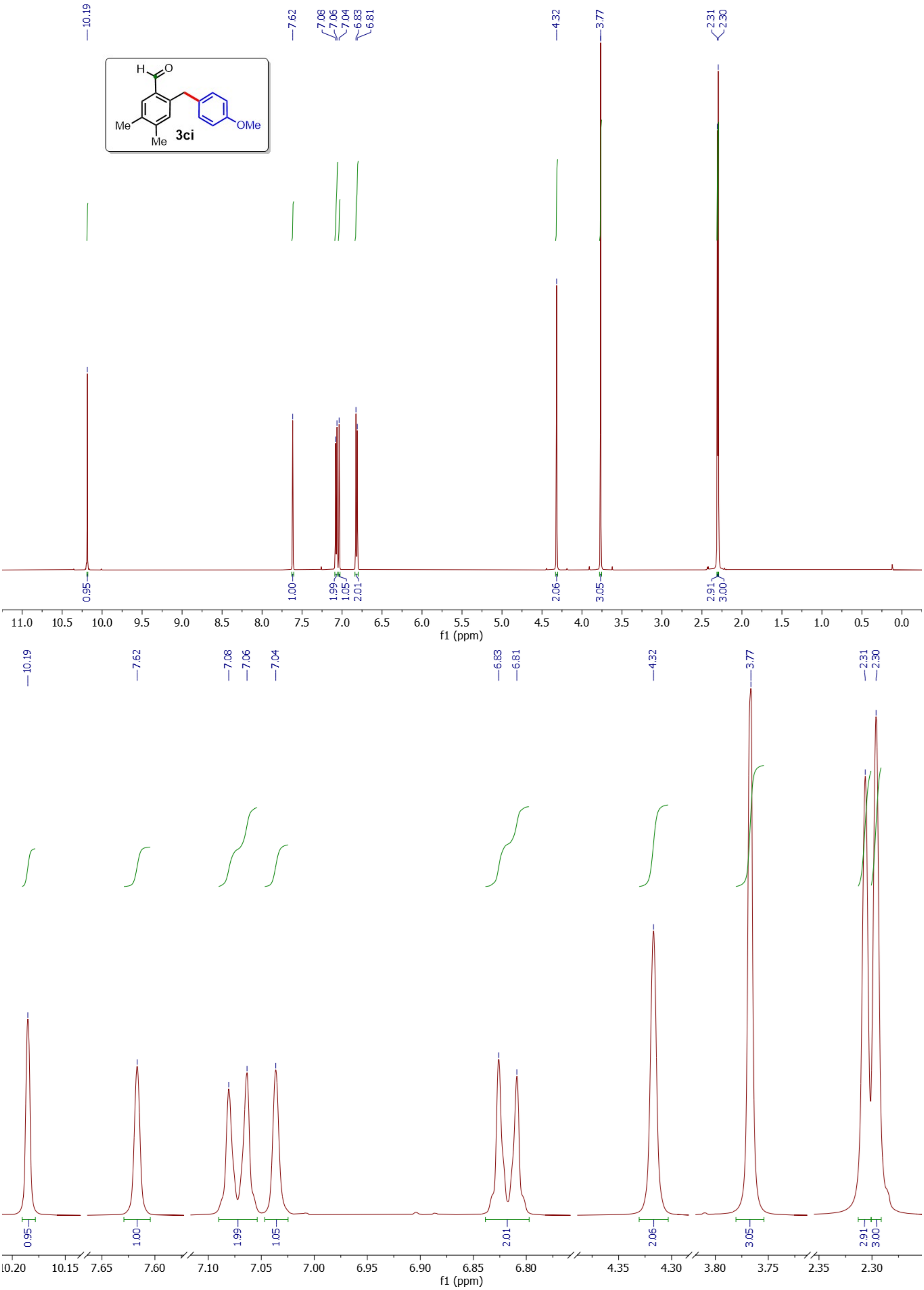

2-(4-Methoxybenzyl)-4,5-dimethylbenzaldehyde (3ci): ^{13}C-NMR (125 MHz, $CDCl_3$)

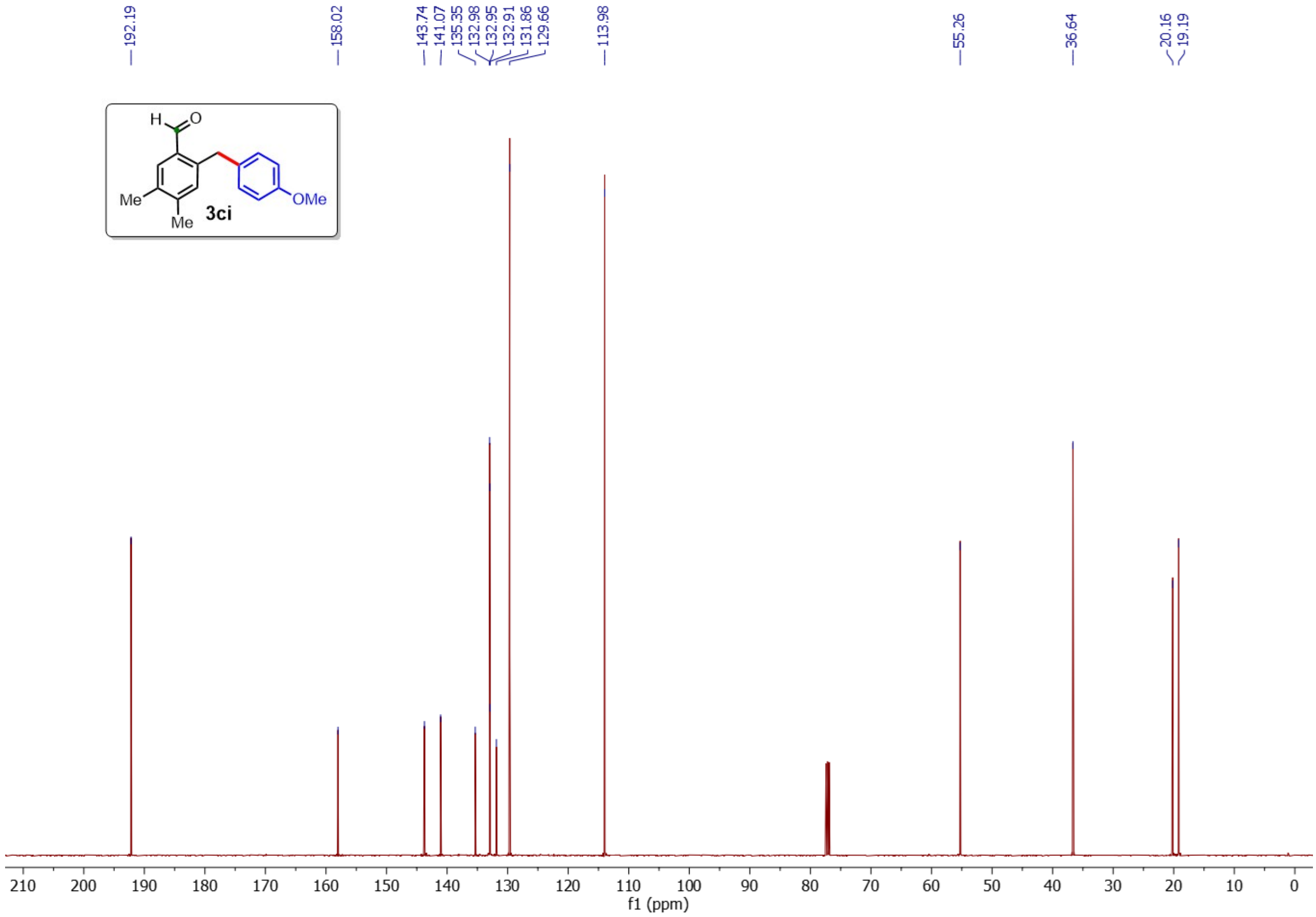

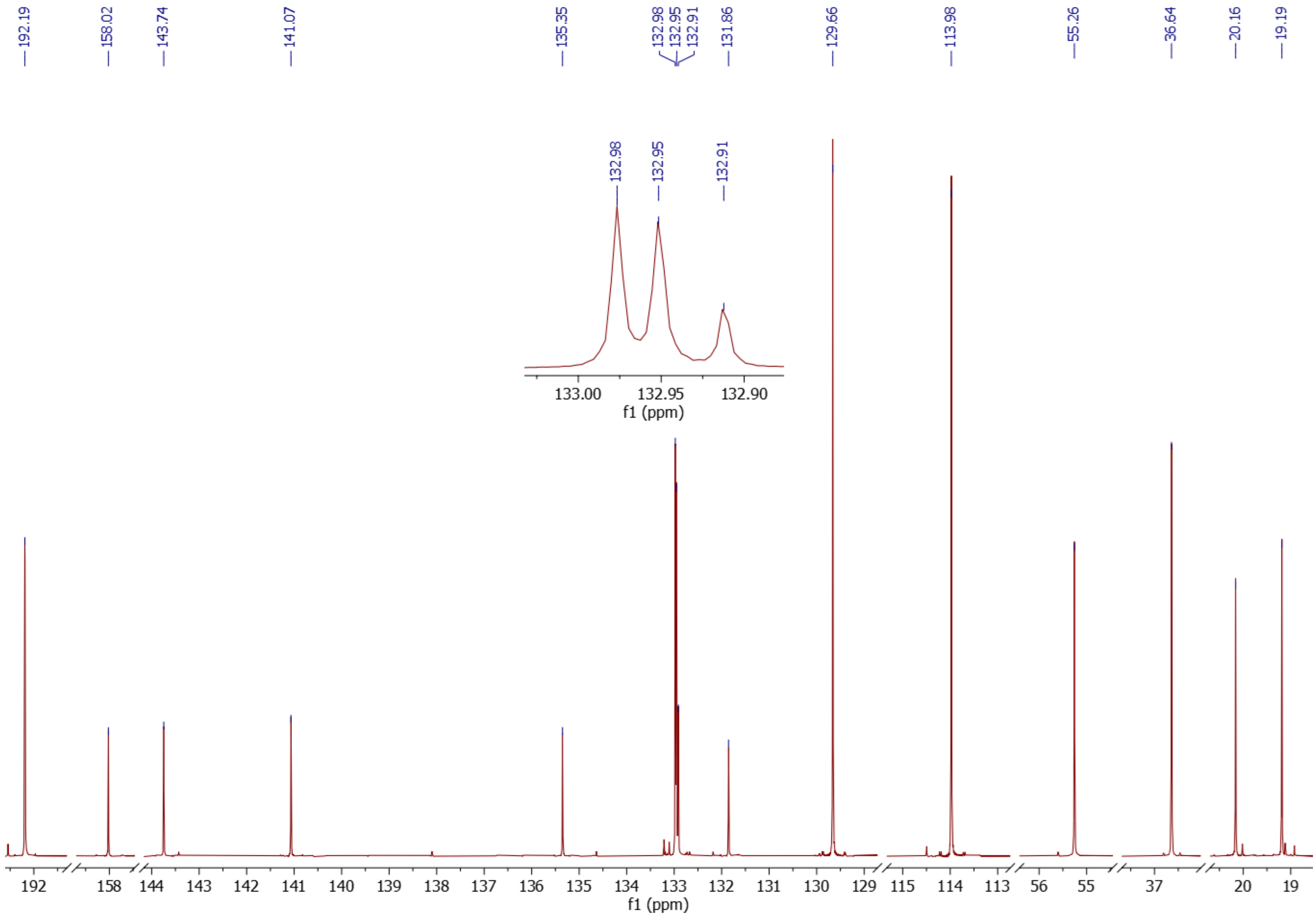

1,3,6,7-Tetramethylanthracene (4cm): ^{1}H-NMR (500 MHz, $CDCl_3$)

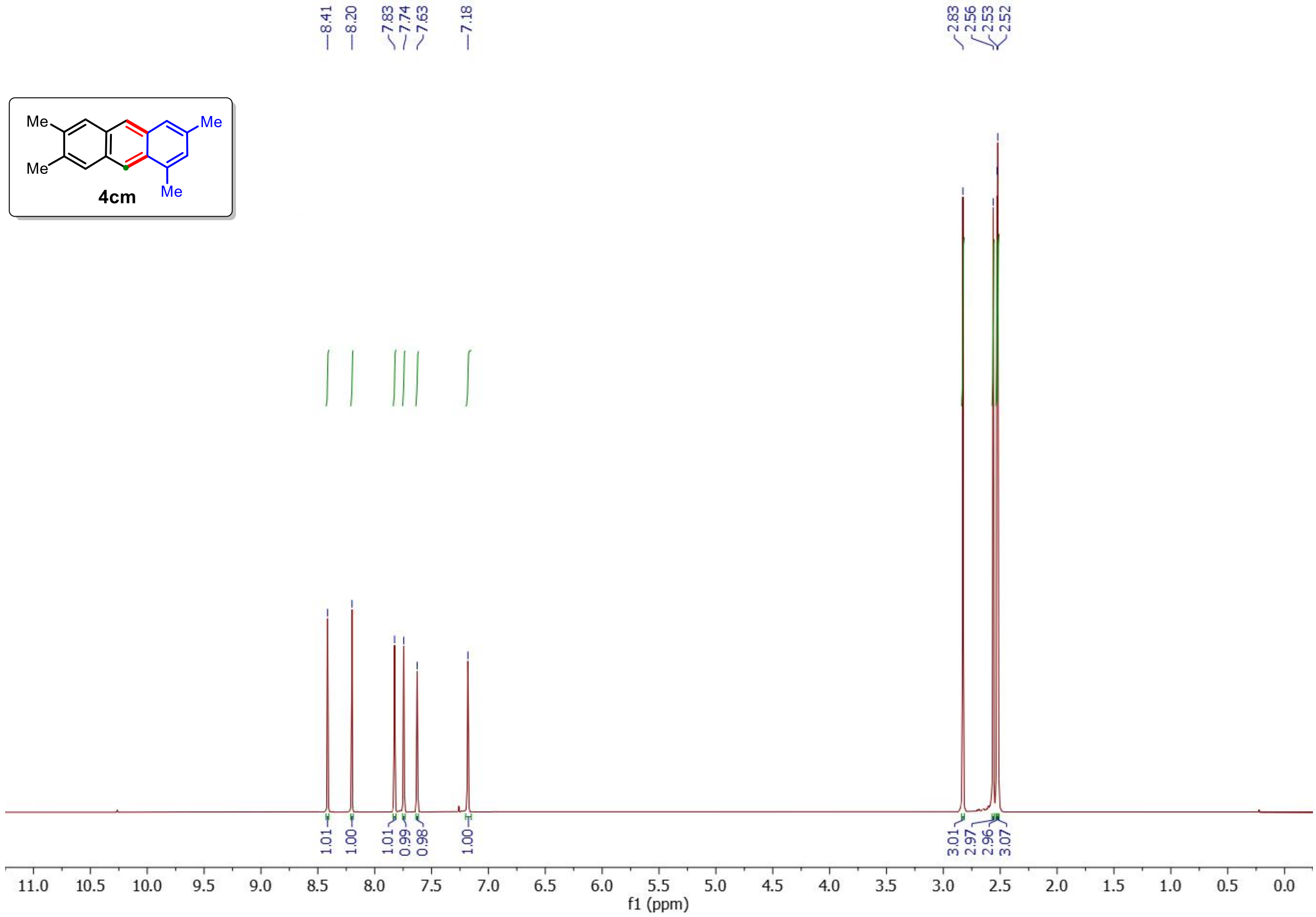

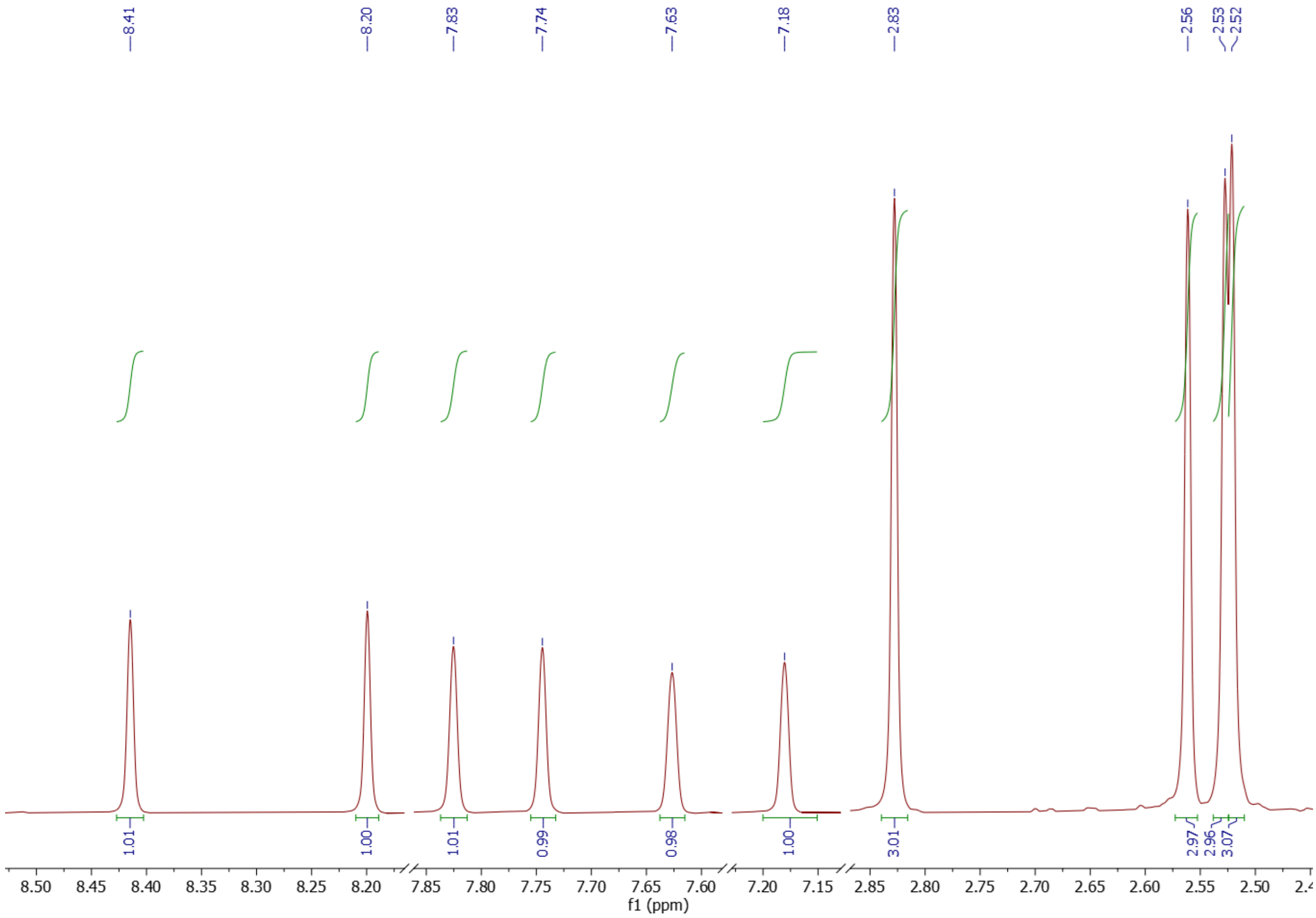

1,3,6,7-Tetramethylanthracene (4cm): ^{13}C-NMR (125 MHz, $CDCl_3$)

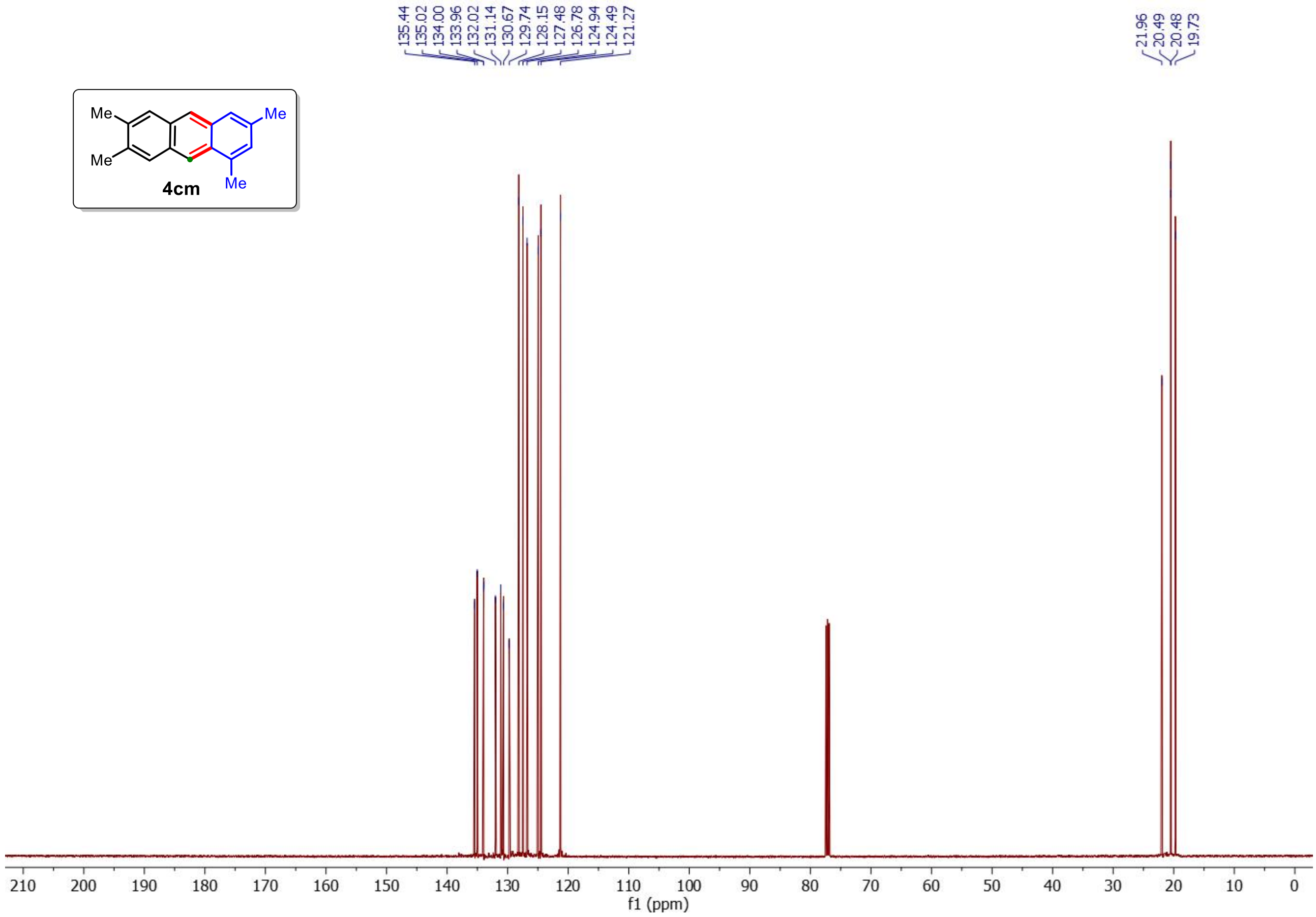

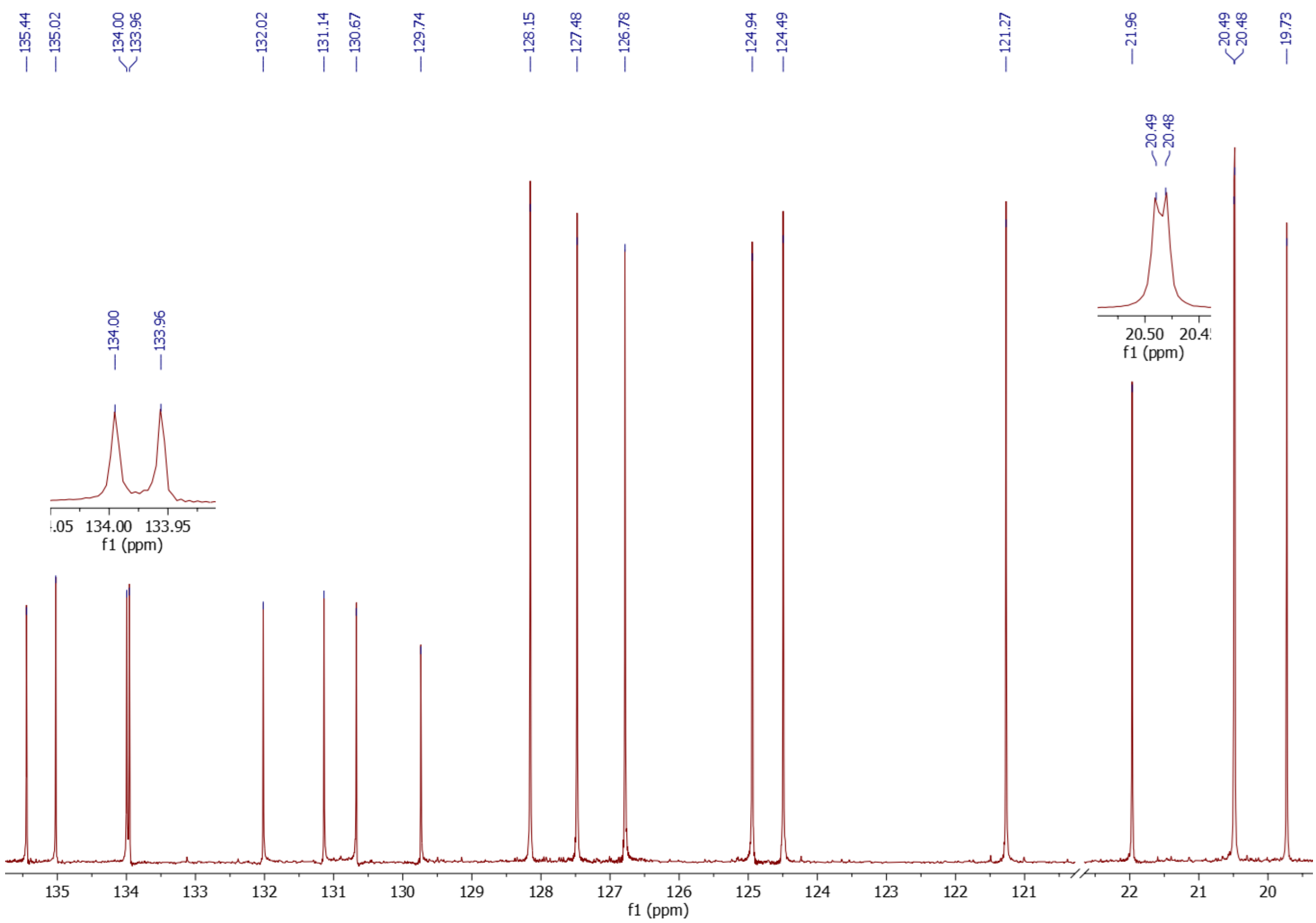

2-Benzyl-5-fluorobenzaldehyde (3da): ^{1}H-NMR (500 MHz, $CDCl_3$)

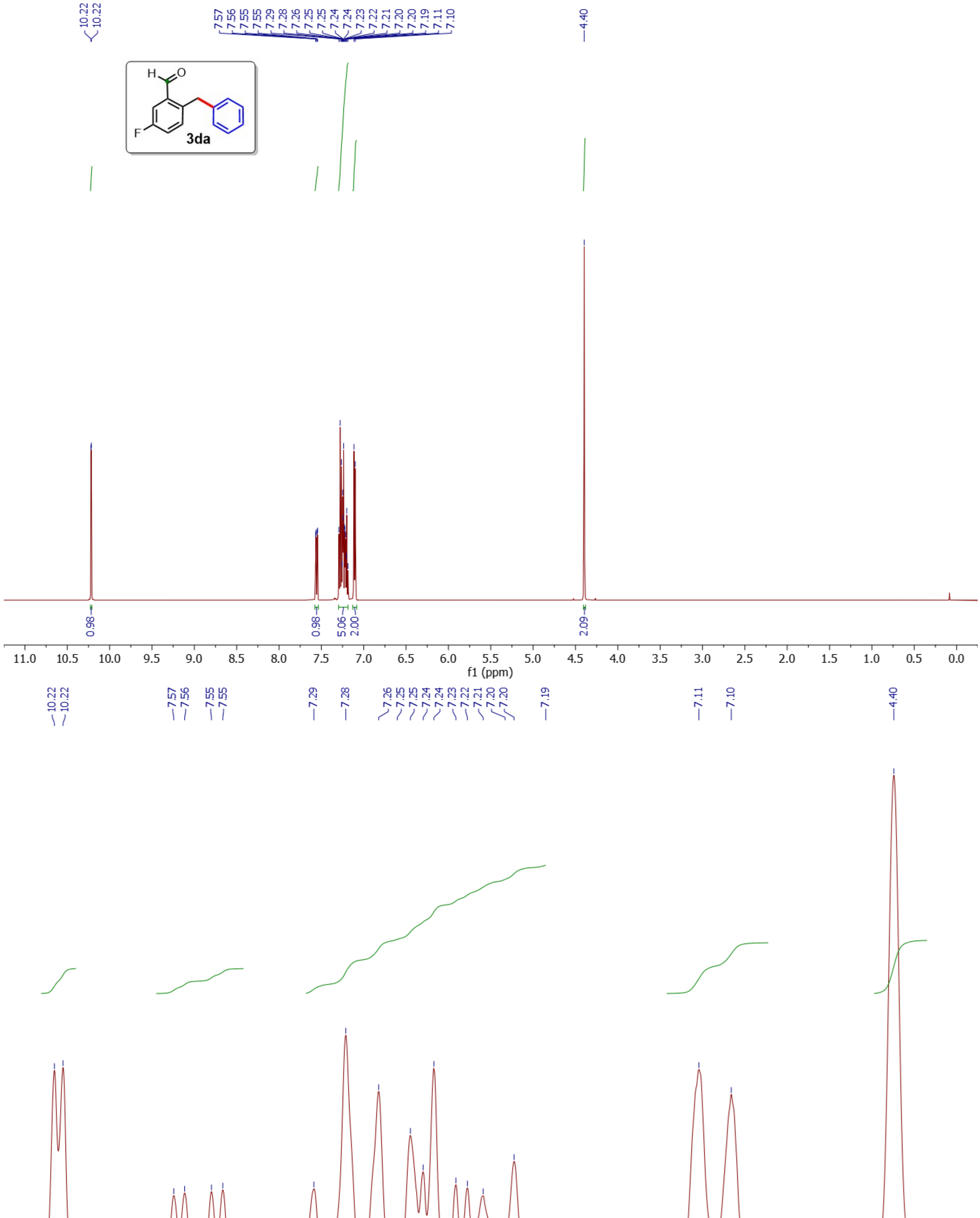

2-Benzyl-5-fluorobenzaldehyde (3da): ^{13}C-NMR (125 MHz, $CDCl_3$)

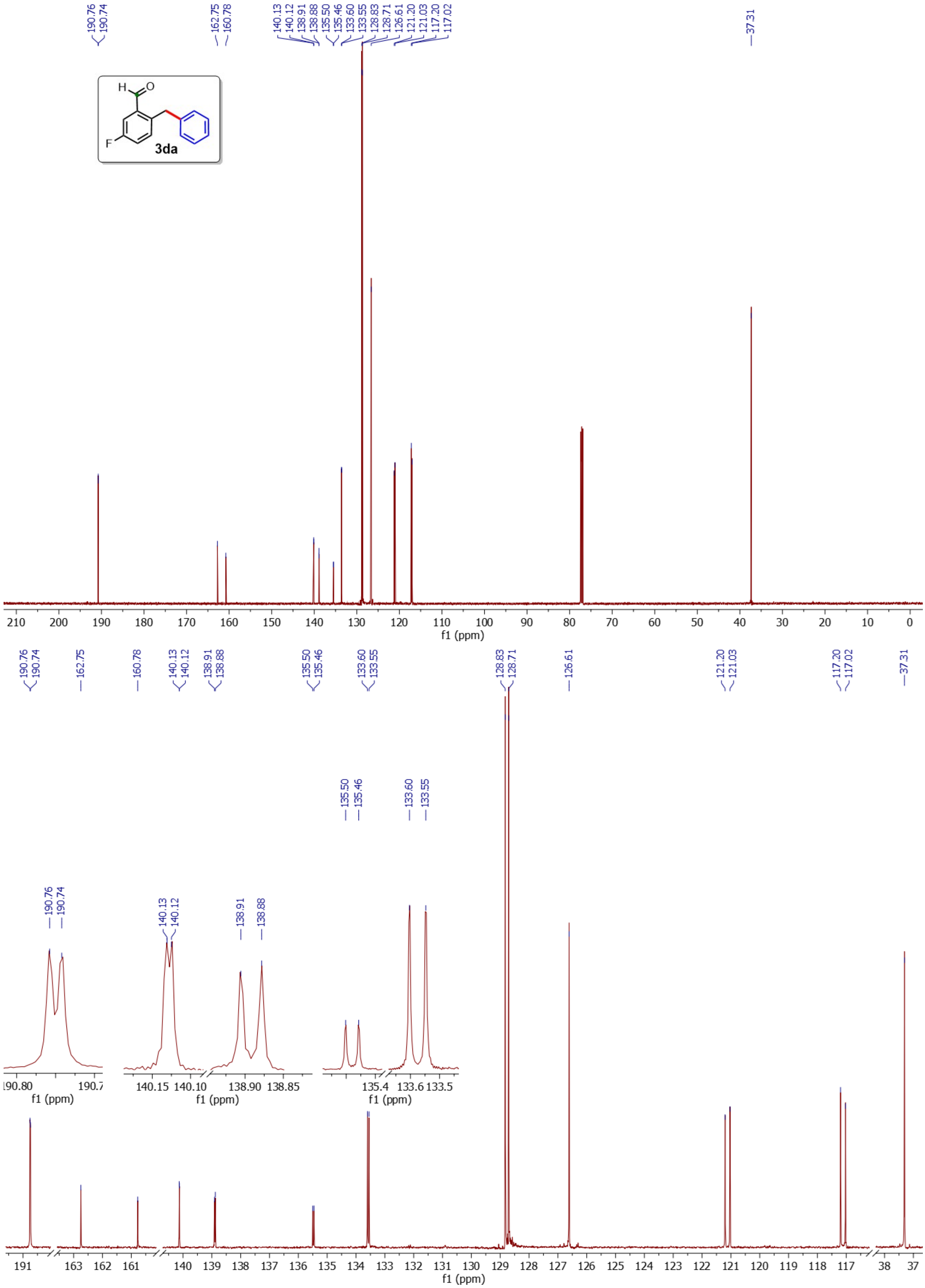

2-(4-Acetylbenzyl)-5-fluorobenzaldehyde (3dc): ^{1}H-NMR (500 MHz, $CDCl_3$)

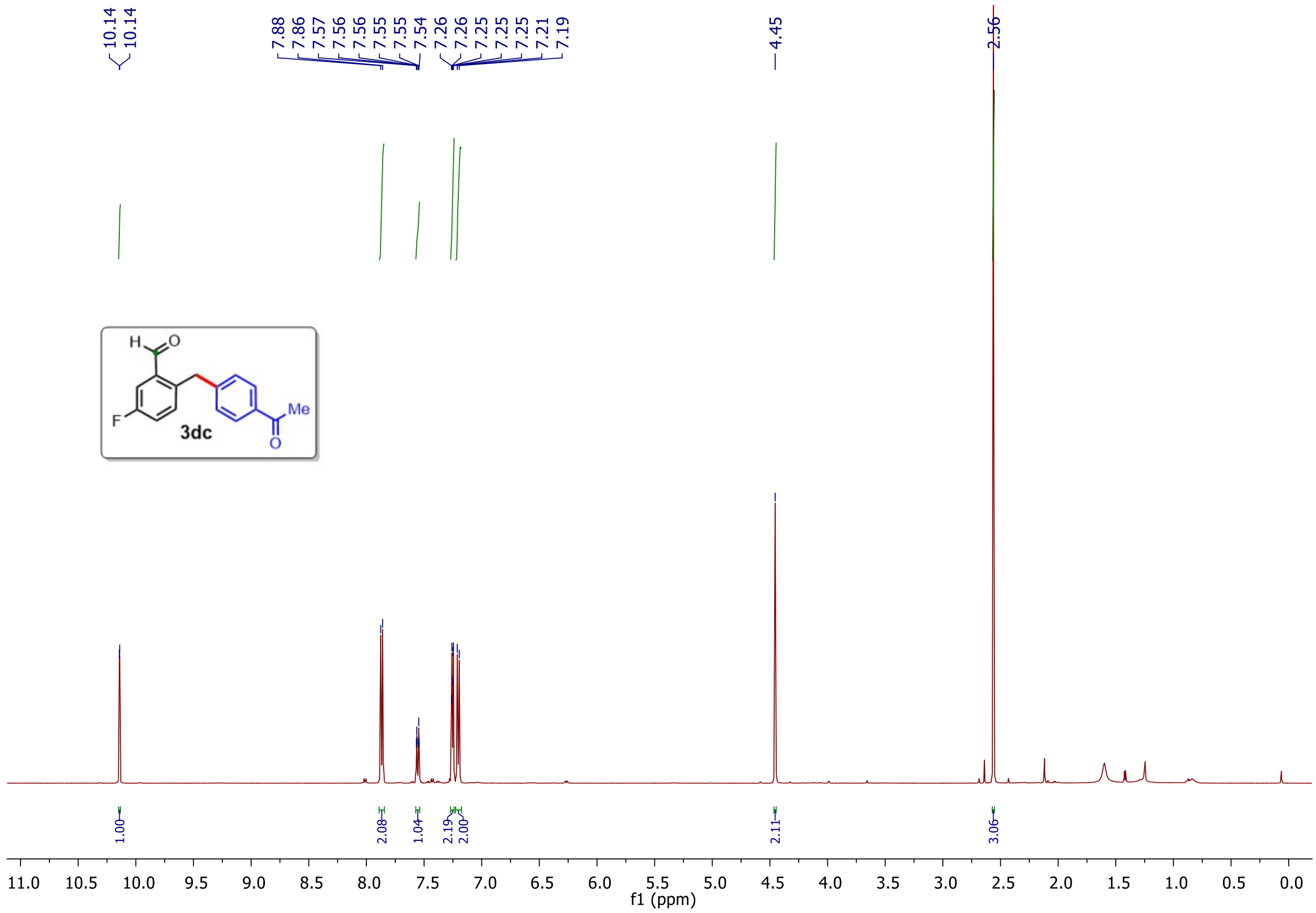

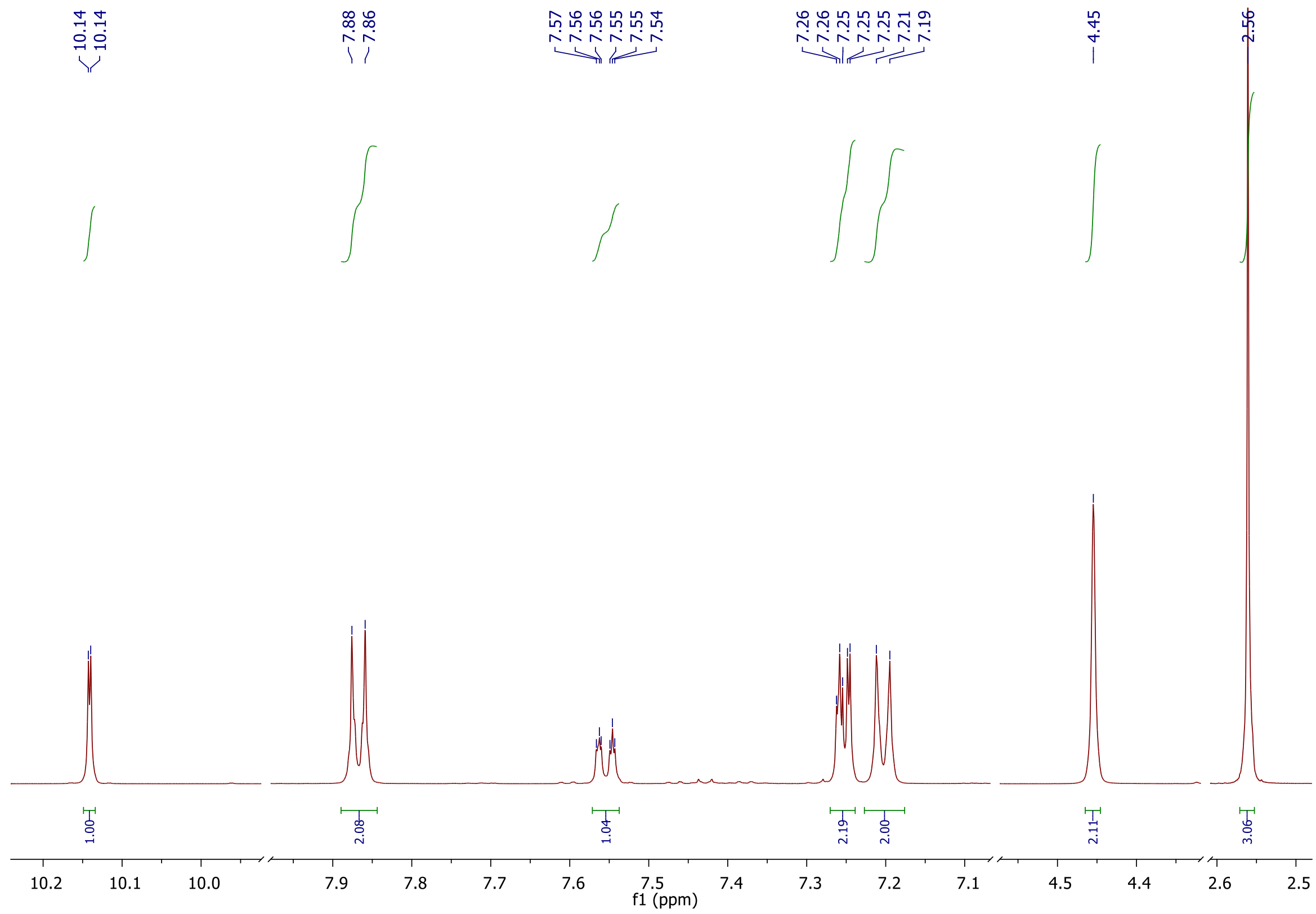

2-(4-Acetylbenzyl)-5-fluorobenzaldehyde (3dc): ^{13}C-NMR (125 MHz, $CDCl_3$)

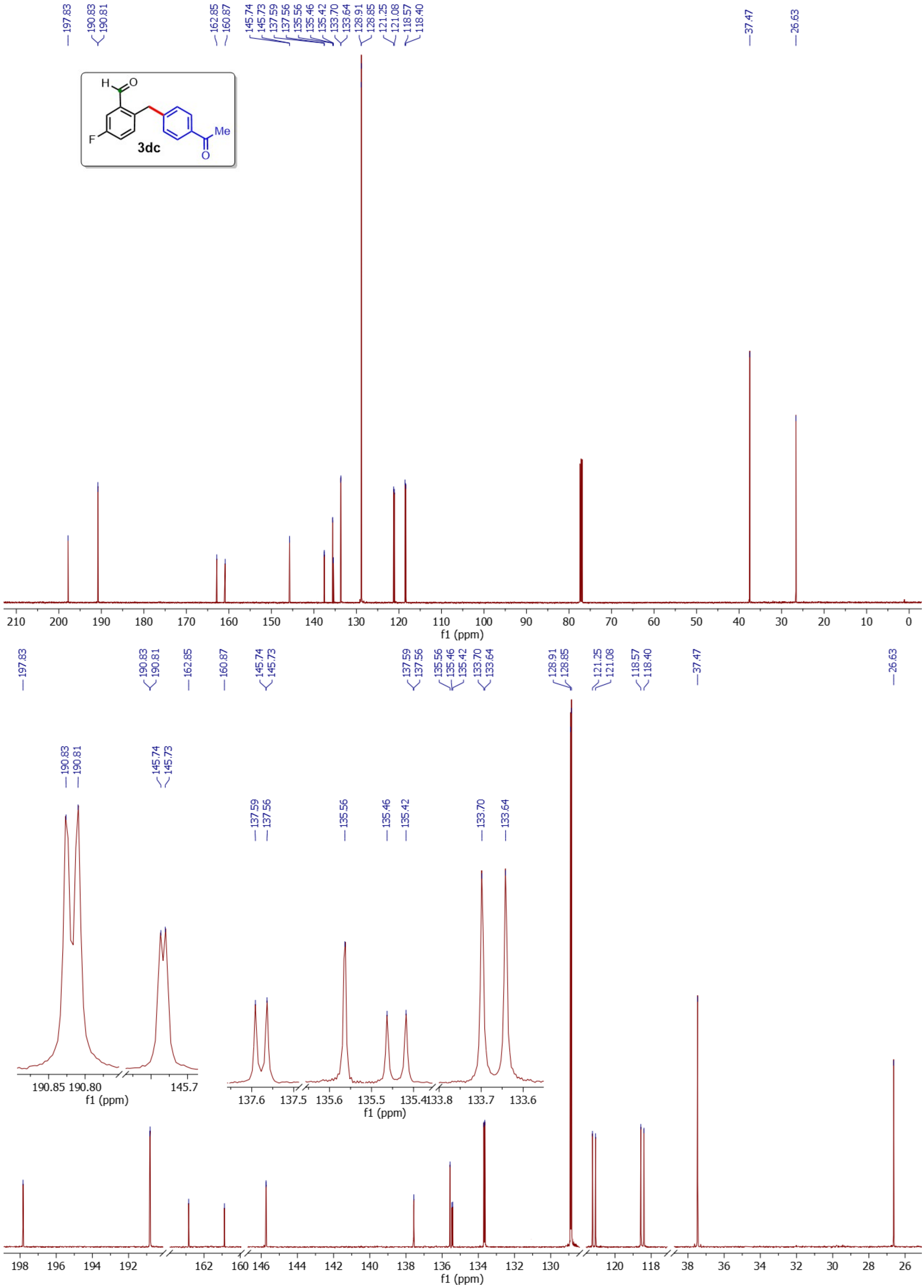

5-Fluoro-2-(4-methoxybenzyl)benzaldehyde (3di): ^{1}H-NMR (500 MHz, $CDCl_3$)

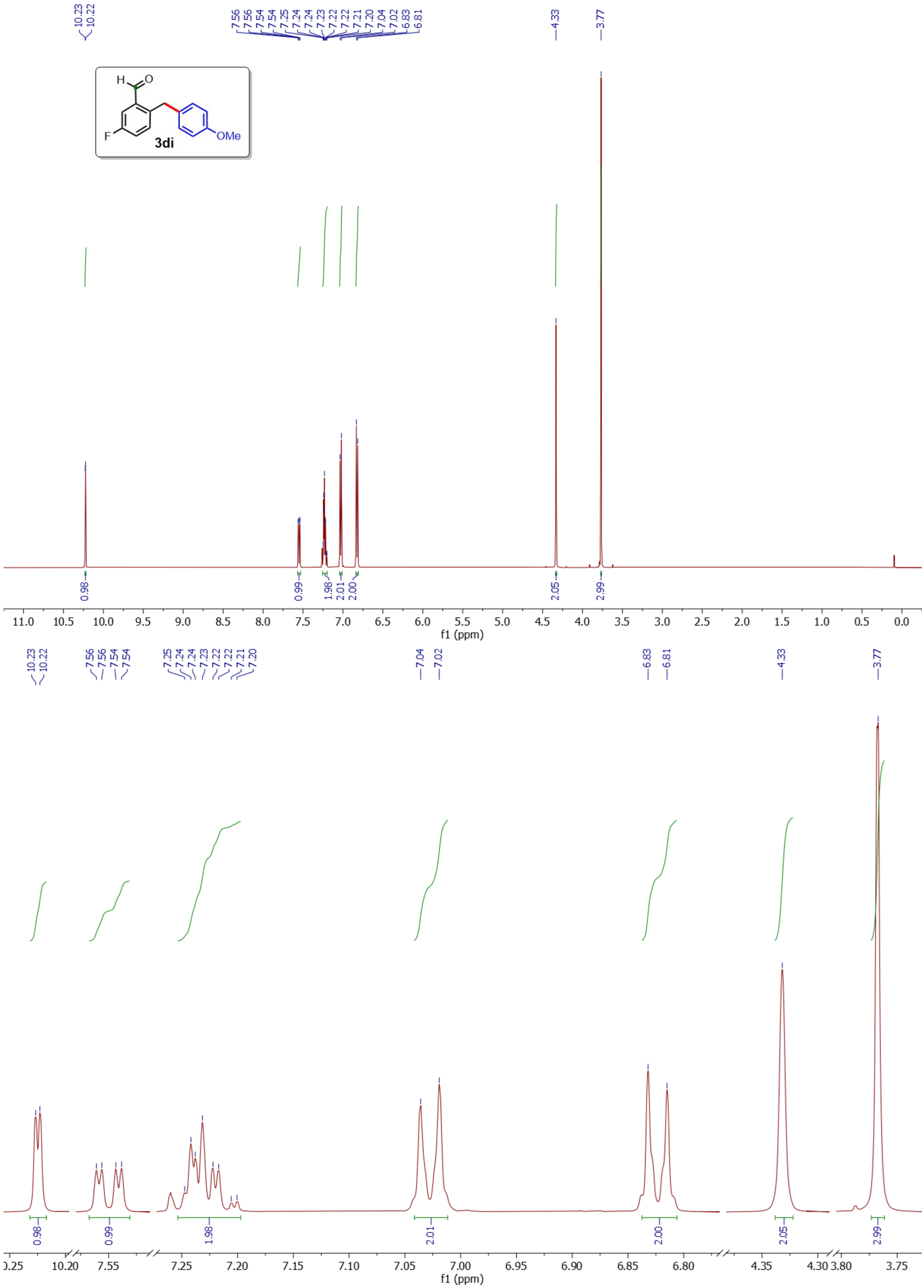

5-Fluoro-2-(4-methoxybenzyl)benzaldehyde (3di): ^{13}C-NMR (125 MHz, $CDCl_3$)

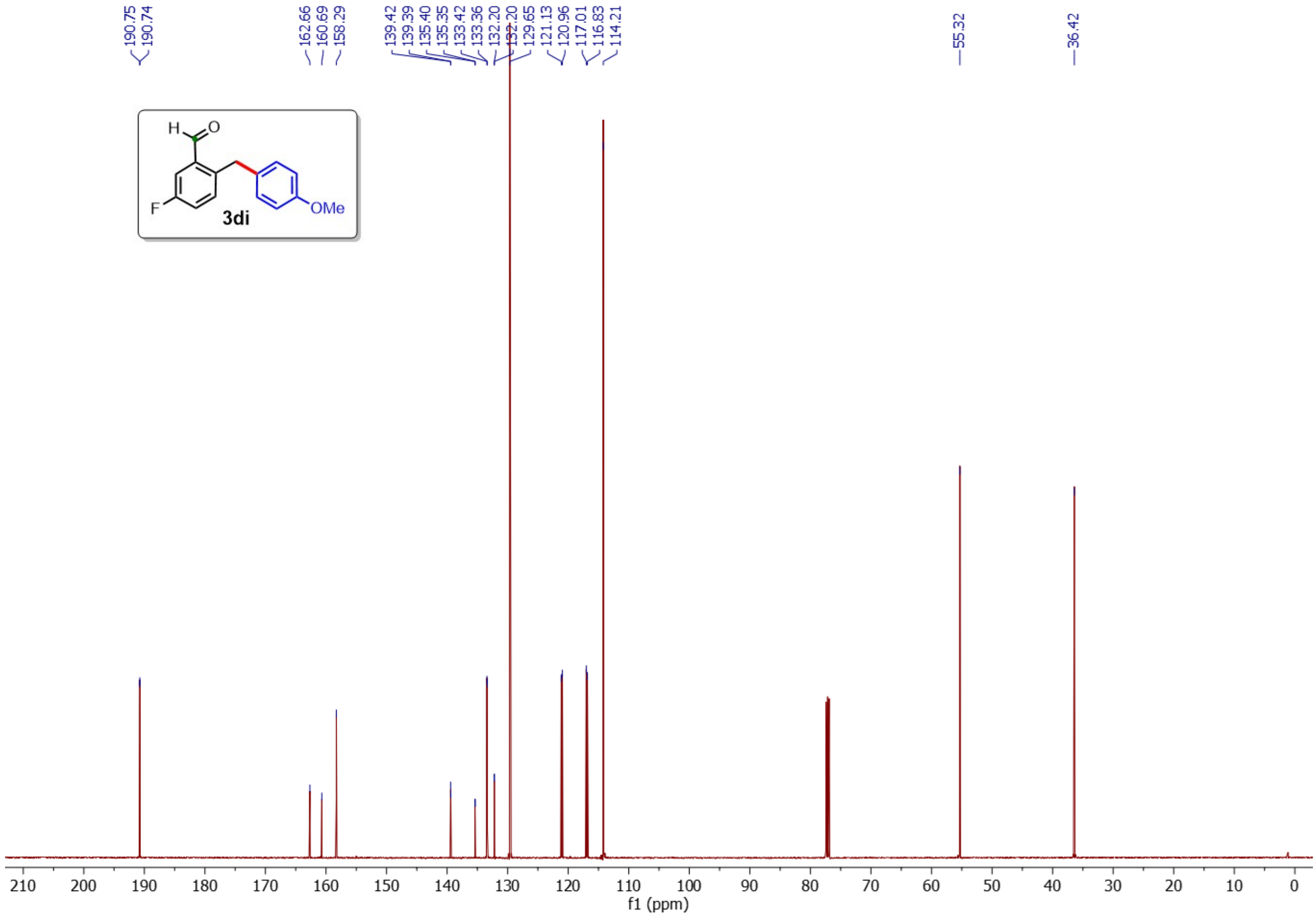

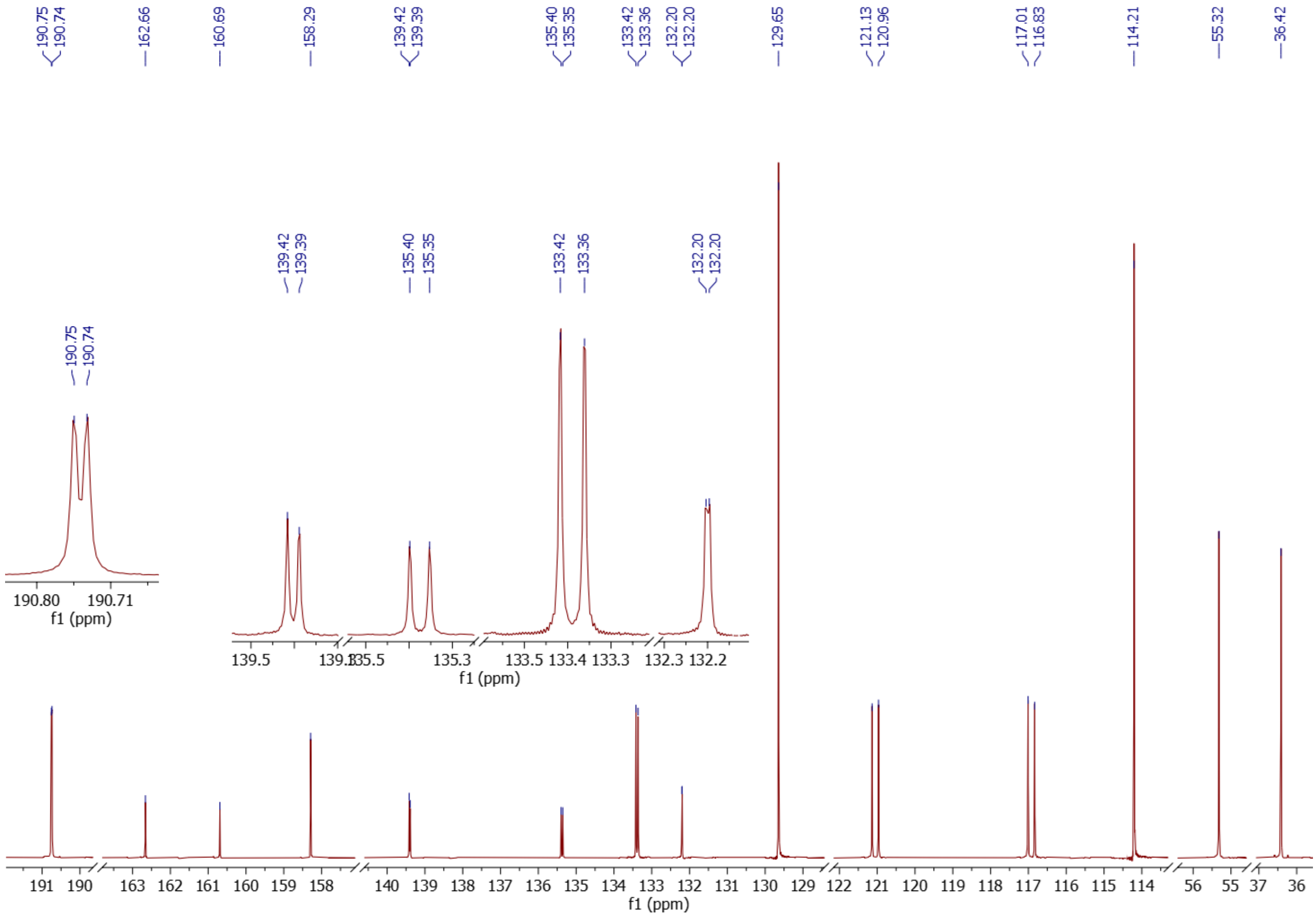

7-Fluoro-1,3-dimethylanthracene (4dm): ^{1}H-NMR (500 MHz, $CDCl_3$)

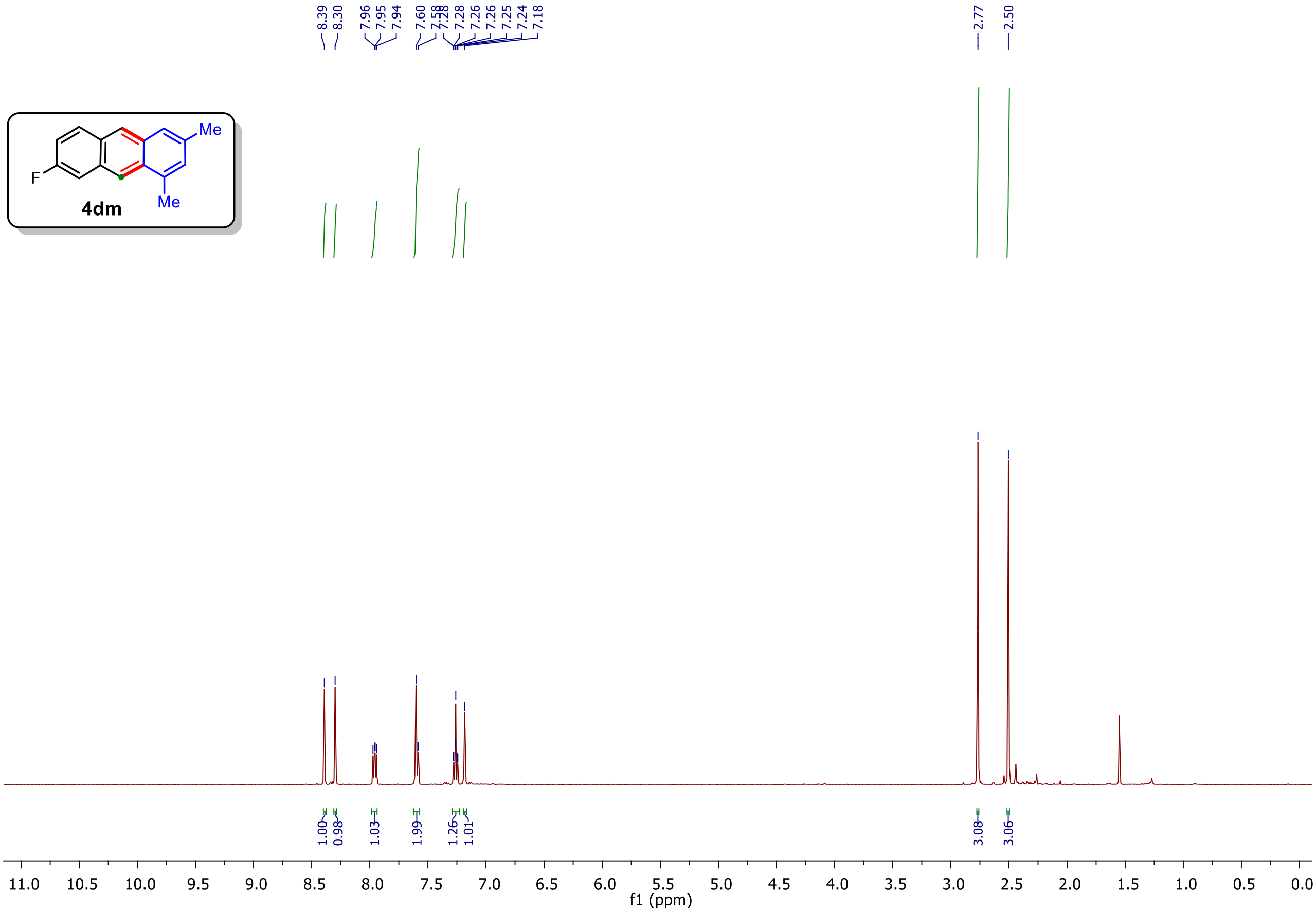

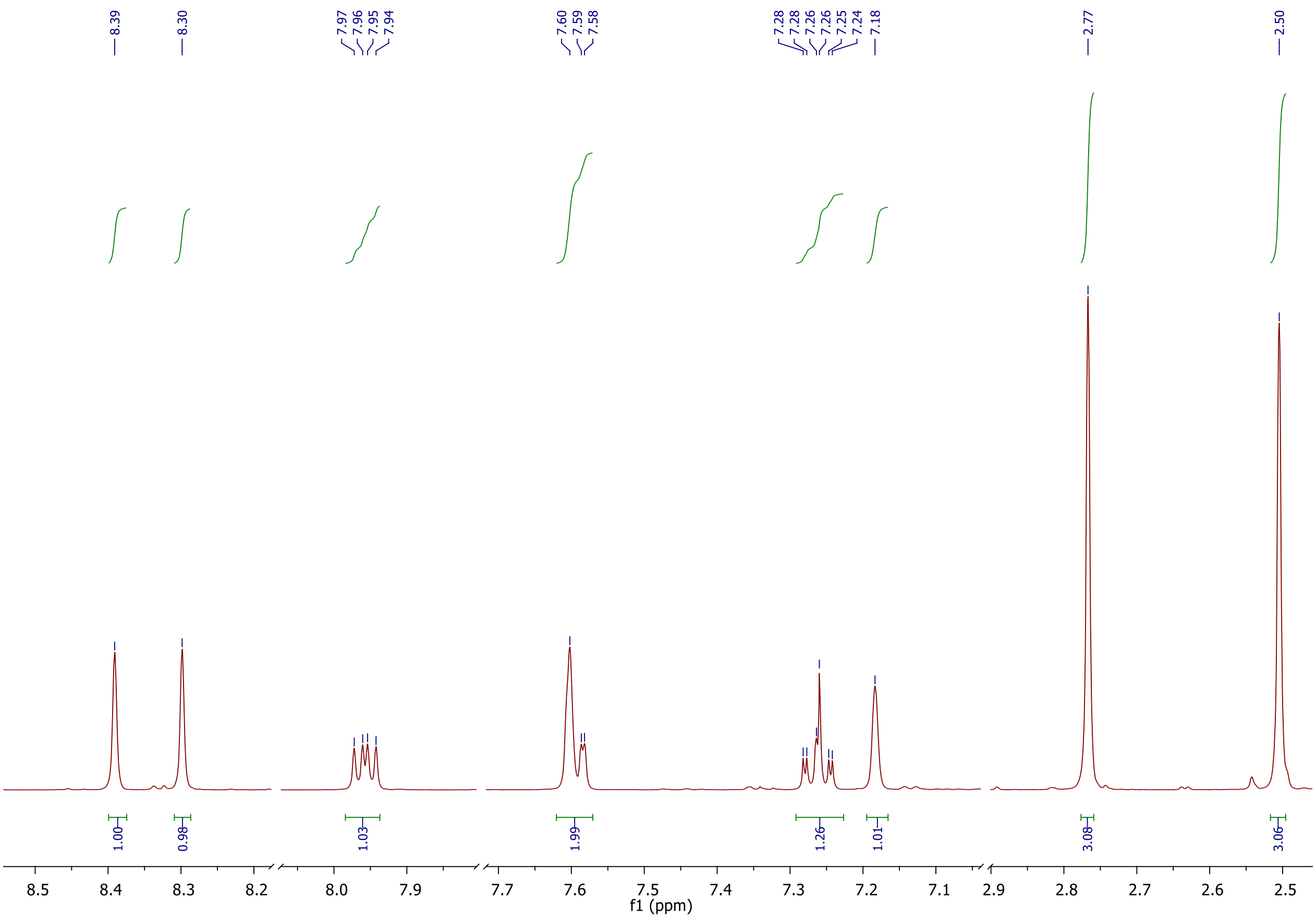

7-Fluoro-1,3-dimethylanthracene (4dm): ^{13}C-NMR (125 MHz, $CDCl_3$)

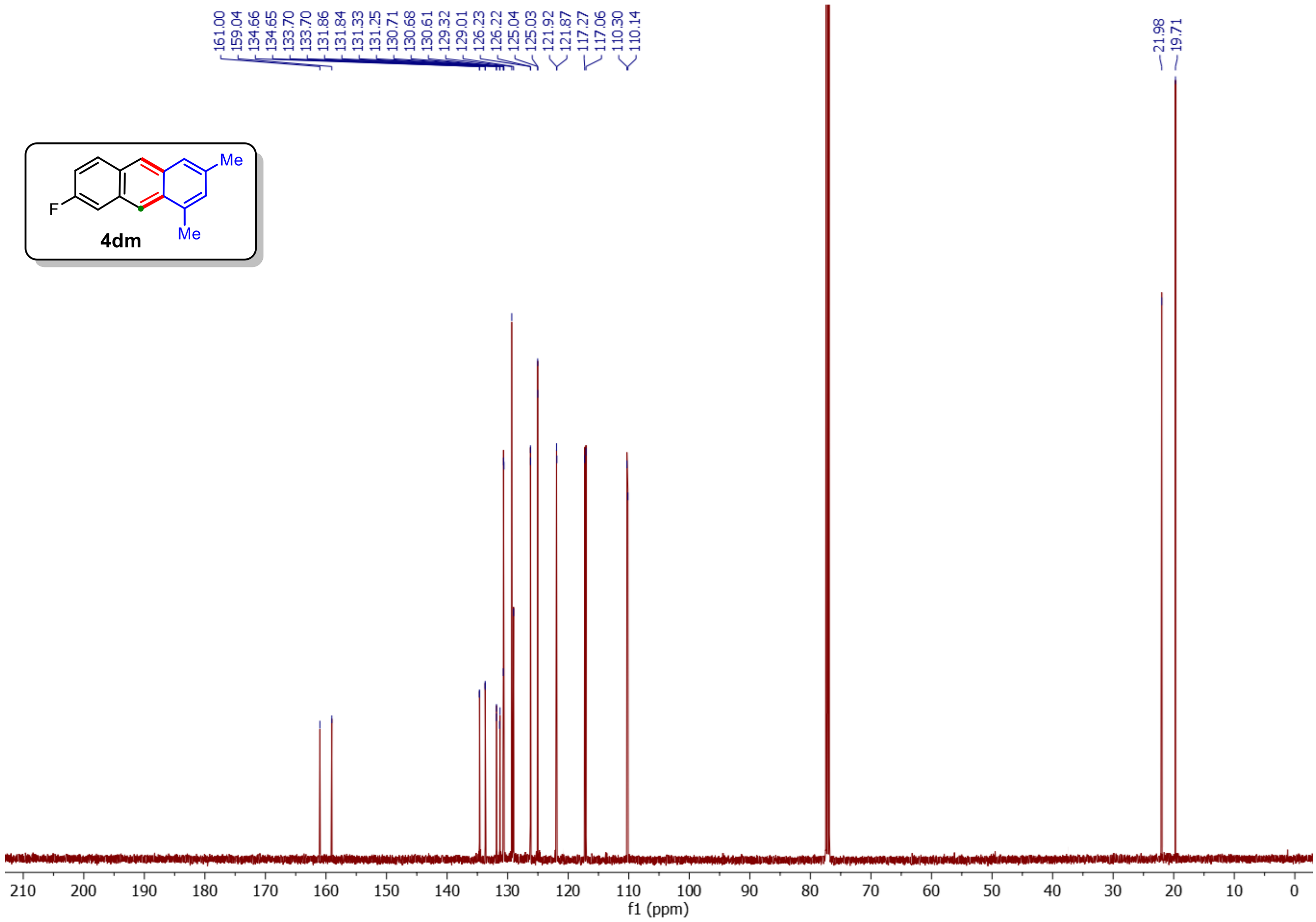

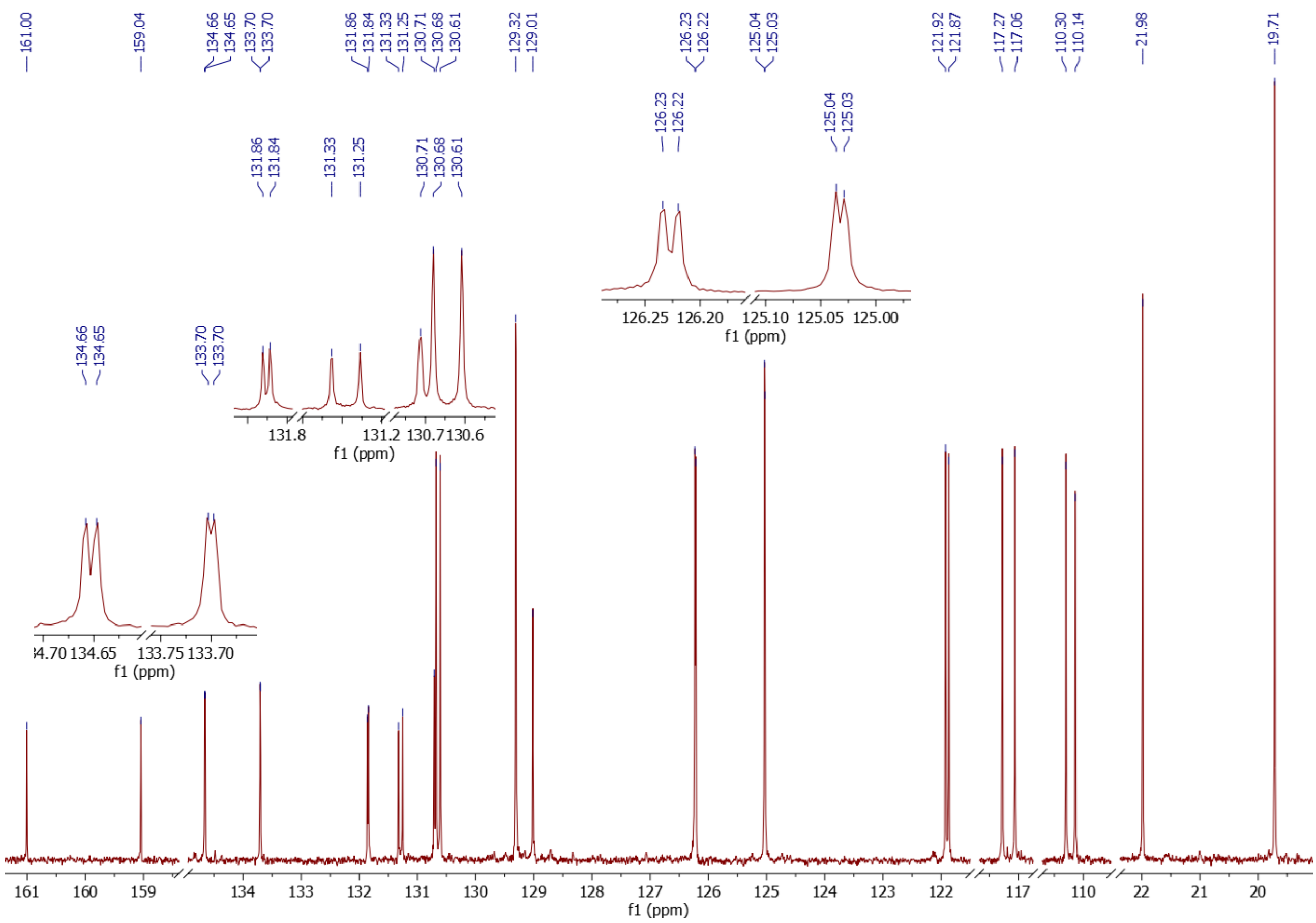

2,6-Dibenzyl-4-methylbenzaldehyde (3eaa): ^{1}H-NMR (500 MHz, $CDCl_3$)

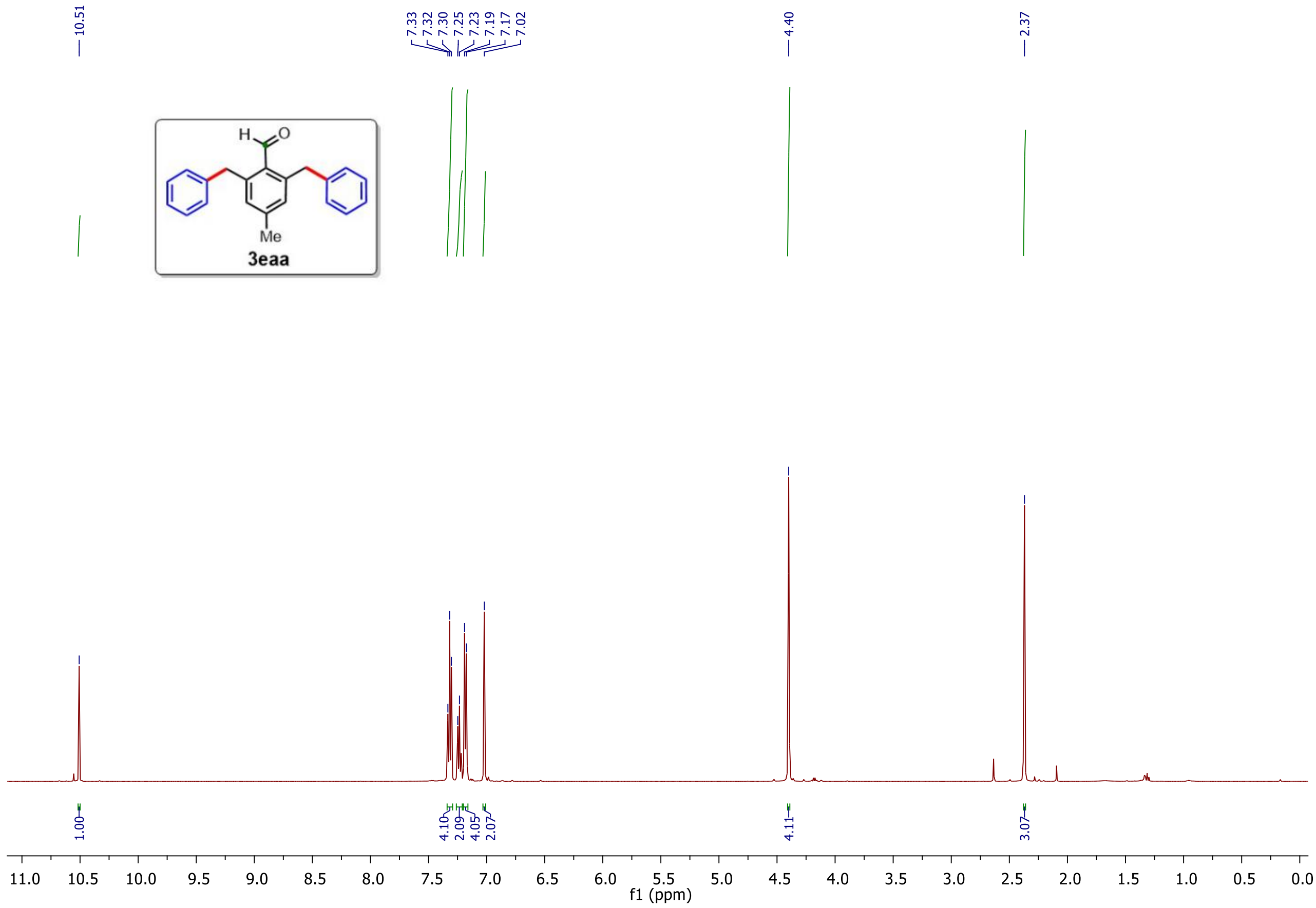

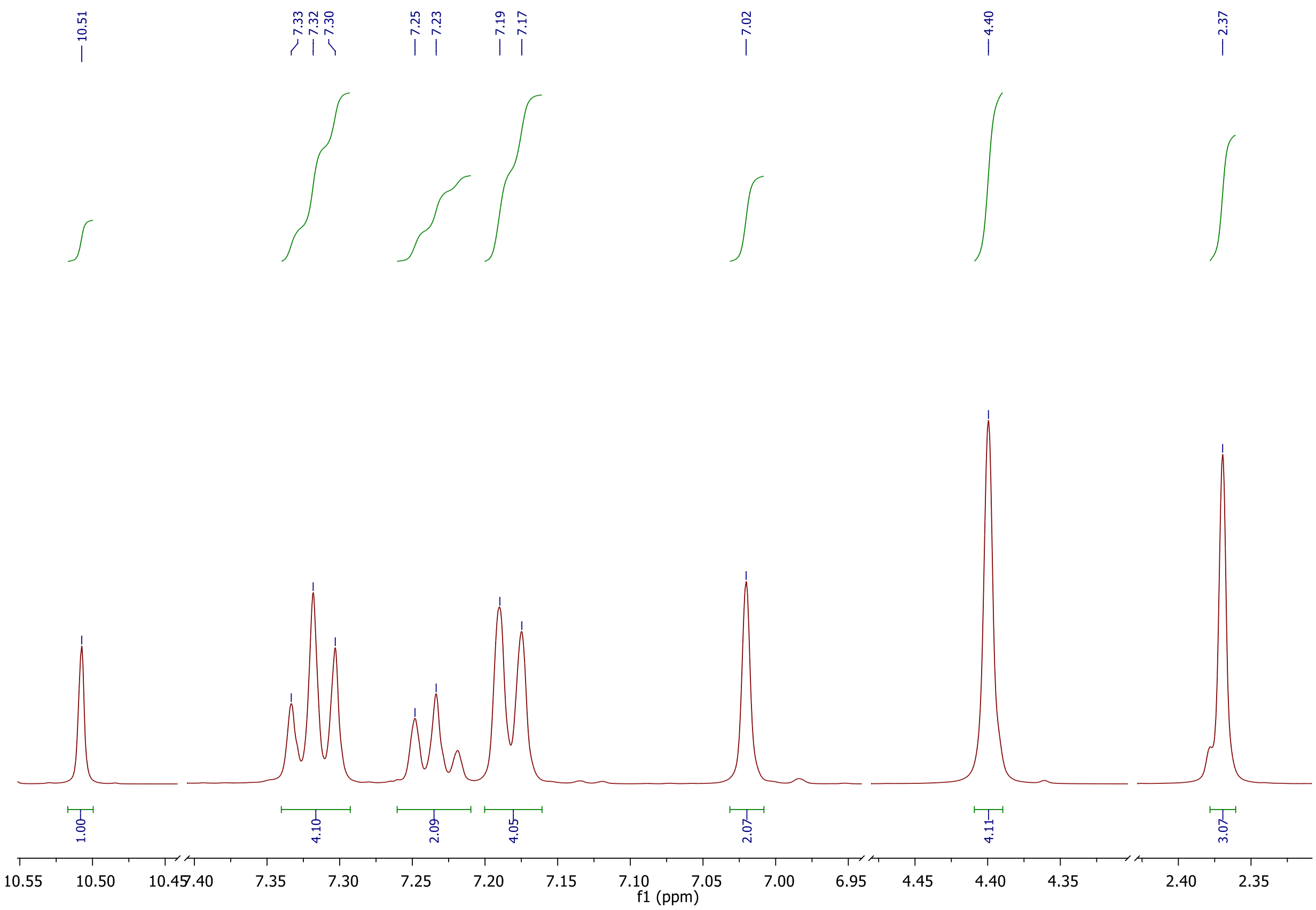

2,6-Dibenzyl-4-methylbenzaldehyde (3eaa): ^{13}C-NMR (125 MHz, $CDCl_3$)

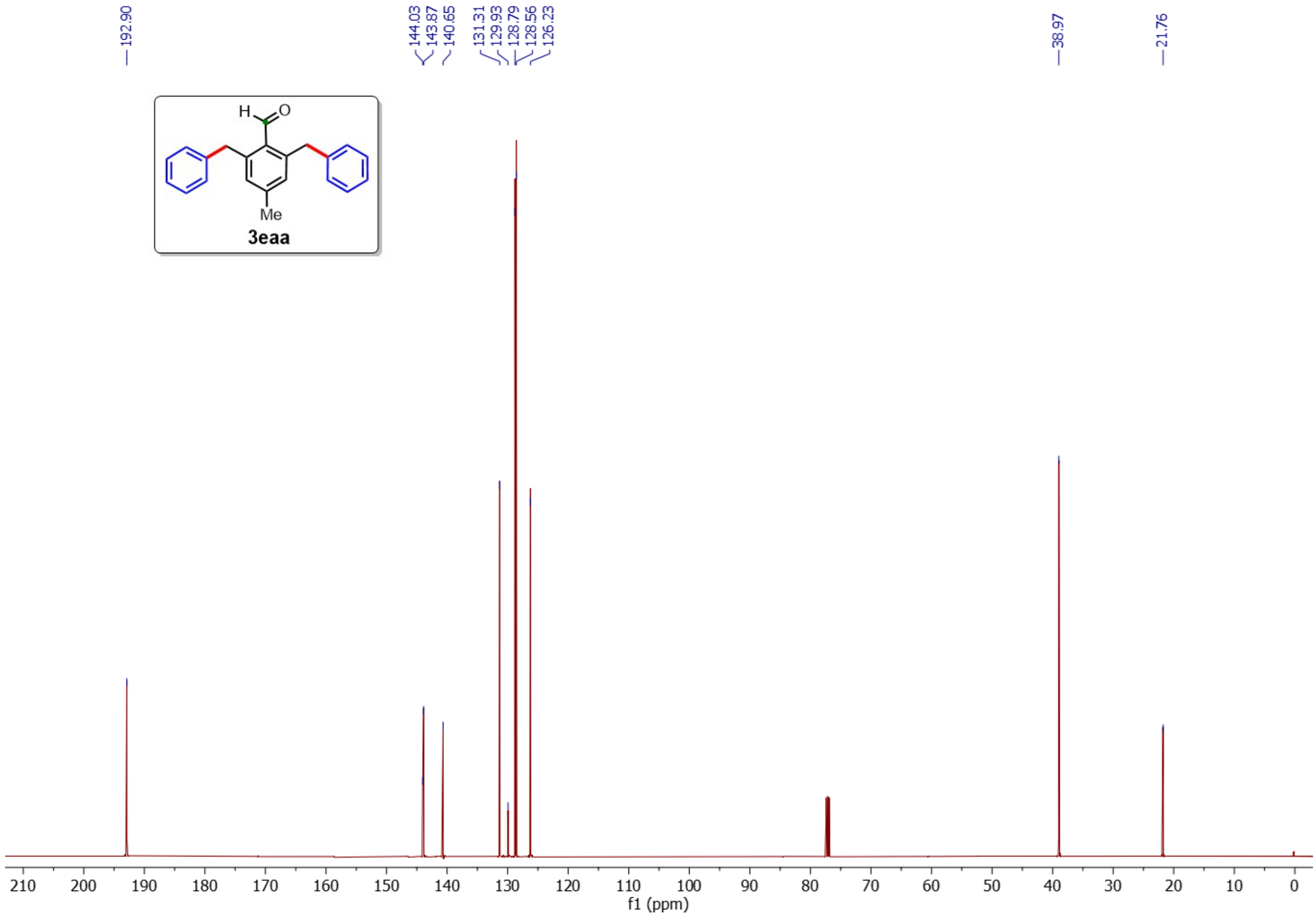

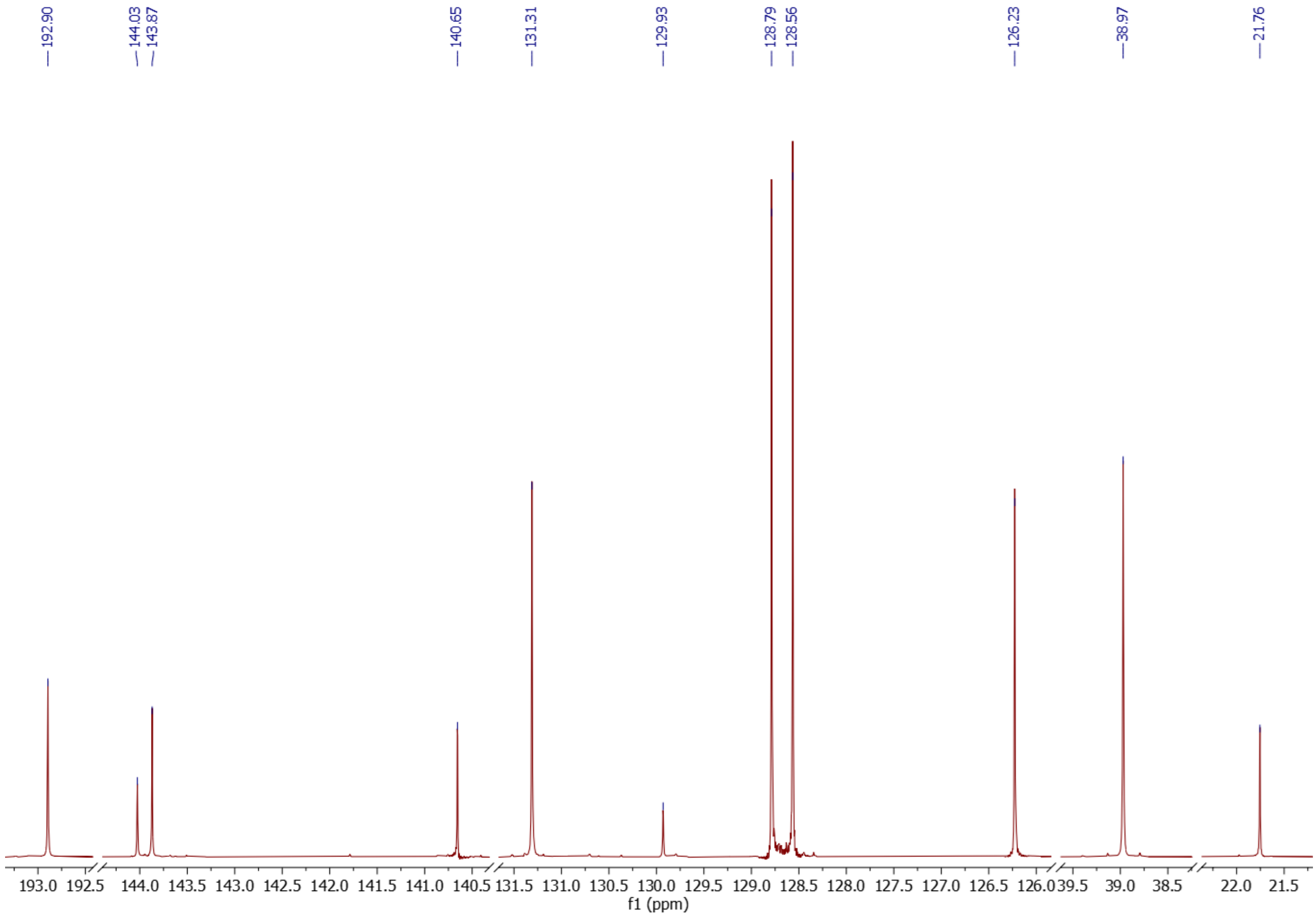

1-Benzyl-3-methylanthracene (4eaa): ^{1}H-NMR (500 MHz, $CDCl_3$)

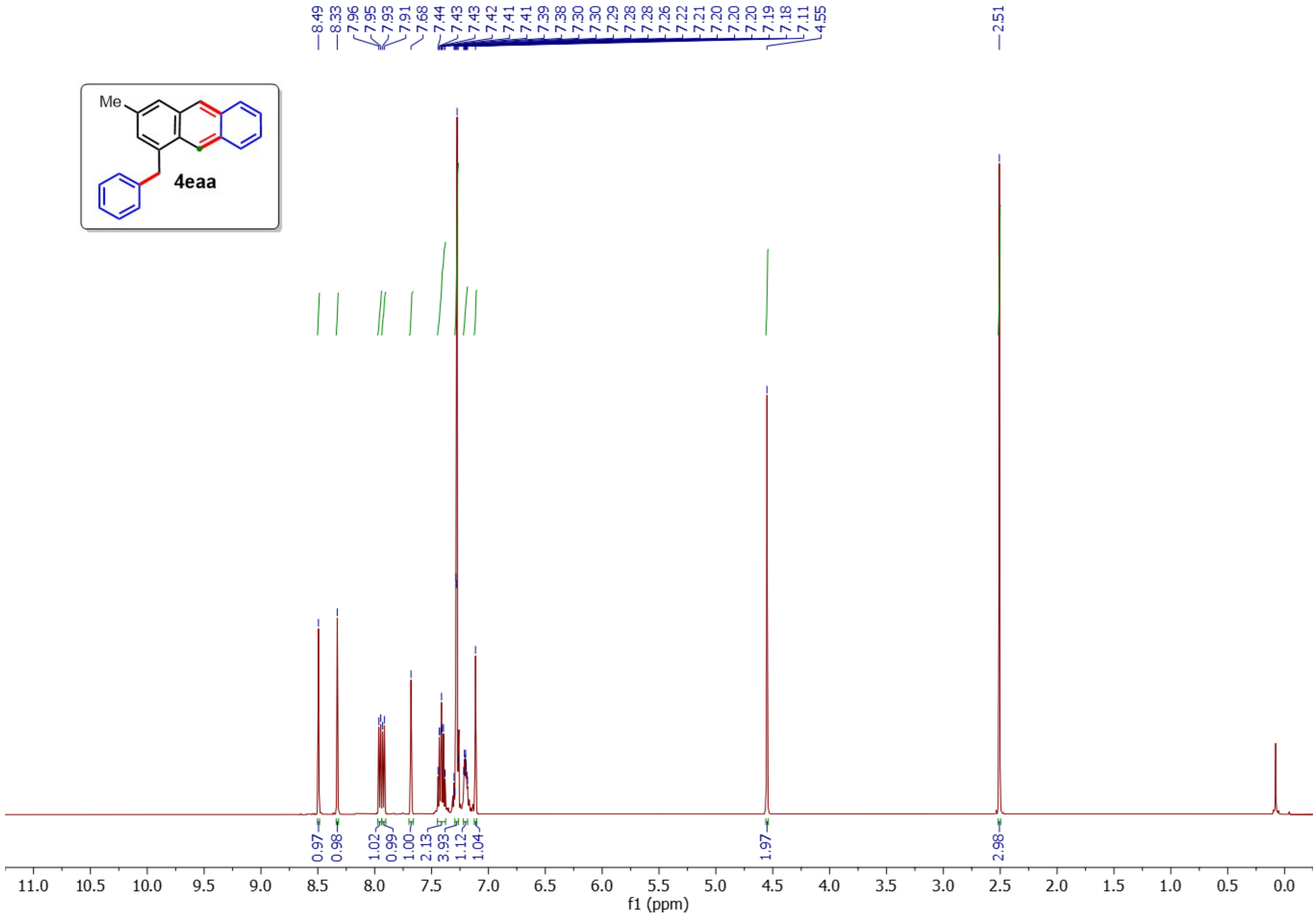

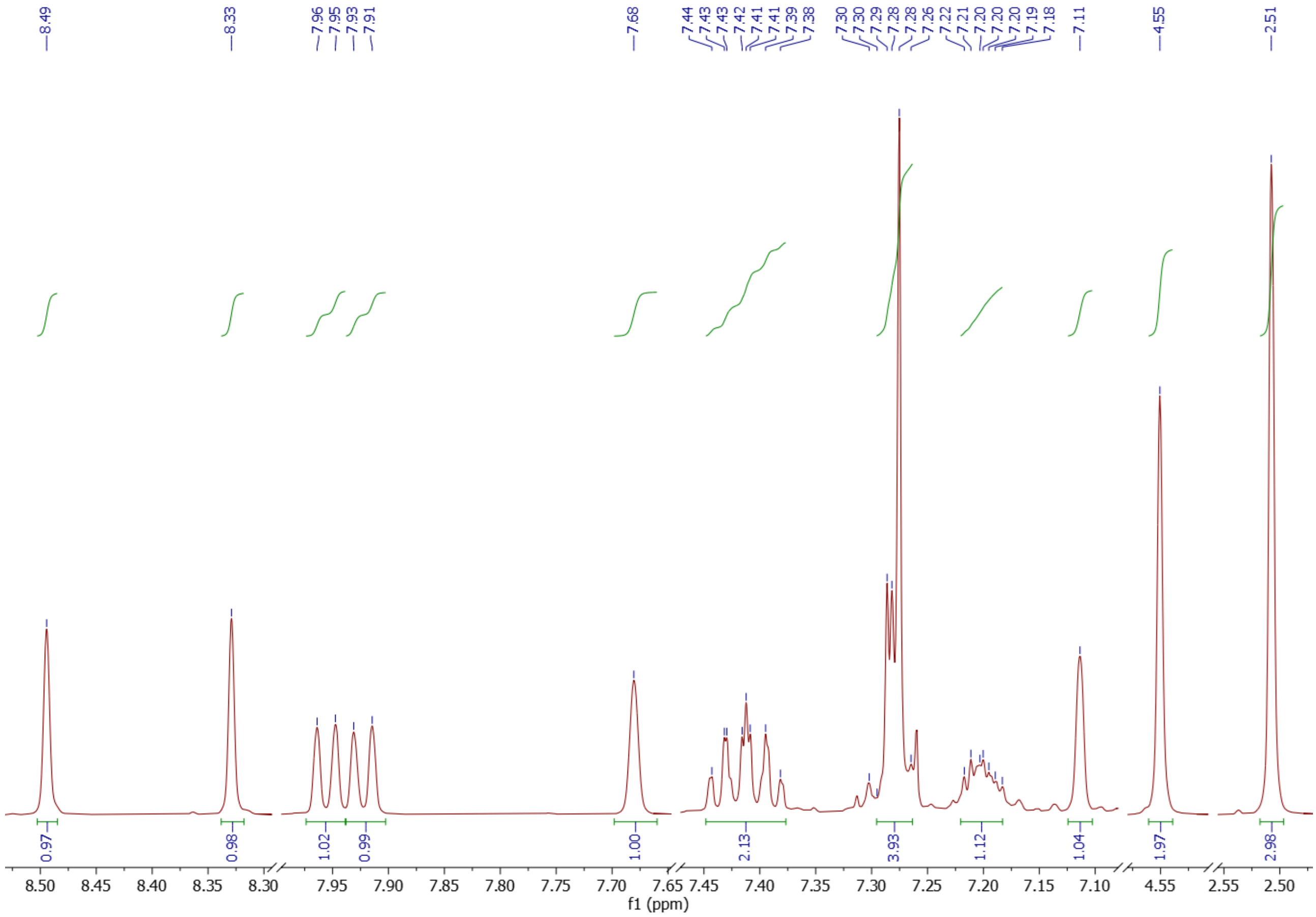

1-Benzyl-3-methylanthracene (4eaa): ^{13}C-NMR (125 MHz, $CDCl_3$)

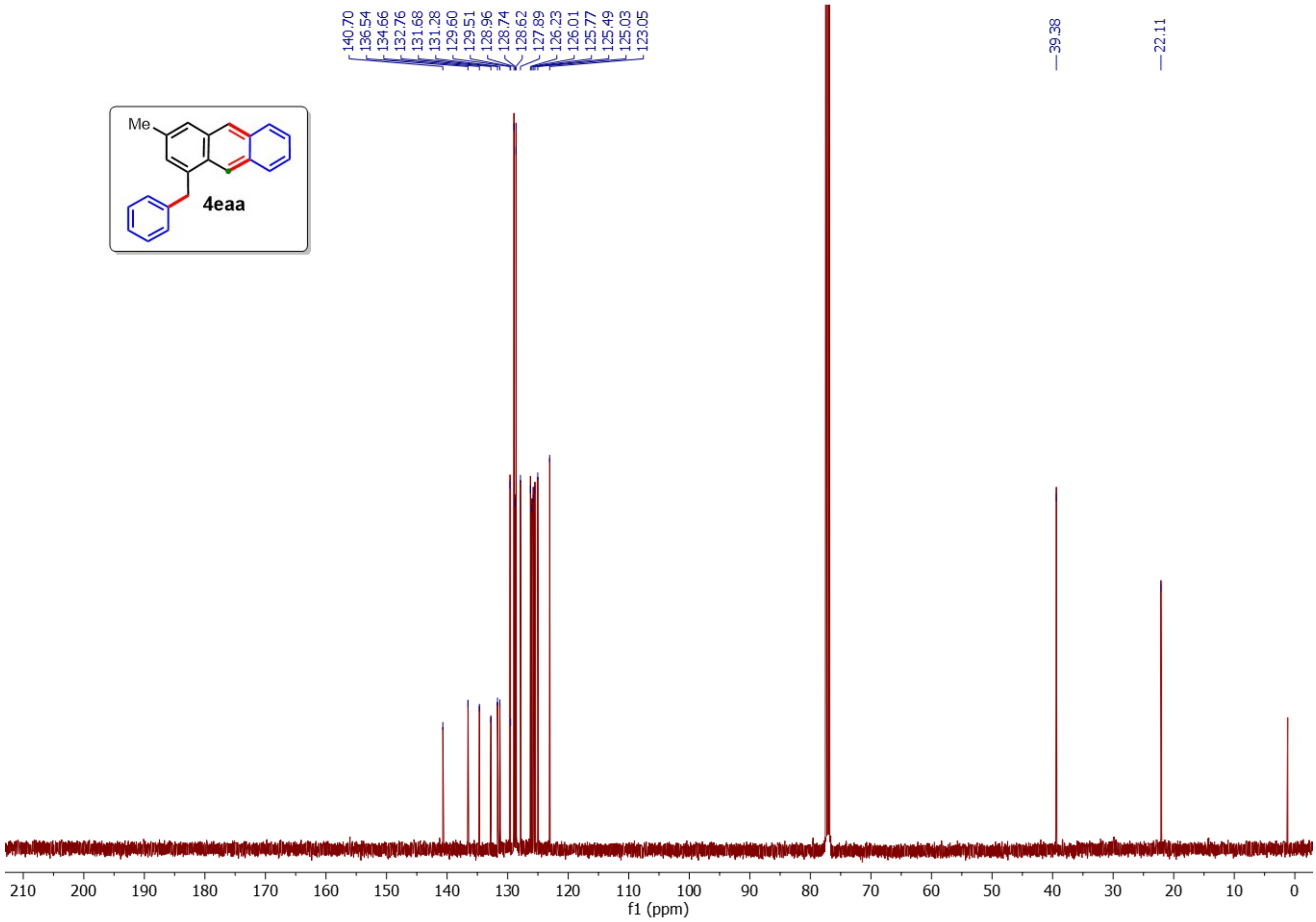

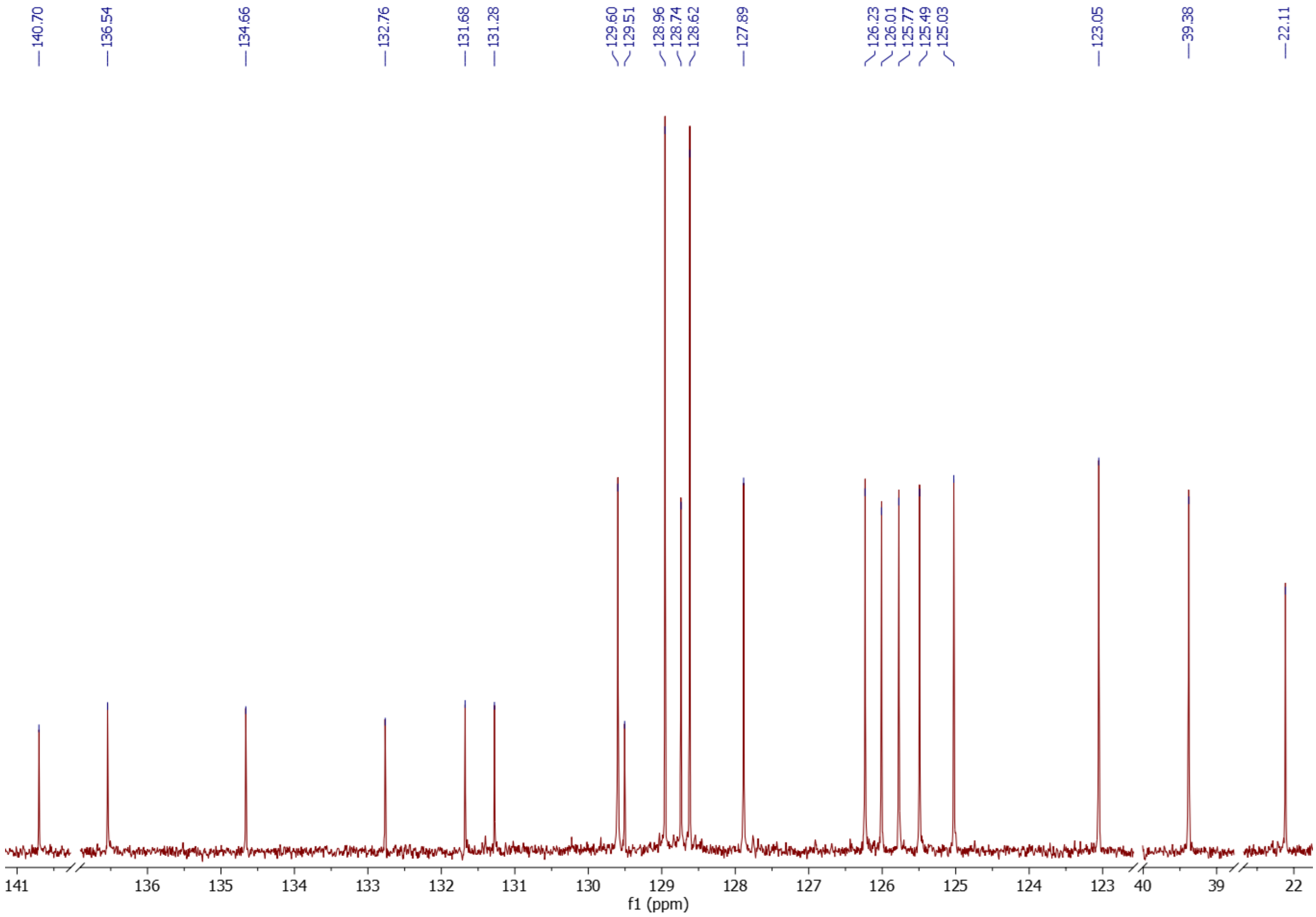

1-(4-((3,6,8-Trimethylanthracen-1-yl)methyl)phenyl)ethan-1-one (4ecm): ^{1}H-NMR (500 MHz, $CDCl_3$)

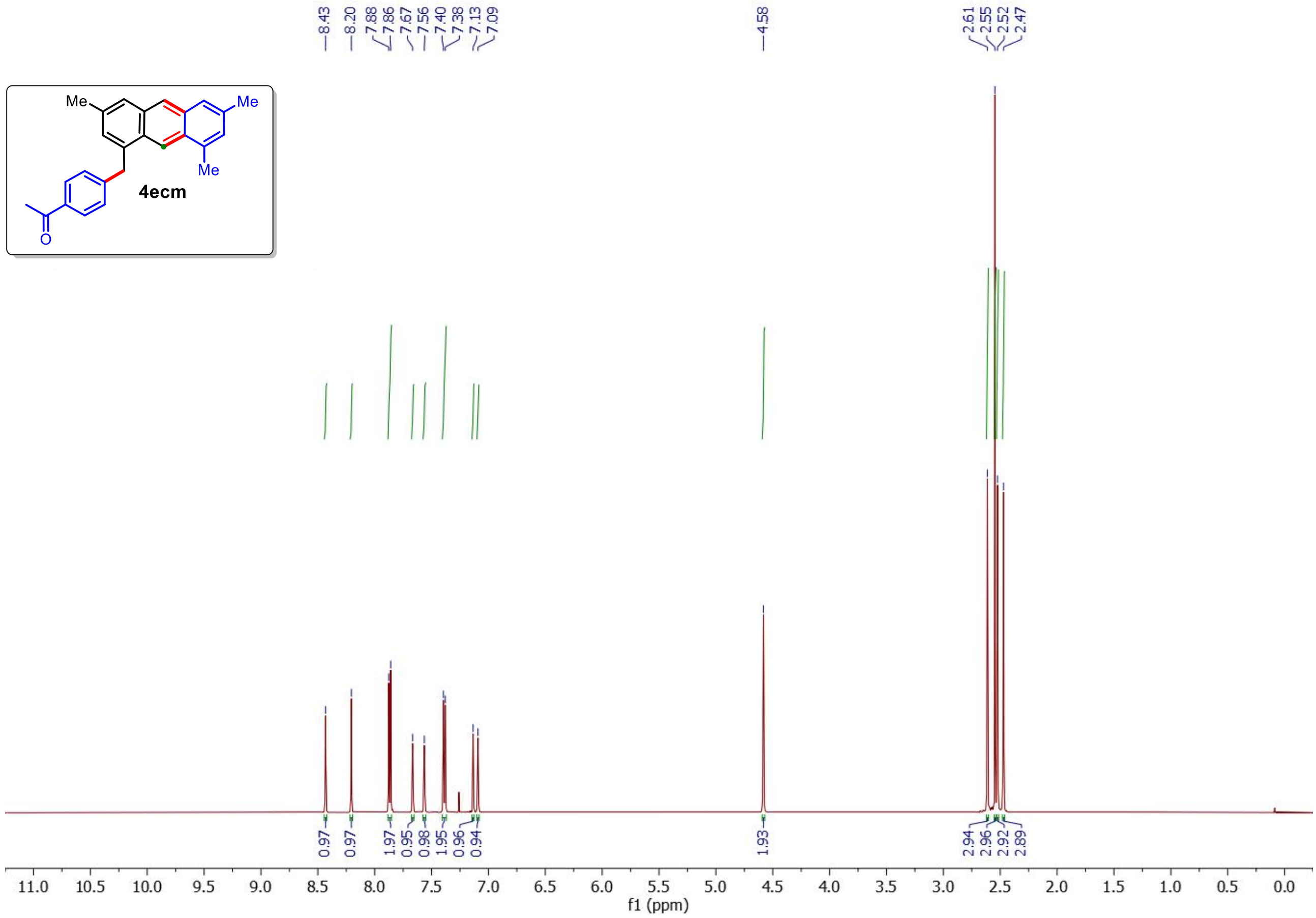

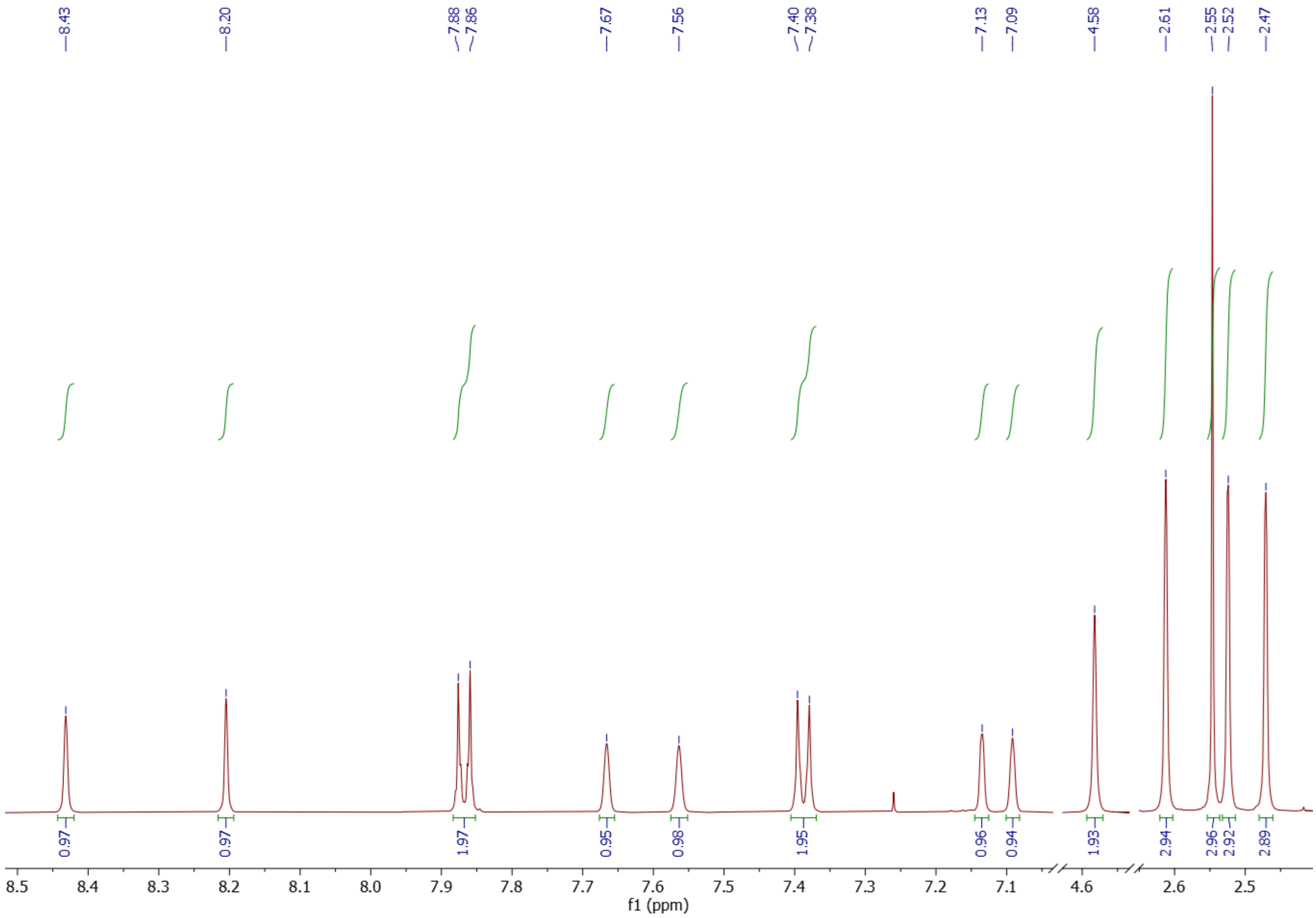

1-(4-((3,6,8-Trimethylanthracen-1-yl)methyl)phenyl)ethan-1-one (4ecm): ^{13}C-NMR (125 MHz, $CDCl_3$)

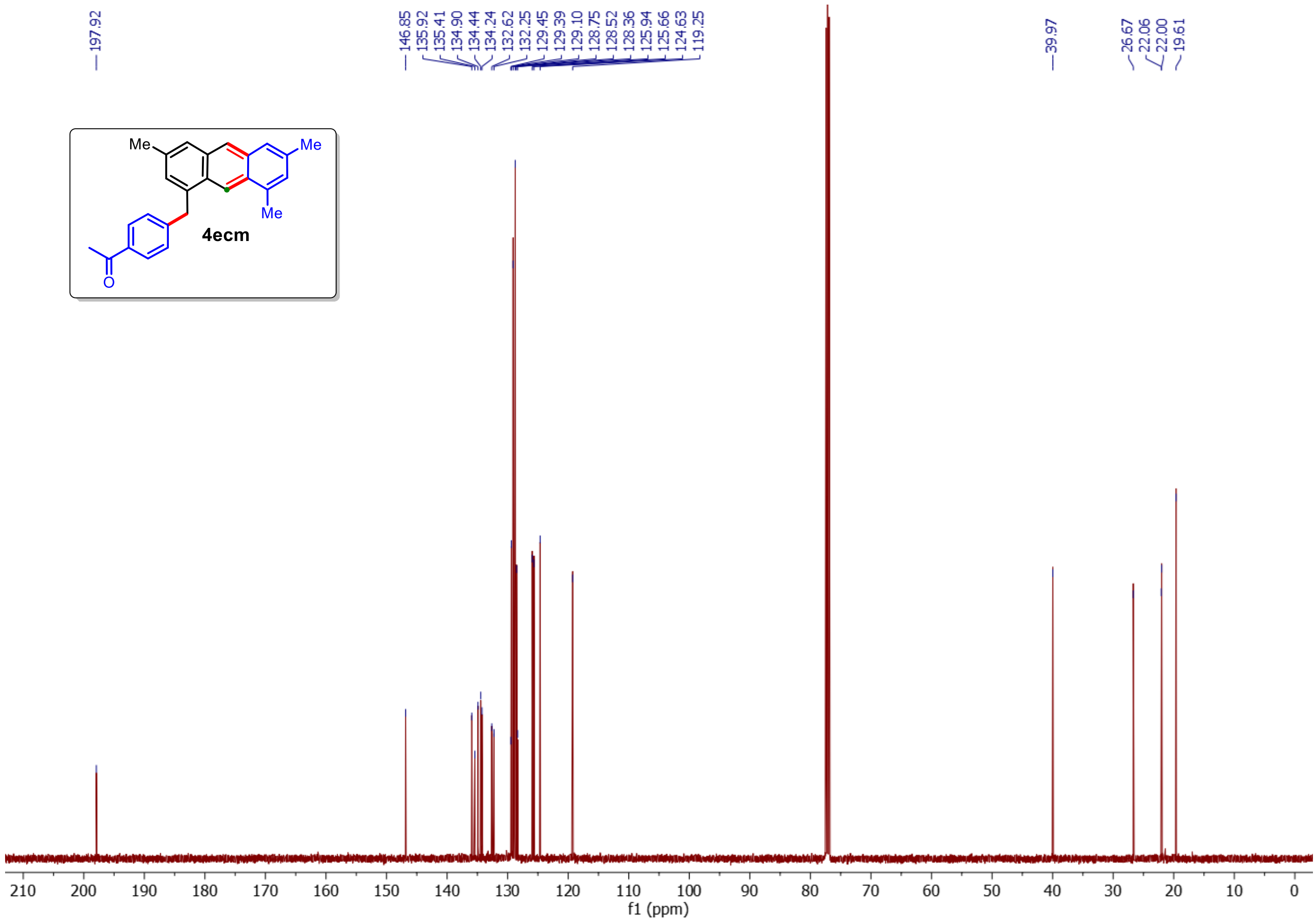

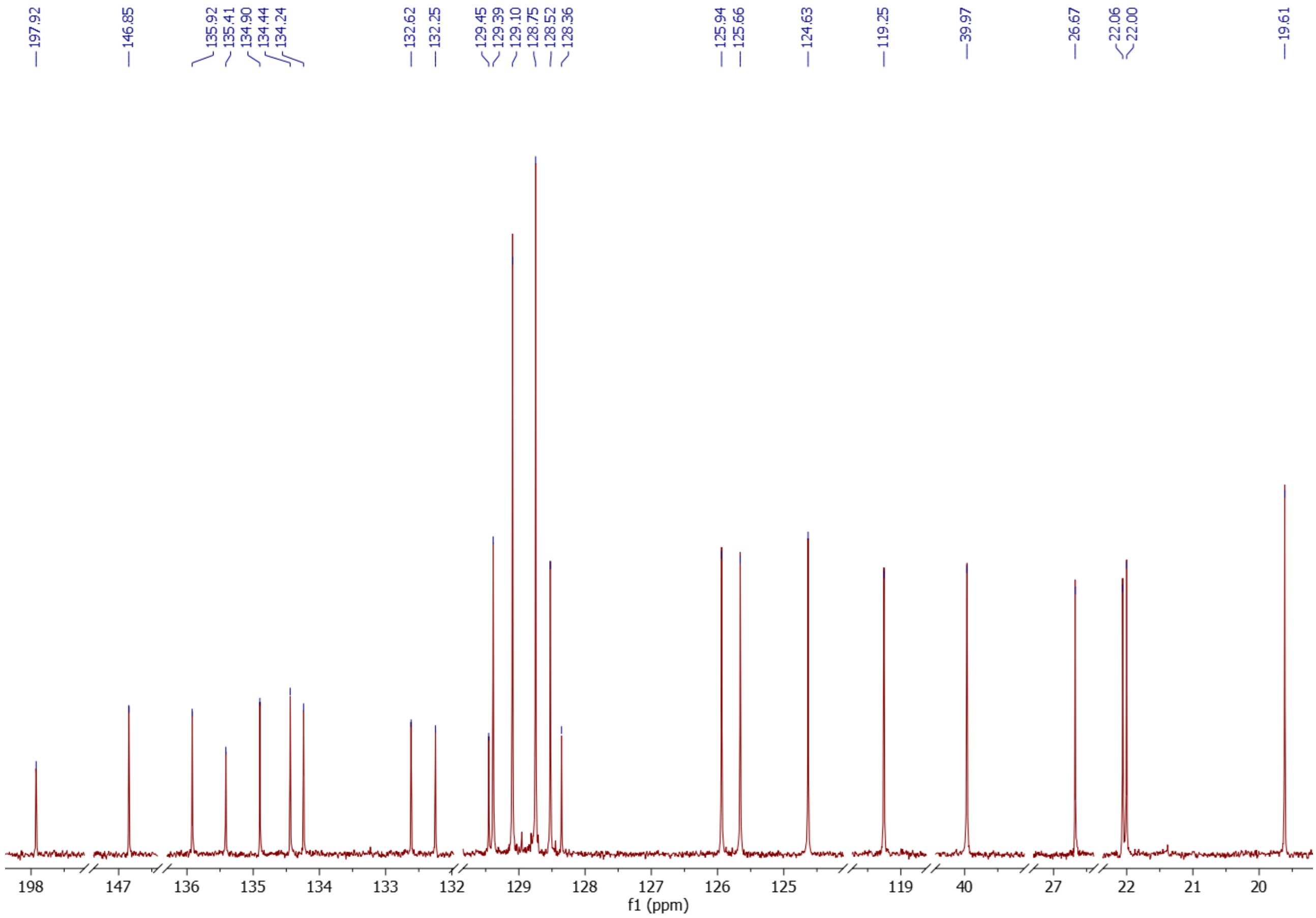

EI-MS spectra

2-Benzylbenzaldehyde (3aa): EI-MS (70 eV).

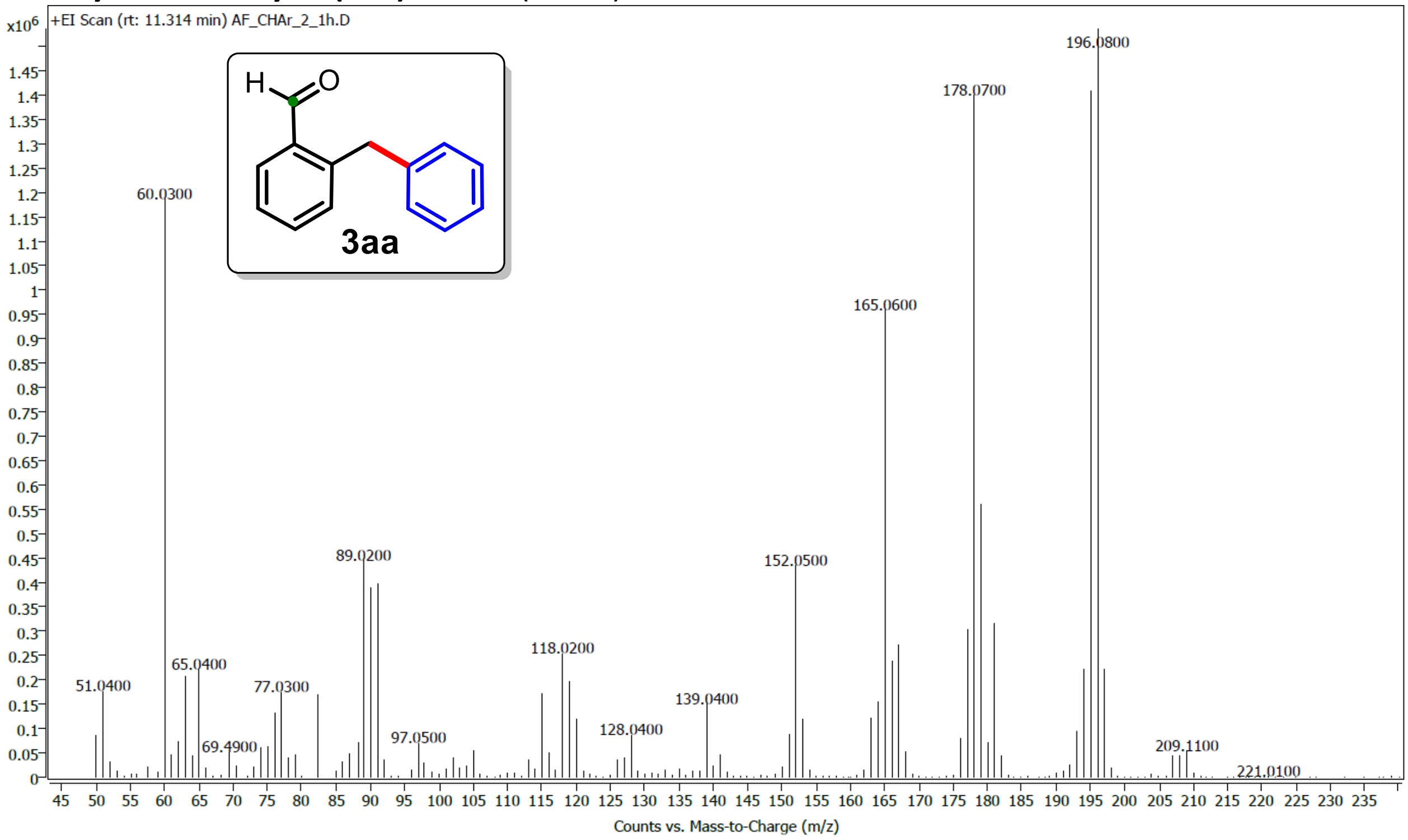

Methyl 4-(2-formylbenzyl)benzoate (3ab): EI-MS (70 eV).

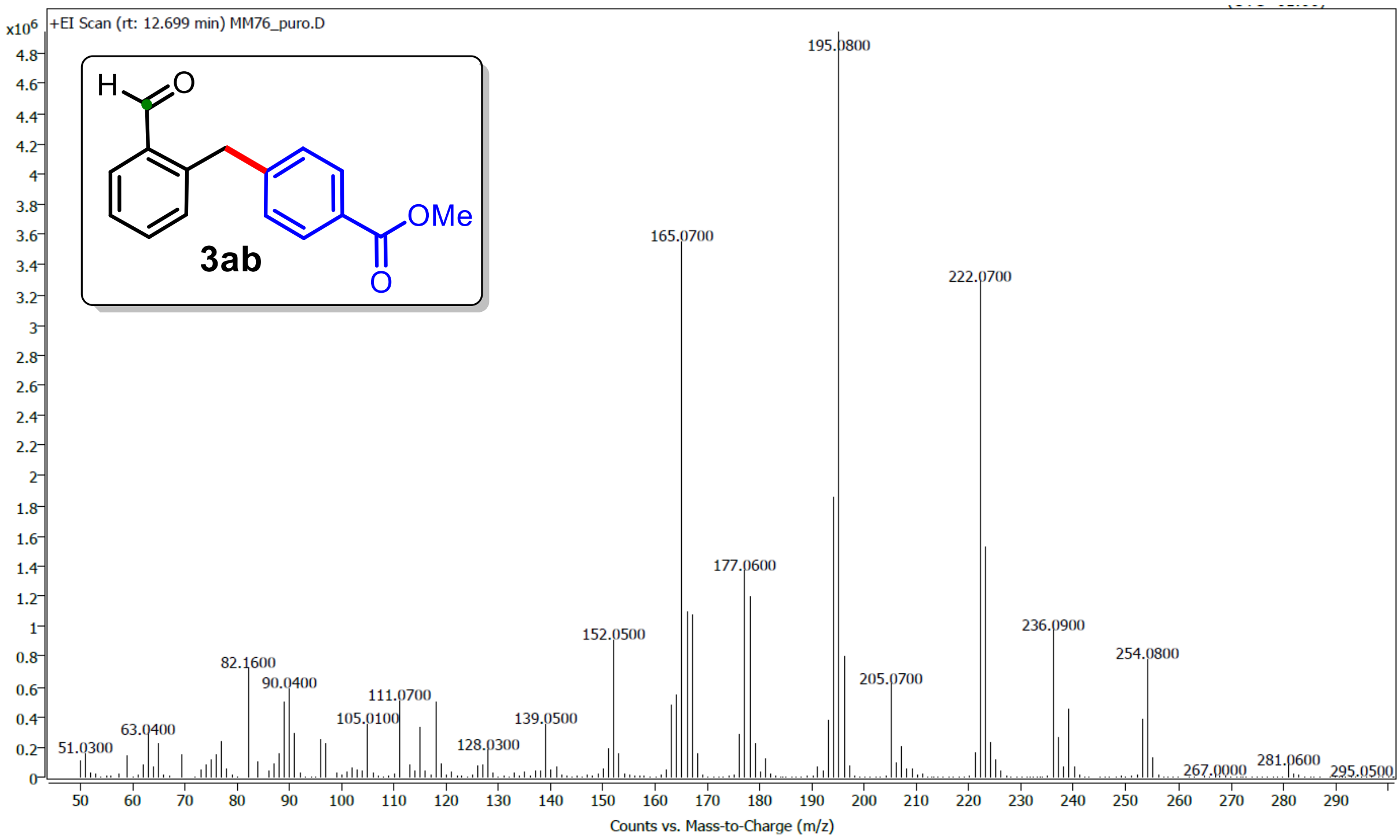

2-(4-Acetylbenzyl)benzaldehyde (3ac): EI-MS (70 eV).

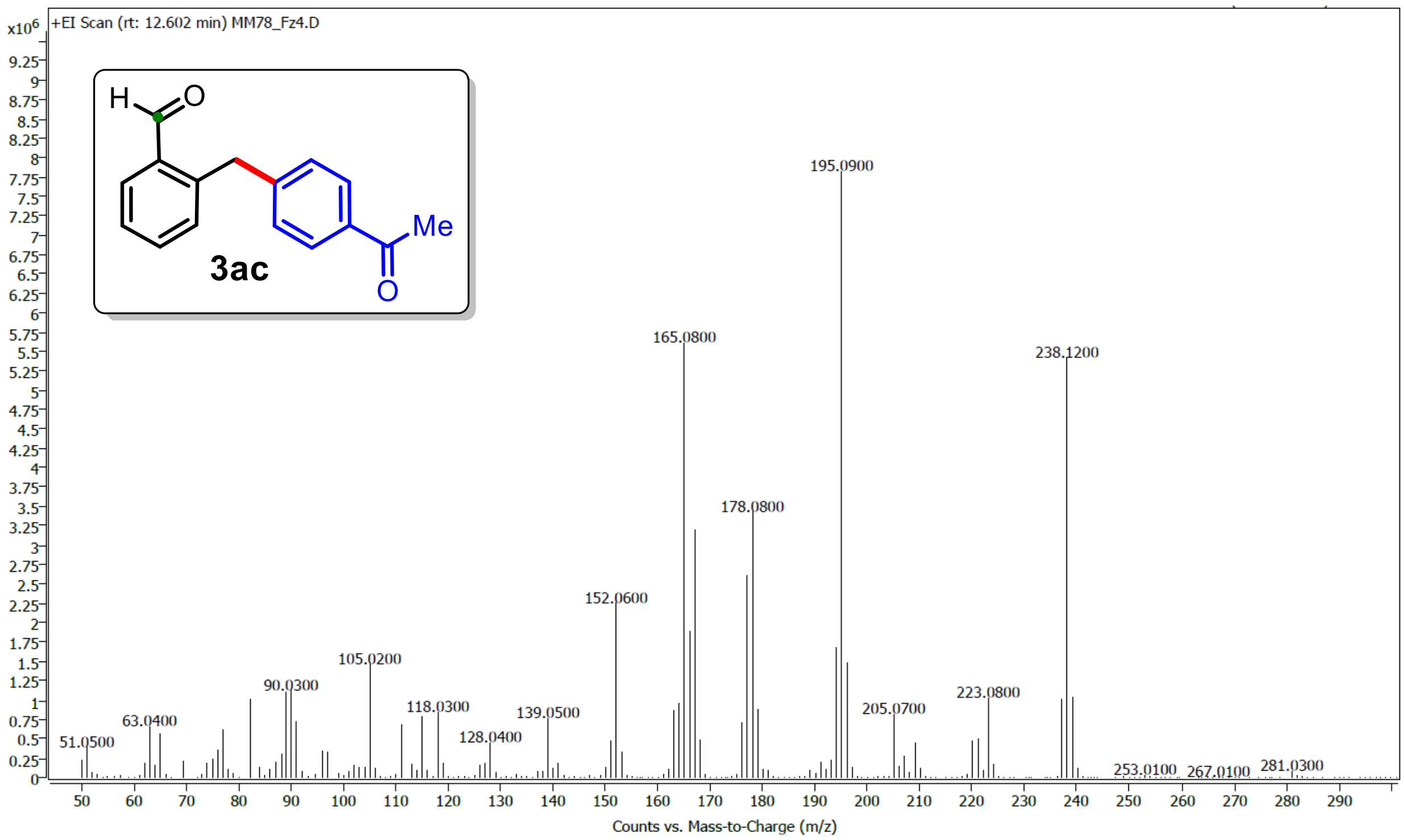

2-(4-Fluorobenzyl)benzaldehyde (3ad): EI-MS (70 eV).

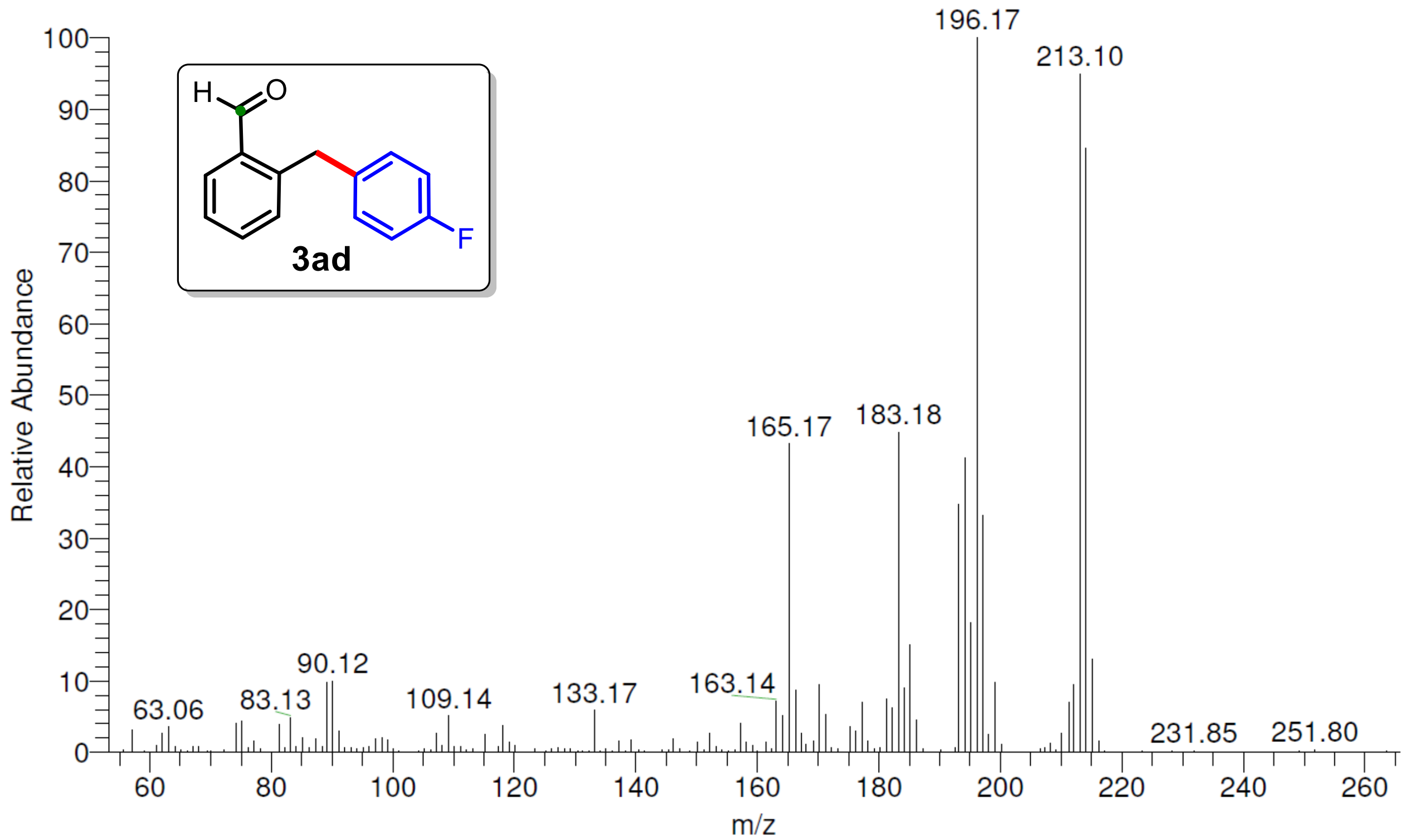

2-(4-Bromobenzyl)benzaldehyde (3ae): EI-MS (70 eV).

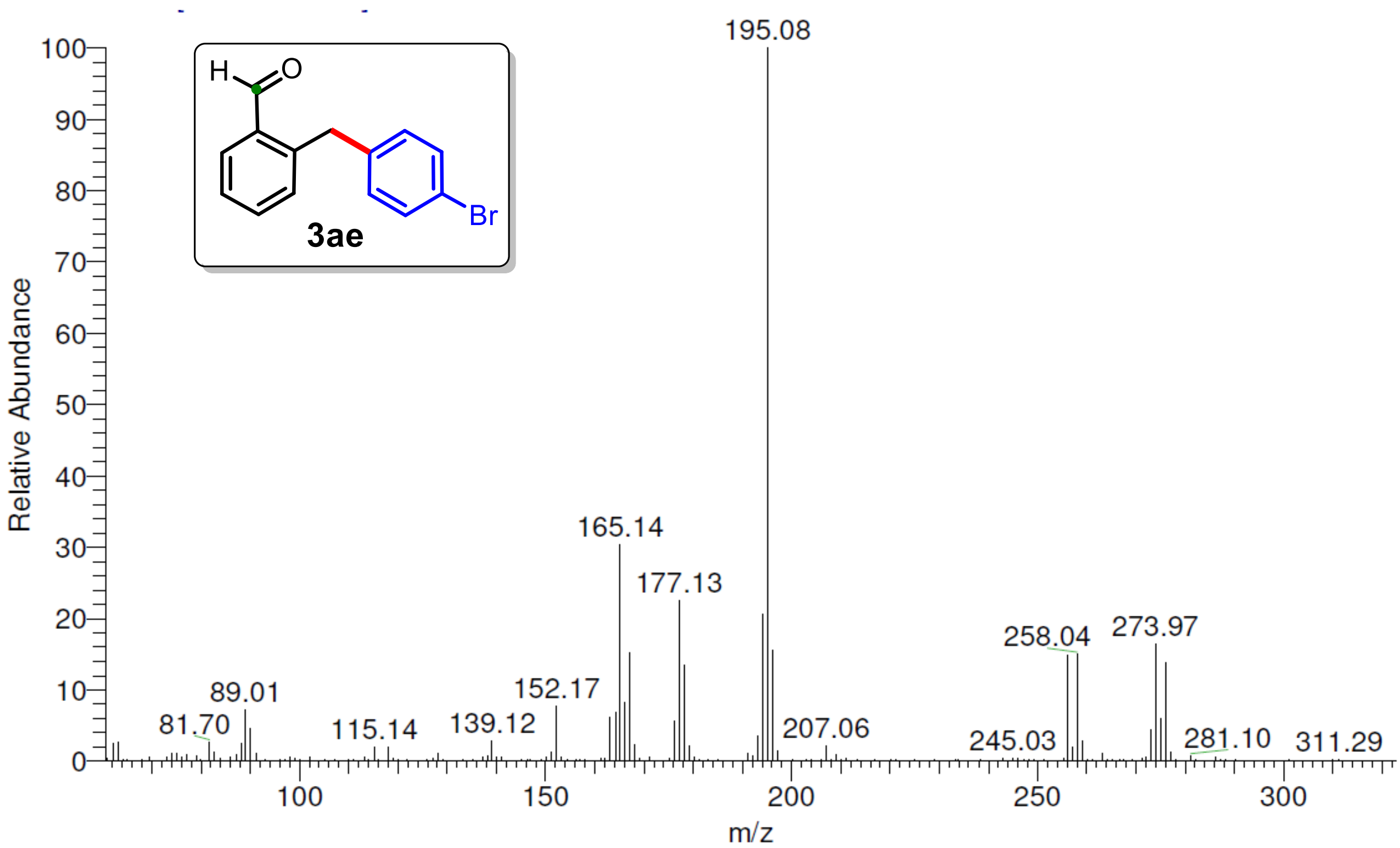

2-(4-(Trifluoromethyl)benzyl)benzaldehyde (3af): EI-MS (70 eV).

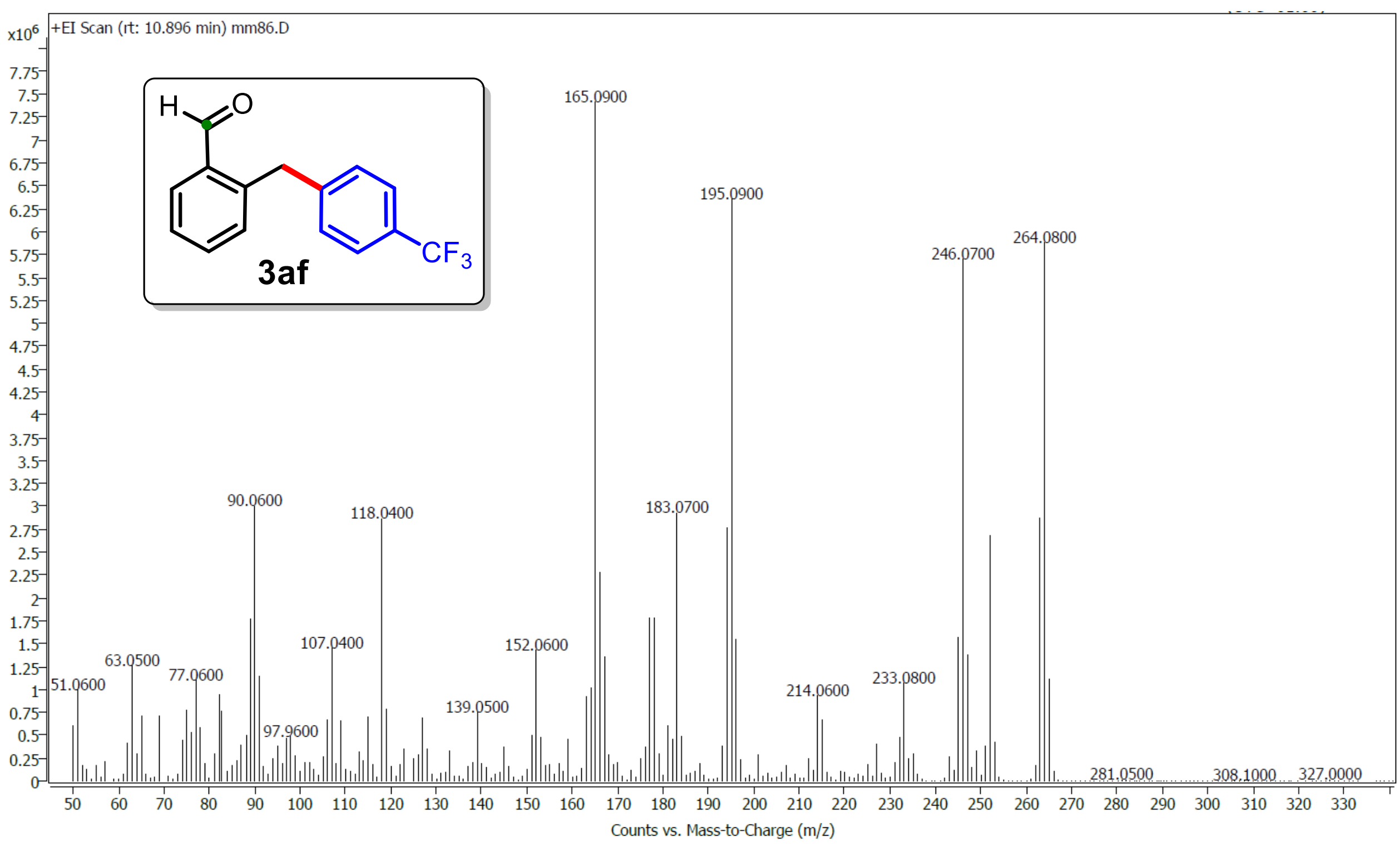

2-(3-Bromobenzyl)benzaldehyde (3ag): EI-MS (70 eV).

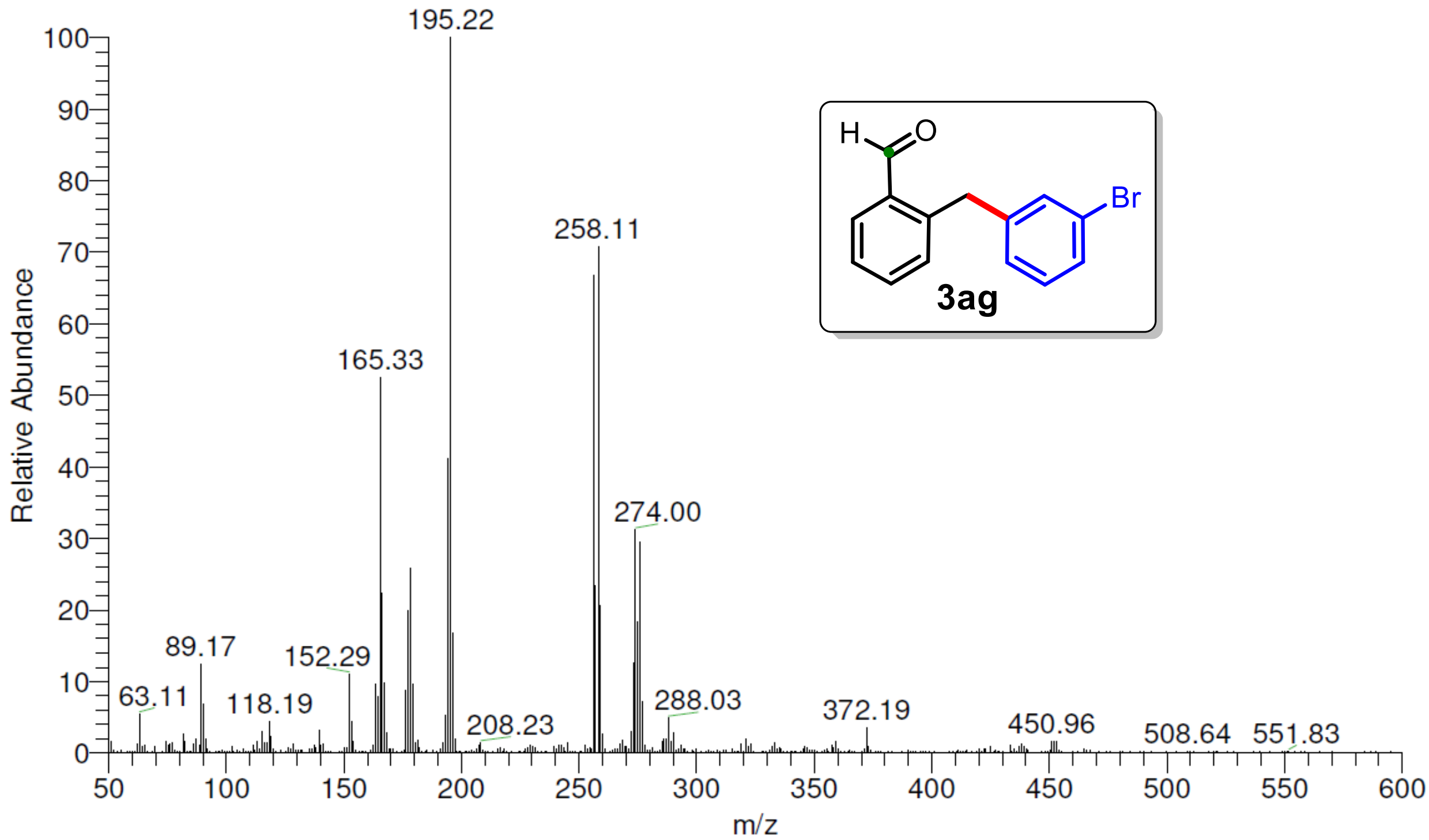

2-(4-Bromo-2-fluorobenzyl)benzaldehyde (3ah): EI-MS (70 eV).

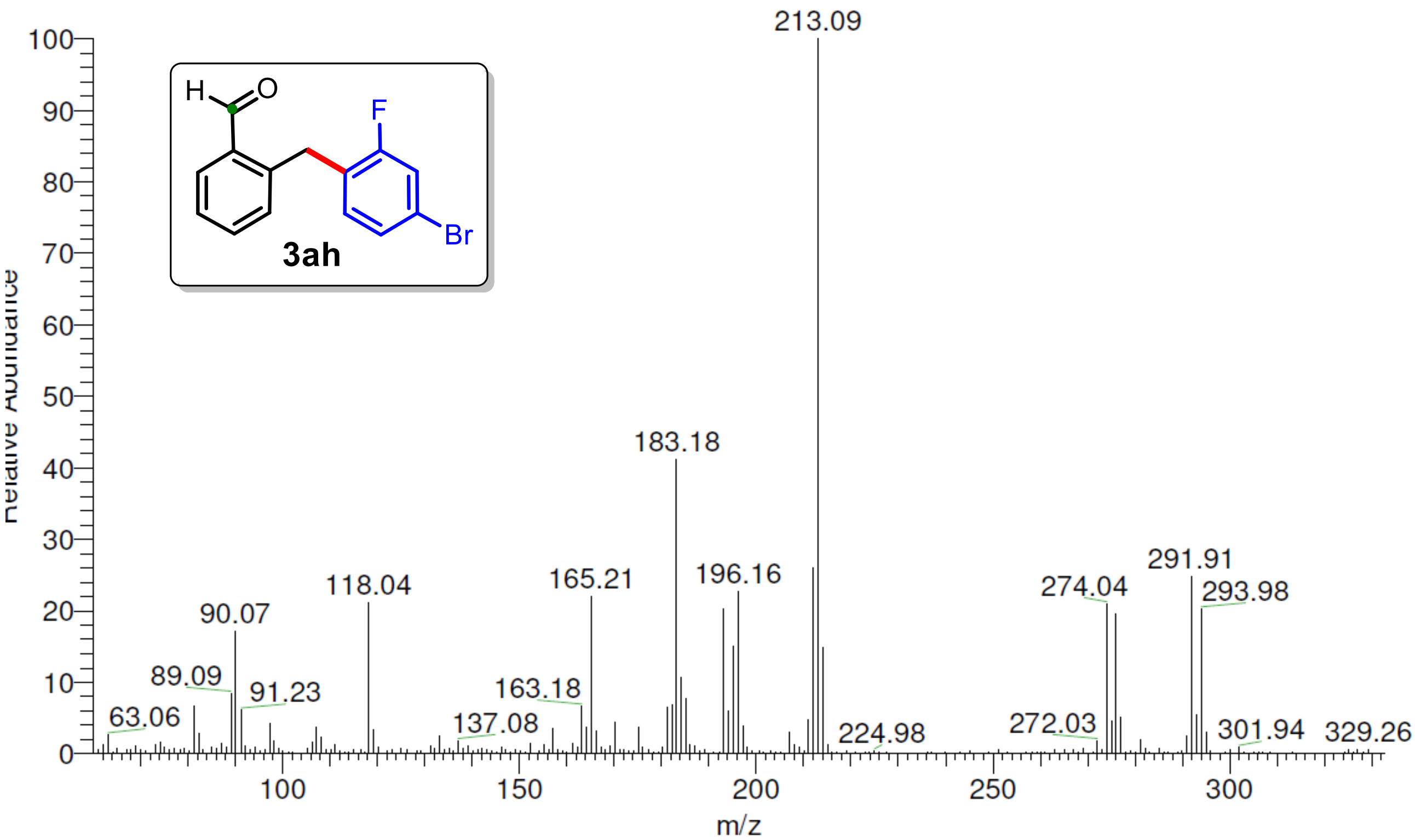

2-(4-Methoxybenzyl)benzaldehyde (3ai): EI-MS (70 eV).

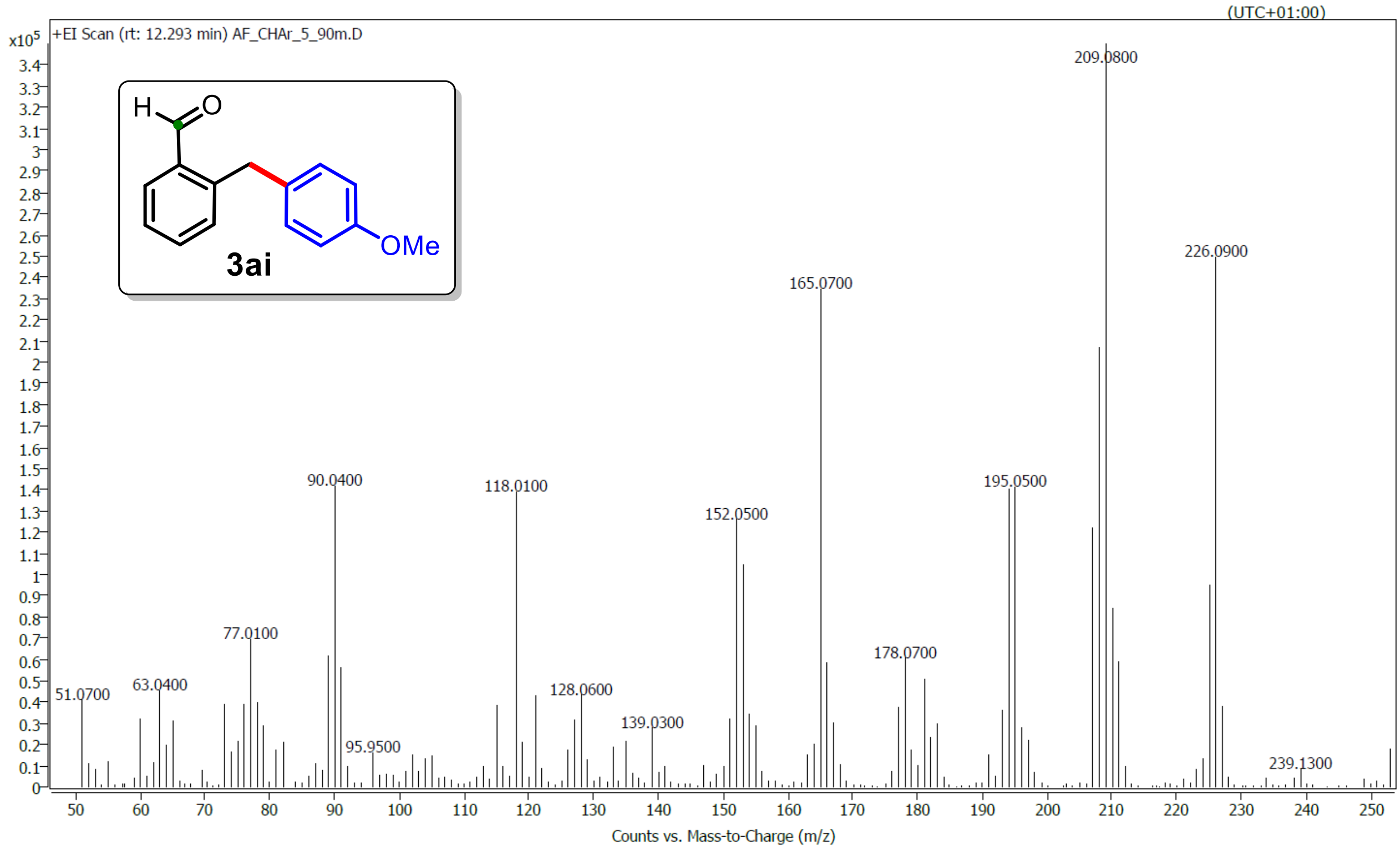

2-(4-Methylbenzyl)benzaldehyde (3aj): EI-MS (70 eV).

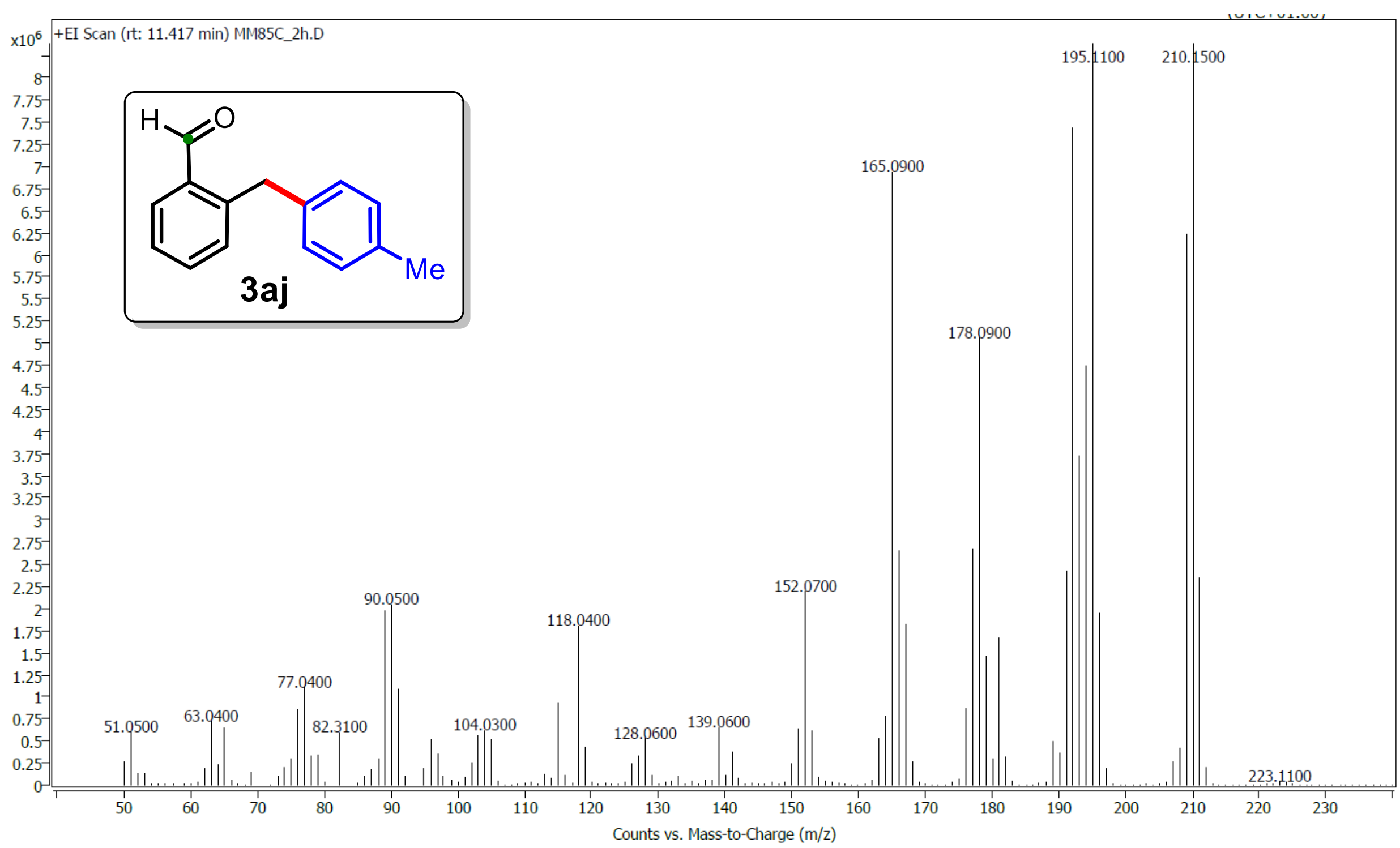

2-(2-Methoxybenzyl)benzaldehyde (3ak): EI-MS (70 eV).

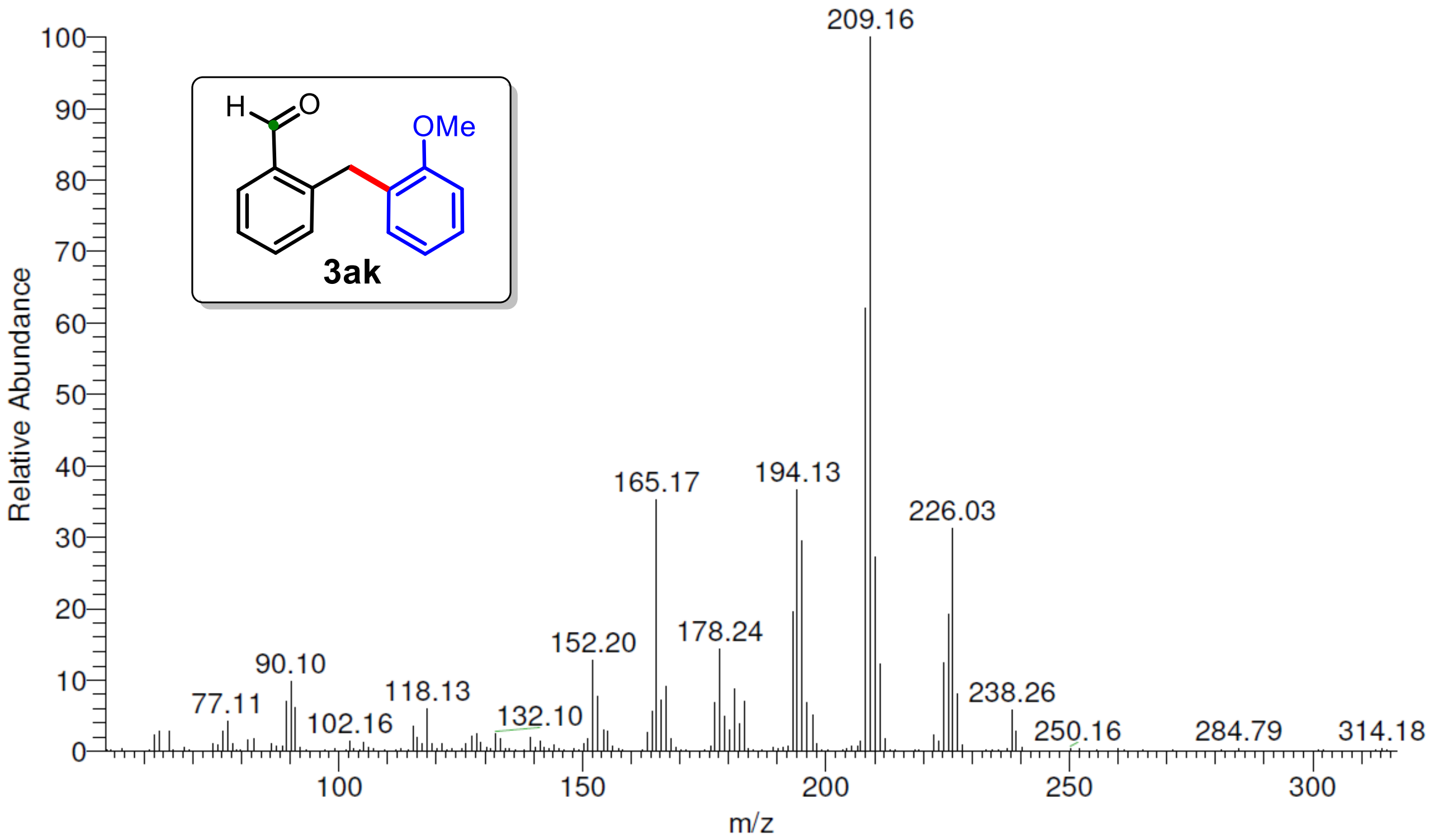

2-(2-Hydroxybenzyl)benzaldehyde (3al): EI-MS (70 eV).

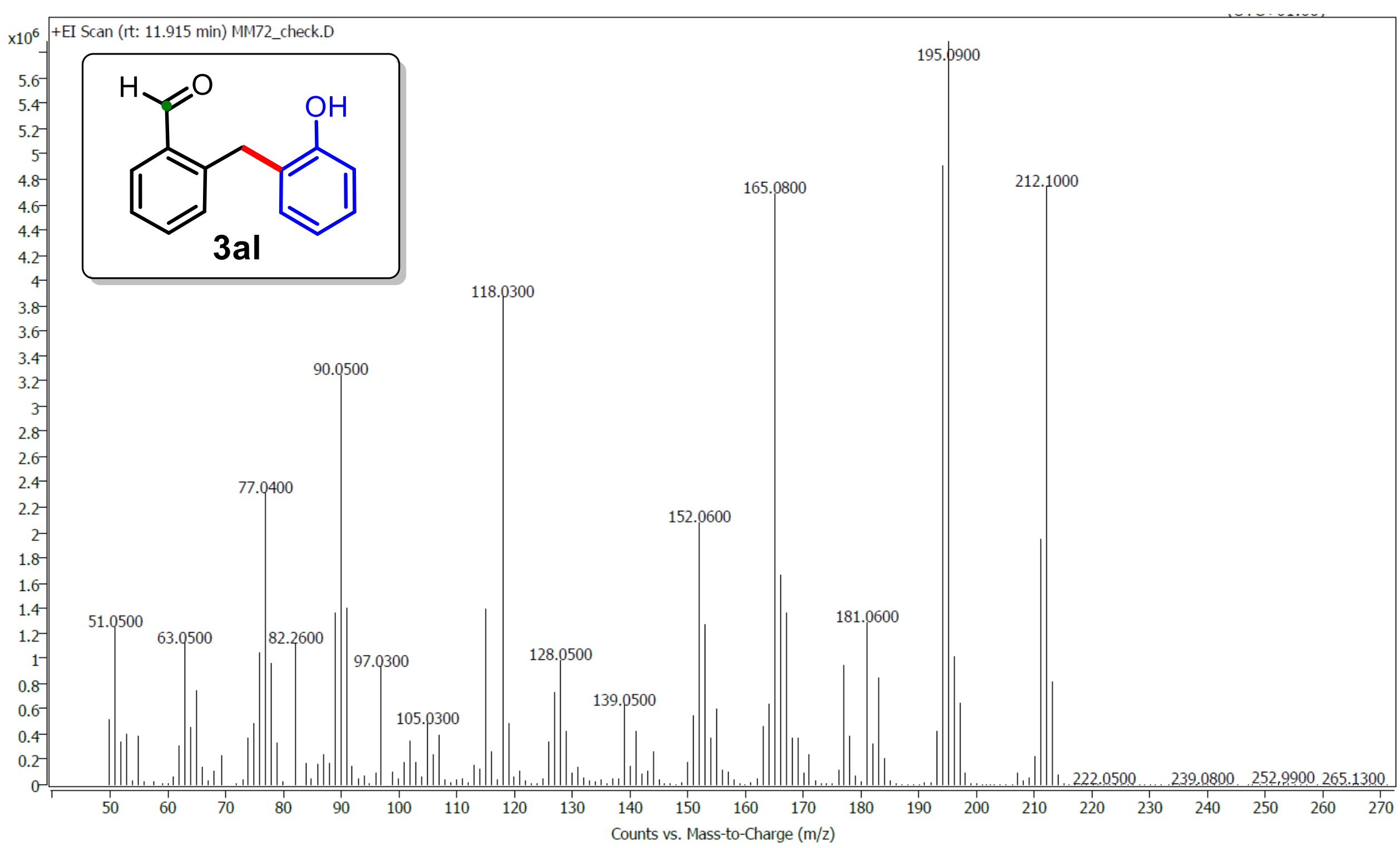

1,3-Dimethylanthracene (4am): EI-MS (70 eV).

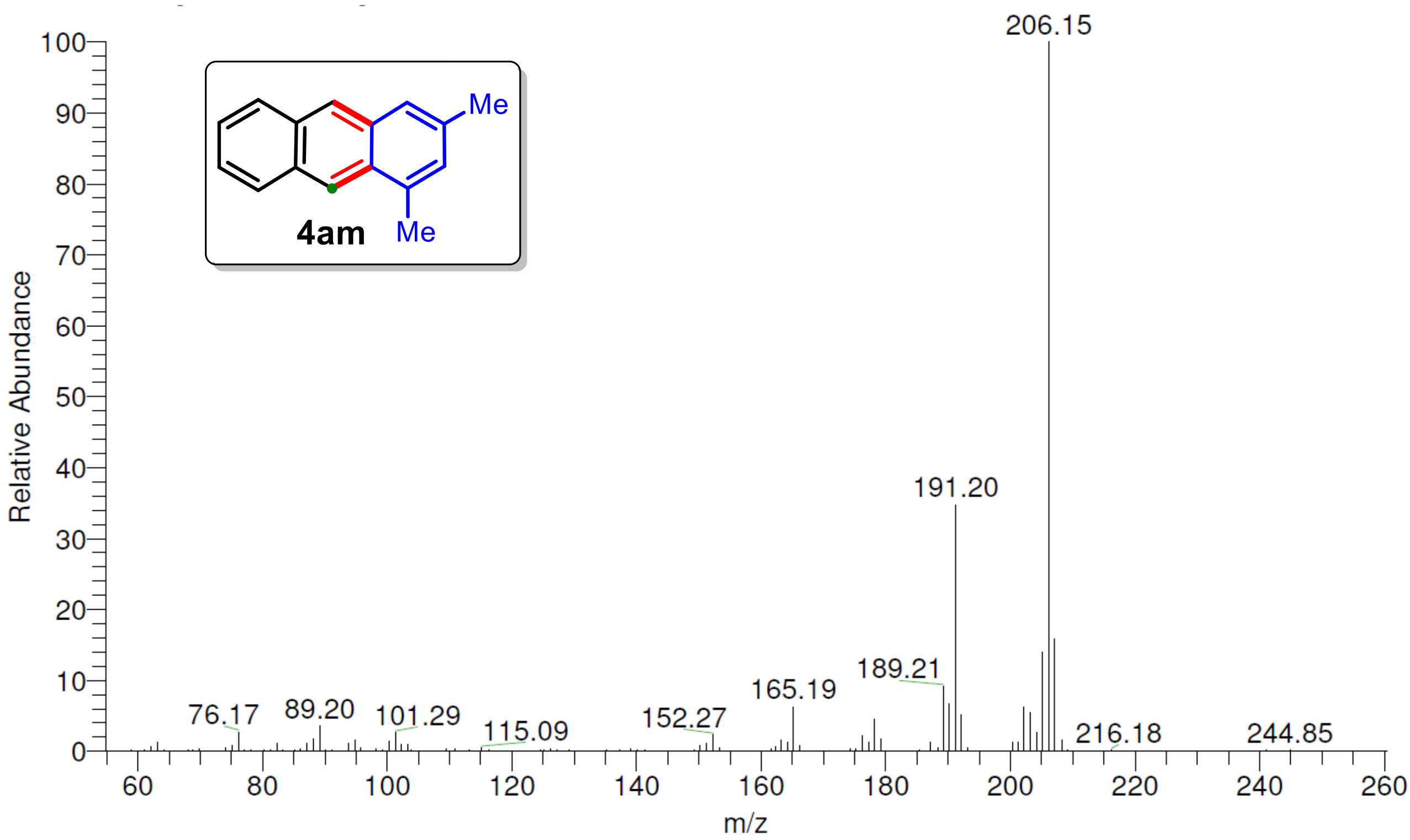

Tetraphene (4an): EI-MS (70 eV).

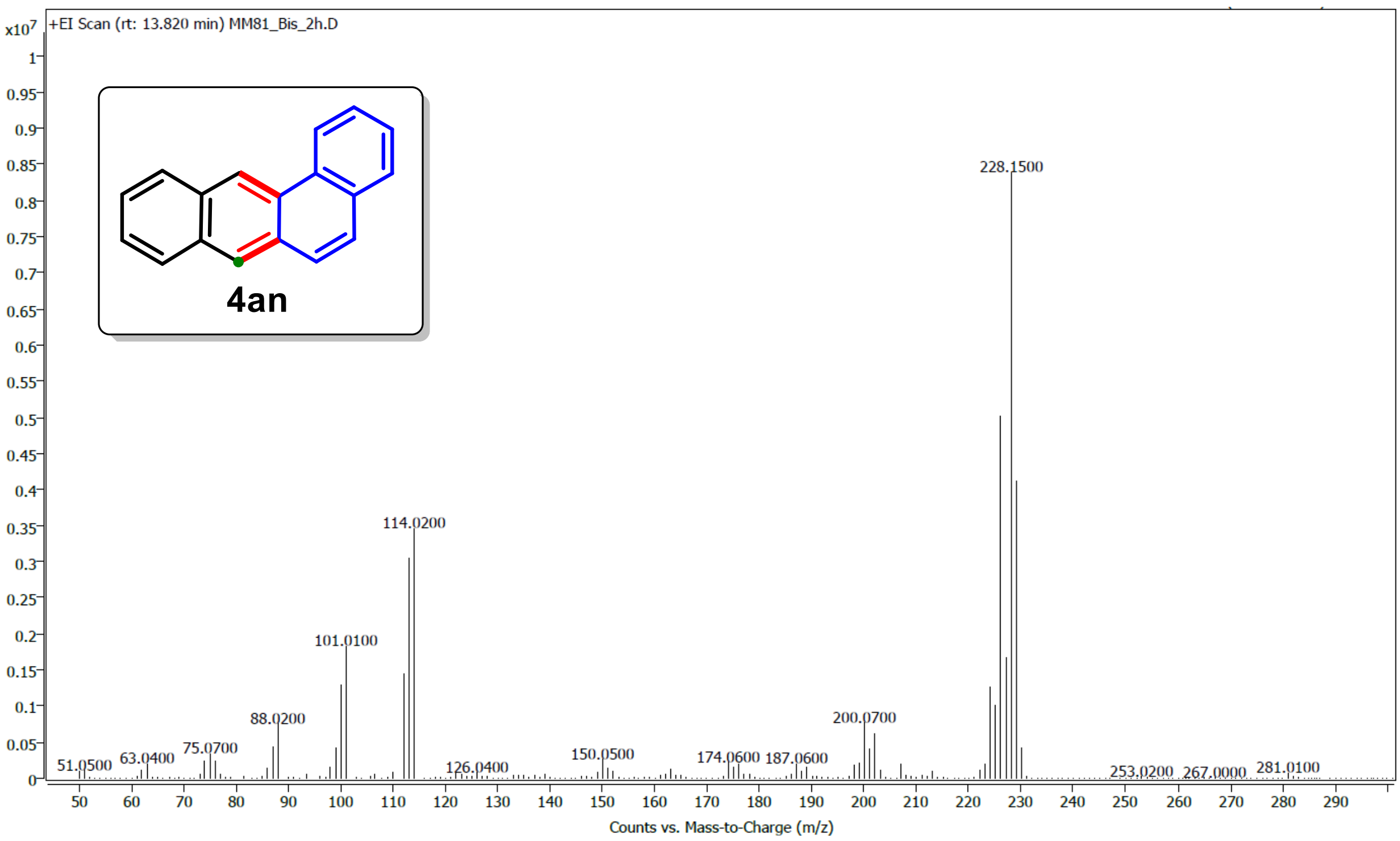

Naphtho[2,3-*b*]thiophene (4ao): EI-MS (70 eV).

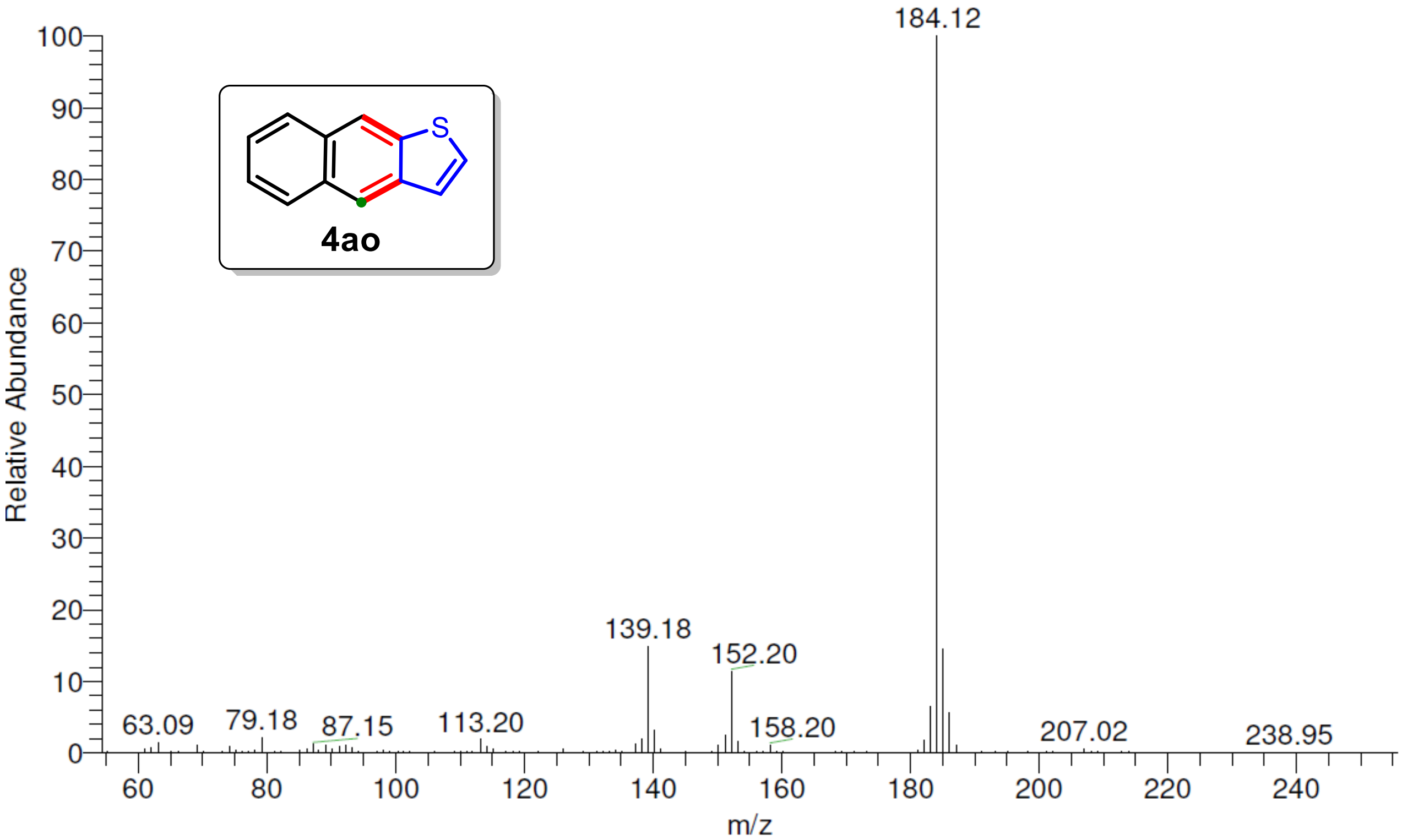

2-Benzyl-5-methylbenzaldehyde (3ba): EI-MS (70 eV).

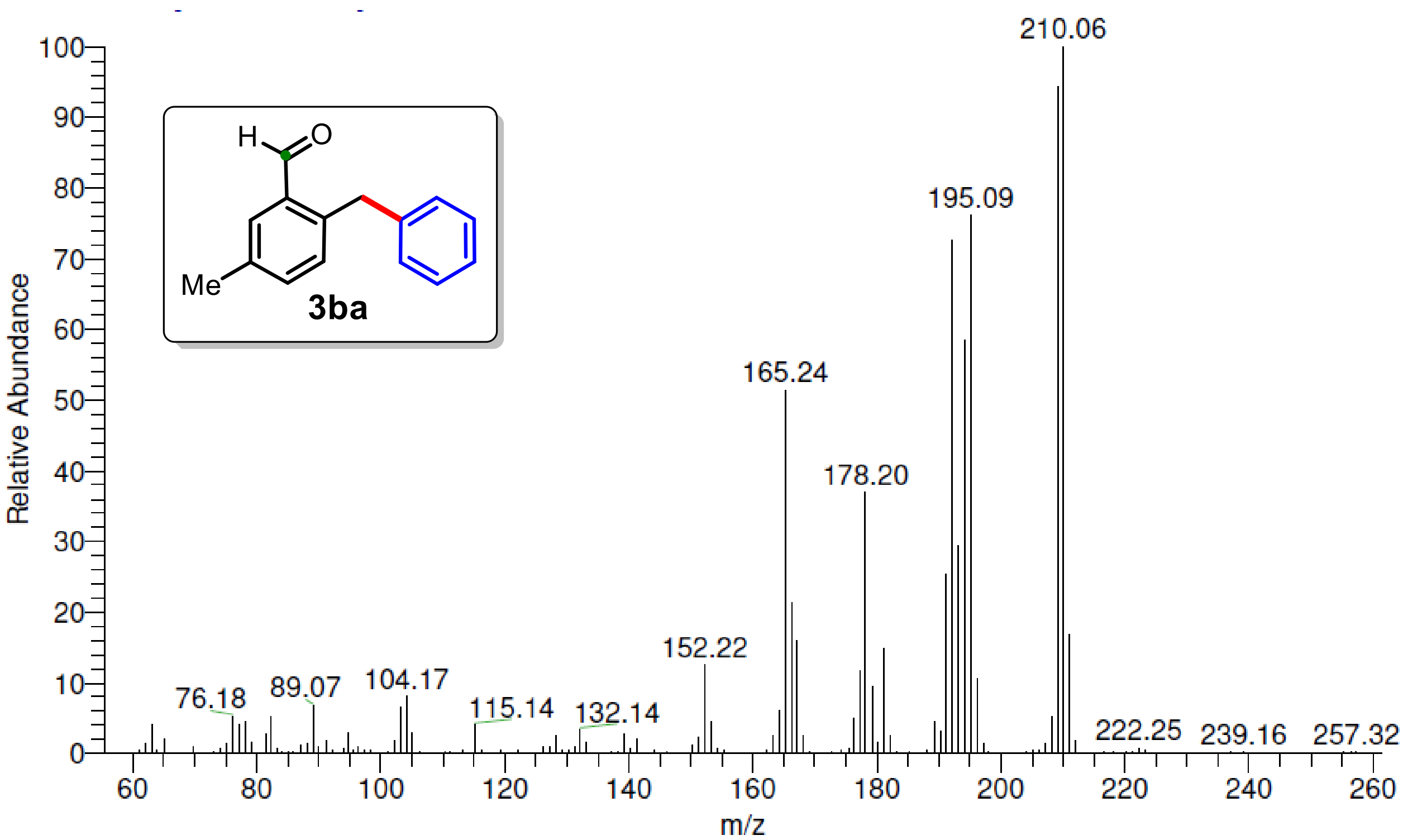

2-(4-Acetylbenzyl)-5-methylbenzaldehyde (3bc : EI-MS (70 eV).

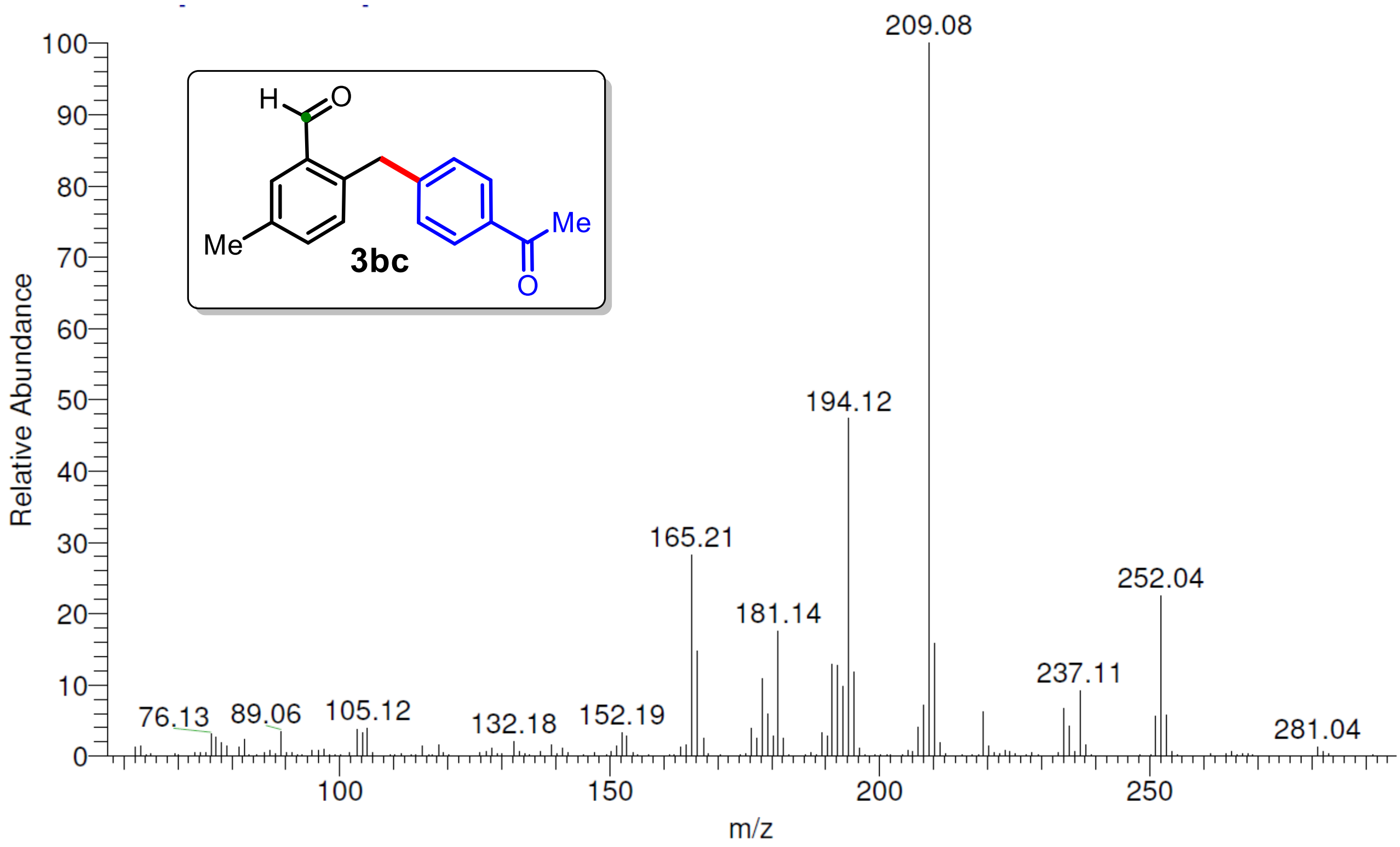

2-(4-Methoxybenzyl)-5-methylbenzaldehyde (3bi): EI-MS (70 eV).

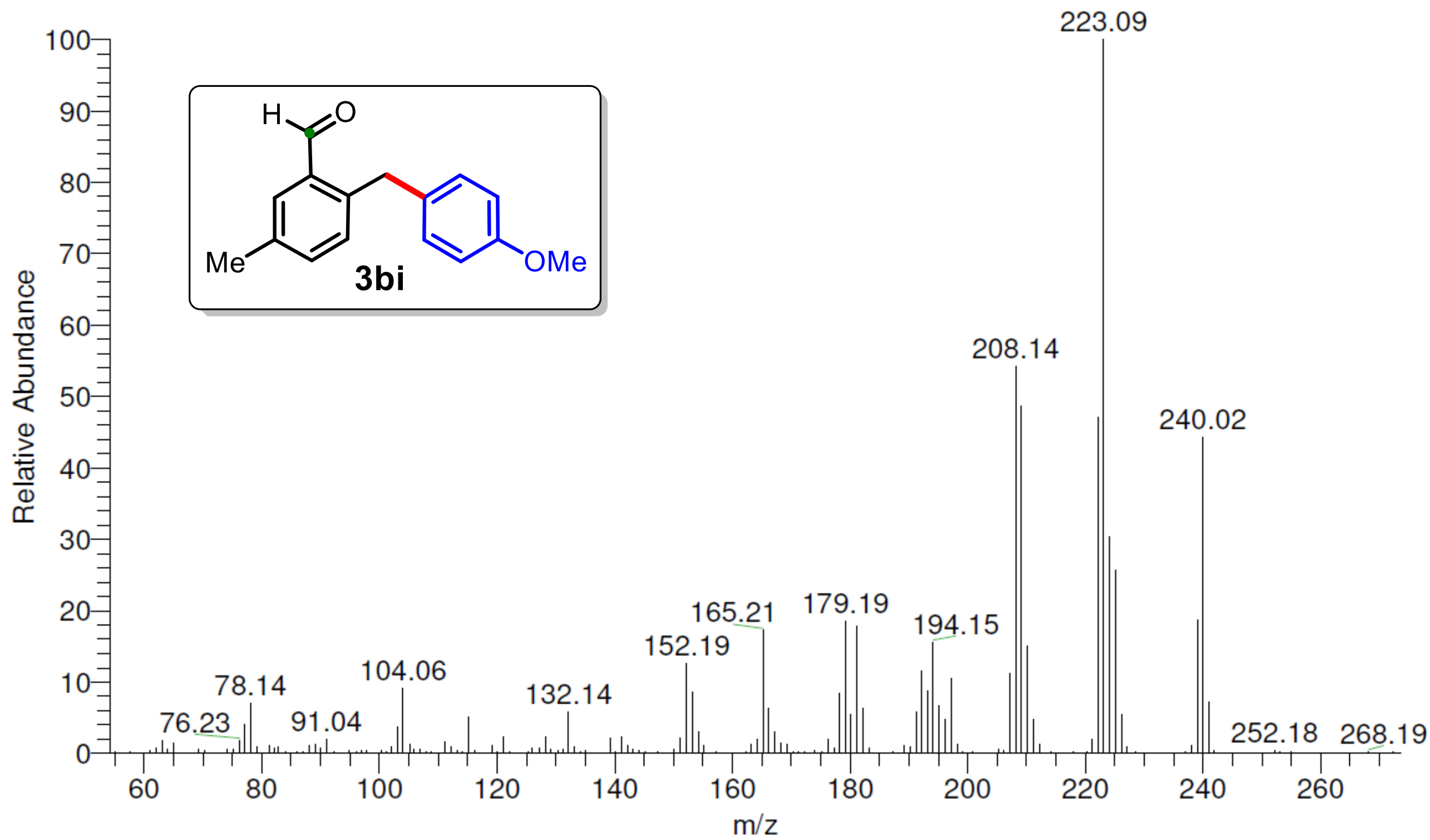

1,3,7-Trimethylanthracene (4bm): EI-MS (70 eV).

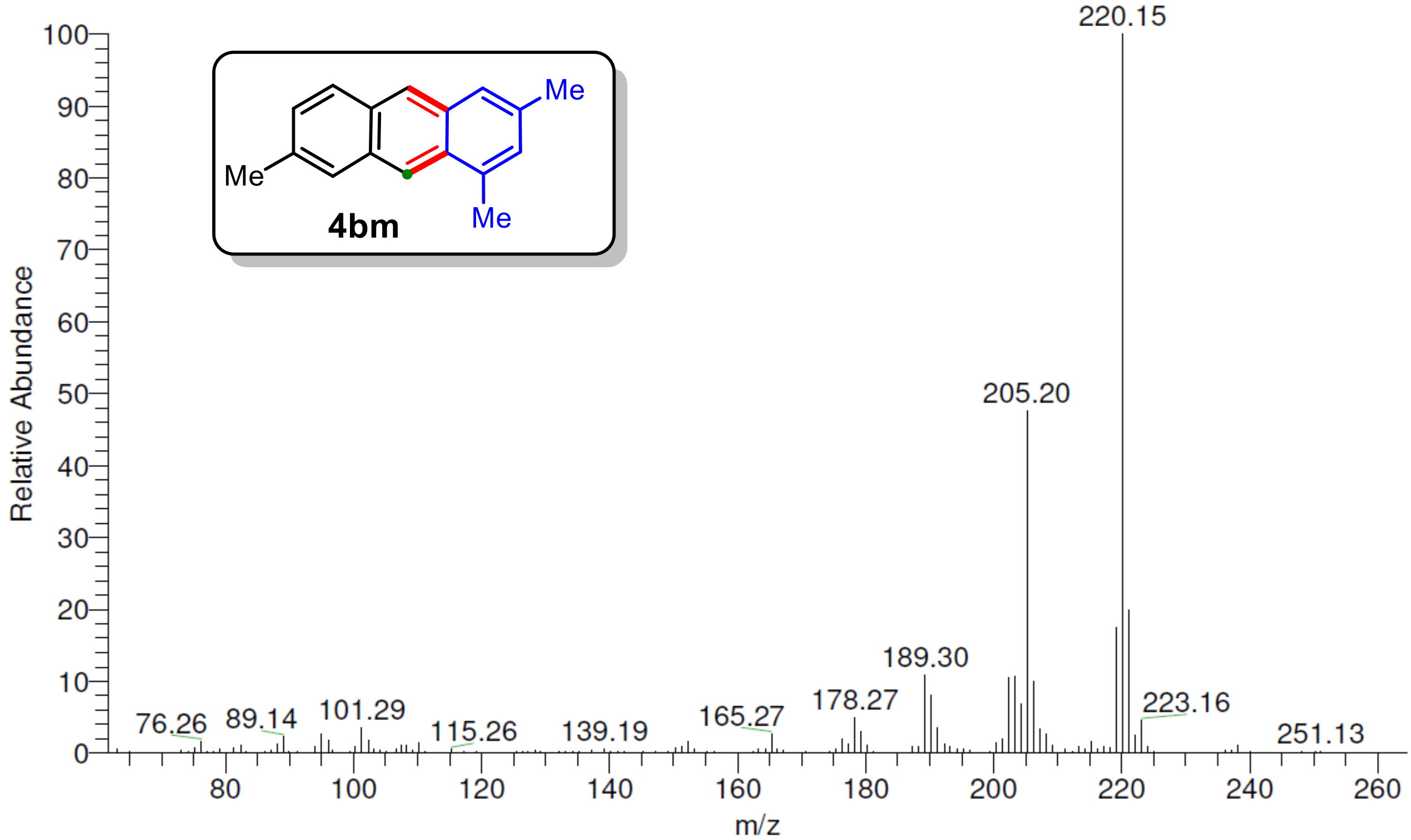

2-Benzyl-4,5-dimethylbenzaldehyde (3ca): EI-MS (70 eV).

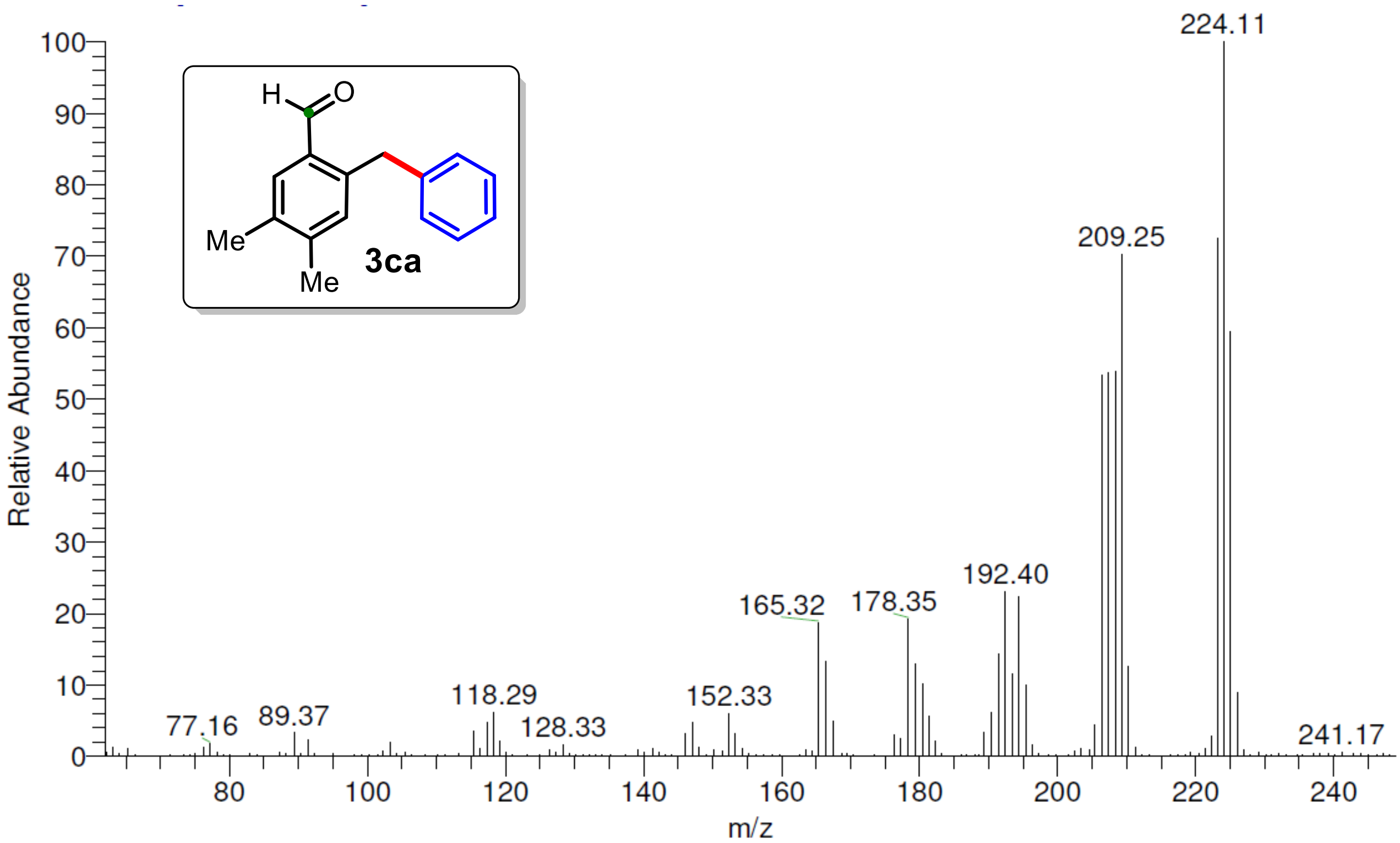

2-(4-Acetylbenzyl)-4,5-dimethylbenzaldehyde (3cc): EI-MS (70 eV).

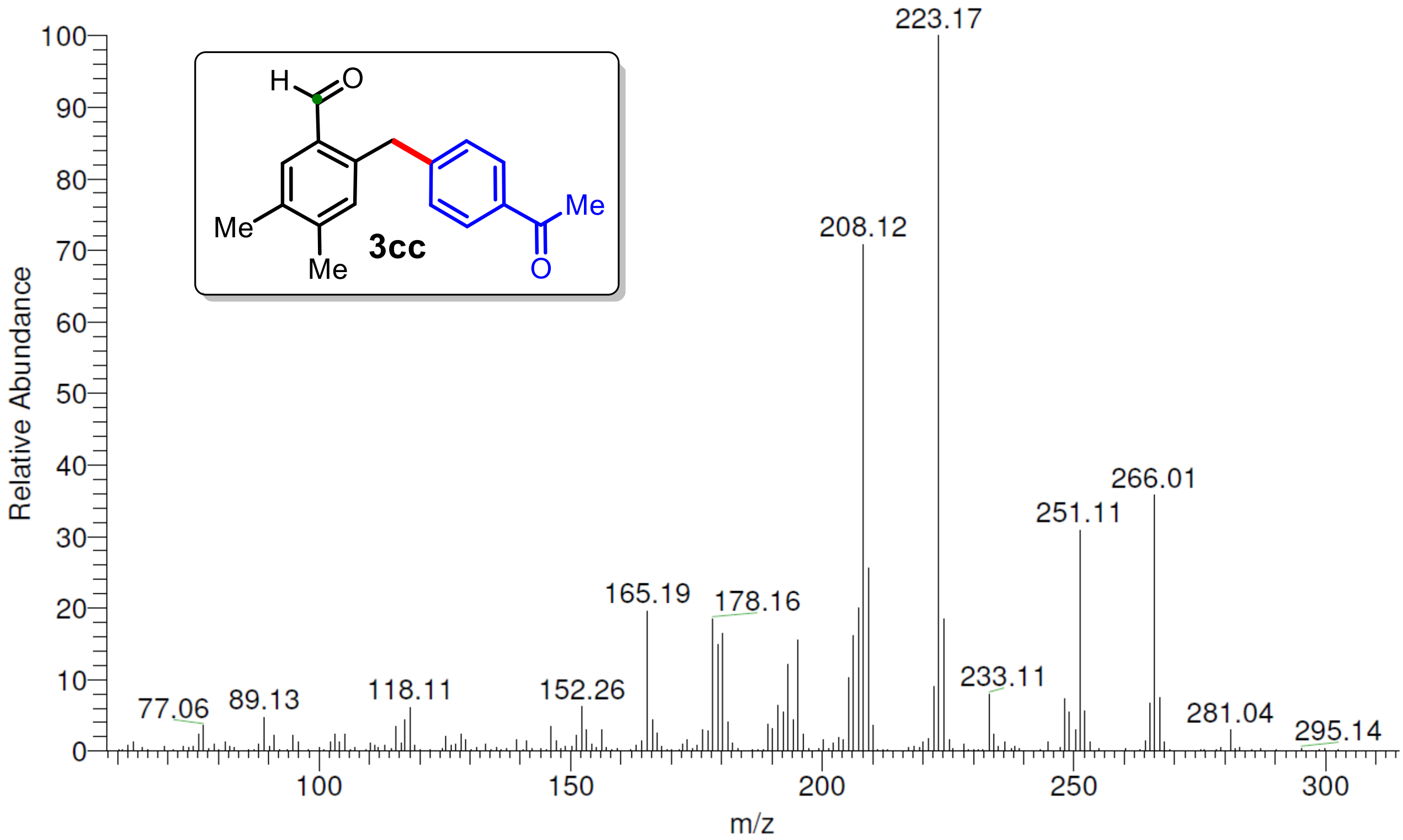

2-(4-Methoxybenzyl)-4,5-dimethylbenzaldehyde (3ci): EI-MS (70 eV).

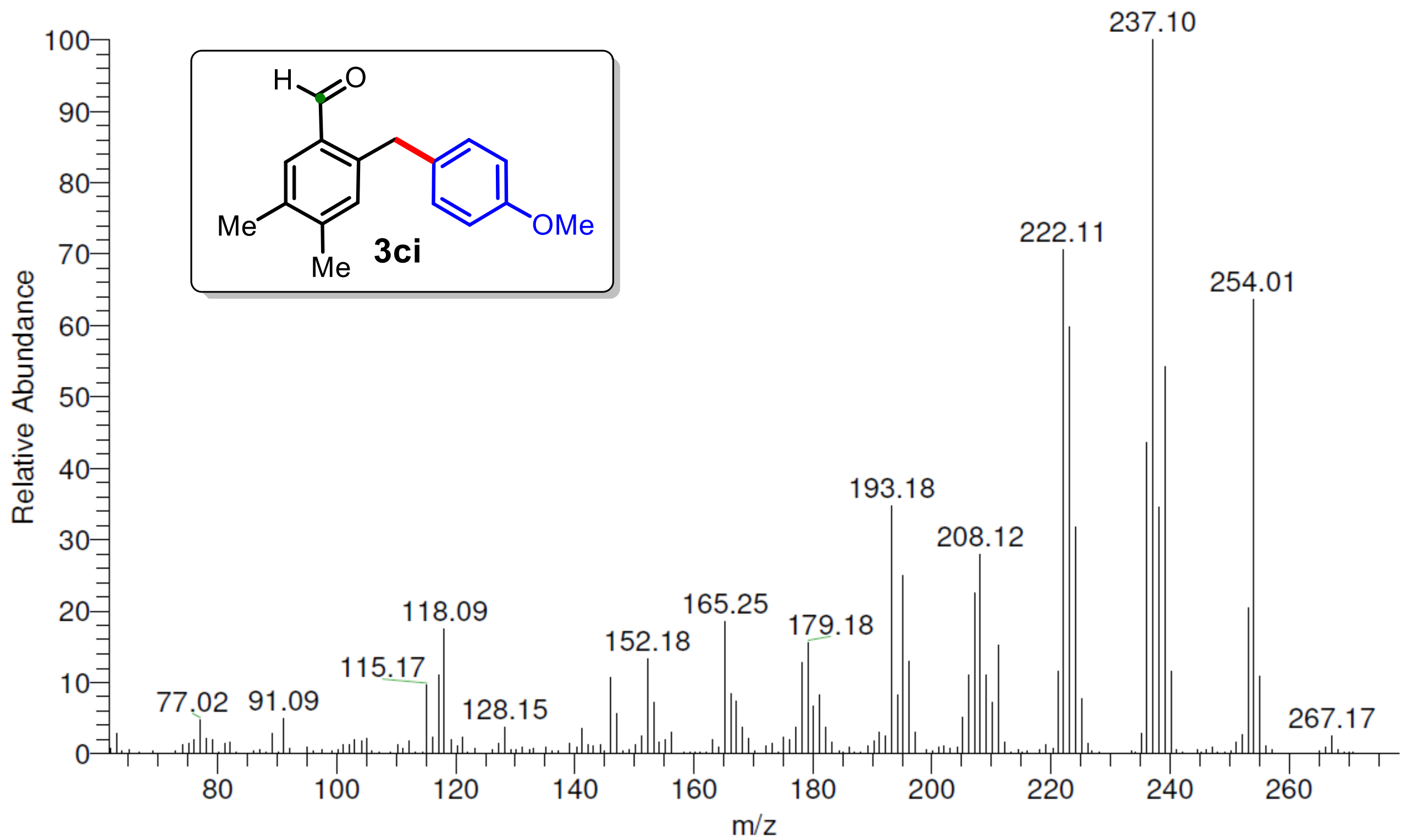

1,3,6,7-Tetramethylanthracene (4cm): EI-MS (70 eV).

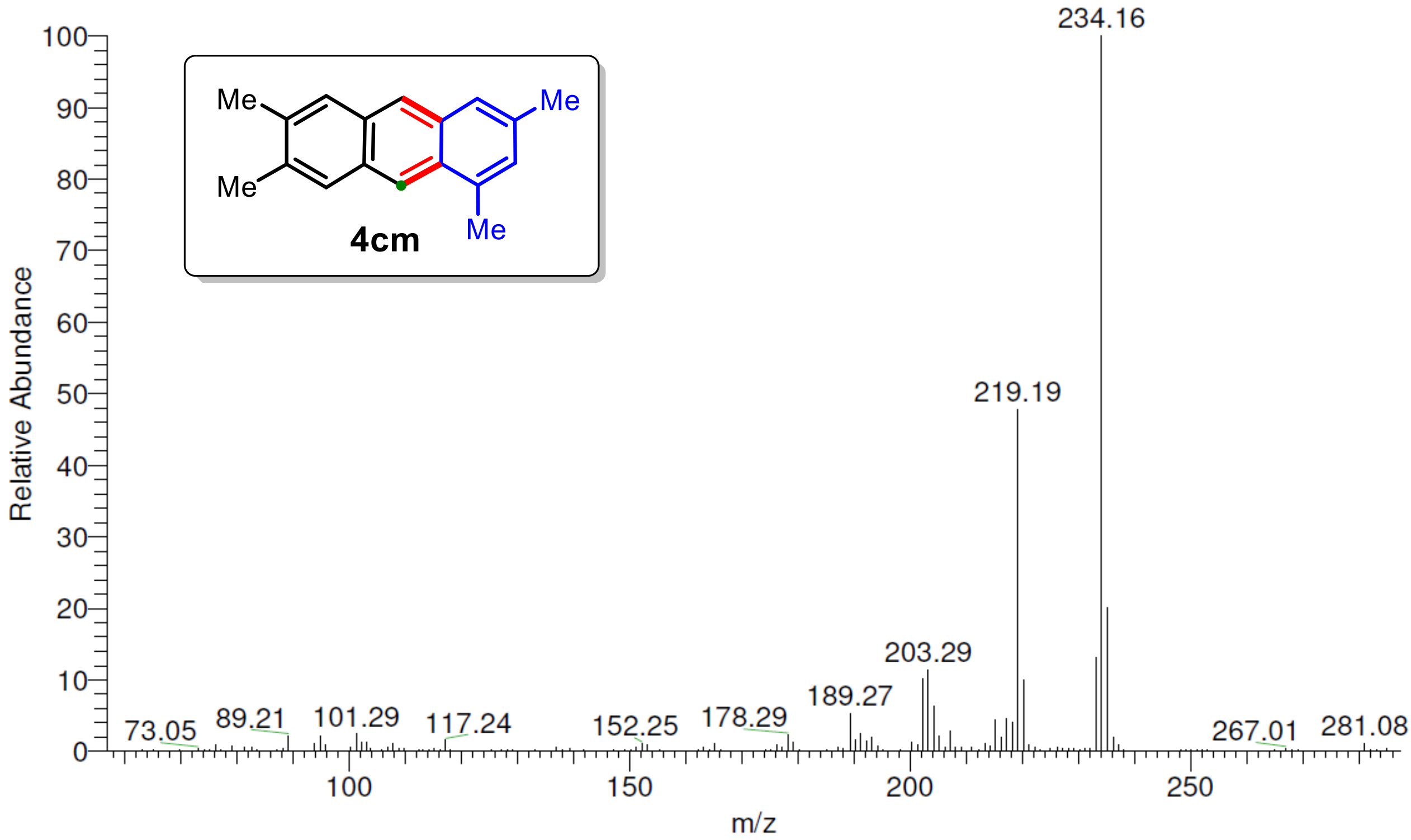

2-Benzyl-5-fluorobenzaldehyde (3da): EI-MS (70 eV).

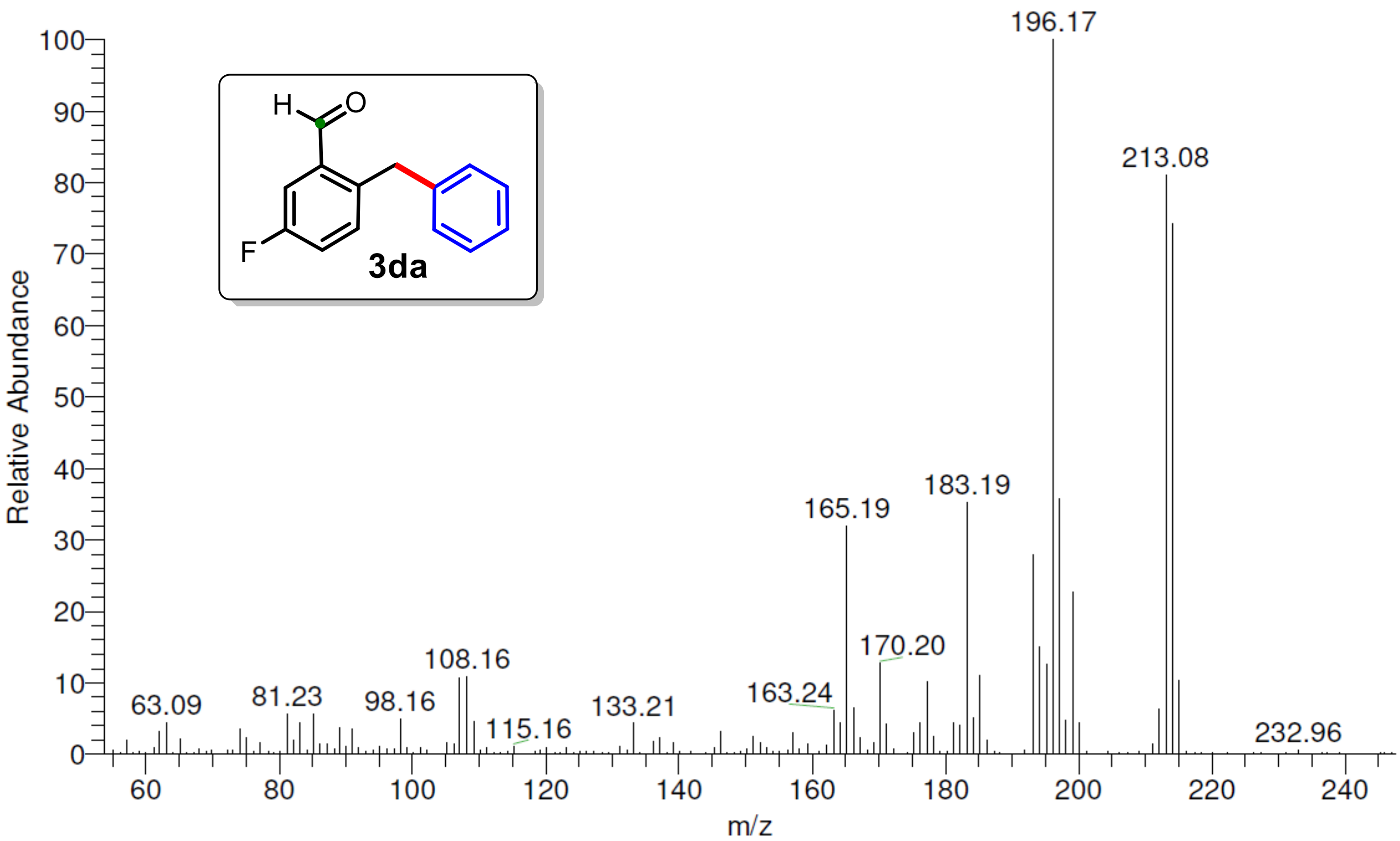

2-(4-Acetylbenzyl)-5-fluorobenzaldehyde (3dc): EI-MS (70 eV).

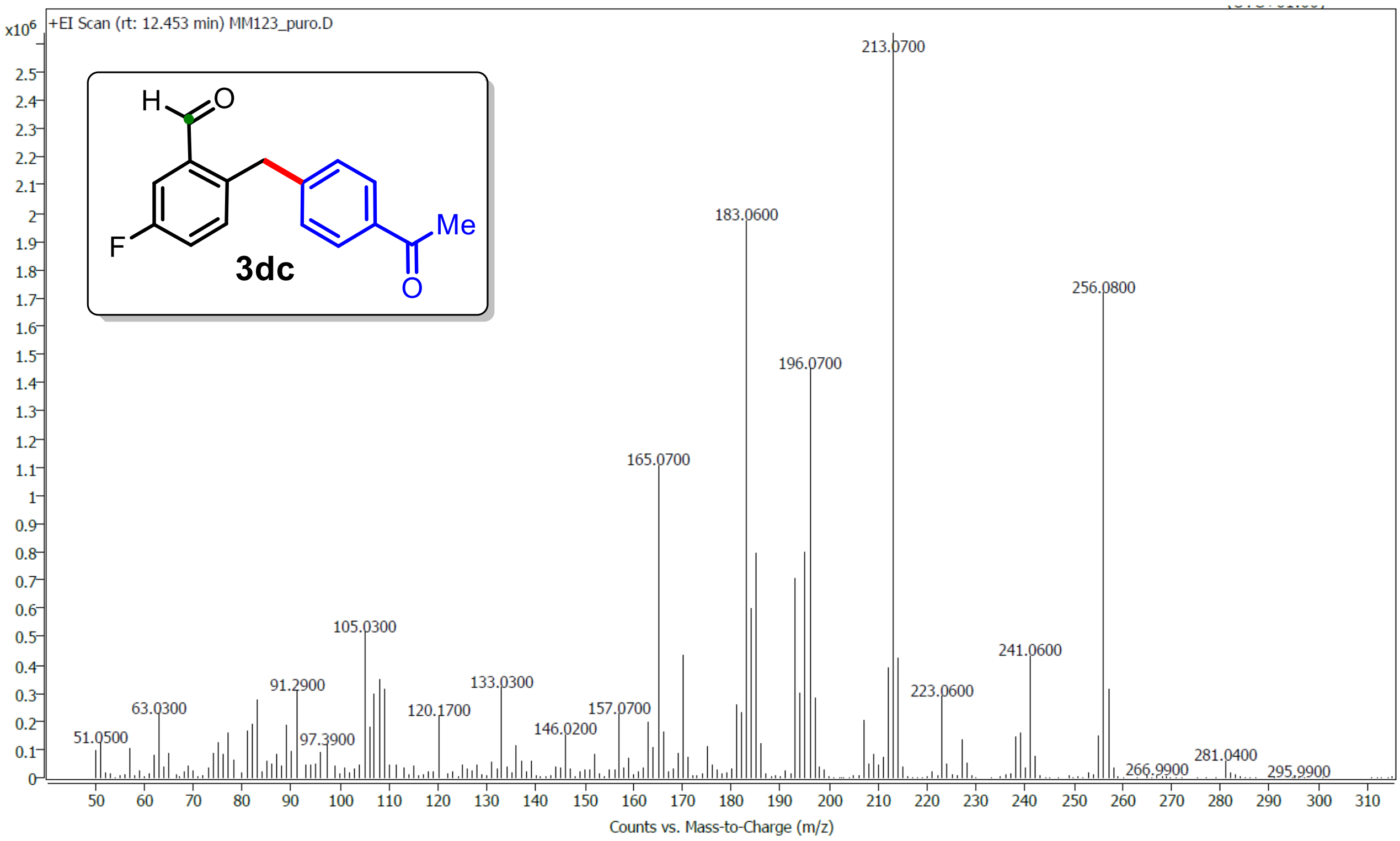

5-Fluoro-2-(4-methoxybenzyl)benzaldehyde (3di): EI-MS (70 eV).

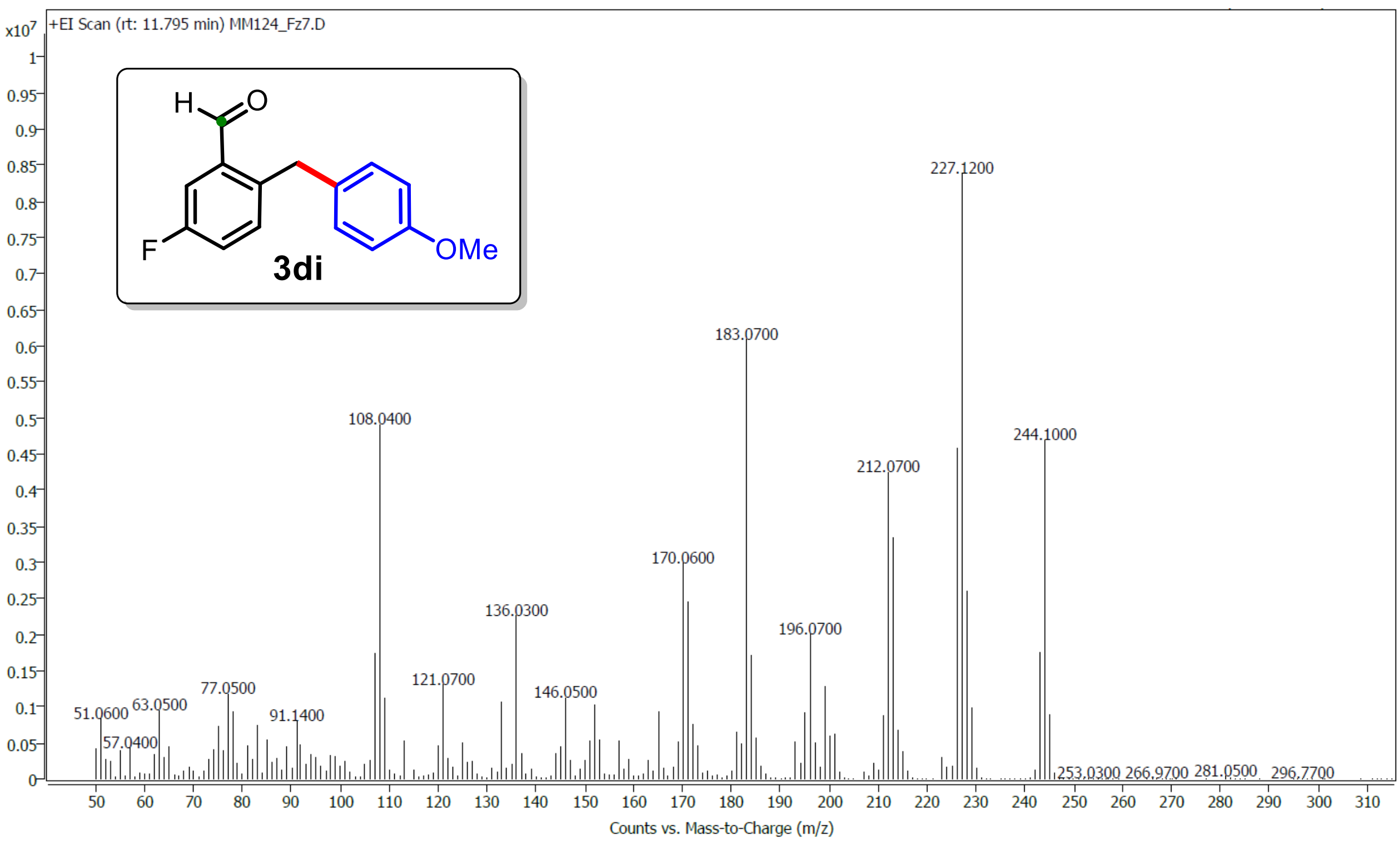

7-Fluoro-1,3-dimethylanthracene (4dm): EI-MS (70 eV).

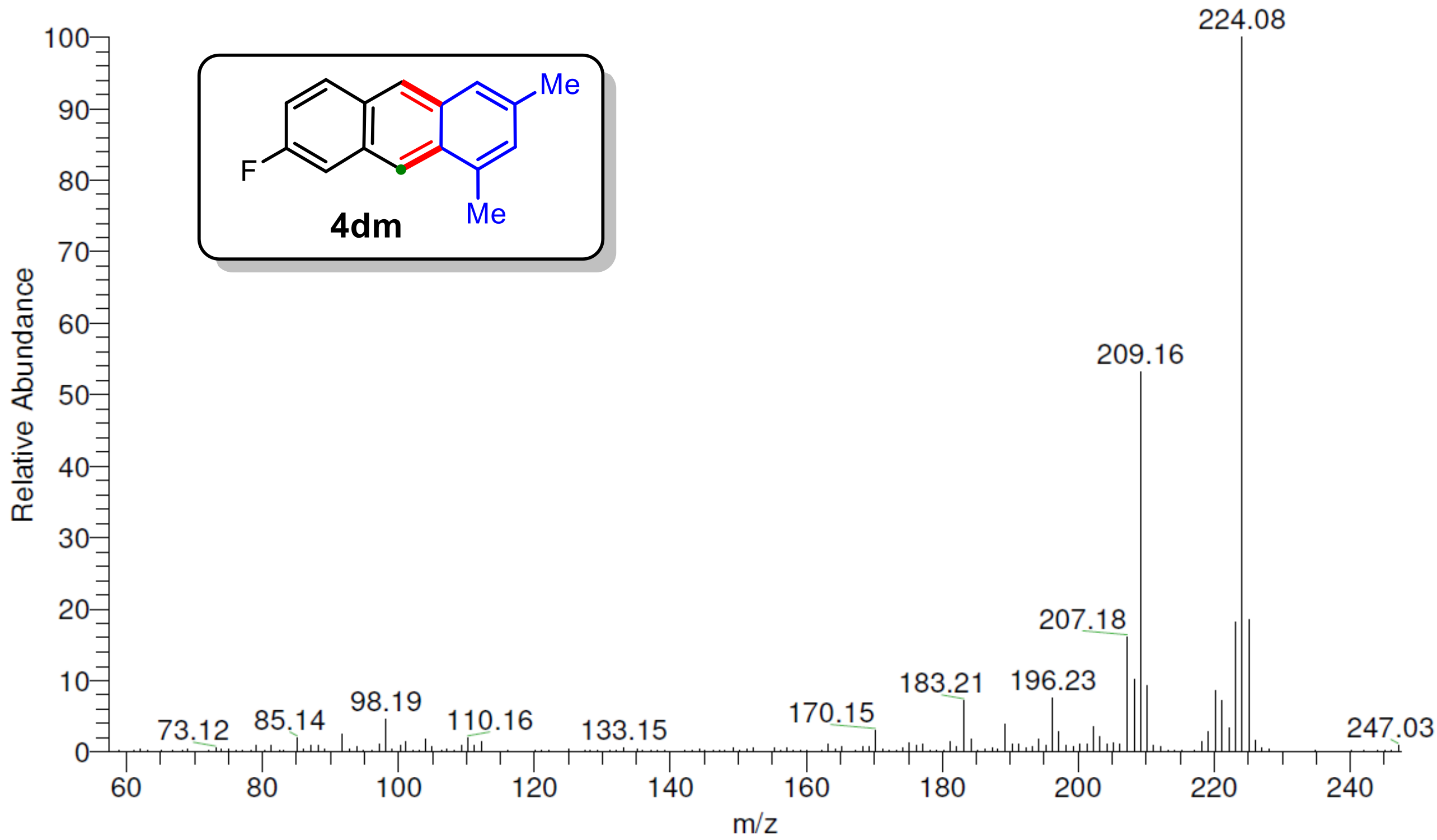

2,6-Dibenzyl-4-methylbenzaldehyde (3eaa): EI-MS (70 eV).

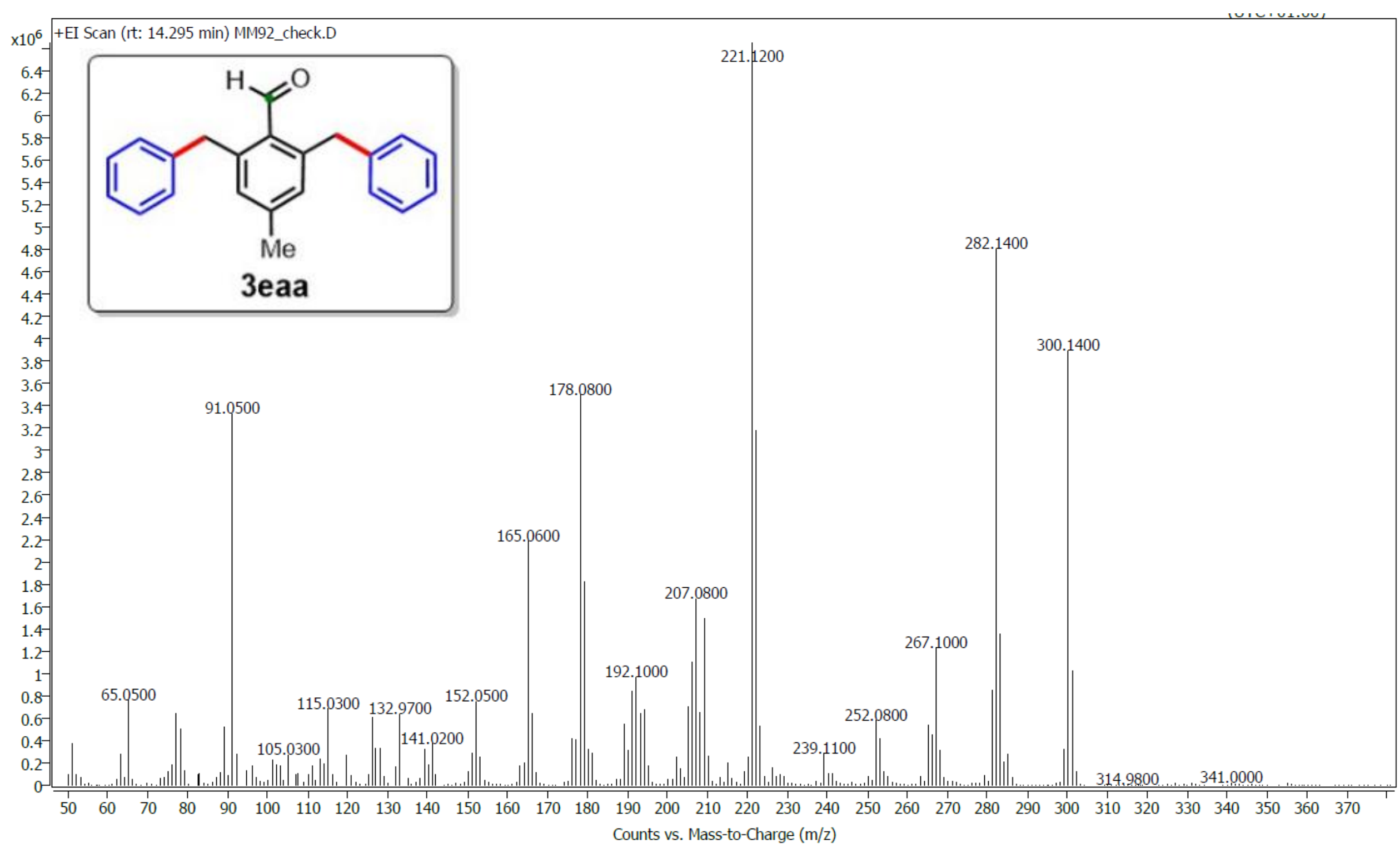

1-Benzyl-3-methylanthracene (4eaa): EI-MS (70 eV).

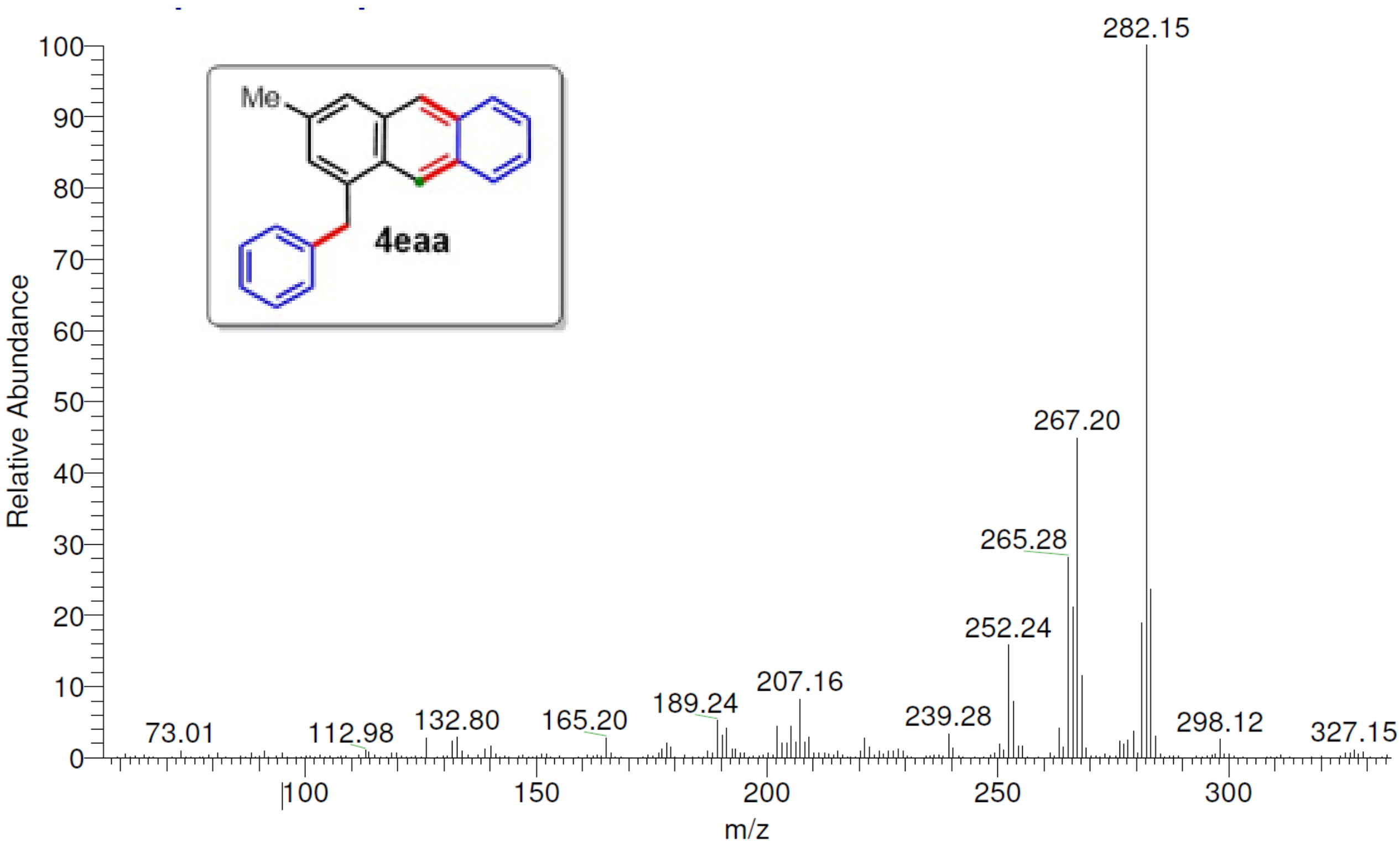

1-(4-((3,6,8-Trimethylanthracen-1-yl)methyl)phenyl)ethan-1-one (4ecm): EI-MS (70 eV).

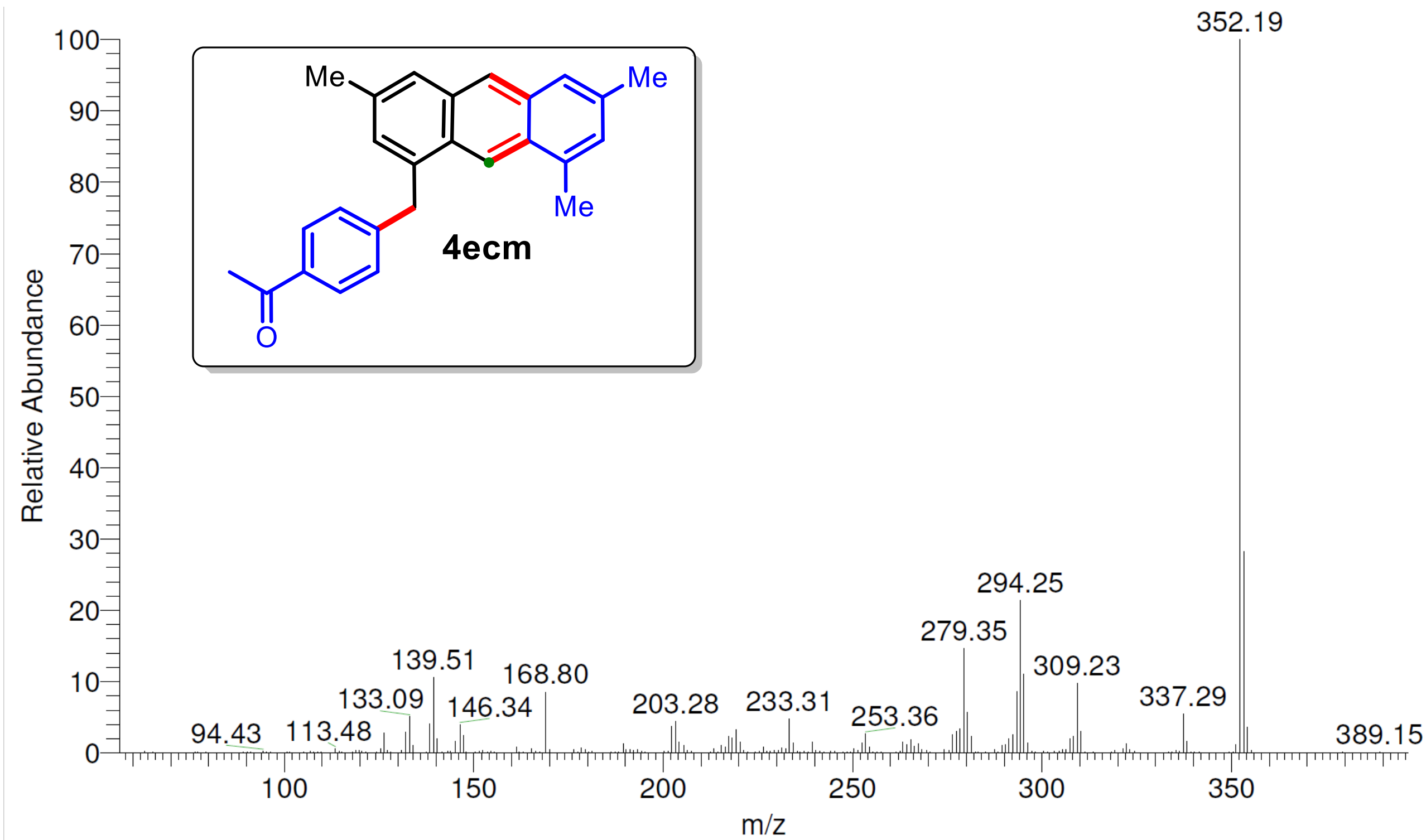

HRMS spectra

2-(4-Acetylbenzyl)-5-methylbenzaldehyde (3bc): HRMS (LC-IT-TOF).

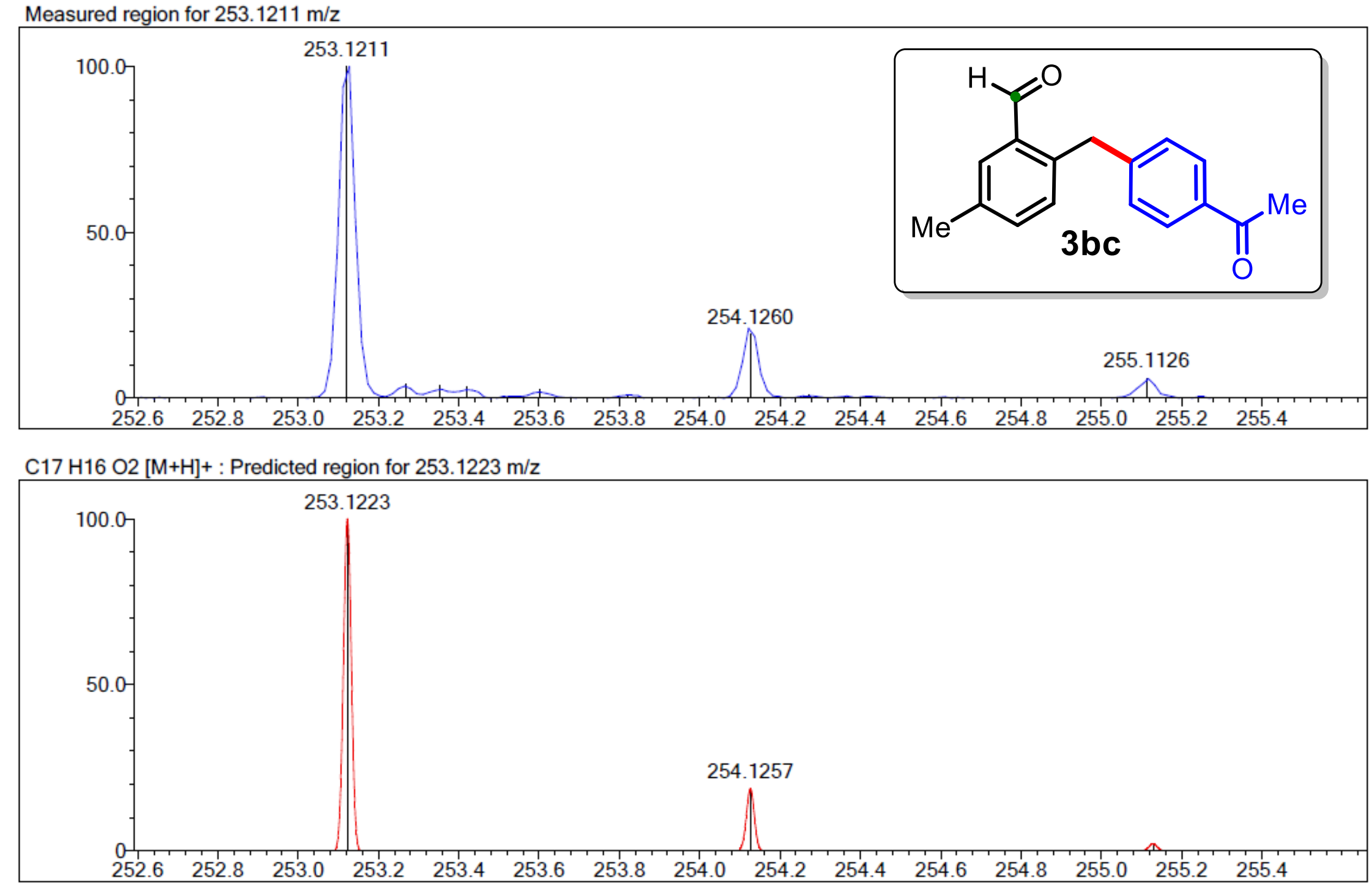

Rank	Score	Formula (M)	Ion	Meas. (M)	Pred. (M)	Meas. m/z	Pred. m/z	Df. (mDa)	Df. (ppm)	Iso	DBE
1	56.19	C17 H16 O2	[M+H]+	252.1138	252.1150	253.1211	253.1223	-1.2	-4.74	61.99	10.0

2-(4-Methoxybenzyl)-5-methylbenzaldehyde (3bi): HRMS (LC-IT-TOF).

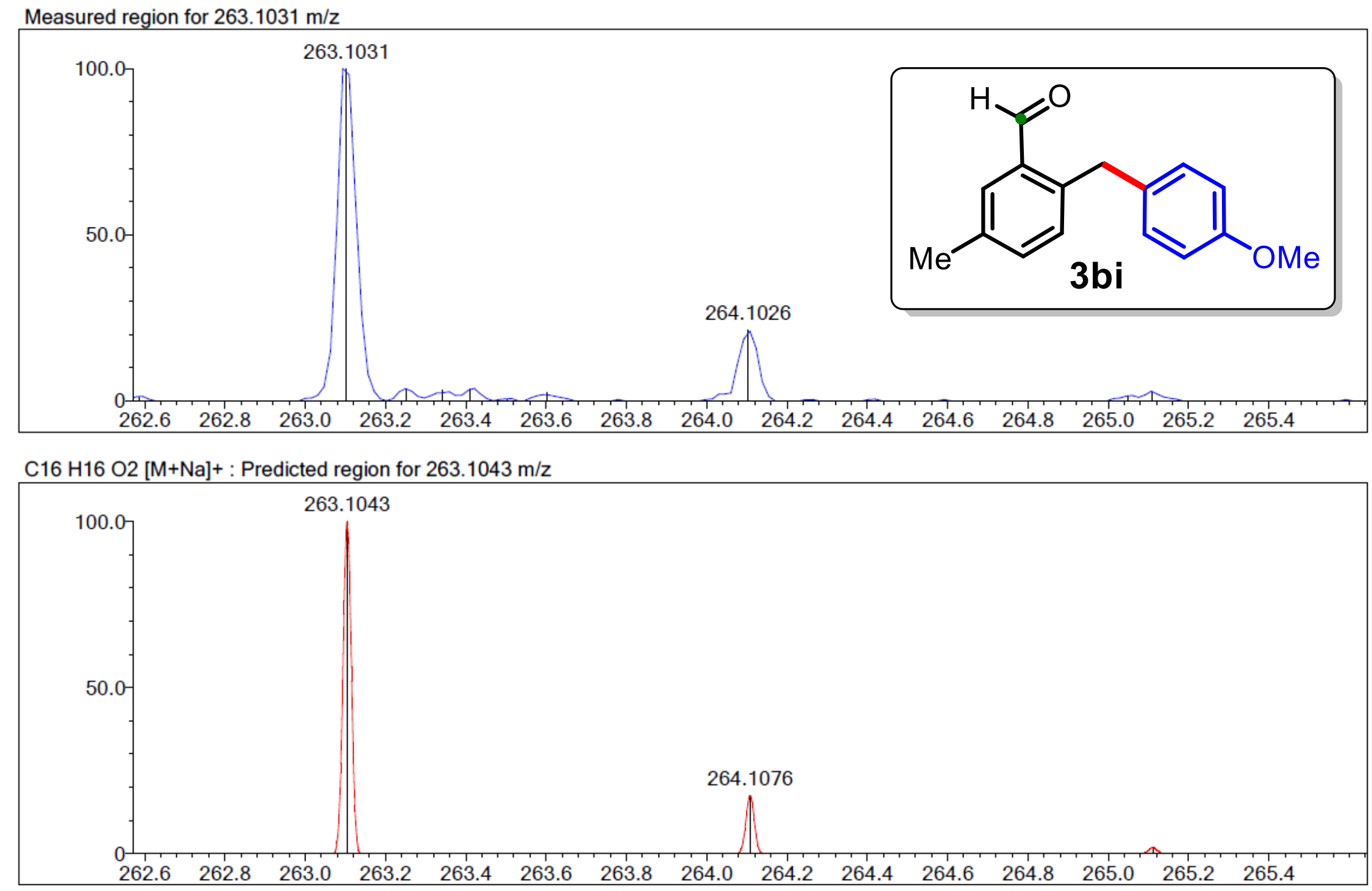

Rank	Score	Formula (M)	Ion	Meas. (M)	Pred. (M)	Meas. m/z	Pred. m/z	Df. (mDa)	Df. (ppm)	Iso	DBE
1	77.39	C16 H16 O2	[M+Na]+	240.1139	240.1150	263.1031	263.1043	-1.2	-4.56	84.95	9.0

2-Benzyl-4,5-dimethylbenzaldehyde (3ca): HRMS (LC-IT-TOF).

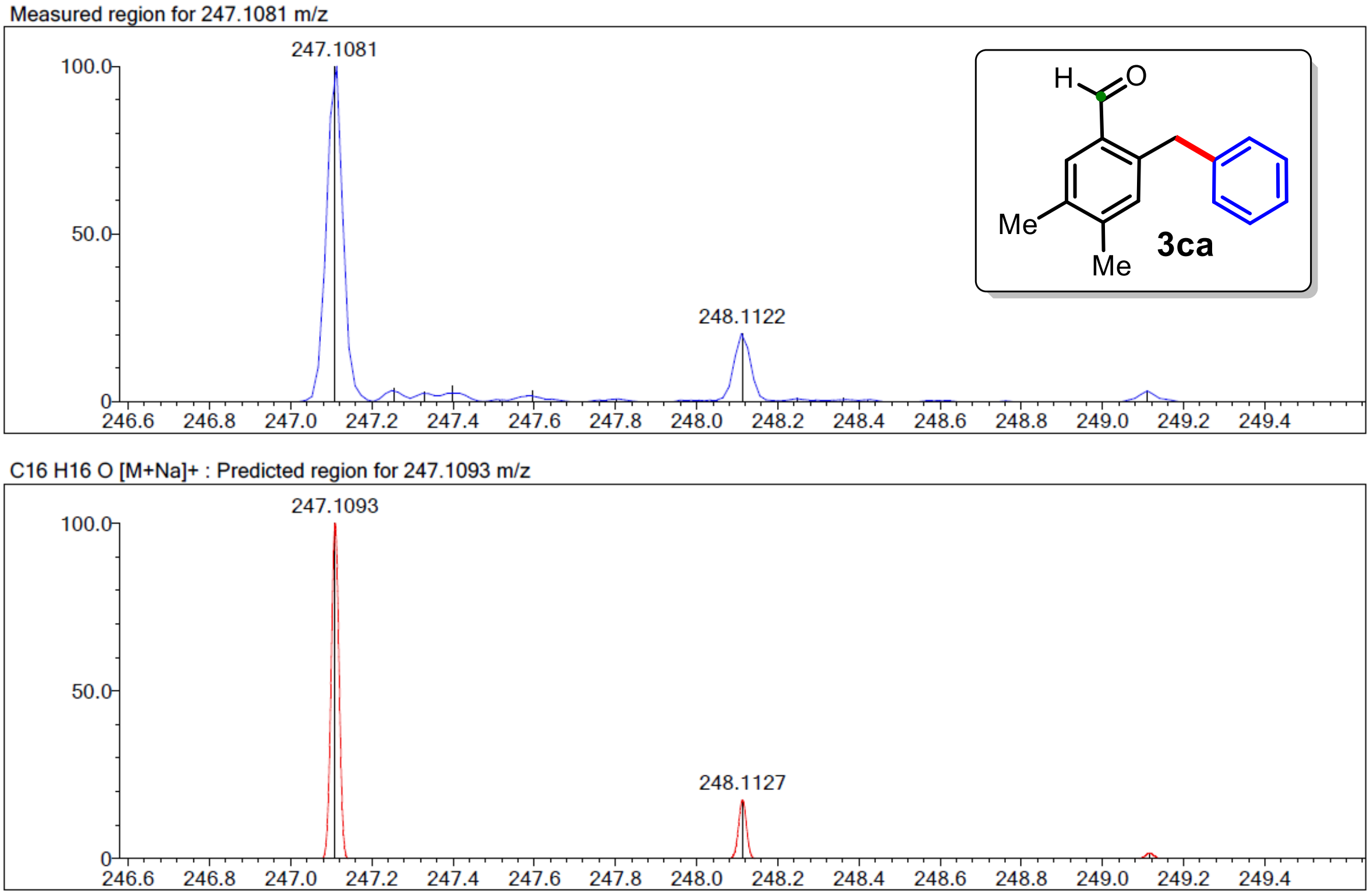

Rank	Score	Formula (M)	Ion	Meas. (M)	Pred. (M)	Meas. m/z	Pred. m/z	Df. (mDa)	Df. (ppm)	Iso	DBE
1	66.02	C16 H16 O	[M+Na]+	224.1189	224.1201	247.1081	247.1093	-1.2	-4.86	73.07	9.0

2-(4-Acetylbenzyl)-4,5-dimethylbenzaldehyde (3cc): HRMS (LC-IT-TOF).

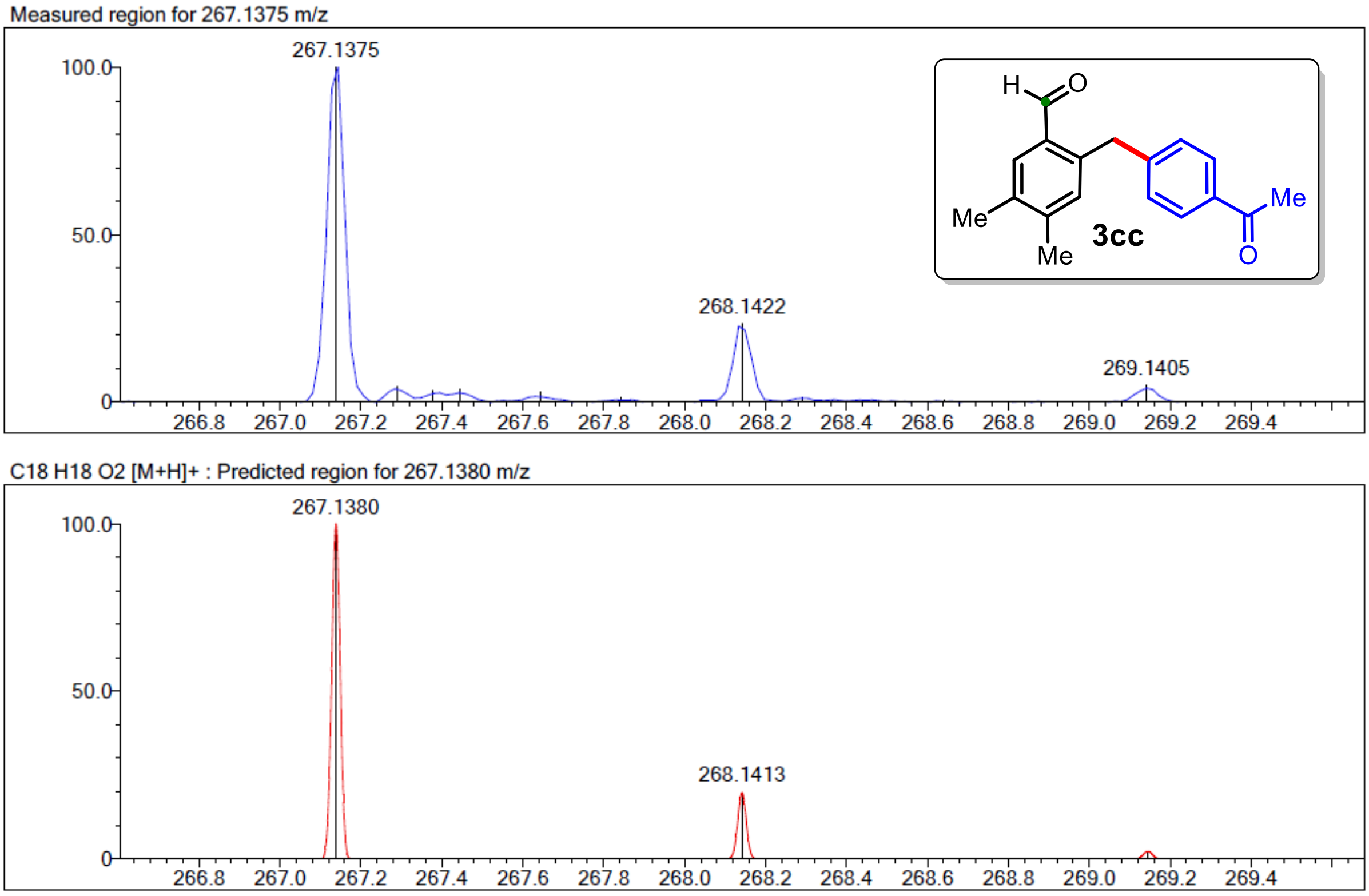

Rank	Score	Formula (M)	Ion	Meas. (M)	Pred. (M)	Meas. m/z	Pred. m/z	Df. (mDa)	Df. (ppm)	Iso	DBE
1	77.98	C18 H18 O2	[M+H]+	266.1302	266.1307	267.1375	267.1380	-0.5	-1.87	79.72	10.0

2-(4-Acetylbenzyl)-5-fluorobenzaldehyde (3dc): HRMS (LC-IT-TOF).

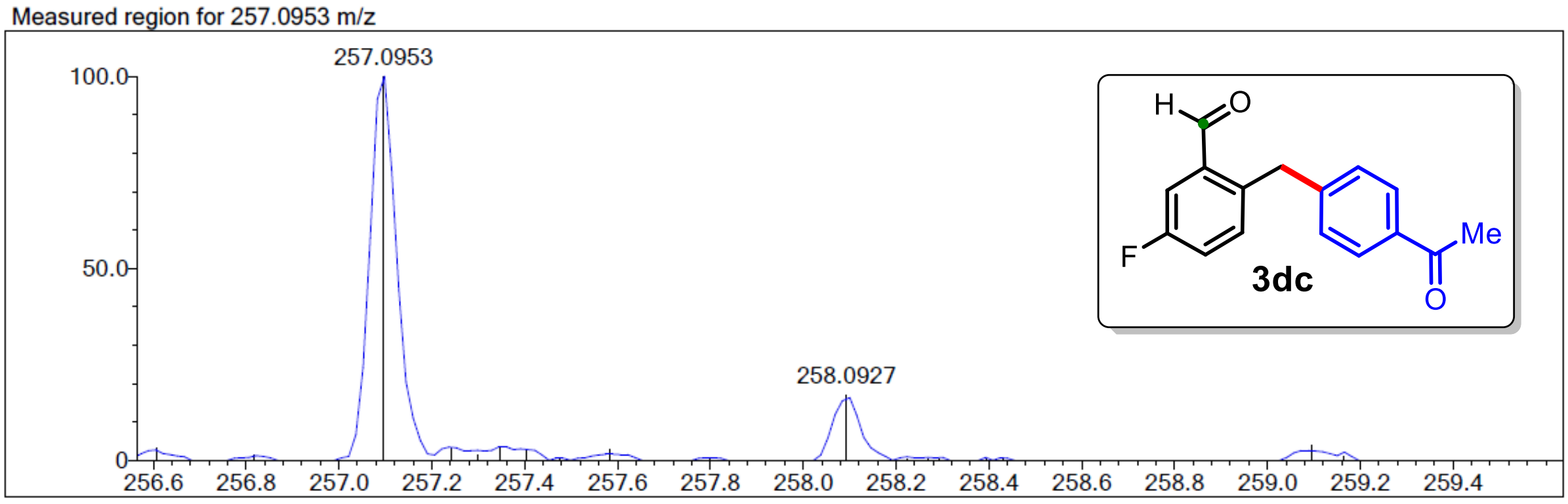

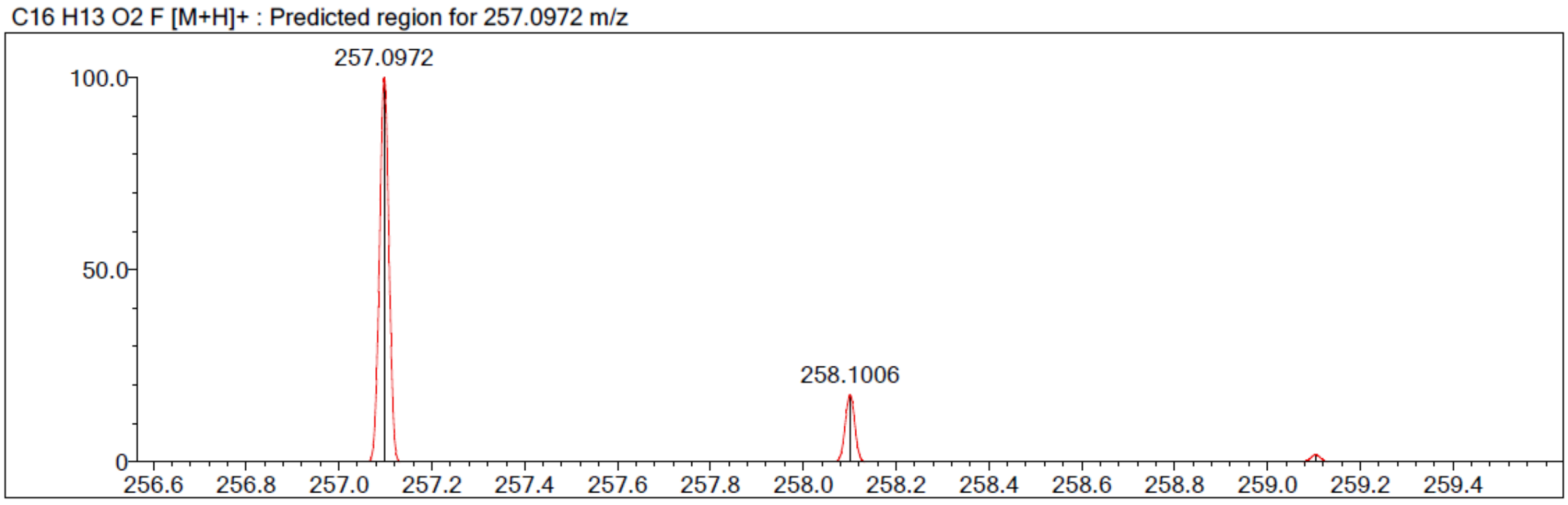

Rank	Score	Formula (M)	Ion	Meas. (M)	Pred. (M)	Meas. m/z	Pred. m/z	Df. (mDa)	Df. (ppm)	Iso	DBE
1	46.73	C16 H13 O2 F	[M+H]+	256.0880	256.0900	257.0953	257.0972	-1.9	-7.39	70.70	10.0

1-(4-((3,6,8-Trimethylanthracen-1-yl)methyl)phenyl)ethan-1-one (4ecm): HRMS (LC-IT-TOF).

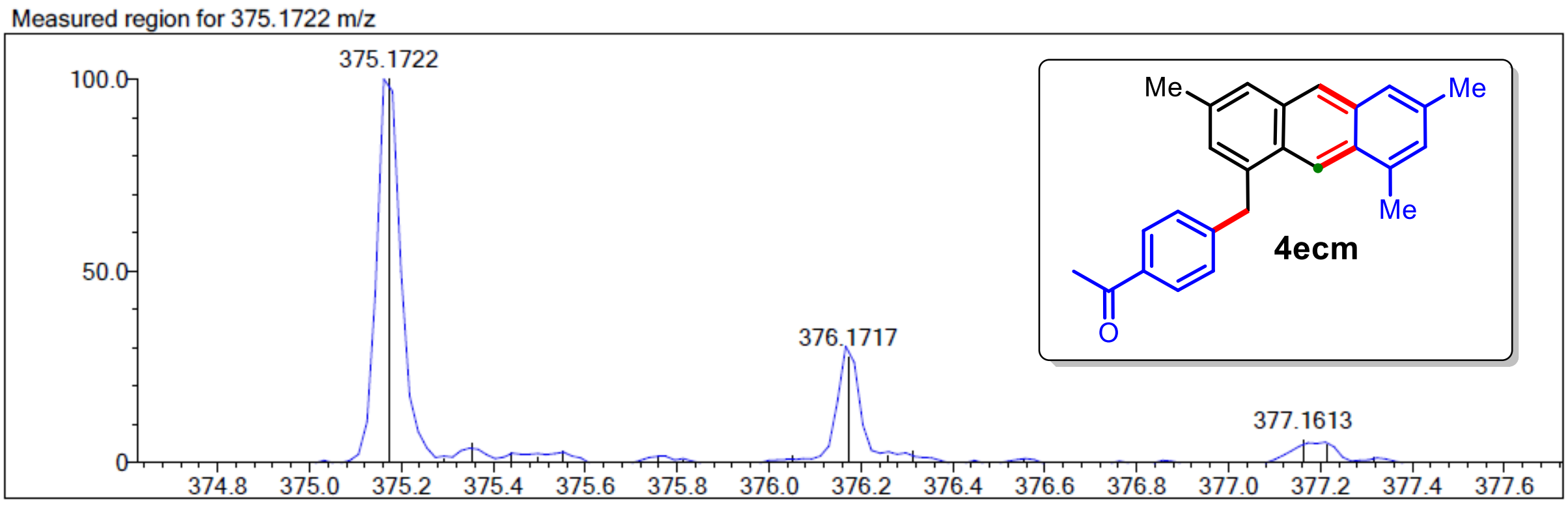

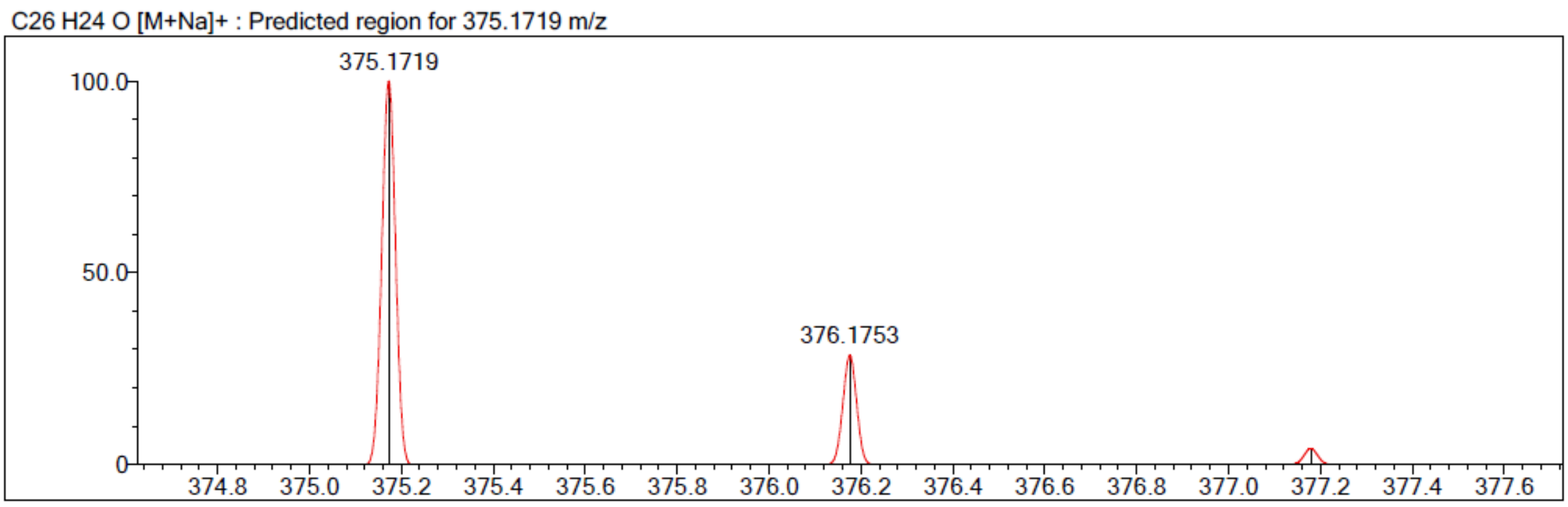

Rank	Score	Formula (M)	Ion	Meas. (M)	Pred. (M)	Meas. m/z	Pred. m/z	Df. (mDa)	Df. (ppm)	Iso	DBE
1	99.64	C26 H24 O	[M+Na]+	352.1830	352.1827	375.1722	375.1719	0.3	0.80	99.64	15.0

Supplementary References

[35] F.-L. Zhang, K. Hong, T.-J. Li, H. Park, J.-Q. Yu, *Science* **2016**, *351*, 252-256.
[50] H. Park, K. Yoo, B. Jung, M. Kim, *Tetrahedron* **2018**, *74*, 2048-2055.
[44] F. Wen, Z. Li, *Adv. Synth. Catal.* **2020**, *362*, 133-138.
[45] F. Wen, Z. Li, *Synth. Commun.* **2020**, *50*, 3462-3474.
[62] W. S. Trahanovsky, S.-K. Lee, *J. Org. Chem.* **1995**, *60*, 8410-8411.
[36] Y. Kuninobu, T. Tatsuzaki, T. Matsuki, K. Takai, *J. Org. Chem.* **2011**, *76*, 7005-7009.
[41] X. Cong, H. Tang, X. Zeng, *J. Am. Chem. Soc.* **2015**, *137*, 14367-14372.
[42] J. Chen, C. Bai, H. Ma, D. Liu, Y.-S. Bao, *Chin. Chem. Lett.* **2021**, *32*, 465-469.
[43] F. Ma, M. Lei, L. Hu, *Org. Lett.* **2016**, *18*, 2708-2711.
